# Catalytic asymmetric Nakamura reaction by gold(I)/chiral *N*,*Nʹ*-dioxide-indium(III) or nickel(II) synergistic catalysis

**DOI:** 10.1038/s41467-021-23105-z

**Published:** 2021-05-21

**Authors:** Xinyue Hu, Xiaoxue Tang, Xiying Zhang, Lili Lin, Xiaoming Feng

**Affiliations:** Key Laboratory of Green Chemistry & Technology, Ministry of Education, College of Chemistry, Sichuan University, Chengdu, China

**Keywords:** Asymmetric catalysis, Organic chemistry, Synthetic chemistry methodology

## Abstract

Intermolecular addition of enols and enolates to unactivated alkynes was proved to be a simple and powerful method for carbon-carbon bond formation. Up to date, a catalytic asymmetric version of alkyne with 1,3-dicarbonyl compound has not been realized. Herein, we achieve the catalytic asymmetric intermolecular addition of 1,3-dicarbonyl compounds to unactivated 1-alkynes attributing to the synergistic activation of chiral *N*,*N*′-dioxide-indium(III) or nickel(II) Lewis acid and achiral gold(I) π-acid. A range of β-ketoamides, β-ketoesters and 1,3-diketones transform to the corresponding products with a tetra-substituted chiral center in good yields with good e.r. values. Besides, a possible catalytic cycle and a transition state model are proposed to illustrate the reaction process and the origin of chiral induction based on the experimental investigations.

## Abstract (web summary)

Although enols and enolates addition to unactivated alkynes is used for carbon-carbon bond modification a catalytic asymmetric alkyne with 1,3-dicarbonyl compound has been elusive. Here, the authors achieve this using the synergistic activation of chiral *N*,*N*′-dioxide-indium(III) or nickel(II) Lewis acid and achiral gold(I) π-acid.”

## Introduction

**Fig. 1:**
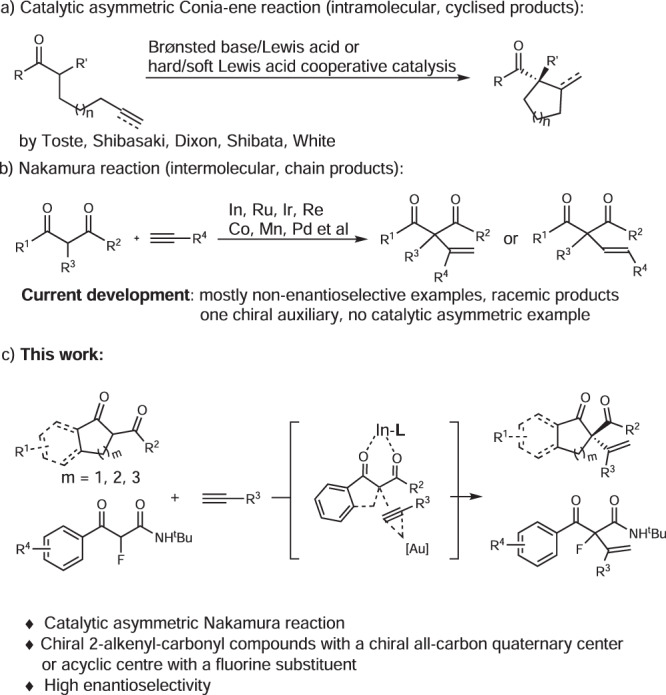
The catalytic asymmetric addition of 1,3-dicarbonyl compounds to alkynes. **a** Catalytic asymmetric Conia-ene reaction. **b** Development of Nakamura reaction. **c** Our strategies for the catalytic asymmetric Nakamura reaction.

The addition of carbonyl compounds without prior enolate formation to unactivated alkynes is an attractive and atom economical method for carbon–carbon bond formation^1^. It results in the introduction of a vinyl substituent to vicinal position of carbonyl groups, possessing an important role in organic synthesis of natural products and drugs^2–5^. The intramolecular type, which is known as the Conia-ene reaction, generating cycloalkene derivatives, has achieved significant progress. Besides the well-developed non-enantioselective systems^6–11^, catalytic asymmetric Conia-ene reactions have already been realized by synergistic hard/soft Lewis acid catalysts (e.g., Pd/Yb, Yb/Zn, Ag/La, Ag/Fe)^12–15^, Lewis basic amine/Lewis acid catalysts (e.g., Cu, Ag-based)^16–19^, and Brønsted basic amine/Lewis acid catalyst (B(C_6_F_5_)_3_/Zn/PMP)^20^. In contrast, the intermolecular reaction of 1,3-dicarbonyl compounds to unactivated 1-alkynes (Nakamura reaction) was less developed. Such a process is unviable because of the unfavorable thermodynamics that there is a high-lying LUMO of an unactivated alkyne compared to the HOMO of 1,3-dicarbonyl compounds^21,22^. In 2003, Nakamura et al. documented an indium-catalyzed addition of 1,3-dicarbonyl compounds to unactivated 1-alkynes^23^, providing an efficient synthetic route to form 2-alkenyl-1,3-dicarbonyl compounds from abundant carbon alkynes sources. After that, In(III)^24–28^, Re(I)^29–31^, Ir(I)^32^, Pd(0)^33^, Co(II)^34^, Mn(I)^35,36^, and Ru(I)-(III)^37–39^ catalytic systems were discovered, all of which were racemic reports except for only one example using substrates with chiral auxiliary^40^. All the above reports, the dicarbonyl compounds and alkynes need to be activated simultaneously. Beyond that, the Shi group reported a synergistic Au(I)/Ga(III) catalysis in Nakamura reaction^41^, in which Au(I) activated the alkynes whereas Ga(III) enhanced the acidity of the 1,3-dicarbonyl compounds^42,43^, affording racemic 2-alkenyl-1,3-dicarbonyl products. Generally, all the Nakamura reactions were still limited to racemic examples (Fig. 1).

Therefore, developing an efficient catalytic system to realize the asymmetric version of the Nakamura reaction is challenging but highly desirable.

Bimetallic catalysis is also promising in asymmetric catalysis^44–46^. However, one of the perceived challenges is that two distinct metals might competitively coordinate with the ligand, as well as potentially affect each other’s catalytic cycles. Recently, chiral *N*,*Nʹ*-dioxides/hard Lewis acid complexes developed by our group were found to be good partners with soft metals^47–51^ in relay catalysis systems. We envisioned that *N*,*Nʹ*-dioxide/Lewis acid complex could also be applied to synergistic catalyst system.

In this work, we developed a gold(I)/chiral *N*,*N’*-dioxide-indium(III) or nickel(II) synergistic catalyst system to realize the catalyic asymmetric Nakamura reaction of unactivated 1-alkynes with β-ketoamides, β-ketoesters, and 1,3-diketones in good reactivity and enantioselectivity. Mechanism study elucidates the process of the reaction and origin of chiral induction.

## Results

### Optimization of the reaction conditions

Indanone-derived β-ketoamide **1a** and phenylacetylene **2a** were selected as the model substrates to conduct our research. First, several cooperative catalytic systems, which showed good ability in catalytic enantioselective Conia-ene reaction, including Pd(II)/Yb(III) dual catalyst system, Zn(II)/Yb(III) catalyst system, and amine–silver system, were investigated^13,16,19^. But all of them gave only trace amount of product without enantioselectivities even rising the reaction temperature to 70 °C (Table 1, entries 1–3). Then chiral *N*,*N’*-dioxide ligand-metal complexes were chosen as the activators of ketoamides, in connection with AuCl∙PPh_3_/AgOTf for the activation of 1-alkyne. First, Sc(OTf)_3_ was used to coordinate with chiral *N*,*N’*-dioxide **L-PiEt**_**2**_ to promote the reaction under air atmosphere, the byproduct **3bb** was obtained as the main product along with the desired product **3aa** in 11% yield with 60:40 e.r. (entry 4). Further research showed that the reaction could possess efficiency in an absolute anaerobic condition, delivering the product **3aa** in 92% yield with 60:40 e.r. (entry 5). Then Ga(OTf)_3_ that showed efficient catalytic activity in Shi’s report^41^ was used to coordinate with chiral *N*,*N’*-dioxide **L-PiEt**_**2**_ to promote the reaction; however, only trace of product **3aa** was obtained (entry 6). To our delight, In(OTf)_3_ could improve the reaction activity greatly and deliver the desired product with 62:38 e.r. (entry 7). The ligand **L-TQ-**(*S*)**-EPh** derived from *S*-tetrahydroisoquinoline acid decreased the e.r. greatly (entry 8). To improve the enantioselectivity, other conditions were carefully studied. Changing the *N*,*N’*-dioxide ligand to **L-PiEt**_**2**_**Me**, which has ethyl groups at *ortho*-positions and methyl group at *para*-position of aniline, the yield could be improved to 99% (entry 9). Moreover, the addition of trace amount of H_2_O (entry 10) and increasing the amount of ligand **L-PiEt**_**2**_**Me** (entry 11) improved the enantioselectivity. The water might be beneficial for formation of the effective catalyst species, as well as beneficial for accelerating the enolization of 1,3-dicarbonyl compounds^52^. Meanwhile, the increasement of ligand might be helpful for the complete coordination with In(OTf)_3_, avoiding the strong background reaction caused by free metal salt. Further exploration showed that the solvent had a great influence on the reaction, when *para*-xylene was used as the solvent, the desired product was isolated in 98% yield with 90:10 e.r. (entry 12). The enantioselectivity enhanced into 94.5:5.5 e.r. after the concentration of **1a** reduced to 0.067 mol/L by enhancing the amount of solvent (entry 13). The steric hindrance of the ligands on [Au] catalyst was another key factor. Changing the AuCl·PPh_3_ into more sterically hindered XPhosAu(TA)OTf, only trace product could be obtained (entry 14). The reason might be that the bulky X-Phos cause larger steric hindrance between the [Au]-activated 1-alkyne and the chiral Lewis acid-activated 1,3-dicarboyl compound, making the reaction happen more difficultly. In comparison, other indium catalysts of the typical chiral ligands such as Pybox **L3**, Box **L2**, or CPA organocatalyst were used, the product **3aa** was obtained in low yield with poor e.r. value (entries 15–17).

**Table 1:** Optimization of the reaction conditions.


Entry	Variation from the “standard conditions”^a^	Yield^b^ (%)	e.r.^c^
1	cat^1^: **L1**/Pd(OTf)_2_, (10 mol%), cat^2^: Yb(OTf)_3_ (20 mol%), AcOH (10 equiv), mesitylene (5.0 mL), 48 h	n.r.	–
2^d^	cat^1^: **L2**/Zn(OTf)_2_,(1:1.1, 10 mol%) cat^2^:Yb(OTf)_3_ (20 mol%), HFIP (1 equiv), mesitylene (1.0 mL), 70 °C	Trace	50:50
3	cat^1^: AgOTf, (2.5 mol%), cat^2^: **L4** (20 mol%), TFA (20 mol%), mesitylene, 48 h	n.r.	–
4^e^	cat^2^: **L-PiEt**_**2**_/Sc(OTf)_3_ (1:1), DCE	11	60:40
5	cat^2^: **L-PiEt**_**2**_/Sc(OTf)_3_ (1:1), DCE	92	60:40
6	cat^2^: **L-PiEt**_**2**_/Ga(OTf)_3_ (1:1), DCE	Trace	–
7	cat^2^: **L-PiEt**_**2**_/In(OTf)_3_ (1:1), DCE	98	62:38
8	cat^2^: **L-TQ-**(*S*)**-EPh**/In(OTf)_3_, (1:1), DCE	94	56.5:43.5
9	cat^2^: **L-PiEt**_**2**_**Me**/In(OTf)_3_ (1:1), DCE	99	63:37
10	cat^2^: **L-PiEt**_**2**_**Me**/In(OTf)_3_ (1:1), H_2_O (2 μL), DCE	99	65:35
11	H_2_O (2 μL), DCE	98	70:30
12	H_2_O (2 μL)	98	90:10
13	H_2_O (2 μL), *p*-xylene (1.5 mL)	98	94.5:5.5
14	cat^1^: XPhosAu(TA)OTf/AgOTf, H_2_O (2 μL), *p*-xylene (1.5 mL)	Trace	–
15	cat^2^: **L3**/In(OTf)_3_, *p*-xylene (1.5 mL)	13	47:53
16	cat^2^: **L2**/In(OTf)_3_, *p*-xylene (1.5 mL)	5	50:50
17	cat^2^: **L5**, *p*-xylene (1.5 mL)	8	50:50

^a^Standard conditions: cat^1^ = AuCl·PPh_3_/AgOTf (1:1, 5 mol%), cat^2^ = In(OTf)_3_/**L-PiEt**_**2**_**Me** (1:1.2, 10 mol%), **1a** (0.10 mmol), and **2a** (2.0 equiv) in *p*-xylene (0.5 mL) under N_2_ atmosphere at 50 °C for 24 h.

^b^Yield of isolated product **3aa**.

^c^Determined by chiral HPLC analysis.

^d^4 Å MS (50 mg) for 72 h.

^e^Under air atmosphere.

### Substrate scope of the reaction about β-ketoamides

**Fig. 2:**
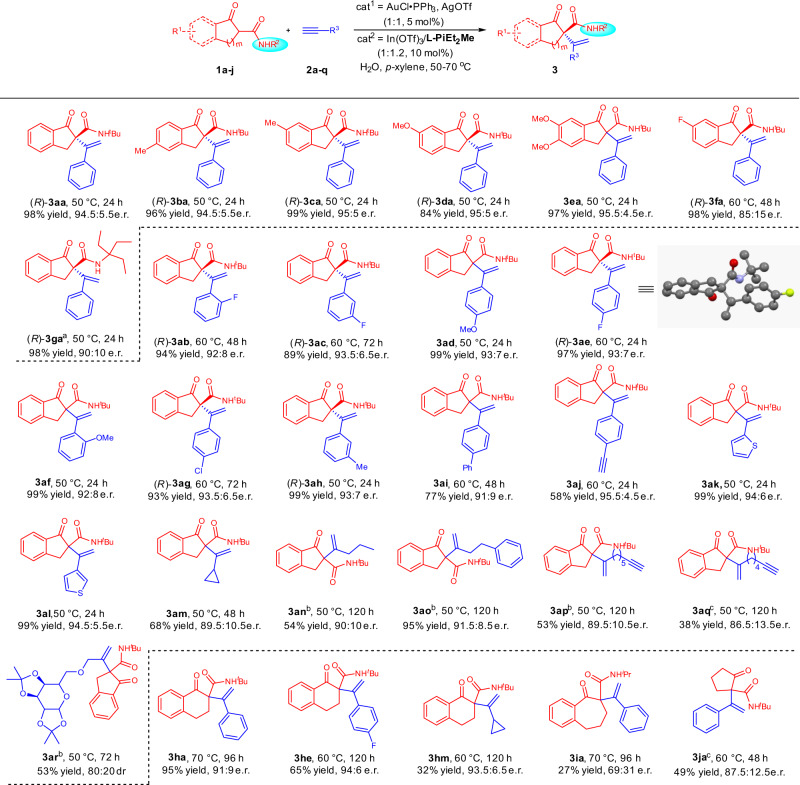
Substrate scope of the reaction about β-ketoamides. Unless otherwise noted, all reactions were carried out, AuCl∙PPh_3_/AgOTf (1:1, 5 mol%), In(OTf)_3_/**L-PiEt**_**2**_**Me** (1:1.2, 10 mol%), **1** (0.10 mmol) and **2** (2.0 equiv), H_2_O (2 μL) as additive in *p*-xylene (1.5 mL) at 50–70 °C for 24–120 h. Isolated yields. The e.r. values were determined by HPLC analysis on chiral column. ^a^**L-PiEt**_**2**_ was used as ligand. ^b^3.0 equiv of **2** was used. ^c^**L-PiMe**_**3**_ was used as ligand.

With the optimized reaction conditions in hand (Table 1, entry 13), the substrate scope was then evaluated (Fig. 2). A variety of ketoamides **1** derived from 1-indanones with different substituents were tested. Substrates with electron-donating groups exhibited excellent yields and enantioselectivities (**3ba**–**3ea**) at 50 °C. Substrate **1f** bearing an electron-withdrawing group transformed to the desired product **3fa** in 98% yield with 85:15 e.r. at higher temperature (60 °C). With respect to 1-alkynes **2**, when the substituents at the aromatic ring of the phenylacetylenes varied, both steric hindrance and electronic properties had little effect on the reaction (**3ab**–**3ai**). However, substrate 1,4-diethynylbenzene **2j** just delivered the product **3aj** in moderate yield with excellent enantioselectivity. It might be caused by the competitive coordination of the alkyne-bearing product with AuOTf∙PPh_3_. The thienyl-substituted alkynes (**2k** and **2l**) were also suitable. Various aliphatic 1-alkynes (**2m**–**2q**) could also transform to the desired products in good enantioselectivities (**3am**–**3aq**); however, the yield was generally moderate. One reason is that an unidentified product generated that might be caused by In(III)-induced olefin isomerization^41^. Importantly, the methodology was applicable to the alkyl-alkyne derived from saccharide **2r**. Next, ring structure of ketoamides was studied. The substrate **1h** derived from 1-tetralone got good results (**3ha**–**3hm**), while **1i** derived from 1-benzosuberone gave much lower yield and e.r.. It might be caused by steric hindrance between methylene of substrate **1i** with AuOTf∙PPh_3_-activated **2a**. Meanwhile, aliphatic substrate **1j** was also tolerated, affording the product **3ja** in moderate yield with good enantioselectivity. The absolute configuration of **3ae** was determined to be *R* by X-ray crystallographic analysis and the absolute configurations of **3aa**–**3ac** and **3ag**–**3ah** were determined to be *R* by comparison of the CD spectra with that of **3ae** (Fig. 2).

### Effect of *N*-protecting group

Other easily removable *N*-protecting groups such as *N*-benzyl or *N*-PMP were investigated by reacting with phenylacetylene (Table 2). The desired products **3ka** and **3la** were obtained in excellent yields but with only 56.5:43.5 e.r. and 72.5:27.5 e.r. under standard conditions. After changing the ligand to **L-TQ-**(*S*)**-EPh** derived from (*S*)-tetrahydroisoquinoline-3-carbonyl acid and (*S*)-phenylethanamine, adjusting the reaction temperature and solvent, the enantioselectivities were improved sharply to 90:10 e.r. and 89:11 e.r., respectively. The possible reason might be that the *N*-protecting group, amide moiety and backbones of the catalyst are included in discrimination of *Re*- and *Si*-face of the 1,3-dicarbonyl compounds. With **L-PiEt**_**2**_**Me** as ligand, the bulky *N*-tertbutylamide moiety could help to shield the *Si*-face of substrate **1a** efficiently. On the contrary, the *N*-benzyl or PMP with smaller steric hindrance showed poor ability to shield the *Si*-face of the 1,3-dicarbonyl compounds, causing the e.r. values of the products decreased sharply in the **L-PiEt**_**2**_**Me**/In(III) system. Changing to the **L-TQ-**(*S*)**-EPh**/In(III) system, the steric hindrance of amide moiety and backbones of the catalyst increased, and the *Si*-face of the 1,3-dicarbonyl compounds could also shield better; therefore, the e.r. values of products increased.

**Table 2:** Effect of *N*-protecting group.


Entry	*R*^2^	Ligand	Yield^a^ (%)	e.r.^b^
1	Bn	**L-PiEt**_**2**_**Me**	99	56.5:43.5
2^c^	(**3ka**)	**L-TQ-**(*S*)**-EPh**	99	90:10
3	PMP	**L-PiEt**_**2**_**Me**	96	72.5:27.5
4^d^	**3la**	**L-TQ-**(*S*)**-EPh**	98	89:11

Unless otherwise noted, all reactions were carried out, AuCl∙PPh_3_/AgOTf (1:1, 5 mol%), In(OTf)_3_/ligand (1:1.2, 10 mol%), **1** (0.10 mmol) and **2a** (2.0 equiv) in para-xylene (1.5 mL) at 50 °C for 24 h.

*Bn* benzyl group, *PMP* *p*-methoxyphenyl group.

^a^Yield of isolated product **3**.

^b^Determined by chiral HPLC analysis.

^c^React at 35 °C for 72 h.

^d^React at 60 °C in toluene (1.5 mL) for 48 h.

### Substrate scope of the reaction about β-ketoesters

**Fig. 3:**
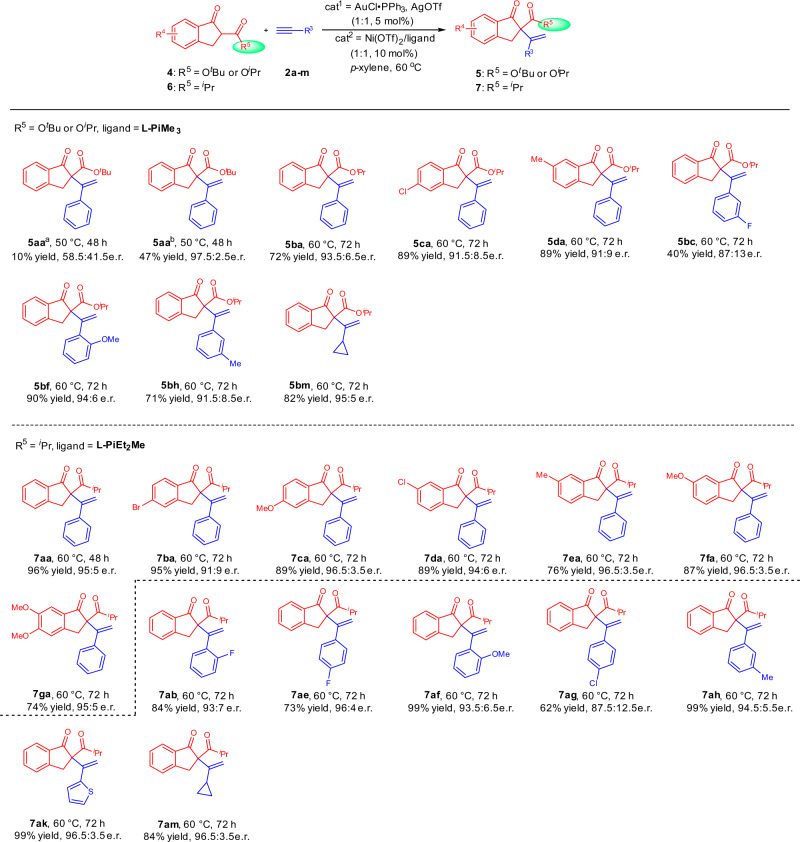
Substrate scope of the reaction about β-ketoesters and 1,3-diketones. Unless otherwise noted, all reactions were carried out, AuCl∙PPh_3_/AgOTf (1:1, 5 mol%), Ni(OTf)_2_/ligand (1:1, 10 mol%), **4** or **6** (0.10 mmol) and **2** (2.0 equiv) in *p*-xylene (1.0 mL) at 60 °C for 48–72 h. Isolated yields. The e.r. values were determined by HPLC analysis on chiral column. ^a^In(OTf)_3_/**L-PiEt**_**2**_**Me** = 1:1.2, 10 mol%, *p*-xylene (1.5 mL), 24 h. ^b^**L-PiMe**_**2**_ was used as ligand.

When β-ketoesters **4a** was applied in the In(OTf)_3_/**L-PiEt**_**2**_**Me** catalytic system, the desired product was obtained in only 10% yield with 58.5:41.5 e.r.. After extensive investigation, including use of Ni(OTf)_2_/**L-PiMe**_**2**_ as catalyst and prolonging the reaction time, the corresponding product **5aa** could be obtained in 47% yield with 97.5:2.5 e.r.. The decomposition of substrate **4a** is responsible for the moderate yield. More stable **4b** with a smaller steric hindrance of ester protecting group could transform to the desired **5ba** in 72% yield with 93.5:6.5 e.r.. Different ketoesters **4** derived from 1-indanones bearing electron-donating group or withdrawing group tolerated well. Moreover, both aliphtic alkynes and aromatic alkynes were suitable in the reaction. The yields were generally good except product **5bc**. The possible reason might be that the electron-withdrawing effect of the fluorine weakened the interaction between gold catalyst and alkyne (Fig. 3).

### Substrate scope of the reaction about 1,3-diketones

1,3-Diketones were next investigated. With Ni(OTf)_2_/**L-PiEt**_**2**_**Me** as catalyst, a variety of 1,3-diketons with no matter electron-donating group or electron-withdrawing group on the C5-position or C6-position transformed to the corresponding products **7aa**–**7ga** in 74–96% yields with 91:9–96.5:3.5 e.r.. Besides, aromatic, aliphatic, and hetero-aromatic 1-alkynes **2** were suitable substrates (Fig. 3).

### Substrate scope limitation

**Fig. 4:**
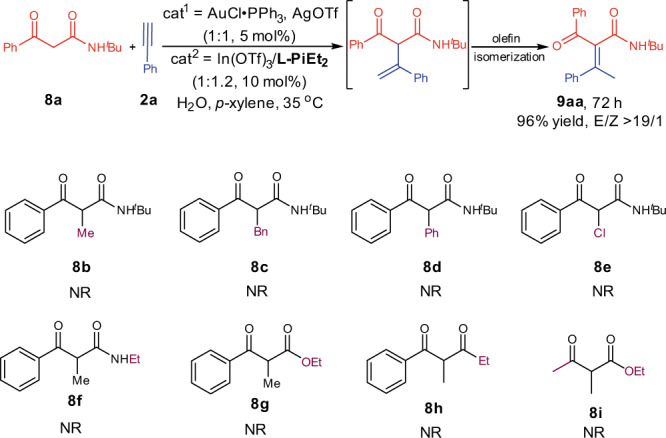
Substrate scope limitation of α-substituted acyclic β-ketoamides. Unless otherwise noted, all reactions were carried out, AuCl∙PPh_3_/AgOTf (1:1, 5 mol%), In(OTf)_3_/**L-PiEt**_**2**_ (1:1.2, 10 mol%), **8** (0.10 mmol) and **2a** (3.0 equiv), H_2_O (2 μL) as additive in *p*-xylene (1.5 mL) at 70 °C for 120 h.

For acyclic β-ketoamide **8a**, which without other substituent on α-position, transformed to thermodynamically stable achiral α,β-conjugated carbonyl product **9aa** through olefin isomerization (Fig. 4). When acyclic β-ketoamides **8b**–**8i** bearing methyl, phenyl, benzyl, or chlorine group on the α-position were used as the nucleophiles, the corresponding products could not be observed. The possible reason might be that the α-substitution on the 1,3-dicarbonyl compounds increased the steric hindrance when the two activated substrates participate in the reaction.

### Substrate scope of α-fluoro substituted acyclic β-ketoamides

**Fig. 5:**
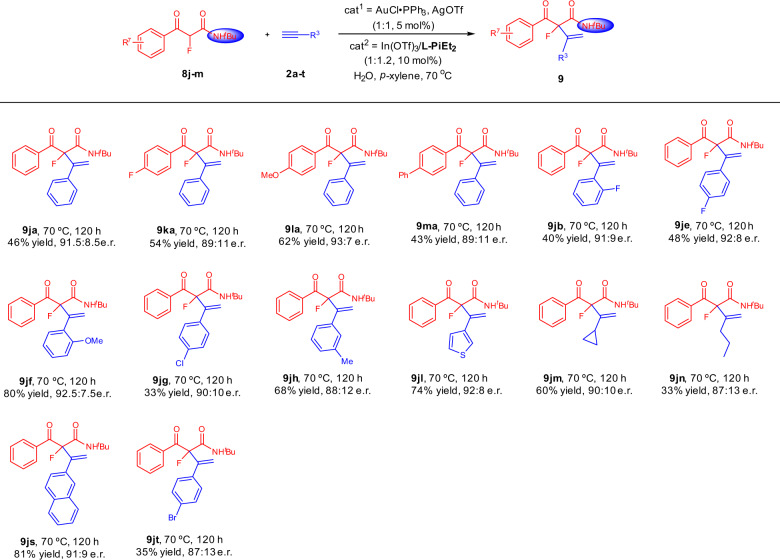
Substrate scope of α-fluoro substituted acyclic β-ketoamides. Unless otherwise noted, all reactions were carried out, AuCl∙PPh_3_/AgOTf (1:1, 5 mol%), In(OTf)_3_/**L-PiEt**_**2**_ (1:1.2, 10 mol%), **8** (0.10 mmol) and **2** (3.0 equiv), H_2_O (2 μL) as additive in *p*-xylene (1.5 mL) at 70 °C for 120 h.

Therefore, α-fluoro substituted **8j** with smaller steric hindrance and stronger acidity of α-proton was evaluated (Fig. 5). Moderate yields with good e.r. could be obtained after adjusting the ligand to **L-PiEt**_**2**_, increasing the reaction temperature and prolonging the reaction time. Electron-donating or electron-withdrawing substitutes on the *para*-position of phenyl ring were tolerated well. Generally, the 1-alkynes **2** with an electron-donating substituent led to better yields than the ones with electron-withdrawing substituents. Compared with the phenylacetylene, the more electron-rich aromatic alkynes like **2l** and **2s** showed better reactivities (**9jl** and **9js**). When aliphatic 1-alkynes **2m** and **2n** were applied to the reaction, the products were delivered in moderate yields with good e.r. values.

### Mechanism investigation

**Fig. 6:**
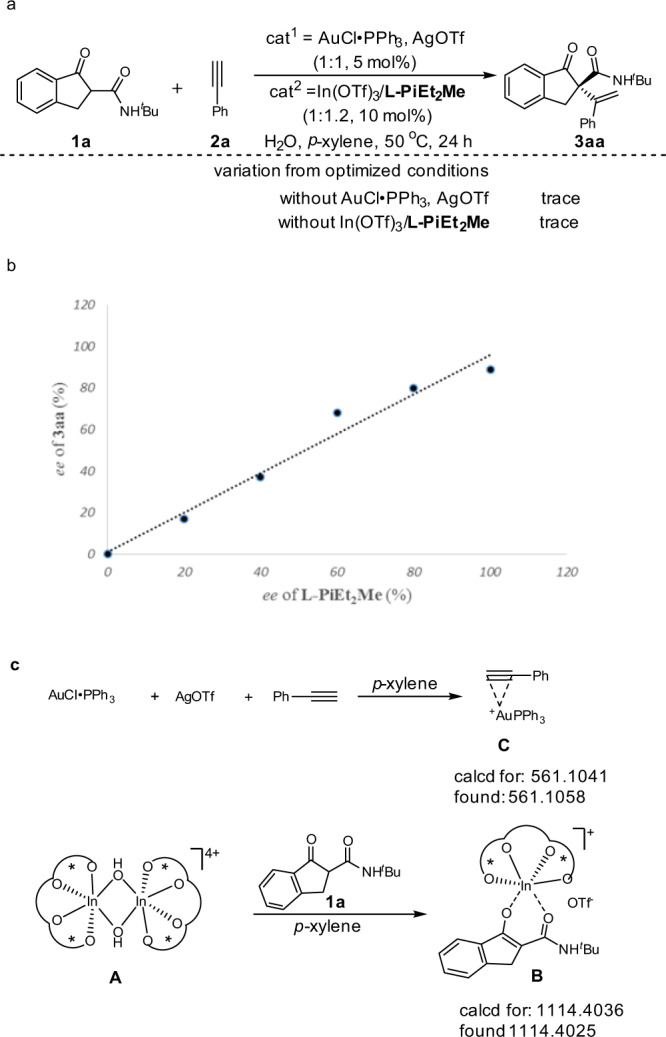
Mechanism investigation. **a** Control experiments for reaction conditions. **b** The relationship between the ee value of **L-PiEt**_**2**_**Me** and **3aa**. **c** The ESI-TOF analysis of intermediate **B** and **C**.

Next, the reaction mechanism was investigated (Fig. 6). Some control experiments were carried out (Fig. 6a). In the absence of AuCl∙PPh_3_/AgOTf or In(OTf)_3_/**L-PiEt**_**2**_**Me**, only trace amount of the product **3aa** was detected, which indicates that the two catalysts work cooperatively. *N*,*Nʹ*-dioxide/In(OTf)_3_ crystal structure obtained in our previous study^49^ showed that a OH-bridged dinuclear indium complex forms in the presence of H_2_O, in which *N*,*Nʹ*-dioxide coordinates to In(III) in a tetradentate manner. Nevertheless, the investigation of relationship between the e.e. value of **L-PiEt**_**2**_**Me** and that of **3aa** showed a clear linear effect (Fig. 6b), implying that the active catalytic species is likely to be the mixture of In(OTf)_3_ and **L-PiEt**_**2**_**Me** in a 1:1 ratio. The OH anion generated from the water in situ preparation of the chiral indium catalyst might act as a base to accelerate the enolization of 1,3-dicarbonyl compounds. In addition, the M^+^ peak (found: 561.1058), which corresponded to a 1:1 complex **C** of [Au·PPh_3_]^+^ and phenylacetylene **2a**, was detected by ESI-TOF analysis in the positive-ion mode. The mixture of **L-PiEt**_**2**_**Me**, In(OTf)_3_, and **1a** (1:1:1) in *p*-xylene displaying an ion at m/z 1114.4025 ([**L-PiEt**_**2**_**Me**+In^3+^+OTf^−^+**1a**-H^**+**^] m/z calcd 1114.4036) suggested that enolized **1a** coordinates to the catalyst in a 1:1 molecular ratio (Fig. 6c), which is consistent with our non-linear effect.

### Proposed catalytic cycle and transition-state model

**Fig. 7:**
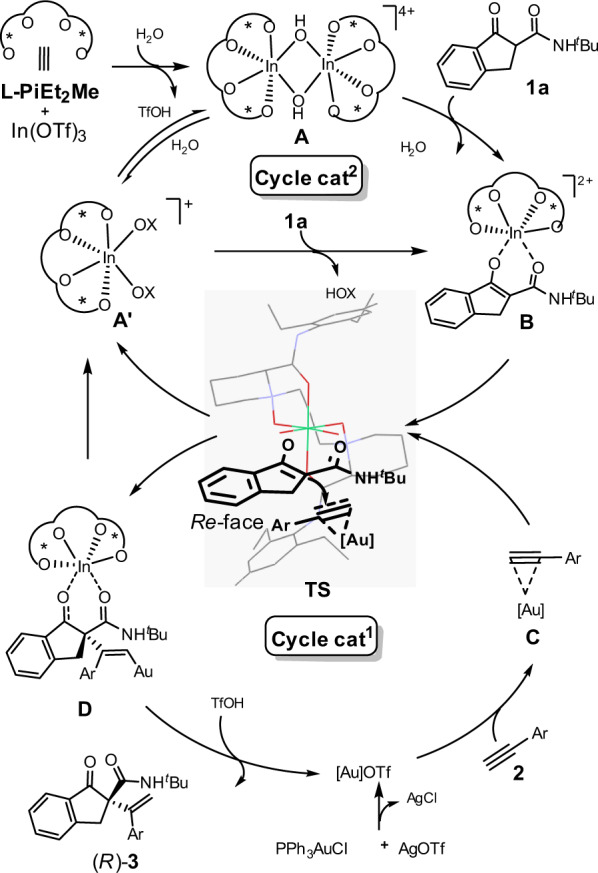
Proposed catalytic cycle and transition-state model. The in situ formed chiral *N*,*N*′-dioxide-indium(III) Lewis acid actives the 1,3-dicarbonyl compounds (intermediate **B**) and achiral gold(I) π-acid actives the alkyne (intermediate **C**) synergistically; the two intermediates reacts following subsequent protonation, giving the desired product **3** and releases the two catalysts.

Based on the above analysis and previous work, a catalytic cycle with a possible transition state is proposed. As illustrated in Fig. 7, in **L-PiEt**_**2**_**Me**/In(OTf)_3_ cycle, initially, the tetradentate **L-PiEt**_**2**_**Me** coordinates to In^III^ to form a six-coordinate octahedral geometry complex **A’** and dimer **A**. When ketoamide **1a** was added, the basic anion of the catalytic species accelerates the deprotonation process, and the enol ion of **1a** coordinates tightly to chiral indium(III) center through two oxygens to form the carbanion nucleophile intermediate **B**. On the other hand, as for [Au] cycle, the [Au]OTf, which is the more reactive species, would bind to the π-bond of 1-alkyne **2** in an unsymmetrical fashion to form species **C**. The intermediate **C** then reacts with the complex **B** to form the Au/In stabilized reactive intermediate **TS**, which is the origin of the stereoselectivity. Due to the *Si*-face of β-ketoamides, **1a** is effectively shielded by the amide moiety of the catalyst, with the assistance of *N*-protecting group on β-ketoamide and piperidine ring on the ligand, cat^1^-activated π-bond of **2a** approaches preferably from the *Re*-face to undergo an energetically favorable C–C bond forming reaction, forming the complex **D** with *R* absolute configuration at the newly formed stereogenic center. Subsequent protonation of **D** gives the desired product **3** and releases the two catalysts.

## Discussion

An efficient catalytic asymmetric Nakamura reaction of β-ketoamides, β-ketoesters, and 1,3-diketones with unactivated 1-alkynes is realized by developing a bimetallic synergistic catalysis. The combination of π-acid gold(I)/chiral *N*,*Nʹ*-doxide-indium(III) or nickel(II) complex enables the activation of alkyne and the efficiency and stereoselectivity of nucleophile. The steric hindrance of α-substituent on 1,3-dicarbonyl compounds and hard Lewis acid are found crucial factors for the reactivity of the reaction. In addition, the substituent of 1,3-dicarbonyl compounds, the amide moiety, and backbones of the catalyst are found to affect the enantioselectivity of the reaction greatly. A possible catalytic cycle with a transition-state model was proposed to elucidate the process of the reaction and origin of chiral induction. Further studies on hetero bimetallic synergistic or relay catalysis are underway in our laboratory.

## Methods

### Typical procedure for cyclic β-ketoamides involved in catalytic asymmetric reaction

A mixture of AuCl∙PPh_3_ (5 mol%, 2.5 mg), AgOTf (5 mol%, 1.3 mg), In(OTf)_3_ (10 mol%, 5.6 mg), **L-PiEt**_**2**_**Me** (12 mol%, 7.4 mg), and the *N*-(tert-butyl)−1-oxo-2,3-dihydro-1*H*-indene-2-carboxamide **1a** (0.10 mmol) was added to a test tube under N_2_ atmosphere. Then, anhydrous *para*-xylene (1.5 mL) was added and the mixture was stirred at 30 °C for 30 min. Subsequently, H_2_O (1.1 equiv, 2.0 μL) was added under stirring at 30 °C. Five minutes later, phenylacetylene **2a** (2.0 equiv, 22 μL) was added at 50 °C, and the reaction mixture continued stirring at 50 °C for 24 h. The residue was purified by flash chromatography on silica gel (petroleum ether/ethyl acetate = 15:1, v/v) to afford the desired product **3aa** (98% yield, 94.5:5.5 e.r.).

### Typical procedure for β-ketoesters involved in catalytic asymmetric reaction

A mixture of AuCl∙PPh_3_ (5 mol%, 2.5 mg), AgOTf (5 mol%, 1.3 mg), Ni(OTf)_2_ (10 mol%, 3.6 mg), **L-PiMe**_**3**_ (10 mol%, 5.6 mg), and the isopropyl 1-oxo-2,3-dihydro-1*H*-indene-2-carboxylate **4b** (0.10 mmol) was added to a test tube under N_2_ atmosphere. Then, anhydrous *para*-xylene (1.0 mL) was added and the mixture was stirred at 30 °C for 30 min. Subsequently, phenylacetylene **2a** (2.0 equiv, 22 μL) was added at 60 °C, and the reaction mixture continued stirring at 60 °C for 72 h. The residue was purified by flash chromatography on silica gel (petroleum ether/ethyl acetate = 20:1, v/v) to afford the desired product **5ba** (72% yield, 93.5:6.5 e.r.).

### Typical procedure for 1,3-diketones involved in catalytic asymmetric reaction

A mixture of AuCl∙PPh_3_ (5 mol%, 2.5 mg), AgOTf (5 mol%, 1.3 mg), Ni(OTf)_2_ (10 mol%, 3.6 mg), **L-PiEt**_**2**_**Me** (10 mol%, 6.2 mg), and the 2-isobutyryl-2,3-dihydro-1*H*-inden-1-one **6a** (0.10 mmol) was added to a test tube under N_2_ atmosphere. Then, anhydrous *para*-xylene (1.0 mL) was added and the mixture was stirred at 30 °C for 30 min. Subsequently, phenylacetylene **2a** (2.0 equiv, 22 μL) was added at 60 °C, and the reaction mixture continued stirring at 60 °C for 48 h. The residue was purified by flash chromatography on silica gel (petroleum ether/ethyl acetate = 25:1, v/v) to afford the desired product **7aa** (96% yield, 95:5 e.r.).

### Typical procedure for acyclic β-ketoamides involved in catalytic asymmetric reaction

A mixture of AuCl∙PPh_3_ (5 mol%, 2.5 mg), AgOTf (5 mol%, 1.3 mg), In(OTf)_3_ (10 mol%, 5.6 mg), **L-PiEt**_**2**_ (12 mol%, 7.1 mg), and the *N*-(tert-butyl)−2-fluoro-3-oxo-3-phenylpropanamide **8j** (0.10 mmol) was added to a test tube under N_2_ atmosphere. Then, anhydrous *para*-xylene (1.5 mL) was added and the mixture was stirred at 30 °C for 30 min. Subsequently, H_2_O (1.1 equiv, 2.0 μL) was added under stirring at 30 °C. Five minutes later, phenylacetylene **2a** (3.0 equiv, 33 μL) was added at 70 °C, and the reaction mixture continued stirring at 70 °C for 120 h. The residue was purified by flash chromatography on silica gel (petroleum ether/ethyl acetate = 6:1, v/v) to afford the desired product **9ja** (46% yield, 91.5:8.5 e.r.).

## Supplementary information

Supplementary Information

## Data Availability

The X-ray crystallographic coordinate for structure **3ae** reported in this study has been deposited at the Cambridge Crystallographic Data Centre (CCDC), under deposition number 1964558. The data can be obtained free of charge from The Cambridge Crystallographic Data Centre via https://www.ccdc.cam.ac.uk/structures/. All other data are available from the corresponding author upon reasonable request.
