# Supplementary material for: Catalytic asymmetric Nakamura reaction by gold(I)/chiral N,Nʹ-dioxide-indium(III) or nickel(II) synergistic catalysis
Source: Nat Commun. 2021 May 21;12:3012. doi: 10.1038/s41467-021-23105-z (PMC8140141; doi:10.1038/s41467-021-23105-z)
Supplement: Supplementary file 1 — Supplementary Information [file 41467_2021_23105_MOESM1_ESM.pdf]

Supplementary Information

Catalytic asymmetric Nakamura reaction by gold(I)/chiral *N,N′*-dioxide-indium(III) or nickel(II) synergistic catalysis

Hu et all

Contents

1. General remarks 3

2. Typical procedure for the catalytic asymmetric reaction 3

3. Supplementary Tables 6

4. Experimental procedure for the scale-up reaction and transformation of the product 14

5. Control experiments 16

6. HRMS analysis and X-ray crystallography data 16

7. The analytical and spectral characterization data of products 21

8. Copies of NMR spectra for products 86

9. Copy of CD spectra in CH_2Cl_2 168

10. Supplementary References...173

Supplementary Method

1. General remarks

^{1}H NMR spectra were recorded on commercial instruments (bruker ASCEND 400 MHz, serial number: 10041818). Chemical shifts were reported in ppm from tetramethylsilane with the solvent resonance as the internal standard ($CDCl_3$, δ = 7.26). Spectra were reported as follows: chemical shift (δ ppm), multiplicity (s = singlet, d = doublet, t = triplet, q = quartet, m = multiplet, dd = doublet of doublets, dt = doublet of triplets), coupling constants (Hz), integration and assignment. $^{13}C\{^{1}H\}$ NMR spectra were collected on commercial instruments (101 MHz) with complete proton decoupling. $^{19}F\{^{1}H\}$ NMR spectra were collected on commercial instruments (376 MHz) with complete proton decoupling. Chemical shifts are reported in ppm from the tetramethylsilane with the solvent resonance as internal standard ($CDCl_3$, δ = 77.06). HRMS was recorded on a commercial apparatus (ESI Source). Enantio ratio (e.r.) were determined by HPLC analysis using the corresponding commercial chiral column as stated in the experimental procedures at 23 ℃ with UV detector at 254 nm. Optical rotations were reported as follows: $[\alpha]^{T}_{D}$ (*c* = g/100 mL, in solvent). Unless otherwise indicated, reagents obtained from commercial sources were used without further purification. Solvents were dried and distilled prior to use according to the standard methods. Para-xylene was purchased from 3A without further purification. $In(OTf)_3$ was purchased from Alfa, $AuCl{\cdot}PPh_3$ was purchased from Aladdin and AgOTf was purchased from Adamas. The chiral *N*,*N'*-dioxide ligands[1], *N*-(tert-butyl)-1-oxo-2,3-dihydro-1*H*-indene-2- carboxamide[2], *N*-(*tert*-butyl)-1-oxo-1,2,3,4-tetrahydronaphthalene-2-carboxamide[3], isopropyl 1-oxo-2,3-dihydro-1*H*-indene-2-carboxylate[4], 2-isobutyryl-2,3-dihydro-1*H*-inden-1-one[5] and *N*-(tert-butyl)-2-fluoro-3-oxo-3-phenylpropanamide[6] were synthesized by the same procedure in the literature.

2. Typical procedure for the catalytic asymmetric reaction

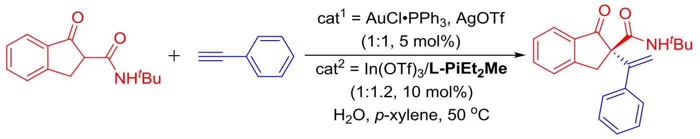

1a **2a** **3aa**

A mixture of AuCl·PPh_3 (5 mol%, 2.5 mg), AgOTf (5 mol%, 1.3 mg), $In(OTf)_3$ (10 mol%, 5.6 mg), **L-PiEt$_2$ Me** (12 mol%, 7.4 mg) and the substrate **1a** (0.10 mmol) were added to a test tube under N_2 atmosphere. Then, anhydrous *para*-xylene (1.5 mL) was added and the mixture was stirred at 30 ℃ for 30 minutes. Subsequently, H_2O (1.1 equiv, 2.0 μL) was added under stirring at 30 ℃. Five minutes later, 1-alkynes **2a** (2.0 equiv, 22μL) was added at 50 °C, and the reaction mixture continued stirring at 50 ℃ for 24 h. The residue was purified by flash chromatography on silica gel (petroleum ether/ethyl acetate = 15:1, v/v) to afford the desired product **3aa** (98% yield, 94.5:5.5 e.r.).

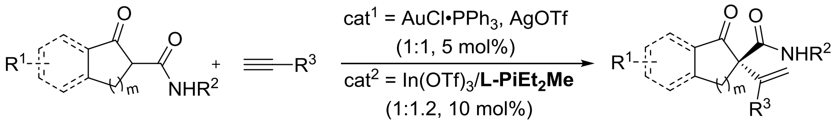

1a-j **2a-q** **3**

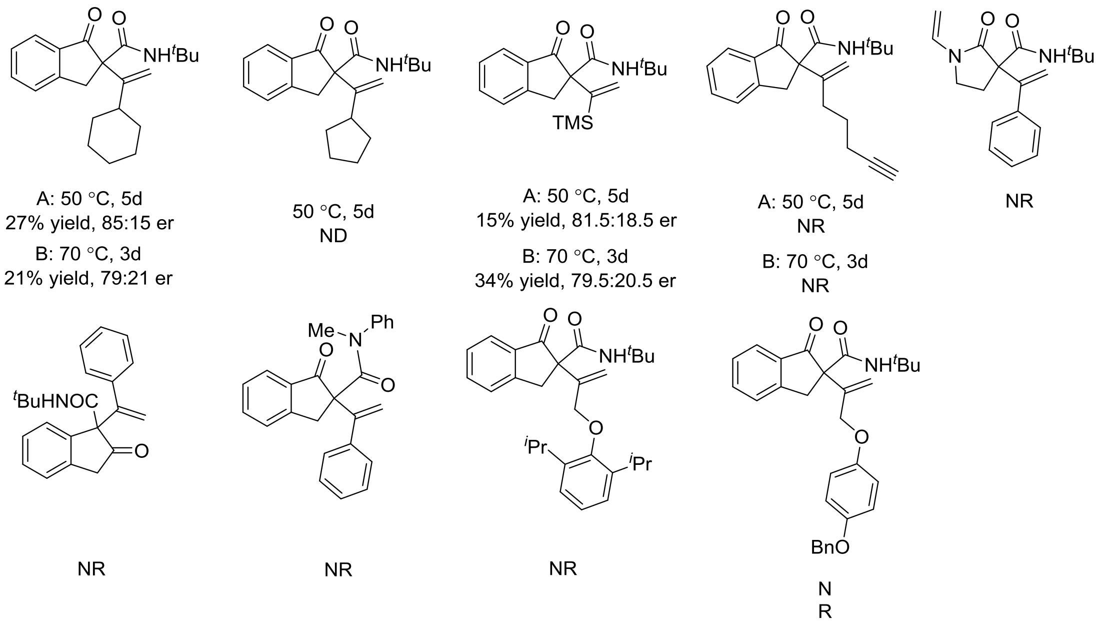

Supplementary Figure 1. Unsuccessful substrate scope of cyclic β-ketoamides.

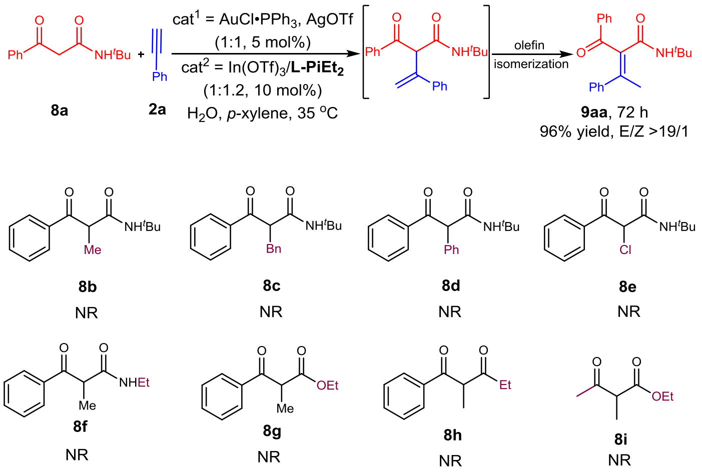

Supplementary Figure 2. Substrate scope limitation of α-substituted acyclic β-ketoamides. Unless otherwise noted, all reactions were carried out, AuCl·PPh_3/AgOTf (1:1, 5 mol%), $In(OTf)_3$/**L-PiEt$_2$** (1:1.2, 10 mol%), **8** (0.10 mmol) and **2a** (3.0 equiv), H_2O (2 μL) as additive in *p*-xylene (1.5 mL) at 70 ℃ for 120 h.

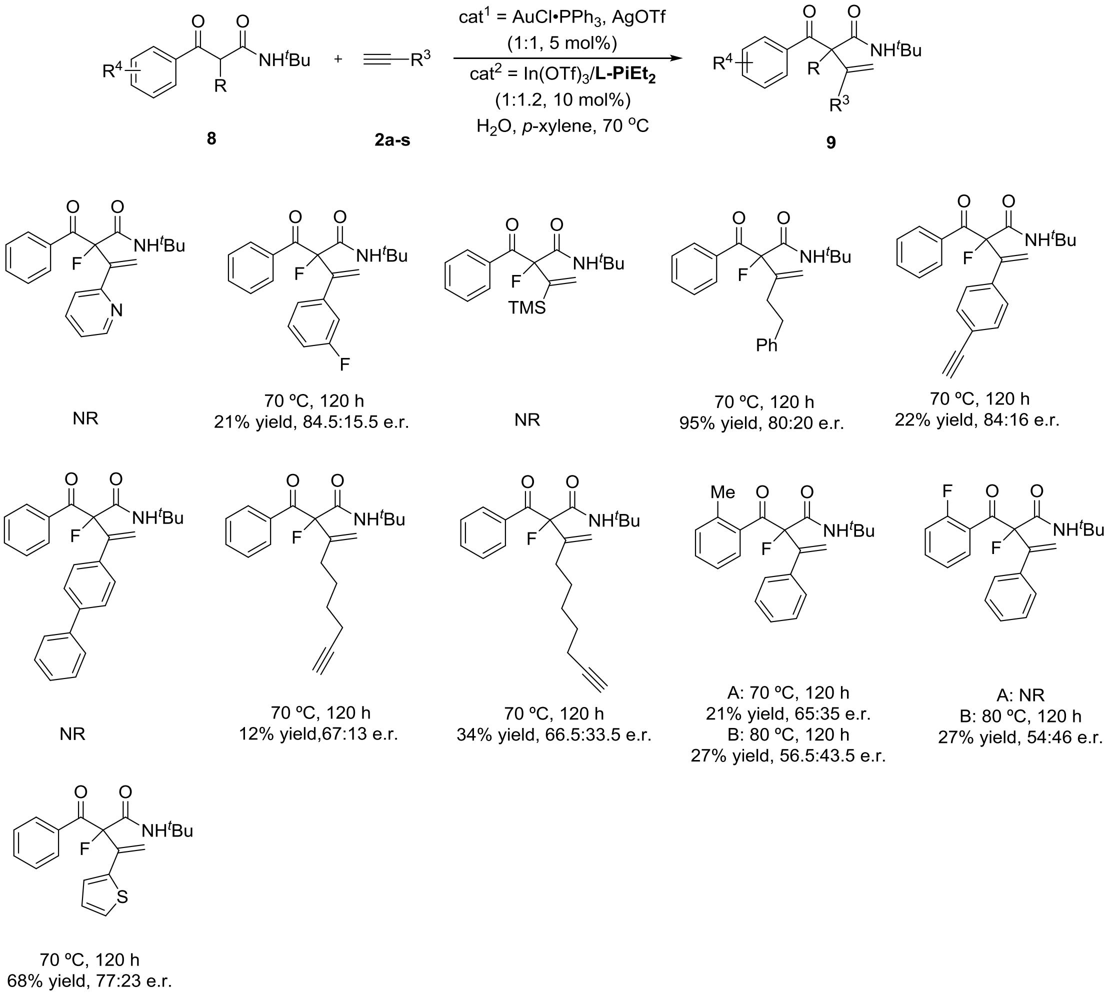

Supplementary Figure 3. Unsuccessful substrate scope of α-fluoro substituted acyclic β-ketoamides.

3. Supplementary Tables

Supplementary Table 1. Screen of metal salts for β-ketoamides[a]

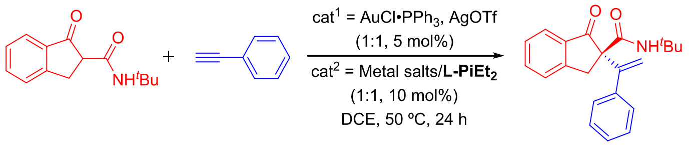

Entry	Metal salts	Yield (%)[b]	e.r.[c]
1	$Sc(OTf)_3$	92	60.5:39.5
2	$Ni(OTf)_2$	8	62.0:38.0
3	$Mg(OTf)_2$	trace	--
4	$Cu(OTf)_2$	14	race
5	$Zn(OTf)_2$	trace	--
6	$Yb(OTf)_3$	$<$10	50:50
7	$Fe(OTf)_3$	16	50:50
8	$Fe(OTf)_2$	32	50:50
9	$Ga(OTf)_3$	trace	--
10	$Gd(OTf)_3$	trace	--
11	$La(OTf)_3$	trace	--
12	$In(OTf)_3$	98	61.5:38.5
13[d]	$In(OTf)_3$	99	50:50
14	$Al(OTf)_3$	78	50:50

[a]Unless otherwise noted, all reactions were carried out with AuCl·PPh_3/AgOTf (1:1, 5 mol%), Metal salts/**L-PiEt$_2$** (1:1, 10 mol%), **1a** (0.10 mmol) and **2a** (2.0 equiv) in DCE (0.5 mL) at 50 ℃ for 24 h. [b]Yield of isolated product. [c]Determined by HPLC analysis on a chiral stationary phase. [d]Without **L-PiEt$_2$**.

Supplementary Table 2. Screening of the ligands for β-ketoamides[a]

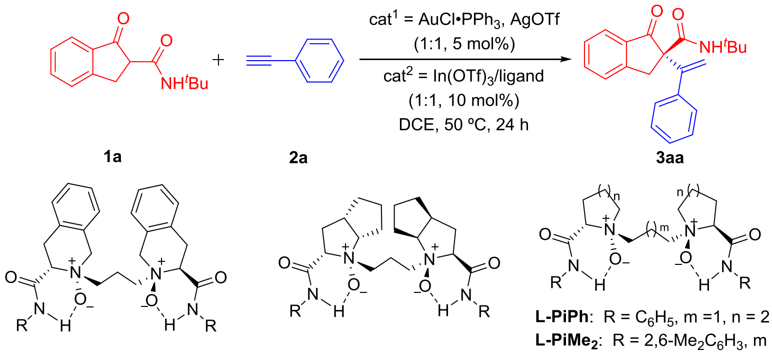

L-TQ-^{t}Bu: R = tert-butyl
L-TQ-Ad: R = 1-adamantyl
L-TQ-(*S*)-EPh: R = (s)-2-phenylethyl

L-RaEt$_2$: R = 2,6-Et$_2$C$_6$H$_3$

L-PiPh: R = C$_6$H$_5$, m =1, n = 2
L-PiMe$_2$: R = 2,6-Me$_2$C$_6$H$_3$, m = 1, n = 2
L$_2$-PiMe$_2$: R = 2,6-Me$_2$C$_6$H$_3$, m = 0, n = 2
L-PiEt$_2$: R = 2,6-Et$_2$C$_6$H$_3$, m = 1, n = 2
L-PiEt$_3$: R = 2,4,6-Et$_3$C$_6$H$_2$, m = 1, n = 2
L-PiMe$_3$: R = 2,4,6-Me$_3$C$_6$H$_2$, m = 1, n = 2
L$_4$-PiMe$_3$: R = 2,4,6-Me$_3$C$_6$H$_2$, m = 2, n = 2
L-PiEt$_2$Me: R = 2,6-Et$_2$-4-MeC$_6$H$_2$ m = 1, n = 2
L-PiEt$_2$Ad: R = 2,6-Et$_2$-AdC$_6$H$_2$, m = 1, n = 2
L-PiPr$_2$: R = 2,6-*i*-Pr$_2$C$_6$H$_2$, m = 1, n = 2
L-PiPr$_2$Br: R = 2,6-*i*-Pr$_2$-4-BrC$_6$H$_2$, m = 1, n = 2
L$_4$-PiPr$_2$: R = 2,6-*i*-Pr$_2$C$_6$H$_2$, m = 2, n = 2
L-PisEPh: R = (*S*)-2-phenylethyl, n = 2
L-PiCHPh$_2$: R = Ph$_2$CH, m = 1, n = 2
L-PicH: R = cyclohexyl, m = 1, n = 2
L-PiAd: R = 1-adamantyl, m = 1, n = 2
L-PrEt$_2$: R = 2,6-Et$_2$C$_6$H$_3$, m = 1, n = 1

Entry	Ligands	Yield (%)[b]	e.r.[c]
1	**L-TQ-tBu**	95	61.5:38.5
2	**L-TQ-Ad**	91	61.5:38.5
3	**L-TQ-(*s*)-EPh**	94	56.5:43.5
4	**L-RaEt$_2$**	88	55:45
5	**L-PiPh**	99	53.5:46.5
6	**L-PiMe$_2$**	99	61:39
7	**L$_2$-PiMe$_2$**	99	57:43
8	**L-PiEt$_2$**	98	61.5:38.5
9	**L-PiEt$_3$**	99	61.5:38.5
10	**L-PiMe$_3$**	99	61:39
11	**L$_4$-PiMe$_3$**	99	race
12	**L-PiEt$_2$Me**	99	63.5:36.5
13[d]	**L-PiEt$_2$Me**	99	65:35
14	**L-PiEt$_2$Ad**	99	58:42
15	**L-PiPr$_2$**	99	54.5:45.5
16	**L-PiPr$_2$Br**	99	52.5:47.5
17	**L$_4$-PiPr$_2$**	99	race
18	**L-Pi*s*EPh**	96	56:44
19	**L-Pi*c*H**	99	53.5:36.5

[a]Unless otherwise noted, all reactions were carried out, AuCl·PPh$_3$/AgOTf (1:1, 5 mol%), In(OTf)$_3$/ligand (1:1, 10 mol%), **1a** (0.10 mmol) and **2a** (2.0 equiv) in DCE (0.5 mL) at 50 ℃ for 24 h. [b]Yield of isolated product. [c]Determined by HPLC analysis on a chiral stationary phase. [d]2 μL H_2O as additive.

Supplementary Table 3. Investigating the ratio of the metal salts and ligand for

β-ketoamides[a]

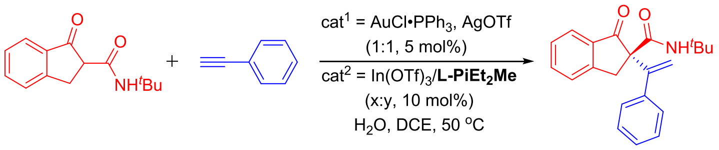

1a **2a** **3aa**

Entry	Ratio of M:L	Yield (%)[b]	e.r.[c]
1	1:1.5	trace	--
2	1:1.4	trace	--
3	1:1.3	99	68:32
4	1:1.2	98	70:30
5	1:1.1	99	65.5:34.5
6	1.1:1	99	63:37
7	1.2:1	98	60.5:39.5
8	1.3:1	99	59:41

[a]Unless otherwise noted, all reactions were carried out, AuCl·PPh$_3$/AgOTf (1:1, 5 mol%), In(OTf)$_3$/**L-PiEt$_2$Me** (x:y, 10 mol%), **1a** (0.10 mmol) and **2a** (2.0 equiv), 2 μL H_2O as additive in DCE (0.5 mL) at 50 ℃ for 24 h. [b]Yield of isolated product. [c]Determined by HPLC analysis on a chiral stationary phase.

Supplementary Table 4. Optimization of the solvents for β-ketoamides[a]

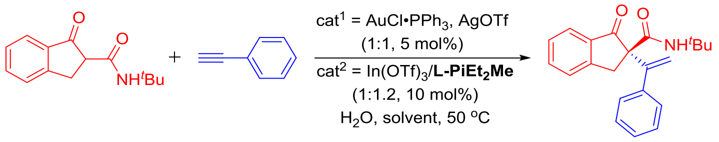

1a **2a** **3aa**

Entry	Solvent	Yield (%)[b]	e.r.[c]
1	toluene	90	86:14
2	*ortho*-xylene	94	86:14
3	*m*-xylene	91	89.5:10.5
4	*para*-xylene	98	90.5:9.5
5	mesitylene	94	90:10
6	bromobenzene	99	76.5:23.5
7	fluorobenzene	99	78:22
8	THF	15	55:45
9	$CHCl_3$	96	80:20
10	MeCN	13	57.5:42.5
11	EtOAc	67	64.5:35.5
12	MeOH	n.r.	--
13	DCE	93	68:32

[a]Unless otherwise noted, all reactions were carried out, AuCl·PPh$_3$/AgOTf (1:1, 5 mol%), In(OTf)$_3$/**L-PiEt$_2$Me** (1:1.2, 10 mol%), **1a** (0.10 mmol) and **2a** (2.0 equiv), 2 μL H_2O as additive in solvent (0.5 mL) at 50 ℃ for 24 h. [b]Yield of isolated product. [c]Determined by HPLC analysis on a

chiral stationary phase.

Supplementary Table 5. Screening of the concentration of reactiono for β-ketoamides[a]

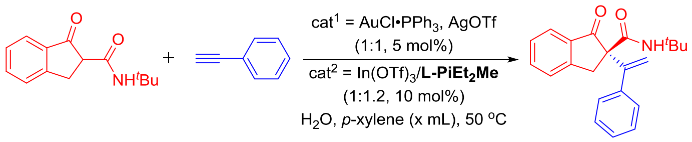

1a **2a** **3aa**

Entry	x (mL)	Yield (%)[b]	e.r.[c]
1	0.3	99	89:11
2	0.5	99	90.5:9.5
3	0.7	99	92:8
4	1.0	98	93:7
5	1.2	99	94:6
6	1.5	99	94.5:5.5
7[d]	1.5	64	90:10
8	1.7	95	94.5:5.5
9	2.0	95	94.5:5.5

[a]Unless otherwise noted, all reactions were carried out, AuCl·PPh$_3$/AgOTf (1:1, 5 mol%), In(OTf)$_3$/**L-PiEt$_2$Me** (1:1.2, 10 mol%), **1a** (0.10 mmol) and **2a** (2.0 equiv), 2 μL H_2O as additive in *p*-xylene (x mL) at 50 ℃ for 24 h. [b]Yield of isolated product. [c]Determined by HPLC analysis on a chiral stationary phase. [d]Sc(OTf)$_3$ as the metal salt.

Supplementary Table 6. Screening of the other catalytic systems for β-ketoamides

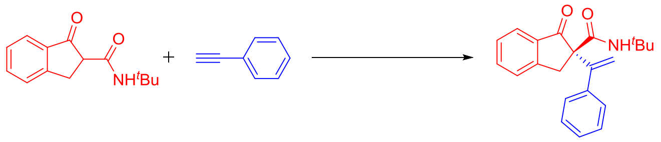

1a **2a** **3aa**

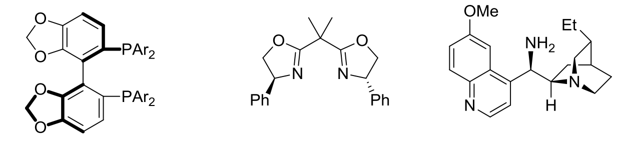

L1: (*R*)-DTBM-SEGPHOS
Ar = 3,5-tBu$_2$-4-MeC$_6$H$_2$

L2: (*S*,*S*)-tBu-Box

L4

Entry	Metal 1	Metal 2	Ligand	additive	Solvent	T (℃)	Yield (%)[b]	e.r.[c]
1[a]	Pd(OTf)$_2$	Yb(OTf)$_3$	**L1**	AcOH	Et$_2$O	r.t.	n.r.	--
2[a]	Pd(OTf)$_2$	Yb(OTf)$_3$	**L1**	AcOH	mesitylene	50	n.r.	--
3[b]	Zn(OAc)$_2$	Yb(OTf)$_3$	**L2**	HFIP, 4ÅMS	DCM	r.t.	n.r.	--
4[b]	Zn(OAc)$_2$	Yb(OTf)$_3$	**L2**	HFIP,	mesitylene	r.t.	n.r.	--

				4ÅMS				
5[b]	$Zn(OAc)_2$	$Yb(OTf)_3$	**L2**	HFIP, 4ÅMS	mesitylene	50	n.r.	--
6[b]	$Zn(OAc)_2$	$Yb(OTf)_3$	**L2**	HFIP, 4ÅMS	mesitylene	70	trace	50:50
7[c]	--	AgOTf	**L4**	TFA	MeOH	r.t.	n.r.	--
8[c]	--	AgOTf	**L4**	TFA	mesitylene	50	n.r.	--

[a]10 mol % **L1**, 10 mol% $Pd(OTf)_2$, 20 mol% $Yb(OTf)_3$, 10 equiv of AcOH, 0.1 mmol **1a** and 2.0 equiv **2a** in 1.0 mL corresponding solvent at r.t.–50 ℃ for 48 h. [b]10 mol% $Zn(OTf)_2$, 11 mol% **L2**, 20 mol% $Yb(OTf)_3$, 100 mol% HFIP, 50 mg 4ÅMS, 0.1 mmol **1a** and 2.0 equiv **2a** in 1.0 mL corresponding solvent at r.t.–70 ℃ for 72 h. [c]2.5 mol% AgOTf, 20 mol% **L3**, 20 mol% TFA, 0.2 mmol **1a** and 2.0 equiv **2a** in 1.0 mL corresponding solvent at r.t.–50 ℃ for 48 h.

Supplementary Table 7. Screening of the catalyst loading for β-ketoamides[a]

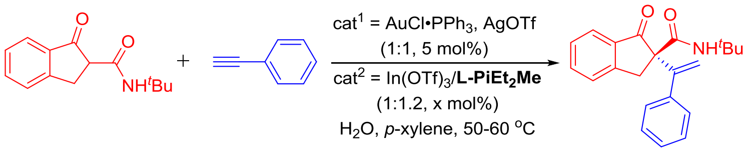

1a **2a** **3aa**

Entry	Catalysts Loading (x mol%)	Yield (%)[b]	e.r.[c]
1	15	98	94.5:5.5
2	10	98	94.5:5.5
3	5.0	53	87.5:12.5
4	2.5	36	72:28
5[d]	5.0	98	87:13
6[e]	5.0	70	88.5:11.5

[a]Unless otherwise noted, all reactions were carried out, $AuCl{\cdot}PPh_3$/AgOTf (1:1, 5 mol%), $In(OTf)_3$/**L-PiEt$_2$Me** (1:1.2, x mol%), **1a** (0.10 mmol) and **2a** (2.0 equiv), 2 μL H_2O as additive in *p*-xylene (1.5 mL) at 50 ℃ for 24 h. [b]Yield of isolated product. [c]Determined by HPLC analysis on a chiral stationary phase. [d]$AuCl{\cdot}PPh_3$/AgOTf (1:1, 5 mol%), $In(OTf)_3$/**L-PiEt$_2$Me** (1:1.2, 5 mol%), **1a** (0.10 mmol) and **2a** (2.0 equiv), 2 μL H_2O as additive in *p*-xylene (1.5 mL) at 60 ℃ for 48 h. [e]$AuCl{\cdot}PPh_3$/AgOTf (1:1, 2.5 mol%), $In(OTf)_3$/**L-PiEt$_2$Me** (1:1.2, 5 mol%), **1a** (0.10 mmol) and **2a** (2.0 equiv), 2 μL H_2O as additive in *p*-xylene (1.5 mL) at 60 °C for 48 h.

Supplementary Table 8. Screen of other Ligands for β-ketoamides[a]

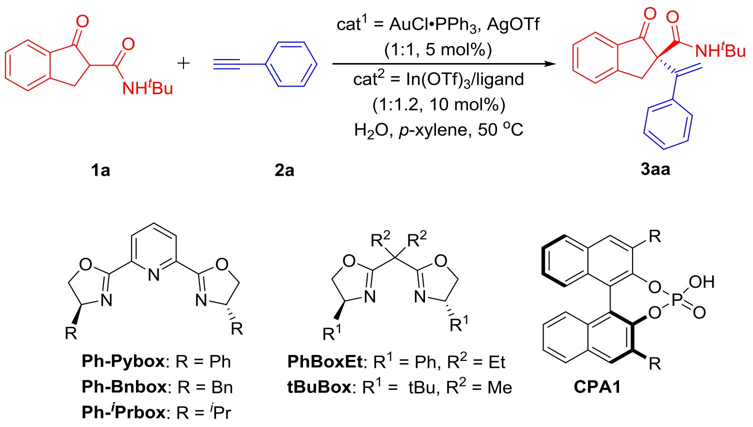

Entry	Ligand	Yield (%)[b]	e.r.[c]
1	**L-PiEt$_2$Me**	98	94.5:5.5
2	**Ph-Pybox**	13	47:53
3	**Ph-Bnbox**	13	49:51
4	**Ph-^{i}Prbox**	7	53.5:46.5
5	**PhBoxEt**	5	46.5:53.5
6	**tBuBox**	5	race
7[d]	**CPA1**	8	race

[a]Unless otherwise noted, all reactions were carried out, AuCl·PPh$_3$/AgOTf (1:1, 5 mol%), In(OTf)$_3$/ligand (1:1.2, 10 mol%), **1a** (0.10 mmol) and **2a** (2.0 equiv), 2 μL H_2O as additive in *p*-xylene (1.5 mL) at 50 ℃ for 24 h. [b]Yield of isolated product. [c]Determined by HPLC analysis on a chiral stationary phase. [d]Without In(OTf)$_3$.

Supplementary Table 9. Screen of the steric resistance of ligands on the gold for β-ketoamides[a]

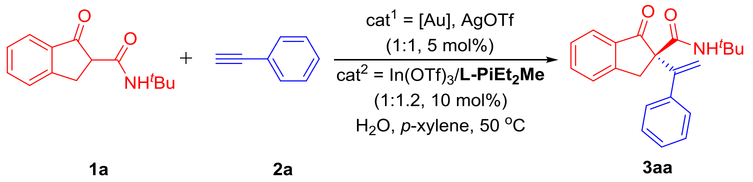

Entry	[Au]	Yield (%)[b]	e.r.[c]
1	AuCl·PPh$_2$Me	54	90.5:9.5
2	AuCl·PPhMe$_2$	38	89.5:10.5
3	AuCl·PPh$_2$OMe	73	83.5:6.5
4	XPhosAu(TA)OTf	trace	--
5	Chloro[tri(o-tolyl)phosphine]gold(I)	8	80:20
6	Chloro[tri(p-tolyl)phosphine]gold(I)	73	83.5:16.5
7	AuCl	n.r.	--
8	AuCl$_3$	n.r.	--

[a]Unless otherwise noted, all reactions were carried out, [Au]/AgOTf (1:1, 5 mol%), In(OTf)$_3$/**L-PiEt$_2$Me** (1:1.2, 10 mol%), **1a** (0.10 mmol) and **2a** (2.0 equiv), 2 μL H_2O as additive in *p*-xylene (1.5 mL) at 50 ℃ for 24 h. [b]Yield of isolated product. [c]Determined by HPLC analysis on a

chiral stationary phase.

Supplementary Table 10. Nonlinear effect of β-ketoamides[a]

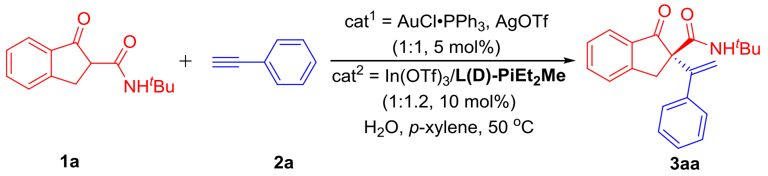

Entry	e.e.$_{Ligand}$ (%)	e.e.$_{Product}$ (%)[b]
1	0	0
2	20	17
3	40	37
4	60	68
5	80	80
6	100	89

[a]Unless otherwise noted, all reactions were carried out, AuCl·PPh$_3$/AgOTf (1:1, 5 mol%), In(OTf)$_3$/ligand (1:1.2, 10 mol%), **1a** (0.10 mmol) and **2a** (2.0 equiv), 2 μL H_2O as additive in *p*-xylene (1.5 mL) at 50 ℃ for 24 h. [b]Determined by HPLC analysis on a chiral stationary phase.

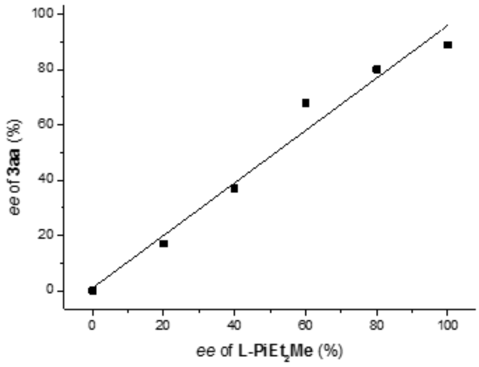

Supplementary Figure 4. Nonlinear effect.

Supplementary Table 11. Screen of metal salts for β-ketoester[a]

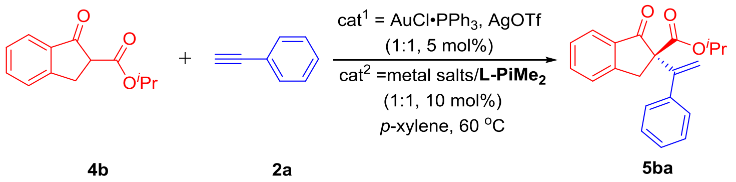

Entry	Metal salts	Yield (%)[b]	e.r.[c]
1	In(OTf)$_3$	73	61.5:38.5
2	Ni(OTf)$_2$	73	87.5:12.5
3	Sc(OTf)$_3$	82	52:48
4	Mg(OTf)$_2$	79	77:23
5	Zn(OTf)$_2$	trace	--
6	Cu(OTf)$_2$	45	47.5:52.5

7	$Al(OTf)_3$	trace	--
8	$Fe(OTf)_3$	63	65.5:34.5
9	$La(OTf)_3$	79	51:49

[a]Unless otherwise noted, all reactions were carried out, AuCl·PPh_3/AgOTf (1:1, 5 mol%), metal salts/**L-PiMe$_2$** (1:1, 10 mol%), **4b** (0.10 mmol) and **2a** (2.0 equiv) in *p*-xylene (1.0 mL) at 60 ℃ for 24 h. [b]Yield of isolated product. [c]Determined by HPLC analysis on a chiral stationary phase.

Supplementary Table 12. Screen of ligands for β-ketoester[a]

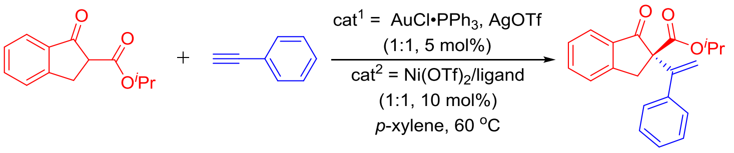

4b **2a** **5ba**

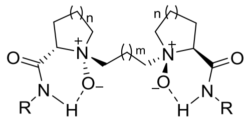

L-PiMe$_2$: R = 2,6-$Me_2C_6H_3$, m = 1, n = 2
L$_2$-PiMe$_2$: R = 2,6-$Me_2C_6H_3$, m = 0, n = 2
L-PiEt$_2$: R = 2,6-$Et_2C_6H_3$, m = 1, n = 2
L-PiMe$_3$: R = 2,4,6-$Me_3C_6H_2$, m = 1, n = 2
L-PiEt$_2$Me: R = 2,6-Et_2-4-MeC_6H_2 m = 1, n = 2
L-PiPr$_2$: R = 2,6-*i*-$Pr_2C_6H_2$, m = 1, n = 2
L-PiOEt$_2$: R = 2,6-$OEt_2C_6H_3$, m = 1, n = 2
L-Pi-(*S*)-EPh: R = (s)-2-phenylethyl

Entry	Ligand	Yield (%)[b]	e.r.[c]
1	**L-PiMe$_2$**	73	61.5:38.5
2	**L-PiMe$_3$**	72	93.5:6.5
3	**L-PiEt$_2$**	83	91:9
4	**L-PiEt$_2$Me**	65	89:11
5	**L-PiPr$_2$**	18	82.5:17.5
6	**L$_2$-PiMe$_2$**	trace	--
7	**L-PiOEt$_2$**	trace	--
8	**L-Pi-(*S*)-EPh**	35	77.5:22.5

[a]Unless otherwise noted, all reactions were carried out, AuCl·PPh_3/AgOTf (1:1, 5 mol%), metal salts/ligand (1:1, 10 mol%), **4b** (0.10 mmol) and **2a** (2.0 equiv) in *p*-xylene (1.0 mL) at 60 ℃ for 24 h. [b]Yield of isolated product. [c]Determined by HPLC analysis on a chiral stationary phase.

Supplementary Table 13. Screen of metal salts for 1,3-diketones[a]

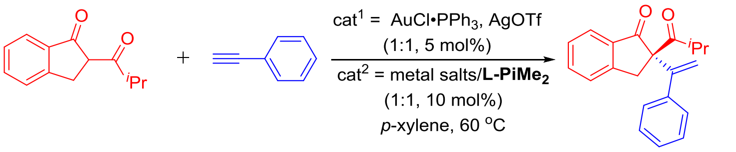

6a **2a** **7aa**

Entry	Metal salts	Yield (%)[b]	e.r.[c]
1	$Sc(OTf)_3$	ND	52.5:47.5
2	$Ni(OTf)_2$	ND	91.5:8.5

3	$Mg(OTf)_2$	trace	--
4	$Cu(OTf)_2$	trace	--
5	$Zn(OTf)_2$	trace	--
6	$Al(OTf)_3$	trace	--
7	$Fe(OTf)_3$	trace	--
8	$La(OTf)_3$	ND	race
9	$In(OTf)_3$	ND	race

[a]Unless otherwise noted, all reactions were carried out, $AuCl{\cdot}PPh_3/AgOTf$ (1:1, 5 mol%), Metal salts/**L-PiMe$_2$** (1:1, 10 mol%), **6a** (0.10 mmol) and **2a** (2.0 equiv) in *p*-xylene (1.0 mL) at 60 ℃ for 24 h. [b]Yield of isolated product. [c]Determined by HPLC analysis on a chiral stationary phase.

Supplementary Table 14. Screen of ligands for 1,3-diketones[a]

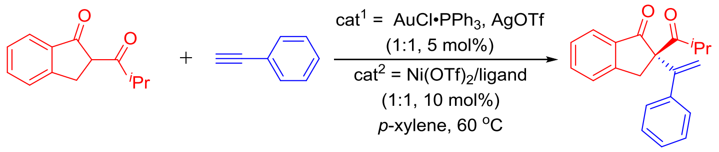

6a **2a** **7aa**

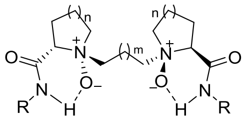

L-PiMe$_2$: R = 2,6-$Me_2C_6H_3$, m = 1, n = 2
L$_2$-PiMe$_2$: R = 2,6-$Me_2C_6H_3$, m = 0, n = 2
L-PiEt$_2$: R = 2,6-$Et_2C_6H_3$, m = 1, n = 2
L-PiEt$_3$: R = 2,4,6-$Et_3C_6H_2$, m = 1, n = 2
L-PiMe$_3$: R = 2,4,6-$Me_3C_6H_2$, m = 1, n = 2
L-PiEt$_2$Me: R = 2,6-Et_2-4-MeC_6H_2 m = 1, n = 2
L-PiPr$_2$: R = 2,6-*i*-$Pr_2C_6H_2$, m = 1, n = 2
L-PrEt$_2$: R = 2,6-$Et_2C_6H_3$, m = 1, n = 1
L-PiOMe$_2$: R = 2,6-$OMe_2C_6H_3$, m = 1, n = 2

Entry	Ligands	Yield (%)[b]	e.r.[c]
1	**L-PrEt$_2$**	86	race
2	**L-PiEt$_2$**	99	87.5:12.5
3	**L-PiMe$_3$**	65	91.5:8.5
4	**L-PiMe$_2$**	ND	91.5:8.5
5	**L-PiEt$_3$**	69	91:9
6	**L-PiEt$_2$Me**	96	95:5
7	**L-PiPr$_2$**	60	92.5:7.5
8	**L-PiOMe$_2$**	63	54:46
9	**L$_2$-PiMe$_2$**	trace	--

[a]Unless otherwise noted, all reactions were carried out, $AuCl{\cdot}PPh_3/AgOTf$ (1:1, 5 mol%), $Ni(OTf)_2$/ligand (1:1, 10 mol%), **6a** (0.10 mmol) and **2a** (2.0 equiv) in *p*-xylene (1.0 mL) at 60 ℃ for 24 h. [b]Yield of isolated product. [c]Determined by HPLC analysis on a chiral stationary phase.

4. Experimental procedure for the scale-up reaction and

transformation of the product

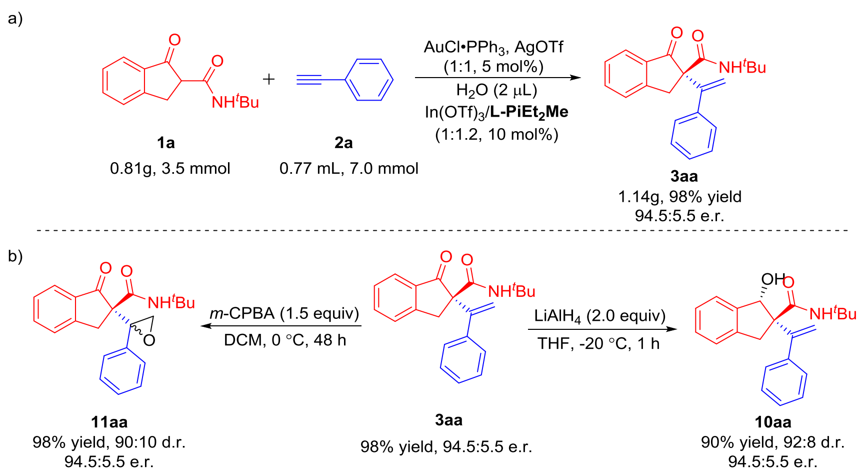

Supplementary Figure 5. Experimental procedure for the scale-up reaction and transformation of the product.

a) A mixture of AuCl·PPh$_3$ (5 mol%, 87.5 mg), AgOTf (5 mol%, 45.5 mg), In(OTf)$_3$ (10 mol%, 196.0 mg), **L-PiEt$_2$Me** (12 mol%, 259.0 mg) and the substrate **1a** (3.5 mmol) were added to a test tube under N_2 atmosphere. Then, anhydrous *p*-xylene (52.5 mL) was added and the mixture was stirred at 30 ℃ for 3.0 h. Subsequently, H_2O (1.1 equiv, 70.0 μL) was added under stirring at 30 °C. Ten minutes later, **2a** (7.0 mmol, 770.0 μL) was added at 50 °C, and the reaction mixture continued stirring at 50 °C for 24 h. The residue was purified by flash chromatography on silica gel (petroleum ether/ethyl acetate = 15:1, v/v) to afford the desired product **3aa** (1.14 g, 98% yield, 94.5:5.5 e.r.).

b) A dry reaction tube was charged with the $LiAlH_4$ (0.20 mmol, 7.6 mg) under N_2 atomosphere. Then, 0.5 mL anhydrous THF added and stirred at -20 °C for 10 minutes, product **3aa** (0.10 mmol, 33.3 mg, 94.5:5.5 e.r.) dissolved in 0.5 mL anhydrous THF and added into $LiAlH_4$, the reaction mixture continued stirring at -20 °C for 1.0 h. Quenched the reaction mixture with 1.0 mL H_2O and extracted product using dichloromethane. Dried the organic layers over Na_2SO_4 and concentrated in vacuo to yield the crude product. Then the residue was purified by flash chromatography on silica gel (Eluent: petroleum ether/ethyl acetate/ = 4:1, v/v) to afford the product **10aa** (30.2 mg, 90% yield, 92:8 d.r., 94.5:5.5 e.r.). The absolute configuration of the major isomer was confirmed to be (1*S*, 2*R*) by X-ray crystal

analysis[7], and the stereo-arrangement at the quaternary carbon center is in consist with that of **3ae**.

c) A dry reaction tube was charged with the *m*-CPBA (0.2 mmol, 34.5 mg), Then anhydrous dichloromethane (1.0 mL) was added and the mixture was stirred at 0 °C for 10 minutes. Subsequently, product **3aa** (0.10 mmol, 33.3 mg, 94.5:5.5 e.r.) was added and reacted at 0 °C for 48 h. The residue was purified by flash chromatography on silica gel (petroleum ether/ethyl acetate = 6:1, v/v) to afford the desired product **11aa** (34.2 mg, 98% yield, 90:10 d.r., 94.5:5.5 e.r.).

5. Control experiments

Supplementary Table 15. Control experiments[a]

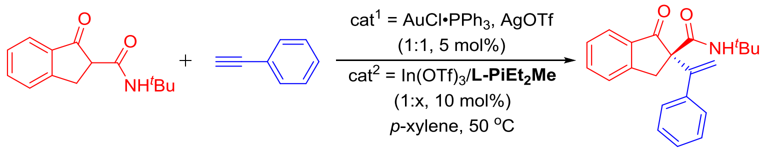

Entry	x	H_2O (2 μL)	Yield (%)[b]	e.r.[c]
1	0	--	48	--
2	0	√	34	--
3	1.0	--	52	86:14
4	1.0	√	41	88.5:11.5
5	1.1	--	54	87:13
6	1.1	√	42	89:11
7	1.2	--	53	89.5:10.5
8	1.2	√	43	91:9

[a]Unless otherwise noted, all reactions were carried out, AuCl·PPh_3/AgOTf (1:1, 5 mol%), $In(OTf)_3$/**L-PiEt$_2$Me** (1:x, 10 mol%), **1a** (0.10 mmol) and **2a** (2.0 equiv) in *p*-xylene (1.5 mL) at 50 ℃ for 6 h. [b]Yield of isolated product. [c]Determined by HPLC analysis on a chiral stationary phase.

6. HRMS analysis and X-ray crystallography data

6.1 HRMS analysis

a) The mixture of **L-PiEt$_2$Me**, $In(OTf)_3$, **1a** in para-xylene

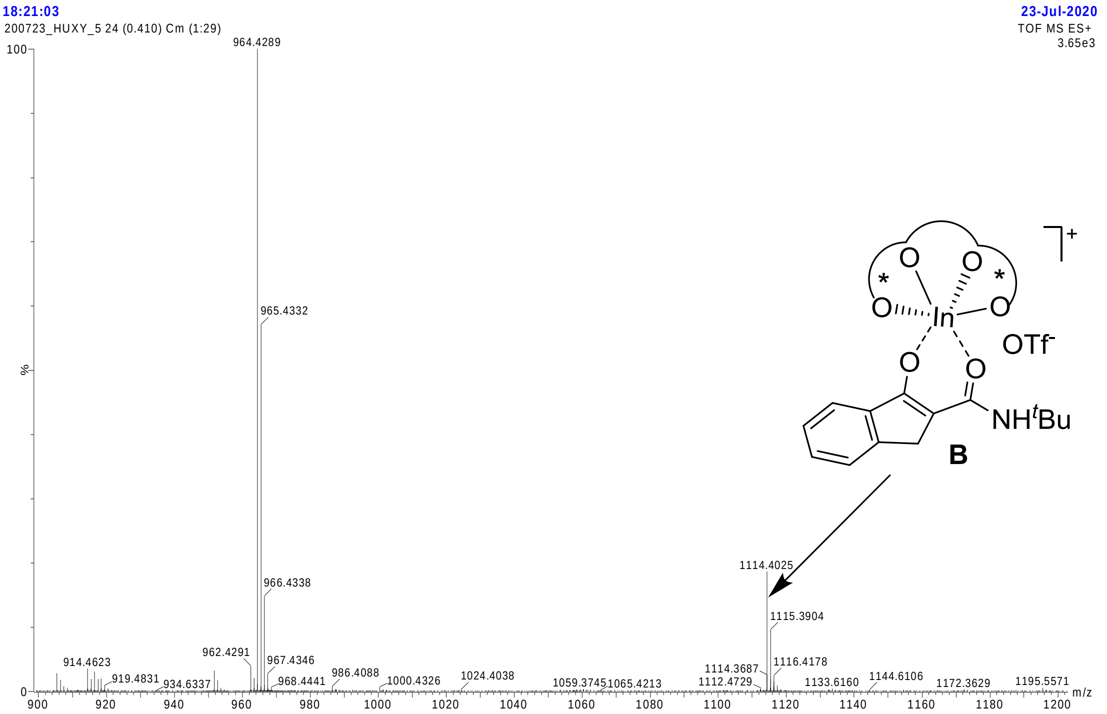

b) The mixture of AuCl ·PPh$_3$/AgOTf, **2a** in para-xylene

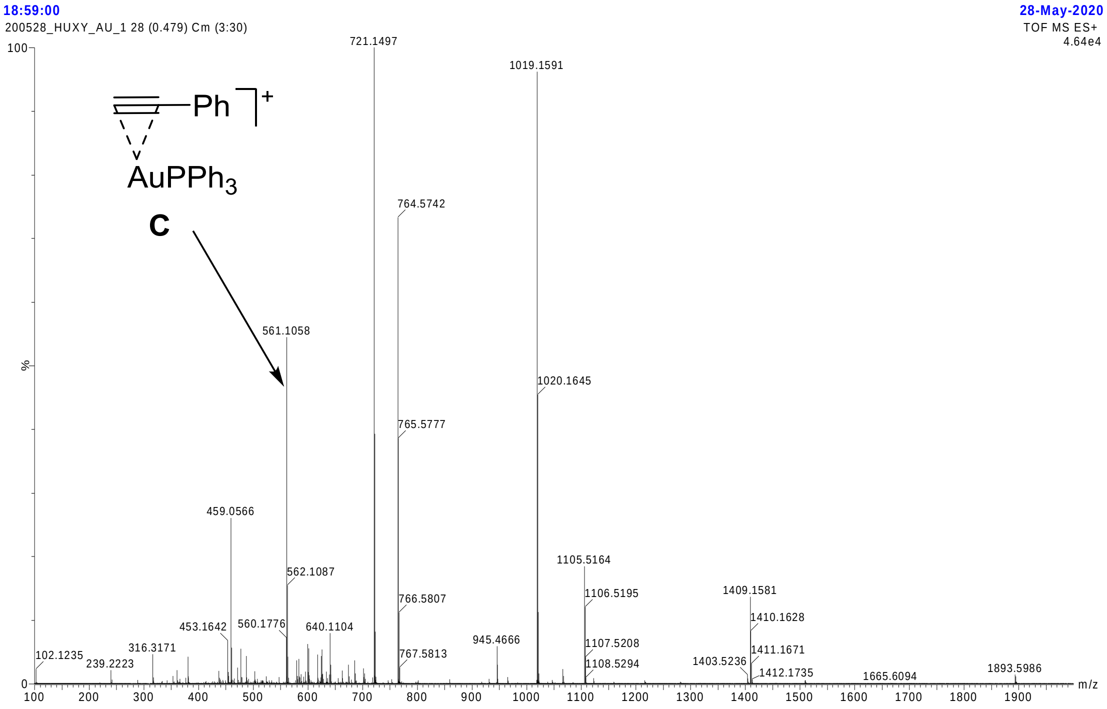

6.2 X-ray crystallography data

a). The absolute configuration of 3ae was determined by X-ray chromatography analysis.

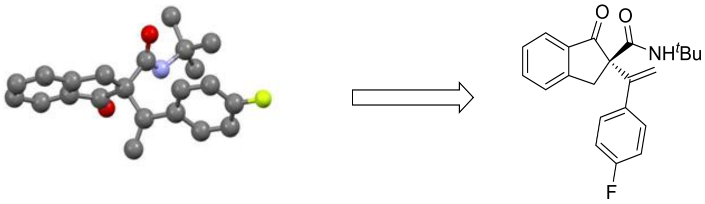

Single crystal of the product **3ae** [$C_{22}H_{22}FNO_2$] was obtained from the mixed solvents of petroleum ether, and dichloromethane (10:1, v/v). The absolute configuration of **3ae** is (*R*). CCDC 1964558 contains the supplementary crystallographic data which can be obtained free of charge from the Cambrige Crystallographic Data Centere via www.ccdc.cam.ac.uk/data_request/cif.

Crystallographic Data for $C_{22}H_{22}FNO_2$.

Formula	$C_{22}H_{22}FNO_2$	*For the 2nd crystal if you need*
Formula mass (amu)	351.40	
Space group	P21 21 21	
a (Å)	a=9.092(3) b=9.540(3) c=21.671(7)<br>alpha=90 beta=90 gamma=90	
c (Å)	a=9.092(3) b=9.540(3) c=21.671(7)<br>alpha=90 beta=90 gamma=90	
c (Å)	a=9.092(3) b=9.540(3) c=21.671(7)<br>alpha=90 beta=90 gamma=90	
α (deg)	a=9.092(3) b=9.540(3) c=21.671(7)<br>alpha=90 beta=90 gamma=90	
β (deg)	a=9.092(3) b=9.540(3) c=21.671(7)<br>alpha=90 beta=90 gamma=90	
γ (deg)	a=9.092(3) b=9.540(3) c=21.671(7)<br>alpha=90 beta=90 gamma=90	
V (Å^3)	1879.8(11)	
Z	4	
λ (Å)	1.54178	
T (K)	185 K	

ρ_{calcd} (g cm^{-3})	1.242
μ (mm^{-1})	0.696
Transmission factors	0.451–0.753
$2\theta_{max}$ (deg)	64.903
No. of unique data, including $F_o^2 < 0$	3127
No. of unique data, with $F_o^2 > 2\sigma(F_o^2)$	3117
No. of variables	242
$R(F)$ for $F_o^2 > 2\sigma(F_o^2)$ [a]	0.0347
$R_w(F_o^2)$ [b]	0.0963
Goodness of fit	1.103

[a] $R(F) = \sum||F_o| - |F_c|| / \sum|F_o|$.

[b] $R_w(F_o^2) = [\sum[w(F_o^2 - F_c^2)^2] / \sum wF_o^4]^{1/2}$; $w^{-1} = [\sigma^2(F_o^2) + (Ap)^2 + Bp]$, where $p = [\max(F_o^2,0) + 2F_c^2] / 3$.

(b) The absolute configuration of 10aa was determined by X-ray chromatography analysis.

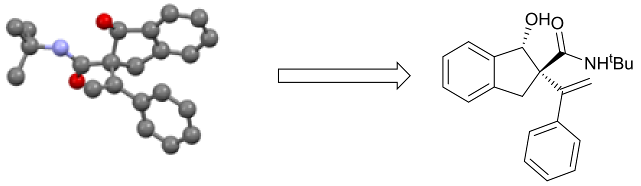

The chiral compound **10aa** was obtained from the reduction of chiral substrate **3aa** in the presence of $LiAlH_4$. Single crystal of the chiral compound **10aa** [$C_{22}H_{25}NO_2$] was obtained from the mixed solvents of petroleum ether, and dichloromethane (8:1, v/v). The absolute configuration of **10aa** is (1*S*, 2*R*). CCDC 1989114 contains the supplementary crystallographic data which can be obtained free of charge from the Cambrige Crystallographic Data Centere via www.ccdc.cam.ac.uk/data_request/cif.

Crystallographic Data for $C_{22}H_{25}NO_2$.

Formula	$C_{22}H_{25}NO_2$	*For the 2nd crystal if you need*
Formula mass (amu)	335.43	
Space group	P 21 21 21	

a (Å)	a = 9.3940 (7)	b = 11.6177 (8)	c = 17.4458 (16)
	alpha = 90	beta = 90	gamma = 90
c (Å)	a = 9.3940 (7)	b = 11.6177 (8)	c = 17.4458 (16)
	alpha = 90	beta = 90	gamma = 90
c (Å)	a = 9.3940 (7)	b = 11.6177 (8)	c = 17.4458 (16)
	alpha = 90	beta = 90	gamma = 90
α (deg)	a = 9.3940 (7)	b = 11.6177 (8)	c = 17.4458 (16)
	alpha = 90	beta = 90	gamma = 90
β (deg)	a = 9.3940 (7)	b = 11.6177 (8)	c = 17.4458 (16)
	alpha = 90	beta = 90	gamma = 90
γ (deg)	a = 9.3940 (7)	b = 11.6177 (8)	c = 17.4458 (16)
	alpha = 90	beta = 90	gamma = 90
V (Å^3)	1904.0 (3)		
Z	4		
λ (Å)	1.54178		
T (K)	195 K		
ρ_{calcd} (g cm^{-3})	1.170		
μ (mm^{-1})	0.583		
Transmission factors	0.828–0.998		
$2\theta_{max}$ (deg)	68.274		
No. of unique data, including $F_o^2 < 0$	3056		
No. of unique data, with $F_o^2 > 2\sigma(F_o^2)$	2770		
No. of variables	237		
$R(F)$ for $F_o^2 > 2\sigma(F_o^2)$ [a]	0.0467		
$R_w(F_o^2)$ [b]	0.1760		
Goodness of fit	1.180		

[a] $R(F) = \sum||F_o| - |F_c|| / \sum|F_o|$.

[b] $R_w(F_o^2) = [\sum[w(F_o^2 - F_c^2)^2] / \sum wF_o^4]^{1/2}$; $w^{-1} = [\sigma^2(F_o^2) + (Ap)^2 + Bp]$, where $p = [\max(F_o^2,0) + 2F_c^2] / 3$

Supplementary Table 16. Screening of the ligands for acyclic substrate[a]

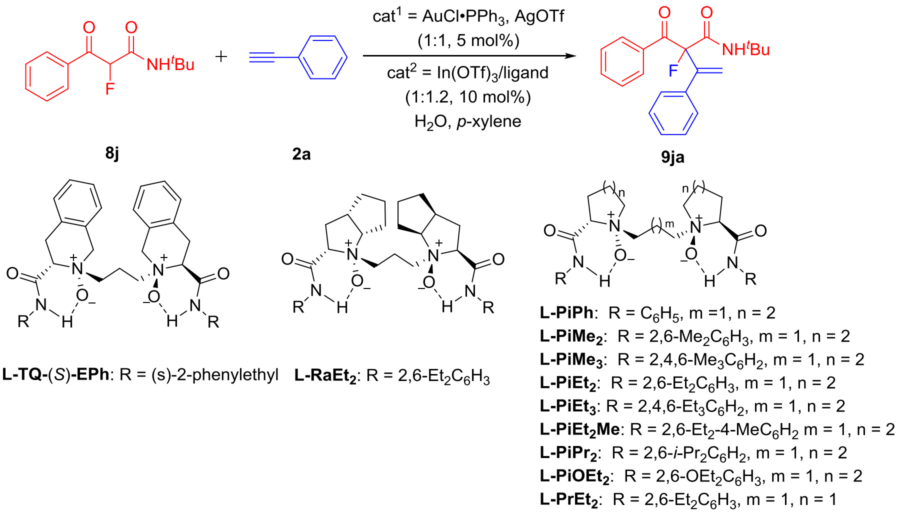

Entry	Ligands	Tem (℃)	Time (h)	Yield (%)[b]	e.r.[c]
1	**L-PiPh**	80	120	trace	--
2	**L-PiMe$_2$**	80	120	30	58:42
3	**L-PiMe$_3$**	80	120	36	77:23
5	**L-PiEt$_2$Me**	80	120	38	86:14
6	**L-PiEt$_3$**	80	120	10	58.5:41.5
7	**L-PiPr$_2$**	80	120	24	62:38
8	**L-PiOEt$_2$**	80	120	34	72.5:27.5
9	**L-PiEt$_2$**	80	120	40	88.5:11.5
10	**L-PiEt$_2$**	70	120	46	91.5:8.5
11	**L-PiEt$_2$**	60	120	31	93:7
12	**L-PiEt$_2$**	50	120	trace	--
13	**L-PiEt$_2$**	70	72	33	90.5:9.5
14	**L-PrEt$_2$**	70	72	15	68:32
15	**L-RaEt$_2$**	70	72	trace	--
16	**L-TQCy**	70	72	31	80:20

[a]Unless otherwise noted, all reactions were carried out, AuCl·PPh$_3$/AgOTf (1:1, 5 mol%), In(OTf)$_3$/ligand (1:1.2, 10 mol%), **8j** (0.10 mmol) and **2a** (3.0 equiv) H_2O (2 μL) as additive in *p*-xylene (1.5 mL) at 50–80 ℃ for 72–120 h. [b]Yield of isolated product. [c]Determined by HPLC analysis on a chiral stationary phase.

7. The analytical and spectral characterization data of products

(*R*)-*N*-(*tert*-Butyl)-1-oxo-2-(1-phenylvinyl)-2,3-dihydro-1*H*-indene-2-carboxamide

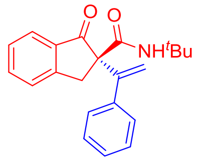

3aa

colorless oil; 98% yield, 94.5:5.5 e.r.; R_f = 0.50 (petroleum ether/ethyl acetate = 10/1); $[\alpha]^{24}_{D}$ = –356.0 (c = 0.49, in DCM).
HPLC DAICEL CHIRALCEL IA, hexane/*i*-PrOH = 95/05, flow rate 1.0 mL/min, λ = 254 nm, t_r (major) = 7.24 min, t_r (minor) = 8.28 min.
IR (neat): 2966, 1700, 1678, 1607, 1519, 1458, 1266, 1218, 1027, 910, 772, 702 cm^{-1}.
^{1}H NMR (400 MHz, $CDCl_3$) δ 7.77 (d, J = 7.6 Hz, 1H), 7.62 – 7.58 (m, 1H), 7.42 – 7.28 (m, 7H), 6.56 (s, 1H), 5.48 (d, J = 8.4 Hz, 2H), 4.41 (d, J = 17.2 Hz, 1H), 3.11 (d, J = 17.6 Hz, 1H), 1.22 (s, 9H).
^{13}C{^{1}H} NMR δ 203.6, 166.5, 153.9, 149.1, 140.2, 135.8, 134.9, 128.4, 128.0, 127.6, 127.3, 126.5, 124.7, 116.9, 67.3, 51.5, 37.8, 28.2.
HRMS (ESI-TOF) calcd for $C_{22}H_{23}NNaO_2^+$ ($[M+Na]^+$) = 356.1621, found 356.1627.
Chiral HPLC spectrum **3aa**:

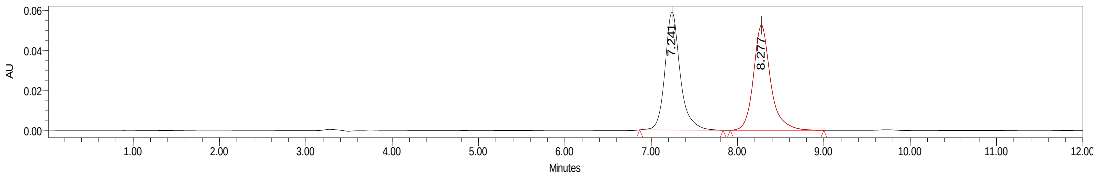

Peak	Retention Time	Area	% Area
1	7.241	721548	50.02
2	8.277	720918	49.98

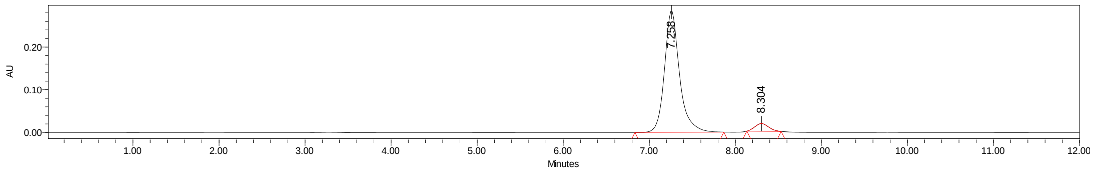

Peak	Retention Time	Area	% Area
1	7.258	3446794	94.53
2	8.304	199308	5.47

(*R*)-*N*-(*tert*-Butyl)-5-methyl-1-oxo-2-(1-phenylvinyl)-2,3-dihydro-1*H*-indene-2-carboxamide

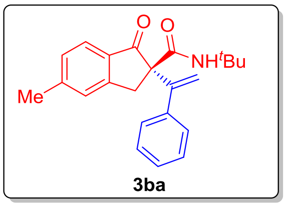

colorless oil; 96% yield, 94.5:5.5 e.r.; R_f = 0.50 (petroleum ether/ethyl acetate = 10/1); $[\alpha]^{23}_{D}$ = –335.2 (c = 0.55, in DCM).
HPLC DAICEL CHIRALCEL IA, hexane/*i*-PrOH = 98/02, flow rate 1.0 mL/min, λ = 254 nm) t_r (major) = 6.54 min, t_r (minor) = 7.60 min.
IR (neat): 2966, 1696, 1607, 1519, 1452, 1218, 1117, 1028, 906, 702 cm^{-1}.
^{1}H NMR (400 MHz, $CDCl_3$) δ 7.66 (d, J = 7.6 Hz, 1H), 7.34 – 7.27 (m, 5H), 7.21 – 7.17 (m, 2H), 6.56 (s, 1H), 5.46 (d, J = 9.2 Hz, 2H), 4.36 (d, J = 17.6 Hz, 1H), 3.06 (d, J = 17.2 Hz, 1H), 2.42 (s, 3H), 1.21 (s, 9H).
^{13}C{^{1}H} NMR (101 MHz, $CDCl_3$) δ 203.0, 166.6, 154.3, 149.3, 147.3, 140.3, 132.7, 128.9, 128.3, 127.9, 127.3, 126.8, 124.5, 116.8, 67.4, 51.4, 37.6, 28.2, 22.2.
HRMS (ESI-TOF) calcd for $C_{23}H_{26}NO_2^+$ ($[M+H]^+$) = 348.1958, found 348.1953.
Chiral HPLC spectrum **3ba**:

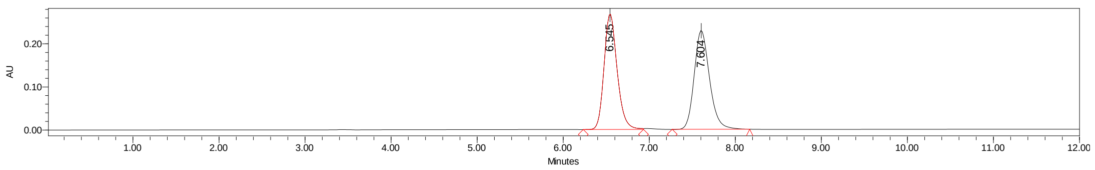

Peak	Retention Time	Area	% Area
1	6.545	2824316	49.93
2	7.604	2832230	50.07

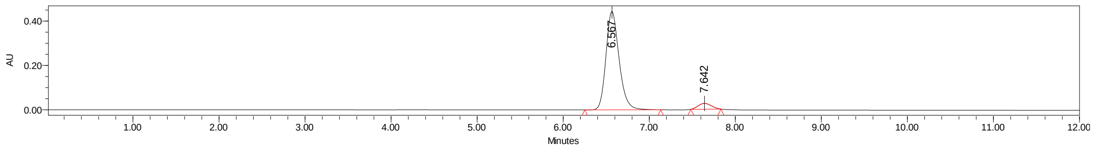

Peak	Retention Time	Area	% Area
1	6.567	4705215	94.40
2	7.642	279262	5.60

(*R*)-*N*-(*tert*-Butyl)-6-methyl-1-oxo-2-(1-phenylvinyl)-2,3-dihydro-1*H*-indene-2-carboxamide

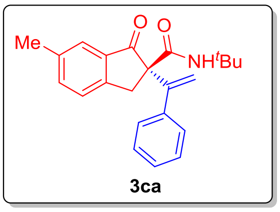

colorless oil; 99% yield, 95:5 e.r.; R_f = 0.50 (petroleum ether/ethyl acetate = 10/1); $[\alpha]^{22}_D$ = –354.2 (*c* = 0.69, in DCM).
HPLC DAICEL CHIRALCEL IA, hexane/*i*-PrOH = 90/10, flow rate 1.0 mL/min, λ = 254 nm) t_r (major) = 4.77 min, t_r (minor) = 6.15 min.
IR (neat): 2924, 1698, 1521, 1494, 1272, 1222, 1154, 775, 701, 502 cm^{-1}.
^{1}H NMR (400 MHz, $CDCl_3$) δ 7.57 – 7.56 (m, 1H), 7.42 (dd, *J* = 8.0, 1.6 Hz, 1H), 7.34 – 7.28 (m, 6H), 6.55 (s, 1H), 5.46 (d, *J* = 6.0 Hz, 2H), 4.35 (d, *J* = 17.2 Hz, 1H), 3.06 (d, *J* = 17.2 Hz, 1H), 2.39 (s, 3H), 1.21 (s, 9H).
^{13}C{^{1}H} NMR (101 MHz, $CDCl_3$) δ 203.7, 166.6, 151.3, 149.2, 140.2, 137.5, 137.2, 135.1, 128.3, 127.9, 127.3, 126.1, 124.5, 116.8, 67.6, 51.5, 37.4, 28.2, 21.1.
HRMS (ESI-TOF) calcd for $C_{23}H_{26}NO_2^+$ ([M]+H^+) = 348.1958, found 348.1951.
Chiral HPLC spectrum **3ca**:

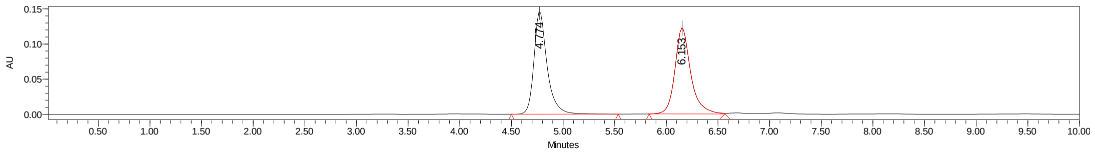

Peak	Retention Time	Area	% Area
1	4.774	1294360	49.78
2	6.153	1305876	50.22

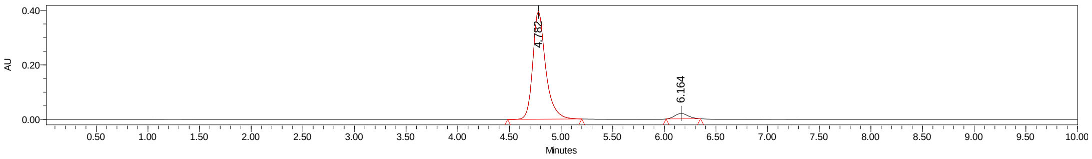

Peak	Retention Time	Area	% Area
1	4.782	3414195	95.05
2	6.164	177683	4.95

(*R*)-*N*-(*tert*-Butyl)-6-methoxy-1-oxo-2-(1-phenylvinyl)-2,3-dihydro-1*H*-indene-2-carboxamide

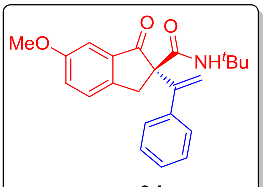

3da

colorless oil; 84% yield, 95:5 e.r.; R_f = 0.35 (petroleum ether/ethyl acetate = 10/1); $[\alpha]^{23}_D$ = –356.6 (*c* = 0.42, in DCM).
HPLC DAICEL CHIRALCEL IA, hexane/*i*-PrOH = 90/10, flow rate 1.0 mL/min, λ = 254 nm) t_r (major) = 6.77 min, t_r (minor) = 10.64 min.
IR (neat): 2965, 1698, 1521, 1493, 1455, 1272, 1226, 1164, 1029, 774, 701 cm^{-1}.
^{1}**H NMR** (400 MHz, $CDCl_3$) δ 7.34 – 7.28 (m, 6H), 7.22 – 7.16 (m, 2H), 6.54 (s, 1H), 5.47 (d, *J* = 2.8 Hz, 2H), 4.32 (d, *J* = 17.2 Hz, 1H), 3.84 (s, 3H), 3.03 (d, *J* = 17.2 Hz, 1H), 1.22 (s, 9H).
^{13}**C{^{1}H} NMR** (101 MHz, $CDCl_3$) δ 203.6, 166.5, 159.6, 149.2, 147.0, 140.2, 136.0, 128.3, 127.9, 127.3, 127.2, 125.4, 116.8, 105.5, 68.2, 55.6, 51.5, 37.2, 28.2.
HRMS (ESI-TOF) calcd for $C_{23}H_{25}NNaO_3^+$ ([M]+Na^+) = 386.1727, found 386.1719.
Chiral HPLC spectrum **3da**:

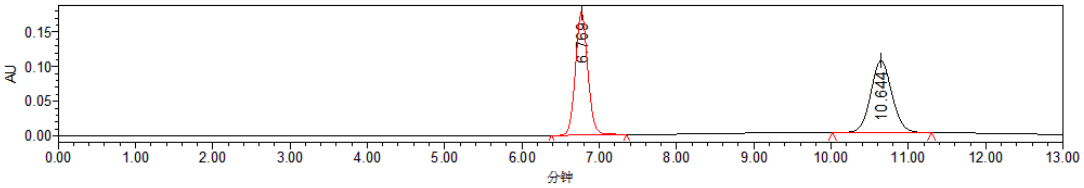

Peak	Retention Time	Area	% Area
1	6.769	1972139	50.03
2	10.644	1969804	49.97

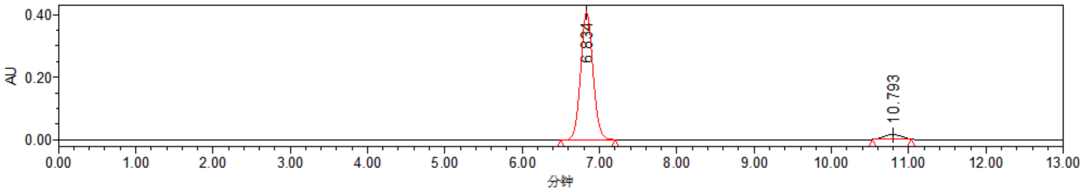

Peak	Retention Time	Area	% Area
1	6.834	4527731	95.08
2	10.793	234539	4.92

N-(*tert*-Butyl)-5,6-dimethoxy-1-oxo-2-(1-phenylvinyl)-2,3-dihydro-1*H*-indene-2-carboxamide

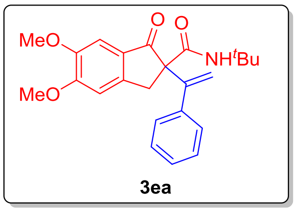

colorless oil; 97% yield, 95.5:4.5 e.r.; R_f = 0.34 (petroleum ether/ethyl acetate = 4/1); $[\alpha]^{23}_D$ = −144.9 (*c* = 0.55, in DCM).
HPLC DAICEL CHIRALCEL IA, hexane/*i*-PrOH = 90/10, flow rate 1.0 mL/min, λ = 254 nm) t_r (major) = 7.61 min, t_r (minor) = 11.69 min.
IR (neat): 2925, 1684, 1591, 1499, 1458, 1311, 1221, 1125, 1031, 778, 701 cm^{-1}.
^{1}H NMR (400 MHz, $CDCl_3$) δ 7.34 – 7.27 (m, 5H), 7.17 (s, 1H), 6.83 (s, 1H), 6.59 (s, 1H), 5.47 (d, *J* = 14.4 Hz, 2H), 4.32 (dd, *J* = 17.2, 0.8 Hz, 1H), 3.93 (d, *J* = 10.0 Hz, 6H), 3.02 (dd, *J* = 17.2, 0.8 Hz, 1H), 1.21 (s, 9H).
^{13}C{^{1}H} NMR (101 MHz, $CDCl_3$) δ 202.0, 166.8, 156.6, 149.7, 149.7, 149.4, 140.4, 128.3, 127.8, 127.6, 127.3, 116.7, 107.2, 104.8, 67.6, 56.3, 56.1, 51.4, 37.6, 28.2.
HRMS (ESI-TOF) calcd for $C_{24}H_{27}NNaO_4^+$ ([M]+Na$^+$) = 416.1832, found 416.1827.
Chiral HPLC spectrum **3ea**:

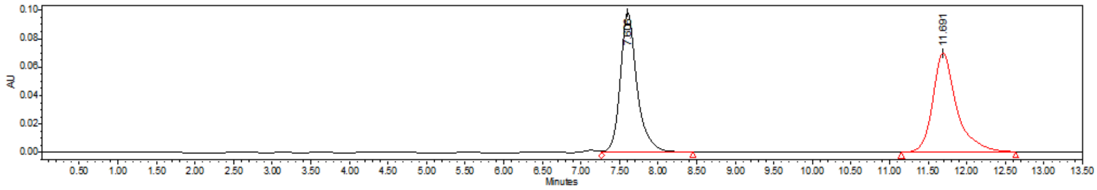

Peak	Retention Time	Area	% Area
1	7.606	1481053	49.64
2	11.691	1502503	50.36

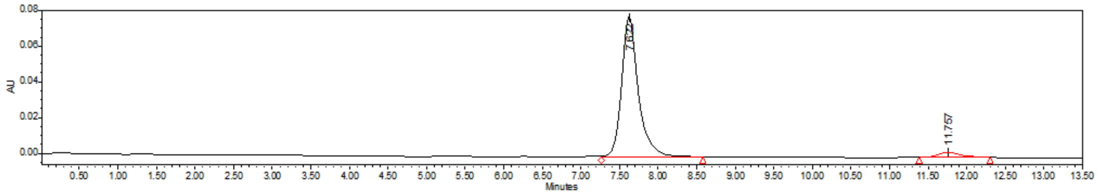

Peak	Retention Time	Area	% Area
1	7.622	1187894	95.46
2	11.757	56538	4.54

(*R*)-*N*-(*tert*-Butyl)-6-fluoro-1-oxo-2-(1-phenylvinyl)-2,3-dihydro-1*H*-indene-2-carboxamide

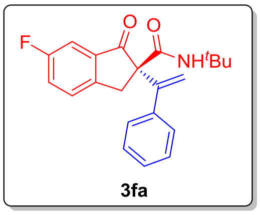

yiellow soild, m.p. 78.7–84.3; 98% yield, 85:15 e.r.; R_f = 0.55 (petroleum ether/ethyl acetate = 10/1); $[\alpha]^{23}_{D}$ = –277.9 (*c* = 0.61, in DCM).
HPLC DAICEL CHIRALCEL IA, hexane/*i*-PrOH = 90/10, flow rate 1.0 mL/min, λ = 254 nm) t_r (major) = 4.48 min, t_r (minor) = 6.53 min.
IR (neat): 2967, 1705, 1678, 1519, 1486, 1445, 1220, 1028, 879, 771, 700 cm^{-1}.
^{1}H NMR (400 MHz, $CDCl_3$) δ 7.42 – 7.29 (m, 8H), 6.51 (s, 1H), 5.50 (d, *J* = 1.6 Hz, 2H), 4.38 – 4.33 (m, 1H), 3.08 – 3.03 (m, 1H), 1.23 (s, 9H).
^{13}C{^{1}H} NMR (101 MHz, $CDCl_3$) δ 202.6 (d, J_{C-F} =3.1), 166.1, 162.4 (d, J_{C-F} =246.5), 149.3 (d, J_{C-F} =2.0), 148.7, 139.9, 136.5 (d, J_{C-F} =8.5), 128.4, 128.1, 127.9 (d, J_{C-F} =7.2), 127.2, 123.6 (d, J_{C-F} =23.7), 117.0, 110.3 (d, J_{C-F} =21.8), 68.3, 51.6, 37.2, 28.2.
^{19}F{^{1}H} NMR (376 MHz, $CDCl_3$) δ –114.3 (s, 1F).
HRMS (ESI-TOF) calcd for $C_{22}H_{22}FNNaO_2^+$ ([M]+Na^+) = 374.1527, found 374.1525.
Chiral HPLC spectrum **3fa**:

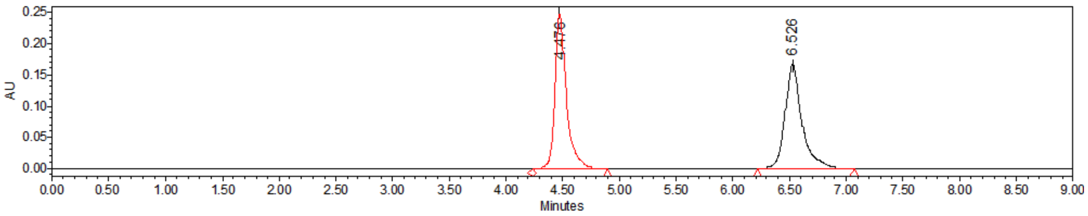

Peak	Retention Time	Area	% Area
1	4.476	1708672	49.99
2	6.526	1709285	50.01

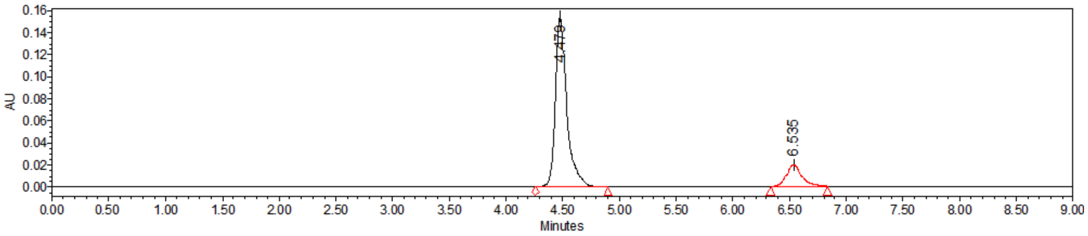

Peak	Retention Time	Area	% Area
1	4.479	1074389	84.75
2	6.535	193351	15.25

(*R*)-*N*-(3-Ethylpentan-3-yl)-1-oxo-2-(1-phenylvinyl)-2,3-dihydro-1*H*-indene-2-carboxamide

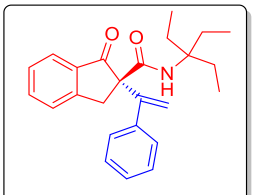

3ga

colorless oil; 98% yield, 90:10 e.r.; R_f = 0.60 (petroleum ether/ethyl acetate = 10/1); $[\alpha]^{22}_{D}$ = –293.4 (*c* = 0.54, in DCM).
HPLC DAICEL CHIRALCEL IA, hexane/*i*-PrOH = 90/10, flow rate 1.0 mL/min, λ = 254 nm) t_r (minor) = 6.34 min, t_r (major) = 8.05 min.
IR (neat): 2967, 2924, 1704, 1663, 1609, 1517, 1458, 1280, 1028, 907, 765, 700, 523 cm^{-1}.
^{1}H NMR (400 MHz, $CDCl_3$) δ 7.78 (d, *J* = 8.0 Hz, 1H), 7.59 (td, *J* = 7.6, 1.2 Hz, 1H), 7.42 – 7.26 (m, 7H), 6.27 (s, 1H), 5.61 (s, 1H), 5.55 (s, 1H), 4.45 (d, *J* = 17.6 Hz, 1H), 3.11 (d, *J* = 17.2 Hz, 1H), 1.69 – 1.55 (m, 6H), 0.65 (t, *J* = 7.6 Hz, 9H).

^{13}C{^{1}H} NMR (101 MHz, $CDCl_3$) δ 203.4, 166.1, 153.8, 148.6, 140.0, 135.8, 134.9, 128.4, 128.0, 127.6, 127.2, 126.4, 124.6, 116.7, 67.3, 60.2, 38.0, 26.6, 7.4.
HRMS (ESI-TOF) calcd for $C_{25}H_{29}NNaO_2^+$ ([M]+Na$^+$) = 398.2091, found 398.2091.
Chiral HPLC spectrum **3ga**:

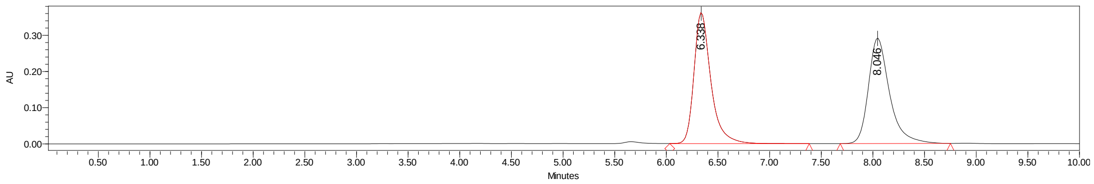

Peak	Retention Time	Area	% Area
1	6.338	4112734	49.96
2	8.046	4118682	50.04

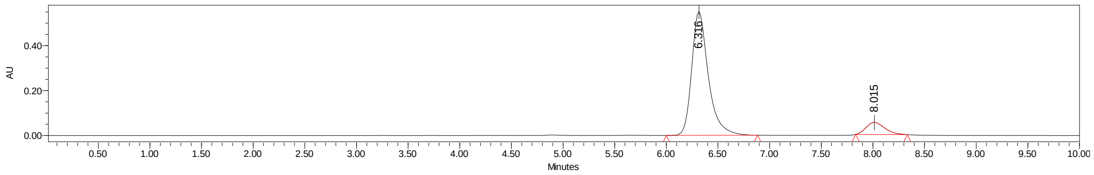

Peak	Retention Time	Area	% Area
1	6.316	6136315	90.10
2	8.015	674513	9.90

(*R*)-*N*-(*tert*-Butyl)-2-(1-(2-fluorophenyl)vinyl)-1-oxo-2,3-dihydro-1*H*-indene-2-carboxamide

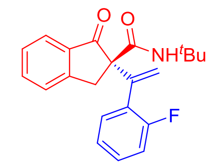

3ab

colorless oil; 94% yield, 92:8 e.r.; R_f = 0.50 (petroleum ether/ethyl acetate = 10/1); $[\alpha]^{22}_D$ = –206.9 (*c* = 0.56, in DCM).
HPLC DAICEL CHIRALCEL IA, hexane/*i*-PrOH = 90/10, flow rate 1.0 mL/min, λ = 254 nm) t_r (major) = 5.88 min, t_r (minor) = 7.98 min.
IR (neat): 2967, 1701, 1607, 1528, 1455, 1270, 1218, 910, 760 cm^{-1}.
^{1}H NMR (400 MHz, $CDCl_3$) δ 7.74 (d, *J* = 7.6, 1H), 7.60 (td, *J* = 7.6, 1.2 Hz, 1H), 7.44 – 7.42 (m, 1H), 7.38 – 7.34 (m, 1H), 7.27 – 7.22 (m, 2H), 7.09 – 7.00 (m, 2H), 6.78 (s, 1H), 5.66 (s, 1H), 5.36 (s, 1H), 4.30 (d, *J* = 17.6 Hz, 1H), 3.25 (d, *J* = 17.6 Hz, 1H), 1.24 (s, 9H).
^{13}C{^{1}H} NMR (101 MHz, $CDCl_3$) δ 204.0, 166.0, 159.6 (d, $J_{C\text{-}F}$ = 244.9), 153.8, 143.5, 135.8, 135.0, 130.4 (d, $J_{C\text{-}F}$ = 3.2), 129.5 (d, $J_{C\text{-}F}$ =8.3), 128.0 (d, $J_{C\text{-}F}$ = 15.5), 127.5, 126.4, 124.6, 123.8 (d, $J_{C\text{-}F}$ = 3.6), 120.3 (d, $J_{C\text{-}F}$ = 2.6), 115.7 (d, $J_{C\text{-}F}$ = 22.7), 67.3, 51.4, 37.9, 28.2.
^{19}F{^{1}H} NMR (376 MHz, $CDCl_3$) δ –113.0 (s, 1F)
HRMS (ESI-TOF) calcd for $C_{22}H_{22}FNNaO_2^+$ ([M]+Na$^+$) = 374.1527, found 374.1526.
Chiral HPLC spectrum **3ab**:

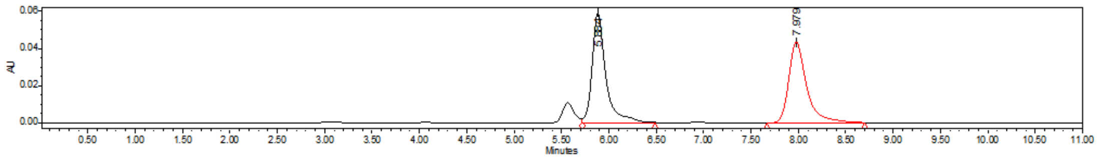

Peak	Retention Time	Area	% Area
1	5.884	592259	50.85
2	7.979	572484	49.15

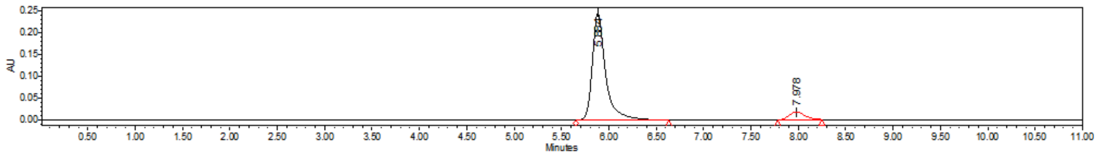

Peak	Retention Time	Area	% Area
1	5.884	2401640	92.07
2	7.978	206953	7.93

(*R*)-*N*-(*tert*-Butyl)-2-(1-(3-fluorophenyl)vinyl)-1-oxo-2,3-dihydro-1*H*-indene-2-carboxamide

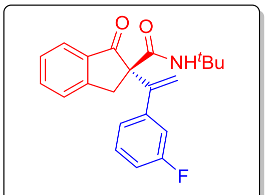

3ac

colorless oil; 89% yield, 93.5:6.5 e.r.; R_f = 0.50 (petroleum ether/ethyl acetate = 10/1); $[\alpha]^{23}_D$ = –346.2 (*c* = 0.66, in DCM).
HPLC DAICEL CHIRALCEL IA, hexane/*i*-PrOH = 90/10, flow rate 1.0 mL/min, λ = 254 nm) t_r (major) = 5.19 min, t_r (minor) = 6.33 min.
IR (neat): 2967, 1702, 1609, 1582, 1521, 1459, 1217, 927, 789, 756 cm^{-1}.
^{1}H NMR (400 MHz, $CDCl_3$) δ 7.78 (d, *J* = 7.6 Hz, 1H), 7.62 (t, *J* = 7.2, 1H), 7.44 – 7.36 (m, 2H), 7.30 – 7.24 (m, 1H), 7.11 (d, *J* = 8.0 Hz, 1H), 7.08 – 7.04 (m, 1H), 6.99 (td, *J* = 2.8, 8.4 Hz, 1H), 6.56 (s, 1H), 5.51 (s, 1H), 5.47 (s, 1H), 4.41 (d, *J* = 17.6 Hz, 1H), 3.10 (d, *J* = 17.2 Hz, 1H), 1.23 (s, 9H).
^{13}C{^{1}H} NMR (101 MHz, $CDCl_3$) δ 203.5, 166.2, 162.6 (d, J_{C-F} = 244.6), 153.7, 148.1 (d, J_{C-F} = 2.2), 142.4 (d, J_{C-F} = 7.4), 136.0, 134.9, 129.8 (d, J_{C-F} = 8.3), 127.7 , 126.5 , 124.7 , 123.0 (d, J_{C-F} = 2.8), 117.7, 114.8 (d, J_{C-F} = 21.9), 114.5 (d, J_{C-F} = 22.0), 67.1, 51.6, 37.7, 28.2.
^{19}F{^{1}H} NMR (376 MHz, $CDCl_3$) δ –112.8 (s, 1F).
HRMS (ESI-TOF) calcd for $C_{22}H_{22}FNNaO_2^+$ ([M]+Na^+) = 374.1527, found 374.1520.
Chiral HPLC spectrum **3ac**:

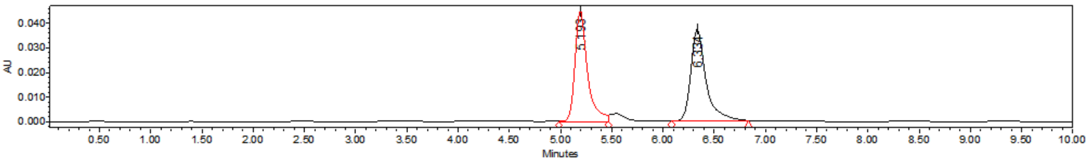

Peak	Retention Time	Area	% Area
1	5.193	380984	49.61

2	6.334	386925	50.39

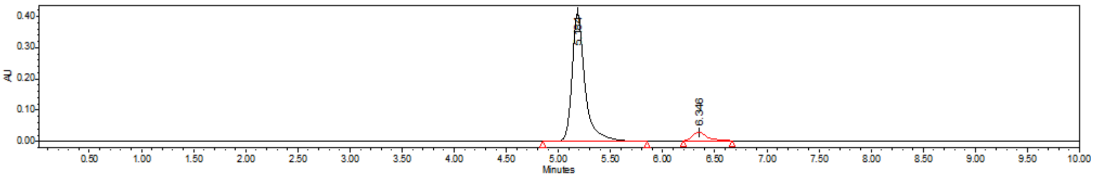

Peak	Retention Time	Area	% Area
1	5.184	3524505	93.51
2	6.346	244629	6.49

_N_-(_tert_-Butyl)-2-(1-(4-methoxyphenyl)vinyl)-1-oxo-2,3-dihydro-1_H_-indene-2-carboxamide

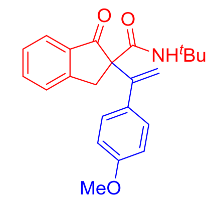

3ad

colorless oil; 99% yield, 93:7 e.r.; R_f = 0.35 (petroleum ether/ethyl acetate = 10/1); $[\alpha]^{22}_D$ = –317.3 (*c* = 0.67, in DCM).
HPLC DAICEL CHIRALCEL IG, hexane/*i*-PrOH = 90/10, flow rate 1.0 mL/min, λ = 254 nm) t_r (major) = 9.90 min, t_r (minor) = 13.13 min.
IR (neat): 2965, 1701, 1675, 1605, 1513, 1459, 1252, 1180, 1031, 909, 837, 755 cm^{-1}.
^{1}H NMR (400 MHz, $CDCl_3$) δ 7.76 (d, *J* = 7.6 Hz, 1H), 7.58 (td, *J* = 7.2, 1.2 Hz, 1H), 7.42 – 7.34 (m, 2H), 7.29 – 7.26 (m, 2H), 6.86 – 6.82 (m, 2H), 6.57 (s, 1H), 5.42 (d, *J* = 3.2 Hz, 2H), 4.40 (d, *J* = 17.6 Hz, 1H), 3.80 (s, 3H), 3.09 (d, *J* = 17.6 Hz, 1H), 1.24 (s, 9H).
^{13}C{^{1}H} NMR (101 MHz, $CDCl_3$) δ 203.5, 166.7, 159.4, 153.8, 148.4, 135.7, 135.0, 132.5, 128.5, 127.5, 126.4, 124.6, 115.5, 113.7, 67.4, 55.3, 51.5, 37.6, 28.2.
HRMS (ESI-TOF) calcd for $C_{23}H_{25}NNaO_3^+$ ([M]+Na^+) = 386.1727, found 386.1729.
Chiral HPLC spectrum **3ad**:

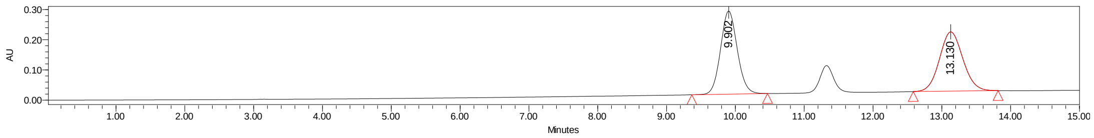

Peak	Retention Time	Area	% Area
1	9.902	4646101	50.10
2	13.130	4627149	49.90

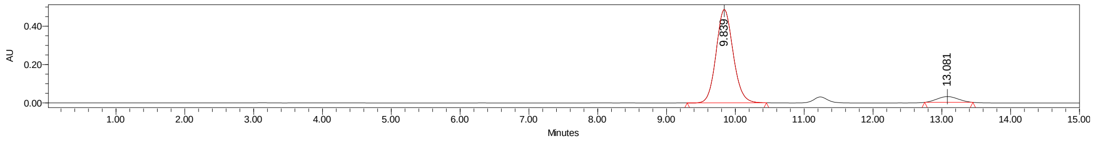

Peak	Retention Time	Area	% Area
1	9.839	8358303	92.95
2	13.081	634105	7.05

(*R*)-*N*-(*tert*-Butyl)-2-(1-(4-fluorophenyl)vinyl)-1-oxo-2,3-dihydro-1*H*-indene-2-carboxamide

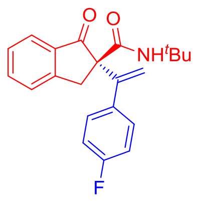

3ae

colorless solid, m.p. 102.0–108.2; 97% yield, 93:7 e.r.; R_f = 0.50 (petroleum ether/ethyl acetate = 10/1); $[\alpha]^{22}_D$ = –244.2 (*c* = 0.71, in DCM).
HPLC DAICEL CHIRALCEL IA, hexane/*i*-PrOH = 95/5, flow rate 1.0 mL/min, λ = 254 nm) t_r (minor) = 7.57 min, t_r (major) = 8.46 min.
IR (neat): 2968, 1700, 1677, 1605, 1511, 1459, 1224, 1162, 910, 843, 757 cm^{-1}.
^{1}H NMR (400 MHz, $CDCl_3$) δ 7.77 (d, *J* = 7.6 Hz, 1H), 7.61 (td, *J* = 7.6, 1.2 Hz, 1H), 7.44 – 7.36 (m, 2H), 7.33 – 7.28 (m, 2H), 7.01 – 6.97 (m, 2H), 6.60 (s, 1H), 5.46 (s, 1H), 5.40 (s, 1H), 4.40 (d, *J* = 17.6 Hz, 1H), 3.10 (d, *J* = 17.6 Hz, 1H), 1.23 (s, 9H).
^{13}C{^{1}H} NMR (101 MHz, $CDCl_3$) δ 203.6, 166.4, 162.5 (d, J_{C-F} =246) , 153.7, 148.2, 136.2 (d, J_{C-F} =3.4), 135.9, 134.9, 129.1 (d, J_{C-F} =7.9), 127.7, 126.5, 124.7, 116.9, 115.2 (d, J_{C-F} =20.9), 67.2, 51.6, 37.7, 28.2.
^{19}F{^{1}H} NMR ((376 MHz, $CDCl_3$) δ –114.1 (s, 1F).
HRMS (ESI-TOF) calcd for $C_{22}H_{22}FNNaO_2^+$ ([M]+Na^+) = 374.1527, found 374.1522.
Chiral HPLC spectrum **3ae**:

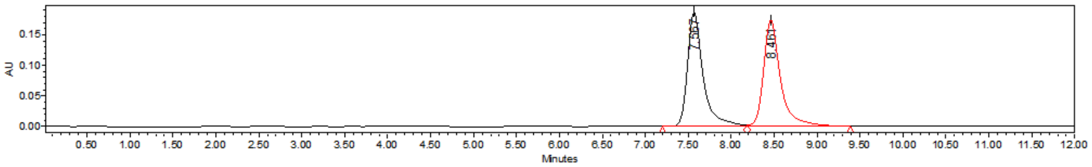

Peak	Retention Time	Area	% Area
1	7.567	2364119	49.86
2	8.461	2377814	50.14

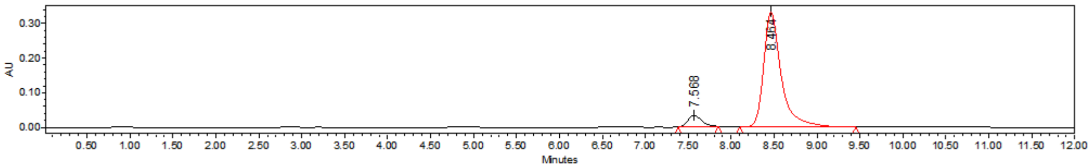

Peak	Retention Time	Area	% Area
1	7.568	363042	7.14
2	8.464	4719237	92.86

***N*-(*tert*-Butyl)-2-(1-(2-methoxyphenyl)vinyl)-1-oxo-2,3-dihydro-1*H*-indene-2-carboxamide**

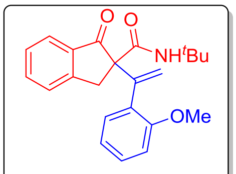

3af

colorless oil; 99% yield, 92:8 e.r.; R_f = 0.40 (petroleum ether/ethyl acetate = 10/1); $[\alpha]^{23}_{D}$ = –50.0 (*c* = 0.63, in DCM).
HPLC DAICEL CHIRALCEL IA, hexane/*i*-PrOH = 90/10, flow rate 1.0 mL/min, λ = 254 nm) t_r (major) = 5.47 min, t_r (minor) = 6.72.
IR (neat): 2964, 1698, 1601, 1526, 1458, 1258, 1022, 905, 752 cm^{-1}.
^{1}H NMR (400 MHz, $CDCl_3$) δ 7.69 (dt, *J* = 7.6, 0.8 Hz, 1H), 7.56 (td, *J* = 7.2, 1.2 Hz, 1H), 7.41 (dt, *J* = 7.6, 1.2 Hz, 1H), 7.34 – 7.30 (m, 1H), 7.28 – 7.24 (m, 1H), 7.15 (dd, *J* = 7.6, 1.6 Hz, 1H), 6.93 (td, *J* = 7.2, 1.2 Hz, 1H), 6.82 – 6.63 (m, 2H), 5.64 (d, *J* = 0.8 Hz, 1H), 5.28 (d, *J* = 0.4 Hz, 1H), 4.31 (d, *J* = 17.6 Hz, 1H), 3.36 – 3.30 (s, 4H), 1.27 (s, 9H).
^{13}C{^{1}H} NMR (101 MHz, $CDCl_3$) δ 203.8, 166.9, 156.2, 153.8, 148.5, 135.5, 135.1, 130.6, 129.9, 129.2, 127.1, 126.1, 124.1, 120.6, 118.6, 110.0, 67.0, 54.4, 51.2, 38.5, 28.3.
HRMS (ESI-TOF) calcd for $C_{23}H_{25}NNaO_3^+$ ([M]+Na^+) = 386.1727, found 386.1729.
Chiral HPLC spectrum **3af**:

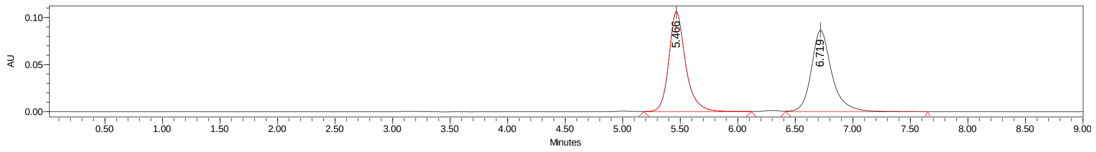

Peak	Retention Time	Area	% Area
1	5.466	1075703	50.22
2	6.719	1066359	49.78

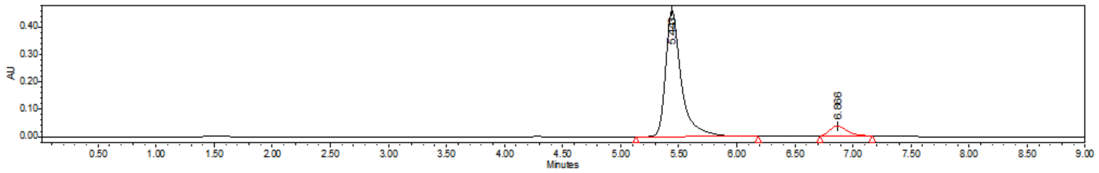

Peak	Retention Time	Area	% Area
1	5.443	4306505	91.88
2	6.866	380668	8.12

(*R*)-*N*-(*tert*-Butyl)-2-(1-(4-chlorophenyl)vinyl)-1-oxo-2,3-dihydro-1*H*-indene-2-carboxamide

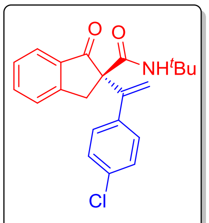

3ag

colorless oil; 93% yield, 93.5:6.5 e.r.; R_f = 0.50 (petroleum ether/ethyl acetate = 10/1); $[\alpha]^{23}_{D}$ = –349.4 (*c* = 0.67, in DCM).
HPLC DAICEL CHIRALCEL IG, hexane/*i*-PrOH = 90/10, flow rate 1.0 mL/min, λ = 254 nm) t_r (major) = 7.03 min, t_r (minor) = 8.39 min.
IR (neat): 2966, 1702, 1608, 1521, 1459, 1219, 1111, 1017, 910, 838 cm^{-1}.
^{1}H NMR (400 MHz, $CDCl_3$) δ 7.77 (d, *J* = 7.6 Hz, 1H), 7.63 – 7.59 (m, 1H), 7.43 – 7.36 (m, 2H), 7.27 (s, 4H), 6.59 (s, 1H), 5.49 (s, 1H), 5.44 (s, 1H), 4.39 (d, *J* = 17.6 Hz, 1H), 3.08 (d, *J* = 17.6 Hz, 1H), 1.23 (s, 9H).
^{13}C{^{1}H} NMR (101 MHz, $CDCl_3$) δ 203.5, 166.3, 153.7, 148.1, 138.6, 136.0, 134.9, 134.0, 128.7, 128.5, 127.7, 126.5, 124.7, 117.3, 67.0, 51.6, 37.6, 28.2.

HRMS (ESI-TOF) calcd for $C_{22}H_{22}{}^{34.9689}ClNNaO_2{}^+$ ([M]+Na$^+$) = 390.1231, found 390.1233, and $C_{22}H_{22}{}^{36.9659}ClNNaO_2{}^+$ ([M]+Na$^+$) = 392.1202, found 392.1201.
Chiral HPLC spectrum **3ag**:

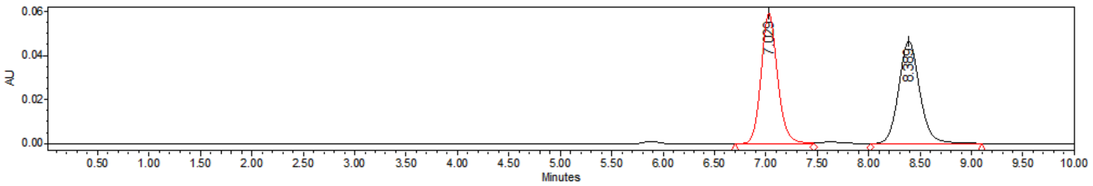

Peak	Retention Time	Area	% Area
1	7.029	659543	49.96
2	8.389	660558	50.04

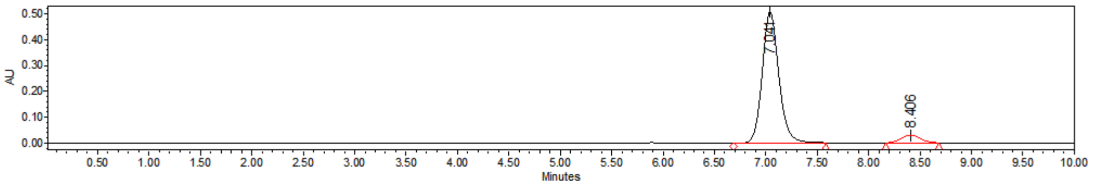

Peak	Retention Time	Area	% Area
1	7.041	5663055	93.52
2	8.406	392628	6.48

(*R*)-*N*-(*tert*-Butyl)-1-oxo-2-(1-(m-tolyl)vinyl)-2,3-dihydro-1*H*-indene-2-carboxamide

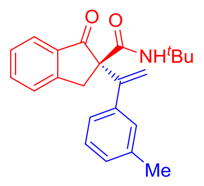

3ah

yellow soild; 99% yield, 93:7 e.r.; R_f = 0.50 (petroleum ether/ethyl acetate = 10/1); $[\alpha]^{22}_D$ = –321.6 (*c* = 0.60, in DCM).
HPLC DAICEL CHIRALCEL IA, hexane/*i*-PrOH = 90/10, flow rate 1.0 mL/min, λ = 254 nm) t_r (major) = 5.00 min, t_r (minor) = 5.56 min.
IR (neat): 2966, 1701, 1678, 1605, 1519, 1458, 1219, 1025, 917, 790, 754, 724 cm^{-1}.
^{1}H NMR (400 MHz, $CDCl_3$) δ 7.77 (d, *J* = 7.6 Hz, 1H), 7.59 (td, *J* = 7.6, 1.2 Hz, 1H), 7.42 – 7.34 (m, 2H), 7.19 (t, *J* = 7.6 Hz, 1H), 7.14 – 7.10 (m, 3H), 6.56 (s, 1H), 5.50 (s, 1H), 5.46 (s, 1H), 4.38 (d, *J* = 17.6 Hz, 1H), 3.09 (d, *J* = 17.6 Hz, 1H), 2.33 (s, 3H), 1.24 (s, 9H).
^{13}C{^{1}H} NMR (101 MHz, $CDCl_3$) δ 203.5, 166.5, 153.9, 149.0, 140.2, 138.0, 135.7, 135.0, 128.7, 128.2, 128.1, 127.5, 126.4, 124.7, 124.4, 116.7, 67.3, 51.5, 37.7, 28.2, 21.5.
HRMS (ESI-TOF) calcd for $C_{23}H_{25}NNaO_2{}^+$ ([M]+Na$^+$) = 370.1778, found 370.1778.
Chiral HPLC spectrum **3ah**:

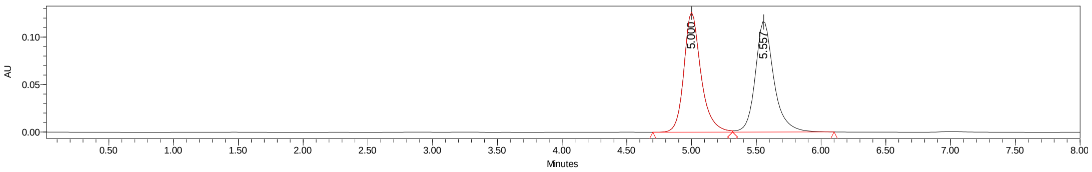

Peak	Retention Time	Area	% Area
1	5.000	1148223	50.02
2	5.557	1147084	49.98

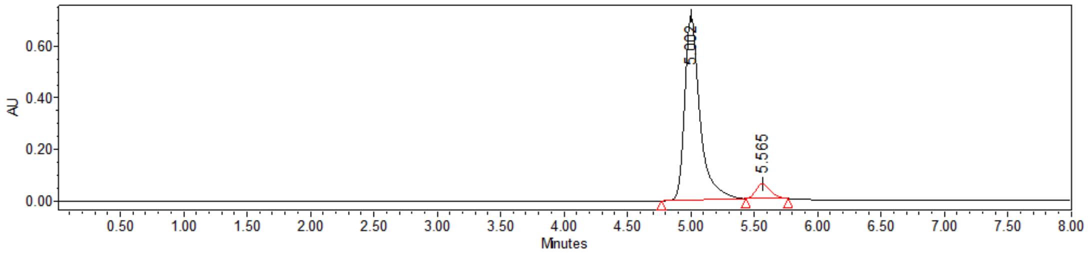

Peak	Retention Time	Area	% Area
1	5.002	5952324	92.87
2	5.565	456693	7.13

2-(1-([1,1'-Biphenyl]-4-yl)vinyl)-*N*-(*tert*-butyl)-1-oxo-2,3-dihydro-1*H*-indene-2-carboxamide

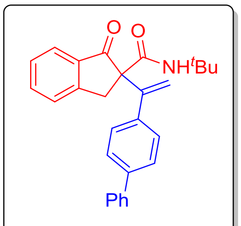

3ai

colorless oil; 77% yield, 91:9 e.r.; R_f = 0.50 (petroleum ether/ethyl acetate = 8/1); $[\alpha]^{27}_D$ = –306.6 (*c* = 0.44, in DCM).
HPLC DAICEL CHIRALCEL IG, hexane/*i*-PrOH = 90/10, flow rate 1.0 mL/min, λ = 254 nm) t_r (major) = 14.53 min, t_r (minor) = 17.16 min.
IR (neat): 2967, 2924, 1702, 1679, 1607, 1520, 1485, 1458, 1393, 1364, 1264, 1219, 1024, 910, 847, 771, 742, 698 cm^{-1}.
^{1}H NMR (400 MHz, $CDCl_3$) δ 7.79 (d, *J* = 7.6 Hz, 1H), 7.62 – 7.54 (m, 5H), 7.50 – 7.33 (m, 7H), 6.58 (s, 1H), 5.53 (d, *J* = 4.8 Hz, 2H), 4.45 (d, *J* = 17.6 Hz, 1H), 3.15 (d, *J* = 17.6 Hz, 1H), 1.25 (s, 9H).
^{13}C{^{1}H} NMR (101 MHz, $CDCl_3$) δ 203.5, 166.6, 153.9, 148.7, 140.8, 140.4, 139.1, 135.8, 135.0, 128.8, 127.7, 127.6, 127.5, 127.0, 126.5, 124.7, 116.8, 67.3, 51.6, 37.8, 28.2.
HRMS (ESI-TOF) calcd for $C_{28}H_{28}NO_2^+$ ([M]+H$^+$) = 410.2114, found 410.2109.
Chiral HPLC spectrum **3ai**:

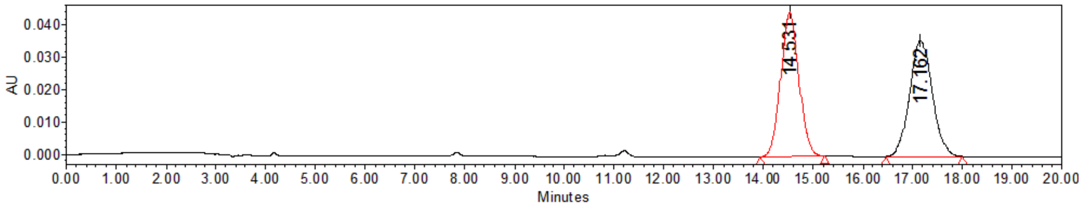

Peak	Retention Time	Area	% Area
1	14.531	1134013	49.95
2	17.162	1136253	50.05

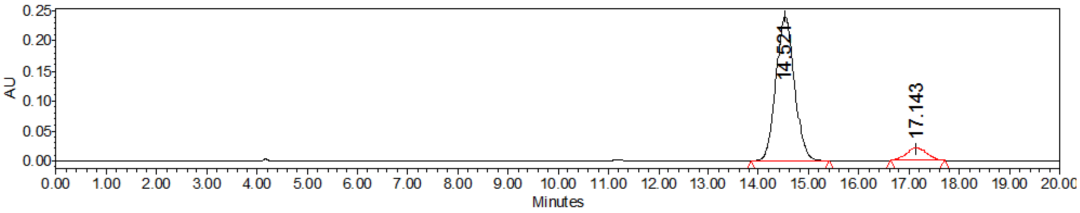

Peak	Retention Time	Area	% Area
1	14.521	6189110	91.20
2	17.143	597334	8.80

***N*-(*tert*-Butyl)-2-(1-(4-ethynylphenyl)vinyl)-1-oxo-2,3-dihydro-1*H*-indene-2-carboxamide**

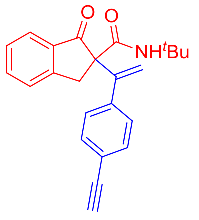

3aj

colorless oil; 58% yield, 95.5:4.5 e.r.; R_f = 0.50 (petroleum ether/ethyl acetate = 10/1); $[\alpha]^{19}_D$ = –376.8 (*c* = 0.85, in DCM).
HPLC DAICEL CHIRALCEL IG, hexane/*i*-PrOH = 90/10, flow rate 1.0 mL/min, λ = 254 nm) t_r (major) = 7.70 min, t_r (minor) = 11.03 min.
IR (neat): 3289, 2965, 2928, 1701, 1678, 1521, 1282, 1215, 1019, 909, 846, 750 cm^{-1}.
^{1}**H NMR** (400 MHz, $CDCl_3$) δ 7.77 (d, *J* = 7.6 Hz, 1H), 7.61 (td, *J* = 7.6, 1.2 Hz, 1H), 7.44 – 7.36 (m, 4H), 7.31 – 7.29 (m, 2H), 6.54 (s, 1H), 5.50 (d, *J* = 10.0 Hz, 2H), 4.42 (d, *J* = 17.6 Hz, 1H), 3.11 – 3.06 (m, 2H), 1.22 (s, 9H).
^{13}**C{^{1}H} NMR** (101 MHz, $CDCl_3$) δ 203.5, 166.3, 153.7, 148.5, 140.5, 135.9, 134.9, 132.1, 127.7, 127.2, 126.5, 124.7, 121.7, 117.5, 83.2, 78.1, 67.0, 51.6, 37.7, 28.2.
HRMS (ESI-TOF) calcd for $C_{24}H_{24}NO_2^+$ ([M]+H^+) = 358.1801, found 358.1796.
Chiral HPLC spectrum **3aj**:

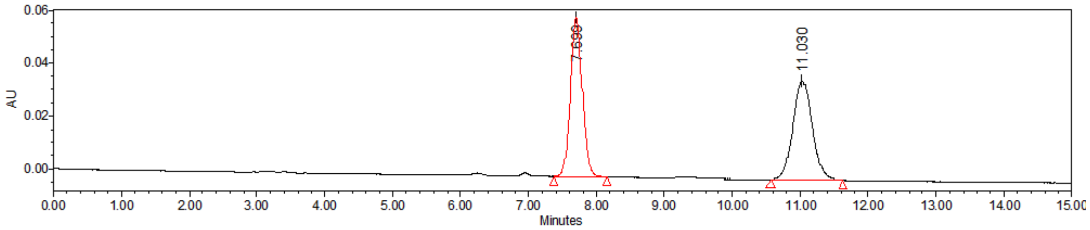

Peak	Retention Time	Area	% Area
1	7.699	744503	50.17
2	11.030	739393	49.83

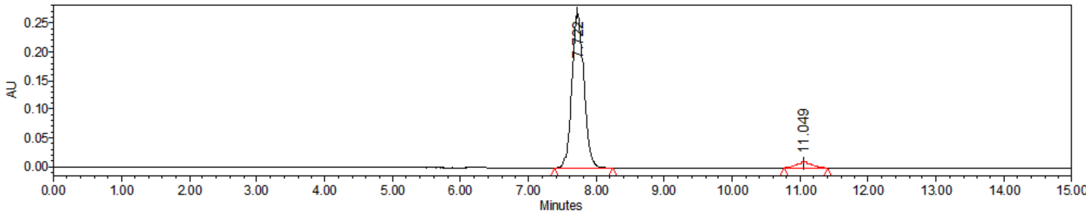

Peak	Retention Time	Area	% Area
1	7.722	3295571	95.46
2	11.049	156837	4.54

***N*-(*tert*-Butyl)-1-oxo-2-(1-(thiophen-2-yl)vinyl)-2,3-dihydro-1*H*-indene-2-carboxamide**

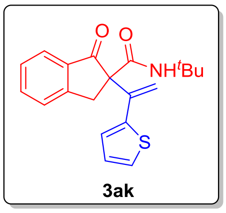

colorless oil; 99% yield, 94:6 e.r.; R_f = 0.50 (petroleum ether/ethyl acetate = 10/1); $[\alpha]^{22}_{D}$ = –314.7 (*c* = 0.71, in DCM).
HPLC DAICEL CHIRALCEL IA, hexane/*i*-PrOH = 90/10, flow rate 1.0 mL/min, λ = 254 nm) t_r (major) = 8.19 min, t_r (minor) = 9.63 min**.**
IR (neat): 2967, 1701, 1678, 1605, 1520, 1458, 1271, 1218, 905, 752, 704 cm^{-1}.
^{1}H NMR (400 MHz, $CDCl_3$) δ 7.78 (dt, *J* = 7.6, 1.2 Hz, 1H), 7.61 (td, *J* = 7.6, 1.2 Hz, 1H), 7.46 – 7.44 (m, 1H), 7.40 – 7.36 (m, 1H), 7.18 (dd, *J* = 5.2, 1.2 Hz, 1H), 7.07 (dd, *J* = 4.0, 1.2 Hz, 1H), 6.96 (dd, *J* = 5.2, 3.6 Hz, 1H), 6.42 (s, 1H), 5.60 (s, 1H), 5.31 (s, 1H), 4.51 (d, *J* = 17.2 Hz, 1H), 3.18 (d, *J* = 17.6 Hz, 1H), 1.25 (s, 9H).
^{13}C{^{1}H} NMR (101 MHz, $CDCl_3$) δ 202.74, 166.2, 154.0, 142.6, 142.1, 135.9 9, 134.9, 127.6, 127.5, 126.5, 125.5, 124.7, 124.7, 115.3, 67.4, 51.7, 37.4, 28.2.
HRMS (ESI-TOF) calcd for $C_{20}H_{21}NNaO_2S^+$ ([M]+Na^+) = 362.1185, found 362.1176.
Chiral HPLC spectrum **3ak**:

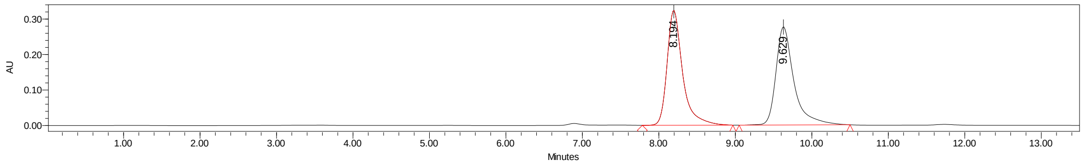

Peak	Retention Time	Area	% Area
1	8.194	4552917	50.20
2	9.629	4516851	49.80

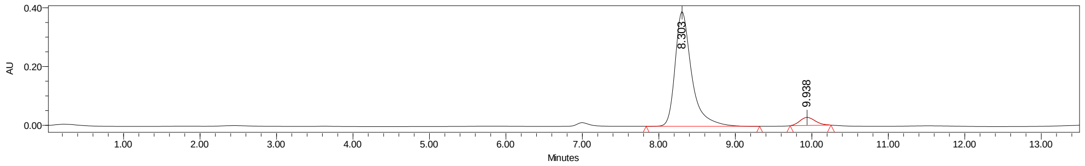

Peak	Retention Time	Area	% Area

1	8.303	5866757	93.89
2	9.938	381613	6.11

N-(*tert*-Butyl)-1-oxo-2-(1-(thiophen-3-yl)vinyl)-2,3-dihydro-1*H*-indene-2-carboxamide

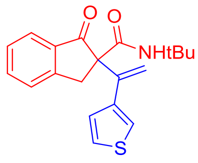

3al

yellow amorphous solid; 99% yield, 94.5:5.5 e.r.; R_f = 0.50 (petroleum ether/ethyl acetate = 10/1); $[\alpha]^{22}_D$ = –447.4 (*c* = 0.67, in DCM).
HPLC DAICEL CHIRALCEL IA, hexane/*i*-PrOH = 95/5, flow rate 1.0 mL/min, λ = 254 nm) t_r (major) = 7.22 min, t_r (minor) = 9.49 min.
IR (neat): 2968, 1702, 1676, 1608, 1525, 1459, 1273, 1219, 789, 751 cm^{-1}.
^{1}H NMR (400 MHz, $CDCl_3$) δ 7.78 (dt, *J* = 7.6, 1.2 Hz, 1H), 7.61 (td, *J* = 7.6, 1.2 Hz, 1H), 7.43 (dt, *J* = 7.6, 1.2 Hz, 1H), 7.40 – 7.36 (m, 1H), 7.30 (dd, *J* = 2.8, 1.6 Hz, 1H), 7.26 (dd, *J* = 5.2, 2.8 Hz, 1H), 7.17 (dd, *J* = 5.2, 1.6 Hz, 1H), 6.47 (s, 1H), 5.55 (s, 1H), 5.36 (s, 1H), 4.48 (d, *J* = 17.6 Hz, 1H), 3.12 (d, *J* = 17.6 Hz, 1H), 1.24 (s, 9H).
^{13}C{^{1}H} NMR (101 MHz, $CDCl_3$) δ 203.1, 166.6, 153.9, 143.5, 140.3, 135.9, 134.9, 127.6, 126.8, 126.5, 125.4, 124.7, 122.3, 114.9, 67.4, 51.6, 37.4, 28.2.
HRMS (ESI-TOF) calcd for $C_{20}H_{21}NNaO_2S^+$ ([M]+Na^+) = 362.1185, found 362.1178.
Chiral HPLC spectrum **3al**:

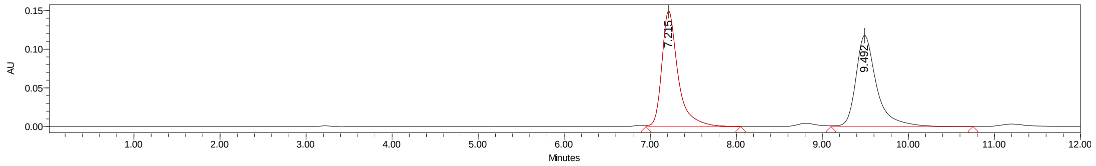

Peak	Retention Time	Area	% Area
1	7.215	1959009	49.86
2	9.492	1970333	50.14

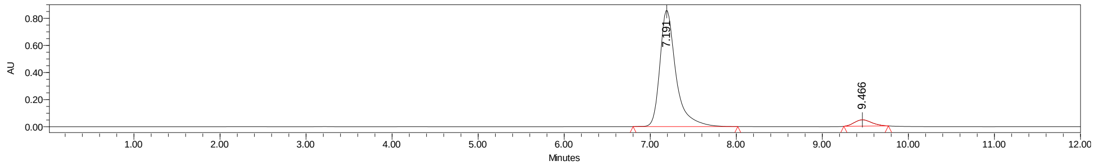

Peak	Retention Time	Area	% Area
1	7.191	11096726	94.40
2	9.466	658046	5.60

N-(*tert*-Butyl)-2-(1-cyclopropylvinyl)-1-oxo-2,3-dihydro-1*H*-indene-2-carboxamide

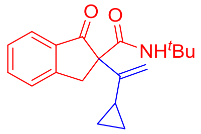

3am

colorless oil; 68% yield, 89.5:10.5 e.r.; R_f = 0.50 (petroleum ether/ethyl acetate = 10/1); $[\alpha]^{22}_{D}$ = –205.2 (*c* = 0.31, in DCM).
HPLC DAICEL CHIRALCEL IA, hexane/*i*-PrOH = 95/5, flow rate 1.0 mL/min, λ = 254 nm) t_r (major) = 5.67 min, t_r (minor) = 6.72 min**.**
IR (neat): 2967, 1698, 1530, 1459, 1270, 1221, 903, 761, 526 cm^{-1}.
^{1}H NMR (400 MHz, $CDCl_3$) δ 7.75 (d, *J* = 8.0 Hz, 1H), 7.62 (td, *J* = 7.6, 1.2 Hz, 1H), 7.48 (dt, *J* = 7.6, 1.2 Hz, 1H), 7.39 – 7.35 (m, 1H), 6.85 (s, 1H), 4.86 (s, 1H), 4.67 (d, *J* = 0.8 Hz, 1H), 4.30 (d, *J* = 17.6 Hz, 1H), 3.30 (d, *J* = 17.6 Hz, 1H), 1.35 (s, 9H), 1.27 – 1.23 (m, 1H), 0.73 – 0.66 (m, 2H), 0.54 – 0.46 (m, 2H).
^{13}C{^{1}H} NMR (101 MHz, $CDCl_3$) δ 204.9, 166.8, 153.67, 151.1, 135.8, 135.55, 127.5, 126.5, 124.4, 107.0, 68.4, 51.4, 36.8, 28.5, 13.6, 8.4, 8.1.
HRMS (ESI-TOF) calcd for $C_{19}H_{23}NNaO_2^+$ ($[M]+Na^+$) = 320.1621, found 320.1626.
Chiral HPLC spectrum **3am**:

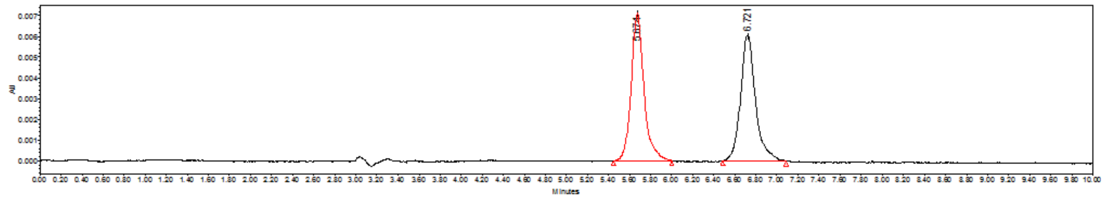

Peak	Retention Time	Area	% Area
1	5.674	60366	50.00
2	6.721	60378	50.00

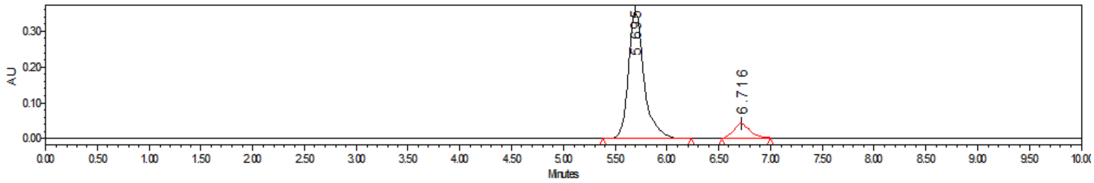

Peak	Retention Time	Area	% Area
1	5.695	3693554	89.49
2	6.716	433597	10.51

***N*-(*tert*-Butyl)-1-oxo-2-(pent-1-en-2-yl)-2,3-dihydro-1*H*-indene-2-carboxamide**

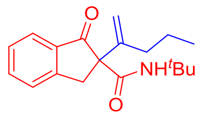

3an

colorless oil; 54% yield, 90:10 e.r.; R_f = 0.50 (petroleum ether/ethyl acetate = 10/1); $[\alpha]^{26}_{D}$ = –212.3 (*c* = 0.34, in DCM).
HPLC DAICEL CHIRALCEL IA, hexane/*i*-PrOH = 95/5, flow rate 1.0 mL/min, λ = 254 nm) t_r (major) = 5.36 min, t_r (minor) = 6.41 min.
IR (neat): 3368, 2962, 2931, 1698, 1682, 1591, 1528, 1456, 1364, 1267, 1223, 903, 775, 747 cm^{-1}.
^{1}H NMR (400 MHz, $CDCl_3$) δ 7.75 (d, *J* = 7.6 Hz, 1H), 7.64 – 7.59 (m, 1H), 7.46 (d, *J* = 7.6 Hz, 1H), 7.39 – 7.35 (m, 1H), 6.79 (s, 1H), 5.02 – 4.95 (m, 2H), 4.24 (d, *J* = 18.0 Hz, 1H), 3.05 (d, *J* = 17.6 Hz, 1H), 2.10 – 1.94 (m, 2H), 1.56 – 1.46 (m, 2H), 1.34 (s, 9H), 0.92 (t, *J* = 7.2 Hz, 3H).
^{13}C{^{1}H} NMR (101 MHz, $CDCl_3$) δ 205.0, 166.8, 153.6, 149.0, 135.8, 135.5, 127.5, 126.5, 124.4, 111.2, 68.2, 51.4, 36.7, 34.5, 28.5, 21.0, 13.9.
HRMS (ESI-TOF) calcd for $C_{19}H_{25}NNaO_2^+$ ($[M]+Na^+$) = 322.1778, found 322.1773.

Chiral HPLC spectrum **3an**:

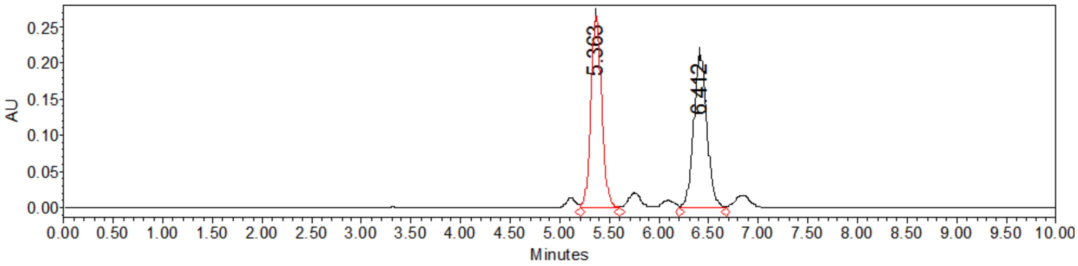

Peak	Retention Time	Area	% Area
1	5.363	1977504	49.97
2	6.412	1979887	50.03

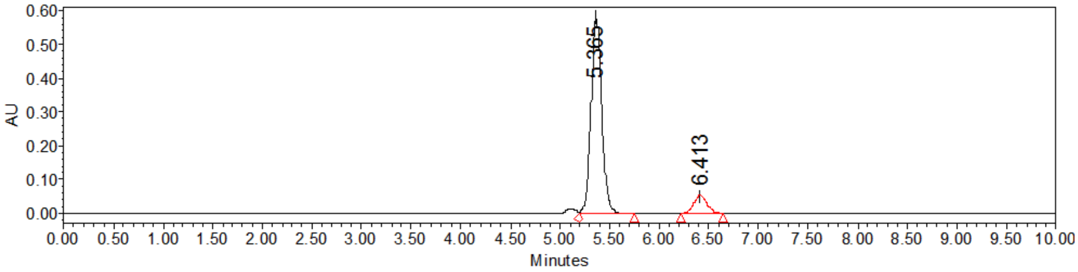

Peak	Retention Time	Area	% Area
1	5.365	4308660	90.22
2	6.413	466931	9.78

***N*-(*tert*-Butyl)-1-oxo-2-(4-phenylbut-1-en-2-yl)-2,3-dihydro-1*H*-indene-2-carboxamide**

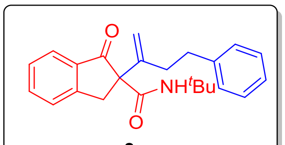

3ao

colorless oil; 95% yield, 91.5:8.5 e.r.; R_f = 0.50 (petroleum ether/ethyl acetate = 10/1); $[\alpha]^{26}_D$ = –177.2 (*c* = 0.25, in DCM).
HPLC DAICEL CHIRALCEL IA, hexane/*i*-PrOH = 90/10, flow rate 1.0 mL/min, λ = 254 nm) t_r (major) = 5.21 min, t_r (minor) = 9.52 min.
IR (neat): 3364, 2962, 2926, 2857, 1699, 1606, 1529, 1455, 1364, 1267, 1223, 1153, 905, 775, 749, 699 cm^{-1}.
^{1}H NMR (400 MHz, $CDCl_3$) δ 7.75 (d, *J* = 7.6 Hz, 1H), 7.63 – 7.57 (m, 1H), 7.44 (d, *J* = 7.6 Hz, 1H), 7.36 (t, *J* = 7.2 Hz, 1H), 7.28 – 7.24 (m, 2H), 7.19 – 7.14 (m, 3H), 6.84 (s, 1H), 5.10 – 5.07 (m, 2H), 4.23 (d, *J* = 17.6 Hz, 1H), 3.02 (d, *J* = 17.6 Hz, 1H), 2.82 – 2.76 (m, 2H), 2.43 – 2.28 (m, 2H), 1.34 (s, 9H).
^{13}C{^{1}H} NMR (101 MHz, $CDCl_3$) δ 205.0, 166.6, 153.6, 148.4, 141.4, 135.9, 135.4, 128.4, 128.3, 127.6, 126.5, 126.0, 124.5, 112.0, 68.1, 51.4, 45.2, 36.7, 34.2, 28.5.
HRMS (ESI-TOF) calcd for $C_{24}H_{27}NNaO_2^+$ ([M]+Na^+) = 384.1934, found 384.1939.
Chiral HPLC spectrum **3ao**:

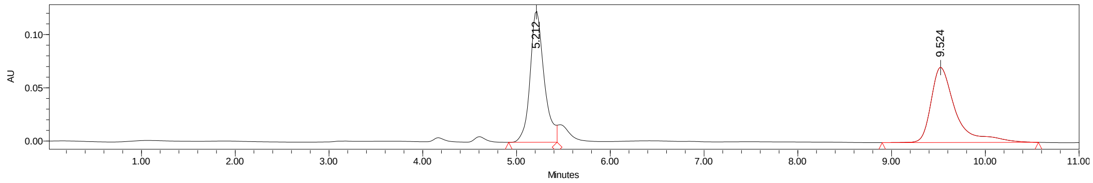

Peak	Retention Time	Area	% Area
1	5.212	1280314	49.93
2	9.524	1283765	50.07

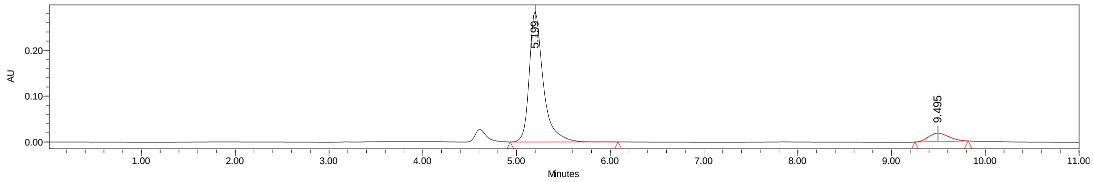

Peak	Retention Time	Area	% Area
1	5.199	2942846	91.44
2	9.495	275513	8.56

***N*-(*tert*-Butyl)-2-(non-1-en-8-yn-2-yl)-1-oxo-2,3-dihydro-1*H*-indene-2-carboxamide**

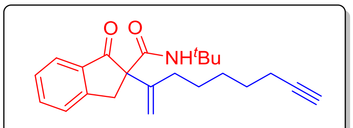

3ap

colorless oil; 53% yield, 89.5:10.5 e.r.; R_f = 0.50 (petroleum ether/ethyl acetate = 10/1); $[\alpha]^{26}_D$ = –174.0 (*c* = 0.65, in DCM).
HPLC DAICEL CHIRALCEL IG, hexane/*i*-PrOH = 90/10, flow rate 1.0 mL/min, λ = 254 nm) t_r (major) = 7.40 min, t_r (minor) = 8.24 min.
IR (neat): 3366, 3306, 2932, 2861, 1698, 1529, 1456, 1364, 1268, 1223, 903, 774, 632 cm^{-1}.
^{1}H NMR (400 MHz, $CDCl_3$) δ 7.75 (d, *J* = 7.6 Hz, 1H), 7.62 (td, *J* = 7.6, 1.2 Hz, 1H), 7.46 (dt, *J* = 7.6, 0.8 Hz, 1H), 7.40 – 7.36 (m, 1H), 6.80 (s, 1H), 5.02 (t, *J* = 1.2 Hz, 1H), 4.96 (t, *J* = 1.6 Hz, 1H), 4.24 (d, *J* = 17.6 Hz, 1H), 3.04 (d, *J* = 18.0 Hz, 1H), 2.18 (td, *J* = 6.8, 2.8 Hz, 2H), 2.13 – 1.97 (m, 2H), 1.93 (t, *J* = 2.8 Hz, 1H), 1.56 – 1.46 (m, 4H), 1.44 – 1.39 (m, 2H), 1.34 (s, 9H).
^{13}C{^{1}H} NMR (101 MHz, $CDCl_3$) δ 205.0, 166.7, 153.6, 149.0, 135.8, 135.5, 127.5, 126.5, 124.4, 111.3, 84.4, 68.3, 68.2, 51.4, 36.8, 32.3, 29.7, 28.5, 28.3, 27.5, 18.3.
HRMS (ESI-TOF) calcd for $C_{23}H_{29}NNaO_2^+$ ([M]+Na^+) = 374.2090, found 370.2080.
Chiral HPLC spectrum **3ap**:

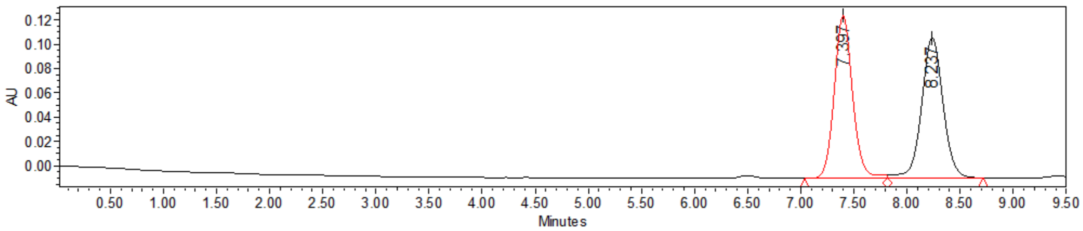

Peak	Retention Time	Area	% Area
1	7.397	1606684	49.76

2	8.237	1622001	50.24

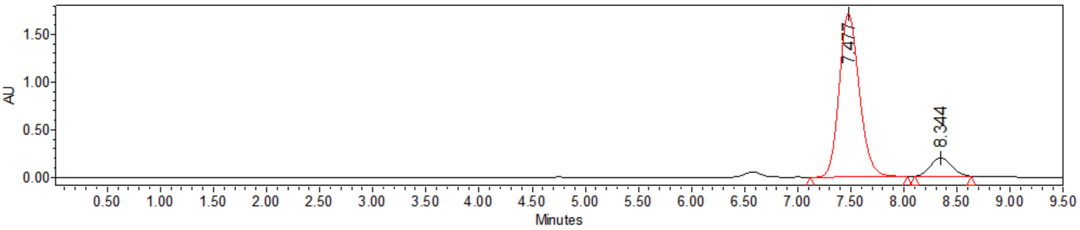

Peak	Retention Time	Area	% Area
1	7.477	22598835	89.41
2	8.344	2676433	10.59

***N*-(*tert*-Butyl)-2-(oct-1-en-7-yn-2-yl)-1-oxo-2,3-dihydro-1*H*-indene-2-carboxamide**

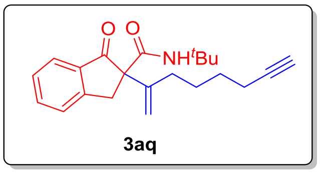

colorless oil; 38% yield, 86.5:13.5 e.r.; R_f = 0.50 (petroleum ether/ethyl acetate = 10/1); $[\alpha]^{25}_D$ = –139.0 (*c* = 0.21, in DCM).
HPLC DAICEL CHIRALCEL IG, hexane/*i*-PrOH = 90/10, flow rate 1.0 mL/min, λ = 254 nm) t_r (major) = 7.37 min, t_r (minor) = 8.56 min**.**
IR (neat): 3366, 3298, 2932, 2866, 1698, 1591, 1530, 1457, 1433, 1270, 1223, 902, 634 cm^{-1}.
^{1}H NMR (400 MHz, $CDCl_3$) δ 7.75 (dt, *J* = 7.6, 1.2 Hz, 1H), 7.64 – 7.59 (m, 1H), 7.46 (dt, *J* = 7.6, 1.2 Hz, 1H), 7.40 – 7.35 (m, 1H), 6.81 (s, 1H), 5.04 (t, *J* = 1.2 Hz, 1H), 4.98 (t, *J* = 1.6 Hz, 1H), 4.24 (d, *J* = 17.6 Hz, 1H), 3.04 (d, *J* = 18.0 Hz, 1H), 2.19 (td, *J* = 6.8, 2.8 Hz, 2H), 2.12 – 1.98 (m, 2H), 1.93 (t, *J* = 2.8 Hz, 1H), 1.65 – 1.57 (m, 2H), 1.55 – 1.49 (m, 2H), 1.34 (s, 9H).
^{13}C{^{1}H} NMR (101 MHz, $CDCl_3$) δ 205.0, 166.7, 153.5, 148.7, 135.8, 135.5, 127.6, 126.5, 124.4, 111.4, 84.2, 68.5, 68.1, 51.4, 36.8, 31.8, 28.5, 28.1, 27.0, 18.3.
HRMS (ESI-TOF) calcd for $C_{22}H_{27}NNaO_2^+$ ($[M+Na]^+$) = 360.1934, found 360.1927.
Chiral HPLC spectrum **3aq**:

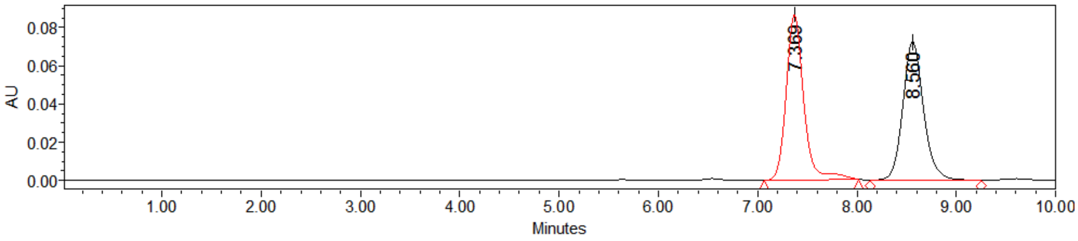

Peak	Retention Time	Area	% Area
1	7.369	1067659	50.15
2	8.560	1061129	49.85

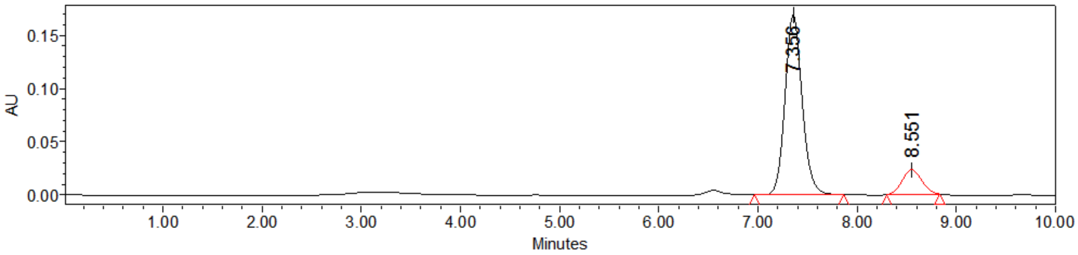

Peak	Retention Time	Area	% Area
1	7.356	2014399	86.34
2	8.551	318601	13.66

***N*-(*tert*-Butyl)-1-oxo-2-(3-(((5aS,8aS)-2,2,7,7-tetramethyltetrahydro-5*H*-bis([1,3]dioxolo)[4,5-b:4',5'-d]pyran-5-yl)methoxy)prop-1-en-2-yl)-2,3-dihydro-1*H*-indene-2-carboxamide**

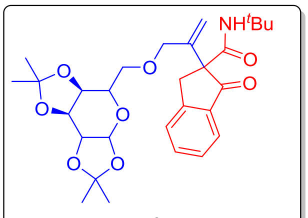

3ar

colorless oil; 53% yield, 4:1 dr; R_f = 0.20 (petroleum ether/ethyl acetate = 10/1); $[\alpha]^{27}_D$ = –120.5 (*c* = 0.37, in DCM).
IR (neat): 3367, 2981, 2932, 1700, 1607, 1529, 1459, 1377, 1257, 1212, 1171, 1108, 1069, 1004, 895, 864, 775, 738 cm^{-1}.
^{1}H NMR (400 MHz, $CDCl_3$) δ 7.74 (d, *J* = 8.0 Hz, 1H), 6.63 – 7.57 (m, 1H), 7.48 – 7.44 (m, 1H), 7.36 (t, *J* = 7.6 Hz, 1H), 6.80 (s, 1H), 5.48 (d, *J* = 4.8 Hz, 1H), 5.33 – 5.32 (m, 1H), 5.21 – 5.19 (m, 1H), 4.51 (dd, *J* = 8.0, 2.4 Hz, 1H), 4.39 – 4.08 (m, 3H), 4.06 (S, 1H), 4.03 – 3.95 (m, 1H), 3.84 (td, *J* = 6.4, 1.6 Hz, 1H), 3.66 – 3.44 (m, 1H), 3.20 – 3.06 (m, 1H), 1.54 (s, 1H), 1.41 (s, 1H), 1.36 – 1.26 (m, 15H).
^{13}C{^{1}H} NMR (101 MHz, $CDCl_3$) δ 204.6, 166.3, 153.7, 145.2, 135.7, 135.2, 127.5, 126.5, 124.4, 114.2, 109.2, 108.6, 96.3, 71.9, 71.0, 70.6, 70.6, 69.2, 66.6, 65.8, 51.5, 36.9, 28.4, 26.2, 26.0, 25.0, 24.4.
HRMS (ESI-TOF) calcd for $C_{29}H_{40}NO_8^+$ ($[M]+H^+$) = 530.2748, found 530.2739.

***N*-(*tert*-Butyl)-1-oxo-2-(1-phenylvinyl)-1,2,3,4-tetrahydronaphthalene-2-carboxamide**

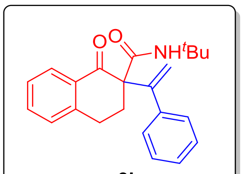

3ha

colorless oil; 95% yield, 91:9 e.r.; R_f = 0.45 (petroleum ether/ethyl acetate = 10/1); $[\alpha]^{22}_D$ = –162.0 (*c* = 0.65, in DCM).
HPLC DAICEL CHIRALCEL IA, hexane/*i*-PrOH = 95/5, flow rate 1.0 mL/min, λ = 254 nm) t_r (major) = 6.00 min, t_r (minor) = 7.85 min.
IR (neat): 2966, 1676, 1600, 1516, 1452, 1222, 918, 776, 742, 704 cm^{-1}.
^{1}H NMR (400 MHz, $CDCl_3$) δ 8.08 (dd, *J* = 8.0, 1.6 Hz, 1H), 7.46 (td, *J* = 7.2, 1.2 Hz, 1H), 7.37 – 7.29 (m, 6H), 7.14 (d, *J* = 7.6 Hz, 1H), 6.46 (s, 1H), 5.48 (s, 1H), 5.14 (s, 1H), 2.79 – 2.65 (m, 3H), 2.34 – 2.29 (m, 1H), 1.43 (s, 9H).

^{13}C{^{1}H} NMR (101 MHz, $CDCl_3$) δ 198.4, 168.4, 147.6, 143.6, 139.6, 133.7, 131.7, 128.6, 128.4, 128.2, 128.2, 127.8, 126.7, 120.6, 64.4, 51.8, 29.3, 28.4, 25.5.
HRMS (ESI-TOF) calcd for $C_{23}H_{25}NNaO_2^+$ ([M]+Na$^+$) = 370.1778, found 370.1771.
Chiral HPLC spectrum **3ha**:

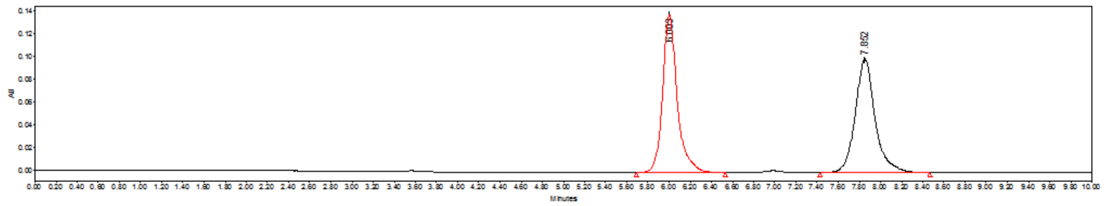

Peak	Retention Time	Area	% Area
1	6.003	1332357	50.51
2	7.852	1305303	49.49

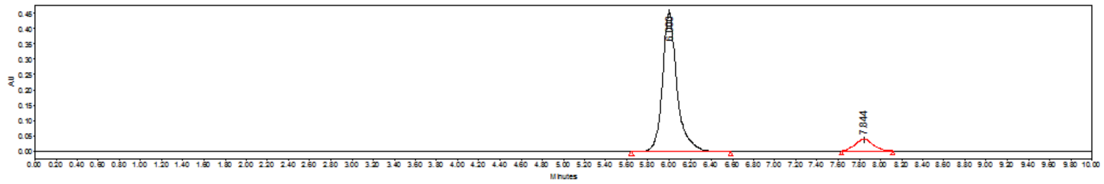

Peak	Retention Time	Area	% Area
1	6.000	4294704	90.84
2	7.844	433115	9.16

***N*-(*tert*-Butyl)-2-(1-(4-fluorophenyl)vinyl)-1-oxo-1,2,3,4-tetrahydronaphthalene-2-carboxamide**

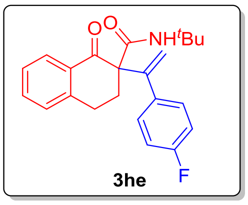

3he

colorless amorphous solid; 65% yield, 94:6 e.r.; R_f = 0.55 (petroleum ether/ethyl acetate = 10/1); $[\alpha]^{22}_D$ = –153.7 (*c* = 0.95, in DCM).
HPLC DAICEL CHIRALCEL IA, hexane/*i*-PrOH = 95/5, flow rate 1.0 mL/min, λ = 254 nm) t_r (major) = 6.10 min, t_r (minor) = 7.80 min.
IR (neat): 2926, 1677, 1600, 1512, 1453, 1224, 1162, 919, 843, 743 cm^{-1}.
^{1}H NMR (400 MHz, $CDCl_3$) δ 8.08 (dd, *J* = 8.0, 1.6 Hz, 1H), 7.47 (td, *J* = 7.6, 1.6 Hz, 1H), 7.34 – 7.29 (m, 3H), 7.16 (d, *J* = 7.6 Hz, 1H), 7.03 – 6.97 (m, 2H), 6.48 (s, 1H), 5.42 (s, 1H), 5.14 (s, 1H), 2.77 – 2.59 (m, 3H), 2.34 – 2.24 (m, 1H), 1.42 (s, 9H).
^{13}C{^{1}H} NMR (101 MHz, $CDCl_3$) δ 196.4, 168.2, 162.6 (d, J_{C-F} = 246.6), 146.8, 143.6, 135.7 (d, J_{C-F} = 3.4), 133.8, 131.7, 129.5 (d, J_{C-F} = 8.0), 128.6, 128.3, 126.8, 120.5, 115.3 (d, J_{C-F} = 21.2), 64.3, 51.8, 29.5, 28.4, 25.5.
^{19}F{^{1}H} NMR (376 MHz, $CDCl_3$) δ –113.7 (s, 1F)
HRMS (ESI-TOF) calcd for $C_{23}H_{24}FNNaO_2^+$ ([M]+Na$^+$) = 388.1683, found 388.1379.
Chiral HPLC spectrum **3he**:

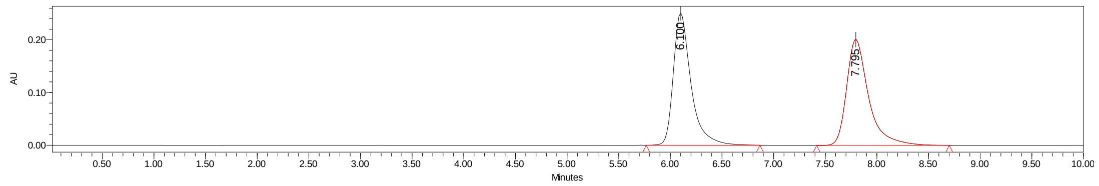

Peak	Retention Time	Area	% Area
1	6.100	2975977	50.03
2	7.795	2972460	49.97

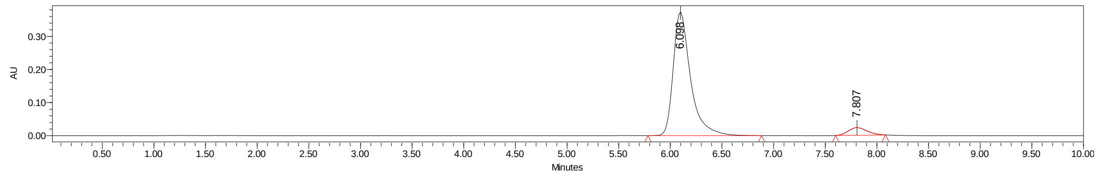

Peak	Retention Time	Area	% Area
1	6.098	4348362	93.75
2	7.807	290080	6.25

***N*-(*tert*-Butyl)-2-(1-cyclopropylvinyl)-1-oxo-1,2,3,4-tetrahydronaphthalene-2-carboxamide**

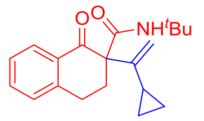

3hm

colorless oil; 32% yield, 93.5:6.5 e.r.; R_f = 0.55 (petroleum ether/ethyl acetate = 10/1); $[\alpha]^{22}_D$ = –214.0 (c = 0.32, in DCM).

HPLC DAICEL CHIRALCEL IA, hexane/*i*-PrOH = 95/5, flow rate 1.0 mL/min, λ = 254 nm) t_r (major) = 5.46 min, t_r (minor) = 6.12 min.

IR (neat): 2964, 1675, 1520, 1453, 1296, 1220, 904, 826, 741 cm^{-1}.

^{1}H NMR (400 MHz, $CDCl_3$) δ 8.07 (dd, J = 8.0, 1.6 Hz, 1H), 7.47 (td, J = 7.6, 1.6 Hz, 1H), 7.30 (t, J = 7.6 Hz, 1H), 7.21 (d, J = 7.6 Hz, 1H), 6.56 (s, 1H), 4.73 (s, 1H), 4.55 (s, 1H), 2.97 – 2.81 (m, 2H), 2.66 – 2.63 (m, 2H), 1.38 (s, 9H), 1.34 – 1.28 (m, 1H), 0.85 – 0.72 (m, 2H), 0.60 – 0.48 (m, 2H).

^{13}C{^{1}H} NMR (101 MHz, $CDCl_3$) δ 197.7, 168.2, 149.6, 144.2, 133.7, 132.4, 128.6, 128.0, 126.6, 109.4, 65.7, 51.4, 29.4, 28.5, 26.1, 13.2, 9.2, 8.0.

HRMS (ESI-TOF) calcd for $C_{20}H_{25}NNaO_2^+$ ($[M]+Na^+$) = 334.1778, found 334.1774.

Chiral HPLC spectrum **3hm**:

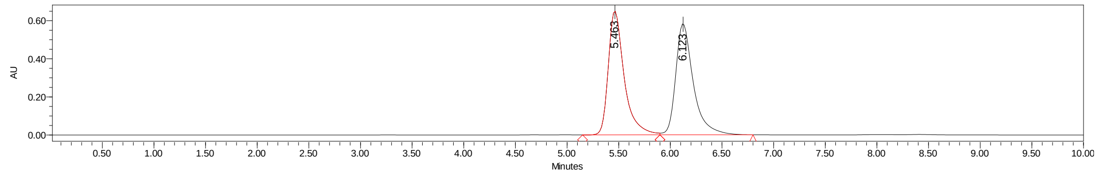

Peak	Retention Time	Area	% Area
1	5.463	7011398	50.05
2	6.123	6998020	49.95

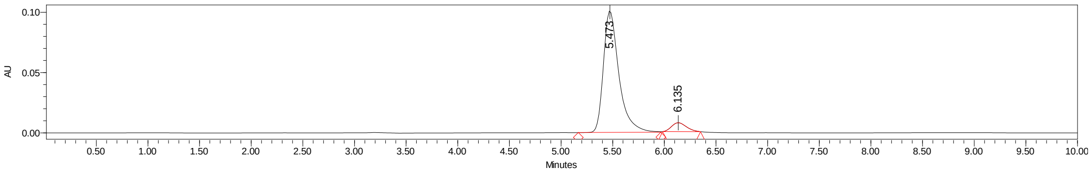

Peak	Retention Time	Area	% Area
1	5.473	1051077	93.50
2	6.135	73110	6.50

N-Isopropyl-5-oxo-6-(1-phenylvinyl)-6,7,8,9-tetrahydro-5H-benzo[7]annulene-6-carboxamide

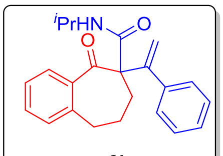

3ia

colorless oil; 27% yield, 69:31 e.r.; R_f = 0.50 (petroleum ether/ethyl acetate = 10/1); $[\alpha]^{26}_D$ = +34.8 (*c* = 0.71, in DCM).
HPLC DAICEL CHIRALCEL IB, hexane/*i*-PrOH = 95/5, flow rate 1.0 mL/min, λ = 254 nm) t_r (minor) = 5.96 min, t_r (major) = 7.13 min.
IR (neat): 2967, 1671, 1599, 1514, 1451, 1243, 957, 771, 704 cm^{-1}.
^{1}H NMR (400 MHz, $CDCl_3$) δ 87.41 – 7.34 (m, 2H), 7.28 – 7.21 (m, 6H), 7.08 (d, *J* = 7.6 Hz, 1H), 6.80 (d, *J* = 7.6 Hz, 1H), 5.42 (dd, *J* = 32.8, 1.6 Hz, 2H), 4.18 – 4.09 (m, 1H), 2.88 – 2.81 (m, 1H), 2.66 – 2.58 (m, 1H), 2.40 – 2.33 (m, 1H), 2.06 – 1.98 (m, 2H), 1.58 – 1.54 (m, 1H), 1.17 (d, *J* = 6.4 Hz, 6H).
^{13}C{^{1}H} NMR (101 MHz, $CDCl_3$) δ 209.3, 167.3, 149.6, 140.7, 139.9, 138.5, 132.4, 128.8, 128.4, 128.3, 128.0, 127.7, 126.8, 118.7, 66.9, 42.0, 31.2, 29.4, 22.6, 22.5, 22.2.
HRMS (ESI-TOF) calcd for $C_{23}H_{25}NNaO_2^+$ ([M]+Na^+) = 370.1778, found 370.1775.
Chiral HPLC spectrum **3ia**:

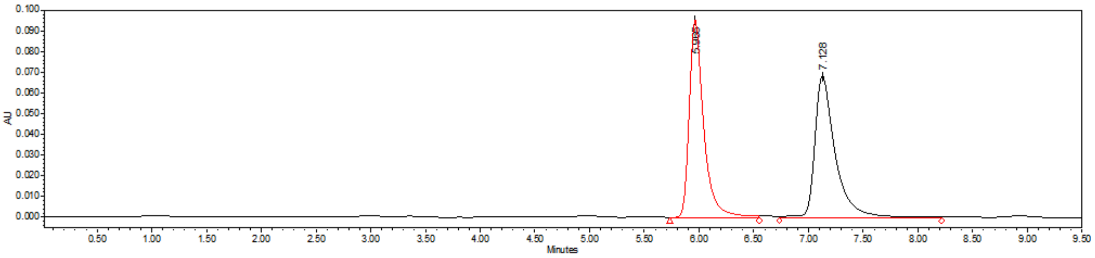

Peak	Retention Time	Area	% Area
1	5.965	874243	50.21
2	7.128	867012	49.79

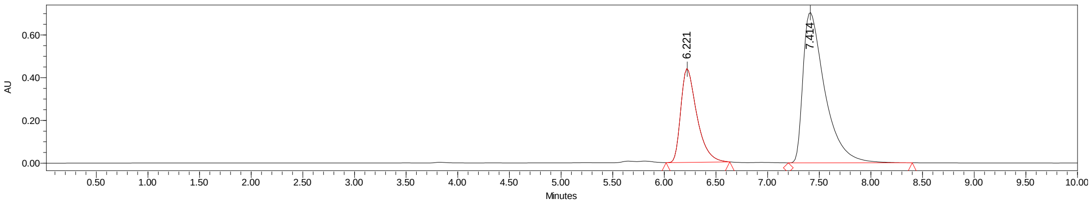

Peak	Retention Time	Area	% Area
1	5.874	4840274	30.96

2	6.975	10793575	69.04

***N*-(*tert*-Butyl)-2-oxo-1-(1-phenylvinyl)cyclopentane-1-carboxamide**

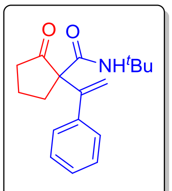

3ja

colorless oil; 49% yield, 87.5:12.5 e.r.; R_f = 0.50 (petroleum ether/ethyl acetate = 10/1); $[\alpha]^{23}_D$ = −134.7 (*c* = 0.27, in DCM).
HPLC DAICEL CHIRALCEL IB, hexane/*i*-PrOH = 98/2, flow rate 1.0 mL/min, λ = 254 nm) t_r (major) = 11.01 min, t_r (minor) = 12.36 min**.**
IR (neat): 2967, 1732, 1675, 1518, 1453, 1272, 1223, 1140, 919, 778, 703 cm^{-1}.
^{1}H NMR (400 MHz, $CDCl_3$) δ 7.25 – 7.19 (m, 5H), 6.27 (s, 1H), 5.37 (s, 1H), 5.24 (s, 1H), 2.76 – 2.68 (m, 1H), 2.36 – 2.19 (m, 2H), 1.93 – 1.86 (m, 1H), 1.84 – 1.75 (m, 1H), 1.65 – 1.53 (m, 1H), 1.23 (s, 9H).
^{13}C{^{1}H} NMR (101 MHz, $CDCl_3$) δ 215.6, 166.9, 147.8, 139.5, 128.3, 128.0, 127.6, 118.0, 67.2, 51.4, 38.4, 32.6, 28.3, 19.0.
HRMS (ESI-TOF) calcd for $C_{18}H_{23}NNaO_2^+$ ([M]+Na$^+$) = 308.1621, found 308.1619.
Chiral HPLC spectrum **3ja**:

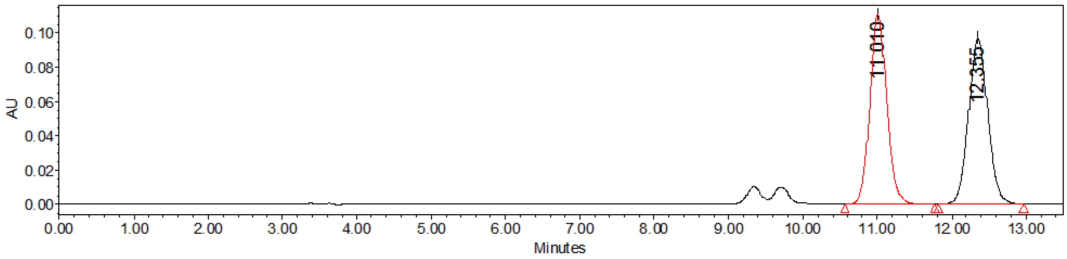

Peak	Retention Time	Area	% Area
1	11.010	1756466	50.11
2	12.355	1748609	49.89

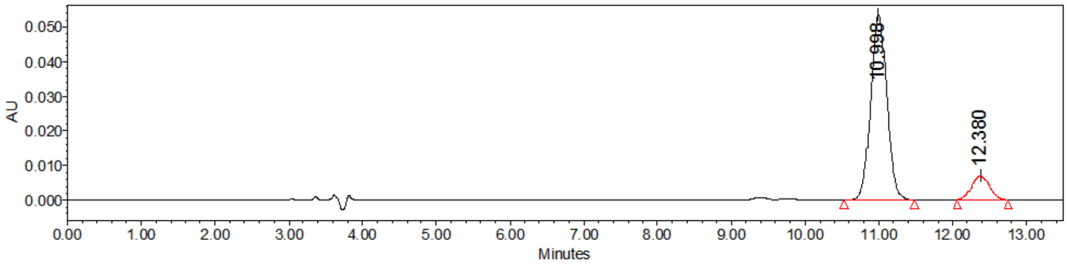

Peak	Retention Time	Area	% Area
1	10.998	822090	87.49
2	12.380	117527	12.51

***N*-Benzyl-1-oxo-2-(1-phenylvinyl)-2,3-dihydro-1*H*-indene-2-carboxamide**

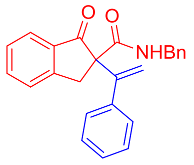

3ka

colorless oil; 99% yield, 90:10 e.r.; R_f = 0.40 (petroleum ether/ethyl acetate = 7/1); $[\alpha]^{22}_{D}$ = -294.5 (*c* = 0.67, in DCM).
HPLC DAICEL CHIRALCEL IA, hexane/*i*-PrOH = 80/20, flow rate 1.0 mL/min, λ = 254 nm) t_r (major) = 11.65 min, t_r (minor) = 16.89 min.
IR (neat): 3384, 3031, 2926, 1703, 1670, 1607, 1518, 1461, 1278, 1028, 911, 781, 724, 699 cm^{-1}.
^{1}H NMR (400 MHz, $CDCl_3$) δ 7.79 (d, *J* = 8.0 Hz, 1H), 7.60 (td, *J* = 7.6, 1.2 Hz, 1H), 7.43 – 7.36 (m, 2H), 7.32 – 7.26 (m, 5H), 7.26 – 7.21 (m, 3H), 7.10 – 7.04 (m, 3H), 5.51 (d, *J* = 8.0 Hz, 2H), 4.53 – 4.40 (m, 2H), 4.34 (dd, *J* = 15.2, 5.6 Hz, 1H), 3.18 (d, *J* = 17.6 Hz, 1H).
^{13}C{^{1}H} NMR (101 MHz, $CDCl_3$) δ 203.1, 167.9, 153.8, 148.1, 139.8, 137.8, 135.9, 134.8, 128.6, 128.5, 128.1, 127.7, 127.5, 127.3, 127.2, 126.5, 124.8, 117.4, 66.7, 44.4, 37.9.
HRMS (ESI-TOF) calcd for $C_{25}H_{22}NO_2^+$ ($[M]+H^+$) = 368.1645, found 368.1648.
Chiral HPLC spectrum **3ka**:

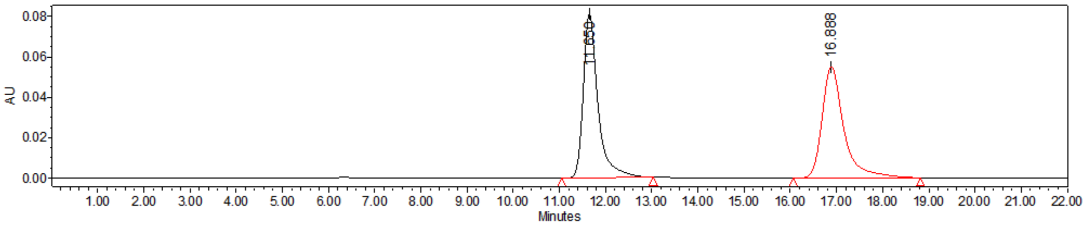

Peak	Retention Time	Area	% Area
1	11.650	1788328	49.87
2	16.888	1797499	50.13

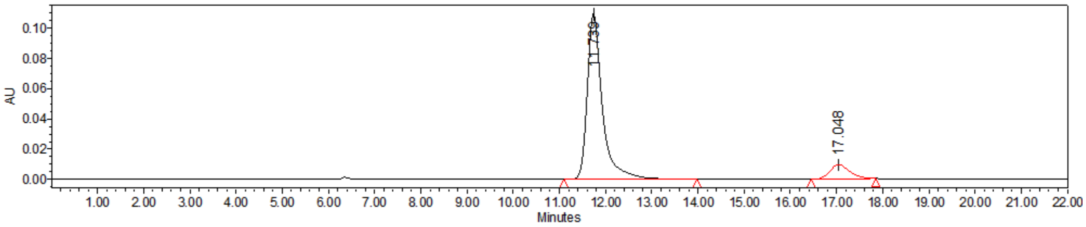

Peak	Retention Time	Area	% Area
1	11.739	2493204	89.91
2	17.048	279930	10.09

***N*-(4-Methoxyphenyl)-1-oxo-2-(1-phenylvinyl)-2,3-dihydro-1*H*-indene-2-carboxamide**

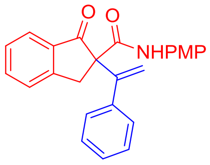

3la

colorless oil; 98% yield; 89:11 e.r.; R_f = 0.35 (petroleum ether/ethyl acetate = 6/1); $[\alpha]^{22}_D$ = -221.1 (*c* = 0.81, in DCM).
HPLC (Chiralcel **IA**, hexane/*i*-PrOH = 70/30, flow rate 1.0 mL/min, λ = 254 nm) t_r (major) = 14.79 min, t_r (minor) = 45.34 min.
IR (neat): 3345, 2931, 2835, 1696, 1599, 1510, 1463, 1276, 1238, 1176, 1109, 1031, 911, 702 cm^{-1}.
^{1}H NMR (400 MHz, $CDCl_3$) δ 8.46 (s, 1H), 7.70 (d, *J* = 7.6 Hz, 1H), 7.50 (td, *J* = 7.2, 1.2 Hz, 1H), 7.33 – 7.17 (m, 9H), 6.74 – 6.70 (m, 2H), 5.54 (s, 1H), 5.42 (s, 1H), 4.33 (d, *J* = 17.6 Hz, 1H), 3.64 (s, 3H), 3.12 (d, *J* = 18.0 Hz, 1H).
^{13}C{^{1}H} NMR (101 MHz, $CDCl_3$) δ 203.3, 165.6, 156.5, 153.6, 148.2, 139.7, 136.0, 134.7, 130.7, 128.4, 128.0, 127.7, 127.4, 126.4, 124.7, 121.8, 117.6, 114.0, 67.1, 55.4, 37.3.
HRMS (ESI-TOF) calcd for $C_{25}H_{22}NO_3^+$ ([M]+H^+) = 384.1594, found 384.1596.

Chiral HPLC spectrum **3la**:

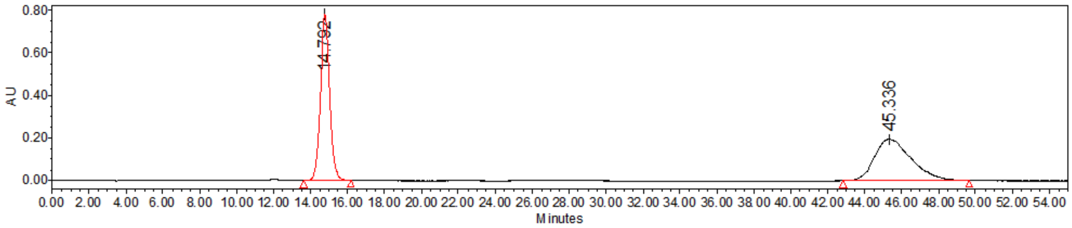

Peak	Retention Time	Area	% Area
1	14.792	27286097	50.46
2	45.336	26786029	49.54

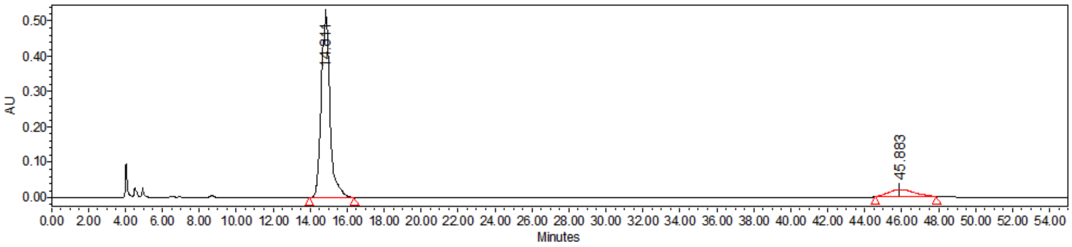

Peak	Retention Time	Area	% Area
1	14.811	16658850	88.90
2	45.883	2080405	11.10

***tert*-Butyl 1-oxo-2-(1-phenylvinyl)-2,3-dihydro-1*H*-indene-2-carboxylate**

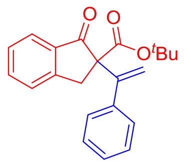

5aa

colorless oil; 47% yield; 97.5:2.5 e.r.; R_f = 0.45 (petroleum ether/ethyl acetate = 10/1); $[\alpha]^{22}_D$ = 139.1 (*c* = 0.41, in DCM).
HPLC (Chiralcel **IG**, hexane/*i*-PrOH = 95/5, flow rate 1.0 mL/min, λ = 254 nm) t_r (minor) = 6.94 min, t_r (major) = 8.52 min.
IR (neat): 2977, 2929, 1708, 1606, 1461, 1390, 1254, 1211, 1147, 1092, 1015, 907, 843, 717 cm^{-1}.
^{1}H NMR (400 MHz, $CDCl_3$) δ 7.76 (d, *J* = 7.6 Hz, 1H), 7.55 (t, *J* = 7.6 Hz, 1H), 7.39 – 7.21 (m, 7H), 5.37 (s, 1H), 5.20 (s, 1H), 4.11 (d, *J* = 17.2 Hz, 1H), 3.25 (d, *J* = 16.8 Hz, 1H), 1.18 (s, 9H).

^{13}C{^{1}H} NMR (101 MHz, $CDCl_3$) δ 200.5, 168.7, 152.7, 147.6, 141.0, 135.5, 128.3, 127.8, 127.5, 126.8, 126.3, 124.9, 116.5, 82.4, 67.2, 40.3, 27.4.
HRMS (ESI-TOF) calcd for $C_{22}H_{22}NaO_3^+$ ([M]+Na$^+$) = 357.1461, found 357.1464.
Chiral HPLC spectrum **5aa**:

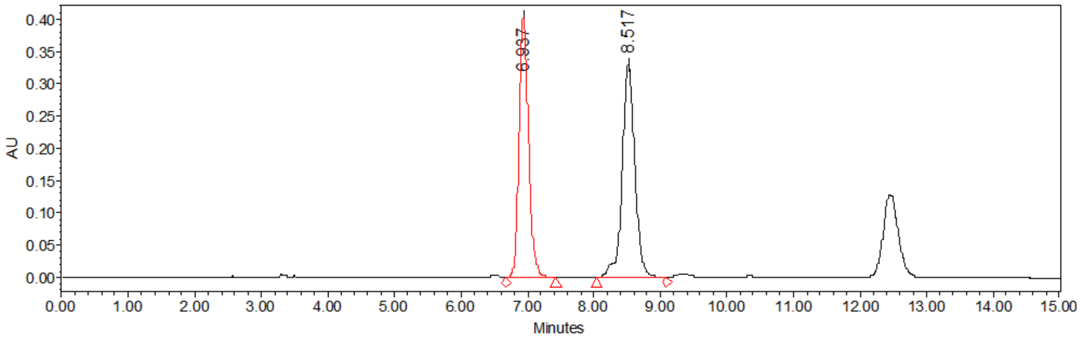

Peak	Retention Time	Area	% Area
1	6.937	3983555	49.13
2	8.517	4124678	50.87

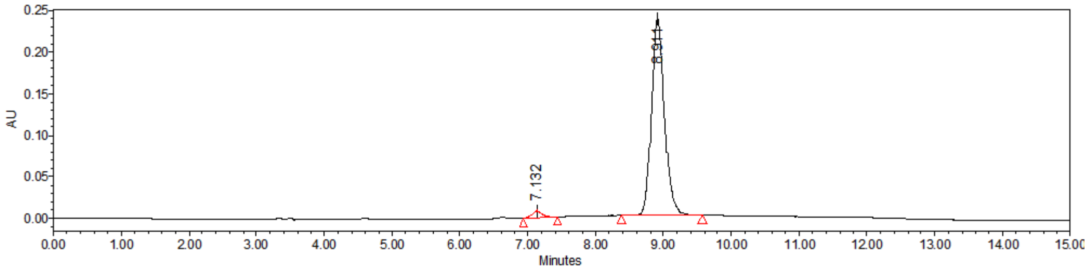

Peak	Retention Time	Area	% Area
1	7.132	83764	2.56
2	8.911	3188232	97.44

Isopropyl 1-oxo-2-(1-phenylvinyl)-2,3-dihydro-1*H*-indene-2-carboxylate

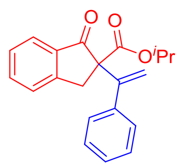

5ba

colorless oil; 72% yield; 93.5:6.5 e.r.; R_f = 0.40 (petroleum ether/ethyl acetate = 10/1); $[\alpha]^{22}_D$ = -387.4 (*c* = 0.31, in DCM).
HPLC (Chiralcel **IG**, hexane/*i*-PrOH = 90/10, flow rate 1.0 mL/min, λ = 254 nm) t_r (major) = 8.96 min, t_r (minor) = 9.99 min.
IR (neat): 2981, 2934, 2361, 1710, 1606, 1464, 1251, 1211, 1106, 1015, 913, 835, 763, 704 cm^{-1}.
^{1}H NMR (400 MHz, $CDCl_3$) δ 7.84 (d, *J* = 7.6 Hz, 1H), 7.63 (td, *J* = 7.6, 1.2 Hz, 1H), 7.46 – 7.39 (m, 2H), 7.36 – 7.27 (m, 5H), 5.48 (s, 1H), 5.30 (s, 1H), 4.92 (hept, *J* = 6.4 Hz, 1H), 4.22 (d, *J* = 17.2 Hz, 1H), 3.34 (d, *J* = 17.2 Hz, 1H), 1.16 (d, *J* = 6.4 Hz, 3H), 0.90 (d, *J* = 6.4 Hz, 3H).
^{13}C NMR (101 MHz, $CDCl_3$) δ 200.2, 169.3, 152.6, 147.2, 140.7, 135.6, 135.0, 128.4, 127.9, 127.6, 126.8, 126.4, 125.0, 116.8, 69.8, 66.6, 40.3, 21.5, 20.9.

HRMS (ESI-TOF) calcd for $C_{21}H_{20}NaO_3^+$ ([M]+Na$^+$) = 343.1305, found 343.1308.
Chiral HPLC spectrum **5ba**:

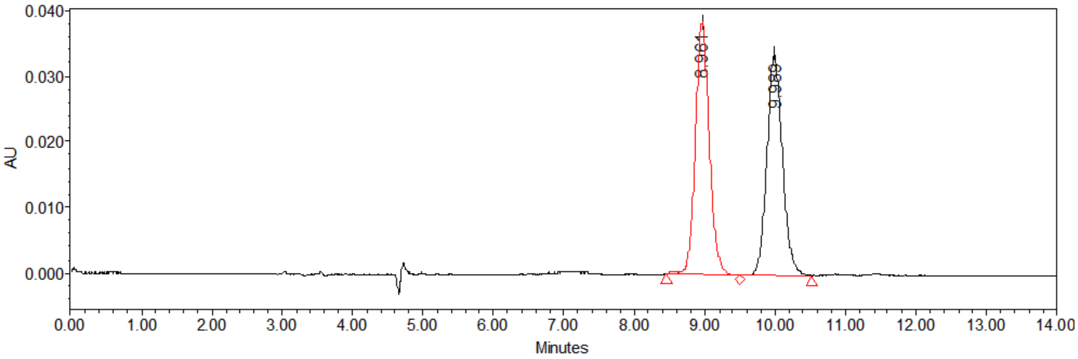

Peak	Retention Time	Area	% Area
1	8.961	522538	50.12
2	9.989	519984	49.88

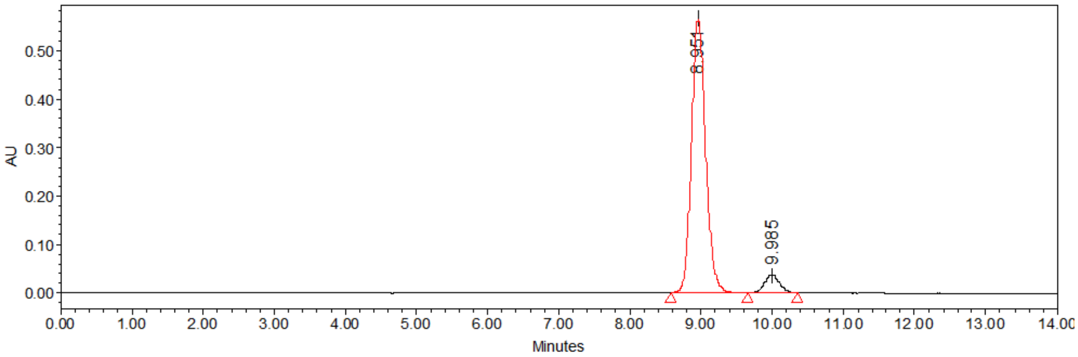

Peak	Retention Time	Area	% Area
1	8.951	7709922	93.42
2	9.985	542783	6.58

Isopropyl 5-chloro-1-oxo-2-(1-phenylvinyl)-2,3-dihydro-1*H*-indene-2-carboxylate

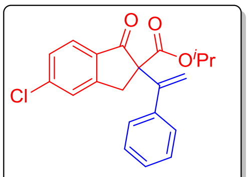

5ca

colorless oil; 89% yield; 91.5:8.5 e.r.; R_f = 0.45 (petroleum ether/ethyl acetate = 10/1); $[\alpha]^{22}_D$ = -283.3 (*c* = 0.53, in DCM).
HPLC (Chiralcel **IA**, hexane/*i*-PrOH = 98/2, flow rate 1.0 mL/min, λ = 254 nm) t_r (minor) = 11.83 min, t_r (major) = 12.97 min.
IR (neat): 2984, 2932, 2361, 1712, 1599, 1581, 1252, 1205, 1106, 1069, 912, 839, 776, 701 cm^{-1}.
^{1}**H NMR** (400 MHz, $CDCl_3$) δ 7.76 (d, *J* = 8.4 Hz, 1H), 7.44 – 7.38 (m, 1H), 7.32 – 7.26 (m, 5H), 5.49 (s, 1H), 5.30 (s, 1H), 4.92 (hept, *J* = 6.4 Hz, 1H), 4.18 (d, *J* = 17.2 Hz, 1H), 3.30 (d, *J* = 17.2 Hz, 1H), 1.16 (d, *J* = 6.4 Hz, 3H), 0.92 (d, *J* = 6.4 Hz, 3H).

^{13}C{^{1}H} NMR (101 MHz, $CDCl_3$) δ 198.8, 169.0, 154.0, 146.8, 142.3, 140.44, 133.4, 128.8, 128.4, 127.8, 126.8, 126.6, 126.1, 117.1, 70.0, 66.8, 39.8, 21.4, 20.9.
HRMS (ESI-TOF) calcd for $C_{21}H_{19}Cl^{34.9689}NaO_3^+$ ([M]+Na$^+$) = 377.0915, found 377.0918, and $C_{21}H_{19}Cl^{36.9659}NaO_3^+$ ([M]+Na$^+$) = 379.0885, found 379.0885.
Chiral HPLC spectrum **5ca**:

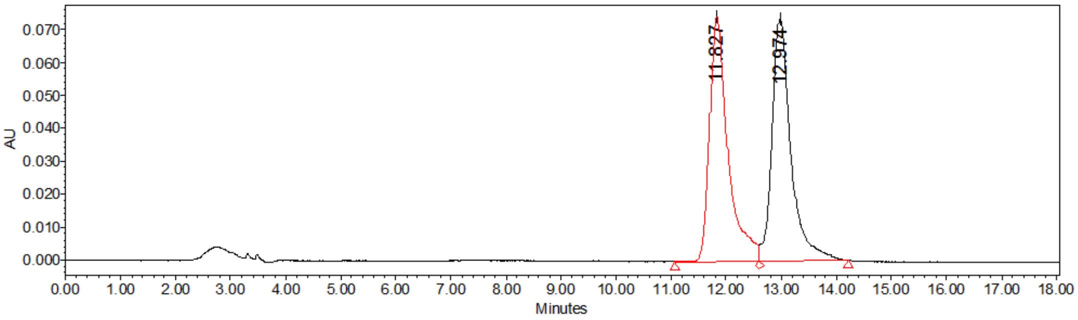

Peak	Retention Time	Area	% Area
1	11.827	1734392	49.97
2	12.974	1736785	50.03

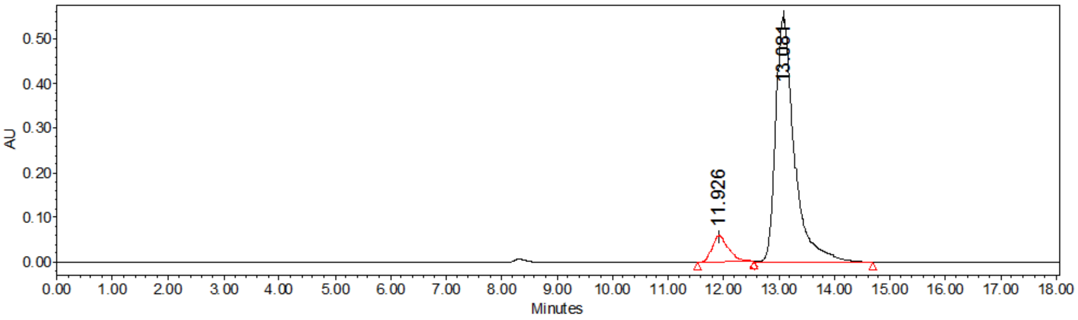

Peak	Retention Time	Area	% Area
1	11.926	1165748	8.32
2	13.081	12839662	91.68

Isopropyl 6-methyl-1-oxo-2-(1-phenylvinyl)-2,3-dihydro-1*H*-indene-2-carboxylate

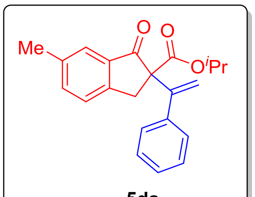

5da

colorless oil; 89% yield; 91:9 e.r.; R_f = 0.45 (petroleum ether/ethyl acetate = 10/1); $[\alpha]^{22}_D$ = -212.7 (*c* = 0.26, in DCM).
HPLC (Chiralcel **IG**, hexane/*i*-PrOH = 95/5, flow rate 1.0 mL/min, λ = 254 nm) t_r (major) = 10.81 min, t_r (minor) = 14.82 min.
IR (neat): 2980, 2930, 2361, 1619, 1494, 1378, 1252, 1195, 1151, 1106, 1030, 950, 830, 776, 701 cm^{-1}.
^{1}H NMR (400 MHz, $CDCl_3$) δ 7.64 (s, 1H), 7.45 (dd, *J* = 8.0, 1.6 Hz, 1H), 7.35 – 7.26 (m, 6H), 5.47 (s, 1H), 5.28 (s, 1H), 4.91 (hept, *J* = 6.4 Hz, 1H), 4.17 (d, *J* = 17.2 Hz, 1H), 3.29 (d, *J* = 17.2 Hz, 1H), 2.41 (s, 3H), 1.15 (d, *J* = 6.0 Hz, 3H), 0.90 (d, *J* = 6.4 Hz, 3H).

$^{13}C\{^1H\}$ NMR (101 MHz, $CDCl_3$) δ 200.3, 169.4, 150.0, 147.3, 140.7, 137.8, 136.9, 135.2, 128.3, 127.6, 126.8, 126.1, 124.8, 116.7, 69.7, 66.9, 40.0, 21.4, 21.1 20.9.
HRMS (ESI-TOF) calcd for $C_{22}H_{22}NaO_3^+$ ([M]+Na$^+$) = 357.1461, found 357.1461.
Chiral HPLC spectrum **5da**:

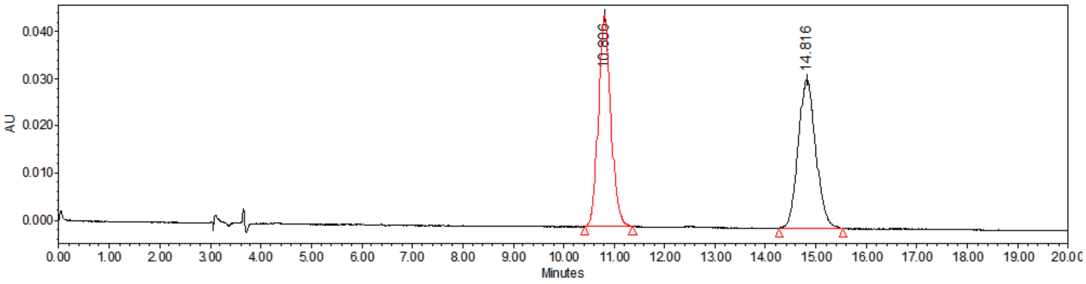

Peak	Retention Time	Area	% Area
1	10.806	769884	50.17
2	14.816	764587	49.83

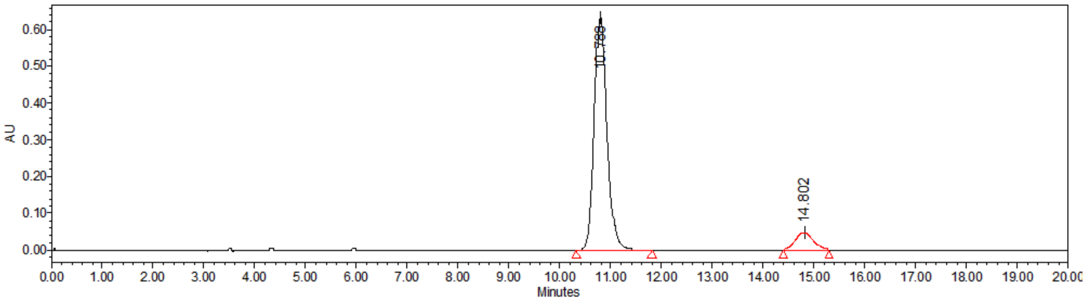

Peak	Retention Time	Area	% Area
1	10.788	11051803	90.97
2	14.802	1097264	9.03

Isopropyl 2-(1-(3-fluorophenyl)vinyl)-1-oxo-2,3-dihydro-1*H*-indene-2-carboxylate

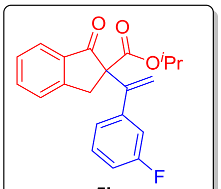

5bc

colorless oil; 40% yield; 87:13 e.r.; R_f = 0.45 (petroleum ether/ethyl acetate = 10/1); $[\alpha]^{26}_D$ = -40.8 (*c* = 0.17, in DCM).
HPLC (Chiralcel **IG**, hexane/*i*-PrOH = 95/5, flow rate 1.0 mL/min, λ = 254 nm) t_r (major) = 10.23 min, t_r (minor) = 13.10 min.
IR (neat): 2982, 2935, 1711, 1608, 1581, 1464, 1252, 1211, 1186, 1106, 1016, 927, 903, 788, 757 cm^{-1}.
^1H NMR (400 MHz, $CDCl_3$) δ 7.84 (d, *J* = 7.6 Hz, 1H), 7.64 (t, *J* = 7.6 Hz, 1H), 7.48 – 7.40 (m, 2H), 7.30 – 7.25 (m, 1H), 7.13 – 7.06 (m, 2H), 6.98 (td, *J* = 8.4, 2.4 Hz, 1H), 5.49 (s, 1H), 5.34 (s, 1H), 4.98 – 4.89 (m, 1H), 4.20 (d, *J* = 17.2 Hz, 1H), 3.32 (d, *J* = 6.8 Hz, 1H), 1.16 (d, *J* = 6.4 Hz, 3H), 0.94 (d, *J* = 6.0 Hz, 3H).
$^{13}C\{^1H\}$ NMR (101 MHz, $CDCl_3$) δ 199.9, 169.1, 162.7 (d, J_{C-F} =244.8) 152.5, 146.1 (d, J_{C-F} =2.2), 143.0 (d, J_{C-F} =7.5), 135.7, 134.9, 129.8 (d, J_{C-F} =8.6), 127.9, 126.4, 125.1, 122.4 (d, J_{C-F} =2.9), 117.8, 114.5 (d, J_{C-F} =21.0), 114.0 (d, J_{C-F} =22.2), 69.9, 66.3, 40.2, 21.4, 20.9.
$^{19}F\{^1H\}$ NMR (376 MHz, $CDCl_3$) δ –112.9 (s, 1F).

HRMS (ESI-TOF) calcd for $C_{21}H_{19}FNaO_3^+$ ([M]+Na$^+$) = 361.1210, found 361.1212.
Chiral HPLC spectrum **5bc**:

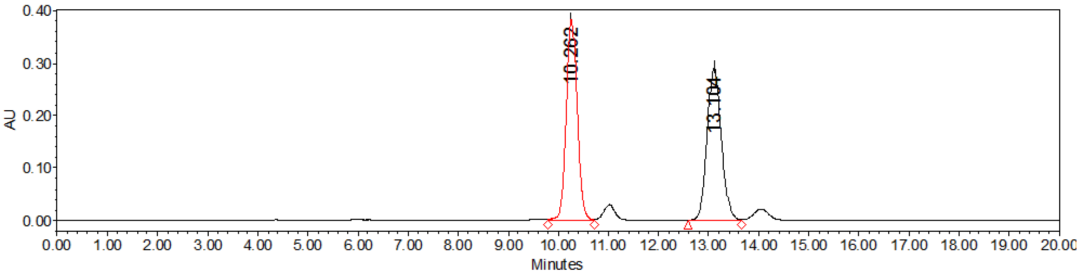

Peak	Retention Time	Area	% Area
1	10.262	5826861	50.25
2	13.104	5769016	49.75

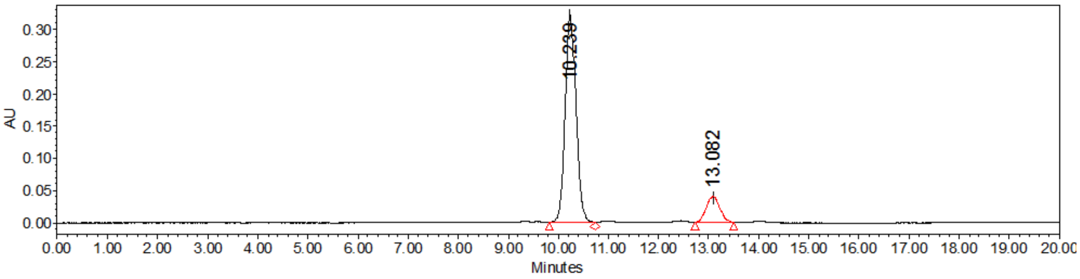

Peak	Retention Time	Area	% Area
1	10.239	4830303	86.83
2	13.082	732786	13.17

Isopropyl 2-(1-(2-methoxyphenyl)vinyl)-1-oxo-2,3-dihydro-1*H*-indene-2-carboxylate

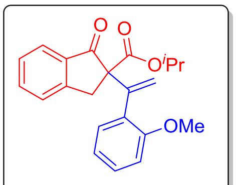

5bf

colorless oil; 90% yield; 94:6 e.r.; R_f = 0.30 (petroleum ether/ethyl acetate = 10/1).; $[\alpha]^{22}_D$ = -69.8 (*c* = 0.53, in DCM).
HPLC (Chiralcel **IG**, hexane/*i*-PrOH = 95/5, flow rate 1.0 mL/min, λ = 254 nm) t_r (major) = 19.96 min, t_r (minor) = 21.23 min.
IR (neat): ν (cm^{-1}) 2980, 2937, 2836, 2361, 1742, 1709, 1603, 1489, 1462, 1434, 1297, 1240, 1183, 1105, 1046, 1024, 908, 837 cm^{-1}.
^{1}H NMR (400 MHz, $CDCl_3$) δ 7.80 – 7.78 (m, 1H), 7.55 (td, *J* = 7.6, 1.2 Hz, 1H), 7.38 – 7.34 (m, 2H), 7.28 – 7.20 (m, 2H), 6.92 (td, *J* = 7.6, 1.2 Hz, 1H), 6.75 (dd, *J* = 8.4, 1.2 Hz, 1H), 5.66 (s, 1H), 5.41 (s, 1H), 4.97 (hept, *J* = 6.4 Hz, 1H), 3.90 (d, *J* = 17.6 Hz, 1H), 3.40 (s, 3H), 3.24 (d, *J* = 17.6 Hz, 1H), 1.19 (d, *J* = 6.4 Hz, 3H), 1.13 (d, *J* = 6.4 Hz, 3H).
^{13}C{^{1}H} NMR (101 MHz, $CDCl_3$) δ 199.5, 169.7, 156.2, 152.8, 145.1, 135.2, 134.8, 130.8, 130.7, 129.0, 127.3, 126.0, 124.7, 120.9, 120.3, 109.8, 69.3, 65.9, 54.3, 39.0, 21.5, 21.4.
HRMS (ESI-TOF) calcd for $C_{22}H_{22}NaO_4^+$ ([M]+Na$^+$) = 373.1410, found 373.1413.
Chiral HPLC spectrum **5bf**:

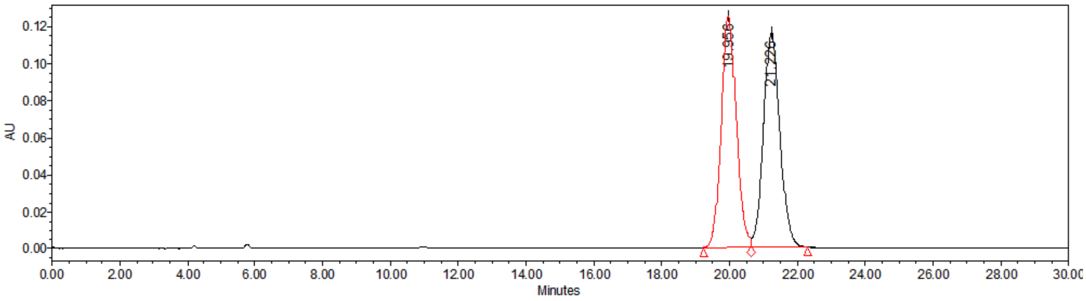

Peak	Retention Time	Area	% Area
1	19.956	3903472	49.82
2	21.226	3932084	50.18

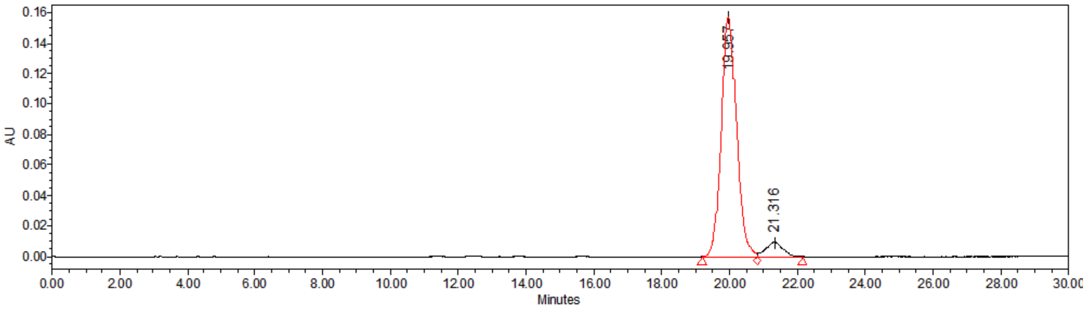

Peak	Retention Time	Area	% Area
1	19.957	4934994	94.04
2	21.316	312844	5.96

Isopropyl 1-oxo-2-(1-(*m*-tolyl)vinyl)-2,3-dihydro-1*H*-indene-2-carboxylate

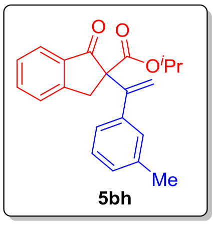

colorless oil; 71% yield; 91.5:8.5 e.r.; R_f = 0.45 (petroleum ether/ethyl acetate = 10/1); $[\alpha]^{23}_D$ = -264.4 (*c* = 0.17, in DCM).
HPLC (Chiralcel **IG**, hexane/*i*-PrOH = 95/5, flow rate 1.0 mL/min, λ = 254 nm) t_r (major) = 10.99 min, t_r (minor) = 12.26 min.
IR (neat): 2980, 2933, 1709, 1604, 1604, 1463, 1377, 1250, 1210, 1183, 1105, 1017, 921, 898, 796, 724 cm^{-1}.
^{1}H NMR (400 MHz, $CDCl_3$) 7.83 δ (d, *J* = 7.6 Hz, 1H), 7.62 (td, *J* = 7.2, 1.2 Hz, 1H), 7.50 – 7.39 (m, 2H), 721 – 7.08 (m, 4H), 5.46 (s, 1H), 5.29 (s, 1H), 4.93 (hept, *J* = 6.4 Hz, 1H), 4.21 (d, *J* = 17.2 Hz, 1H), 3.33 (d, *J* = 17.2 Hz, 1H), 2.33 (s, 3H), 1.17 (d, *J* = 6.4 Hz, 3H), 0.93 (d, *J* = 6.4 Hz, 3H).
^{13}C{^{1}H} NMR (101 MHz, $CDCl_3$) δ 200.2, 169.3, 152.7, 147.2, 140.7, 137.9, 135.6, 135.0, 128.4, 128.2, 127.8, 127.7, 126.4, 125.0, 123.8, 116.7, 69.8, 66.6, 40.2, 21.5, 21.5, 20.9.
HRMS (ESI-TOF) calcd for $C_{22}H_{22}NaO_3^+$ ([M]+H^+) = 357.1461, found 357.1462.

Chiral HPLC spectrum **5bh**:

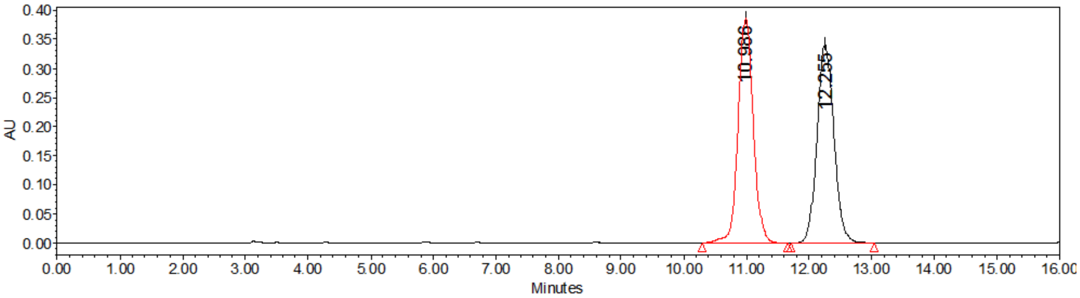

Peak	Retention Time	Area	% Area
1	10.986	6482529	50.38
2	12.255	6384919	49.62

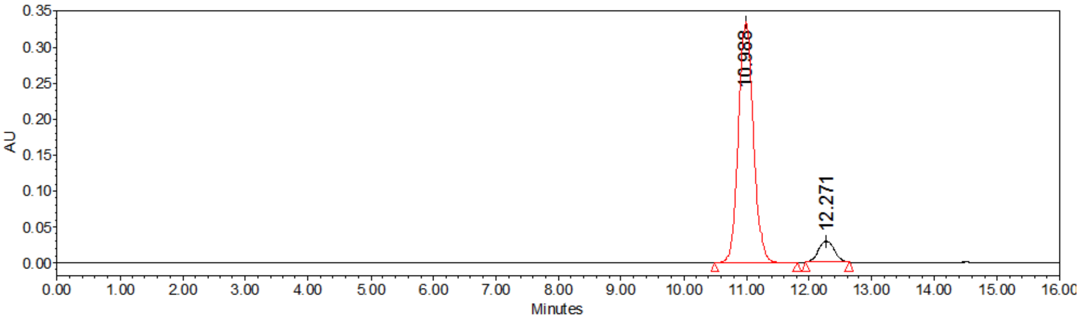

Peak	Retention Time	Area	% Area
1	10.988	5527177	91.42
2	12.271	519039	8.58

Isopropyl 2-(1-cyclopropylvinyl)-1-oxo-2,3-dihydro-1*H*-indene-2-carboxylate

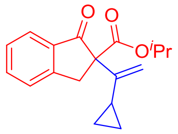

5bm

colorless oil; 82% yield; 95:5 e.r.; R_f = 0.50 (petroleum ether/ethyl acetate = 10/1); $[\alpha]^{23}_D$ = -168.3 (*c* = 0.16, in DCM).
HPLC (Chiralcel **IG**, hexane/*i*-PrOH = 95/5, flow rate 1.0 mL/min, λ = 254 nm) t_r (major) = 11.58 min, t_r (minor) = 12.29 min.
IR (neat): 2979, 2927, 2866, 2361, 1710, 1607, 1502, 1462, 1378, 1251, 1211, 1183, 1106, 924, 809 cm^{-1}.
^{1}H NMR (400 MHz, $CDCl_3$) δ 7.79 (d, *J* = 7.6 Hz, 1H), 7.61 (td, *J* = 7.6, 1.2 Hz, 1H), 7.47 – 7.46 (m, 1H), 7.43 – 7.36 (m, 1H), 5.07 (hept, *J* = 6.0 Hz, 1H), 4.87 (s, 1H), 4.77 (d, *J* = 0.8 Hz, 1H), 4.01 (d, *J* = 17.2 Hz, 1H), 3.51 (d, *J* = 17.6 Hz, 1H), 1.34 – 1.28 (m, 1H), 1.25 (dd, *J* = 8.8, 6.4 Hz, 6H), 0.76 – 0.68 (m, 2H), 0.63 – 0.55 (m, 1H), 0.54 – 0.46 (m, 1H).
^{13}C{^{1}H} NMR (101 MHz, $CDCl_3$) δ 200.0, 169.6, 152.2, 148.0, 135.3, 135.3, 127.7, 126.3, 124.8, 108.5, 69.6, 67.8, 38.2, 21.6, 21.5, 14.4, 8.1, 7.9.
HRMS (ESI-TOF) calcd for $C_{18}H_{20}KO_3^+$ ($[M+K]^+$) = 323.1044, found 323.1046.
Chiral HPLC spectrum **5bm**:

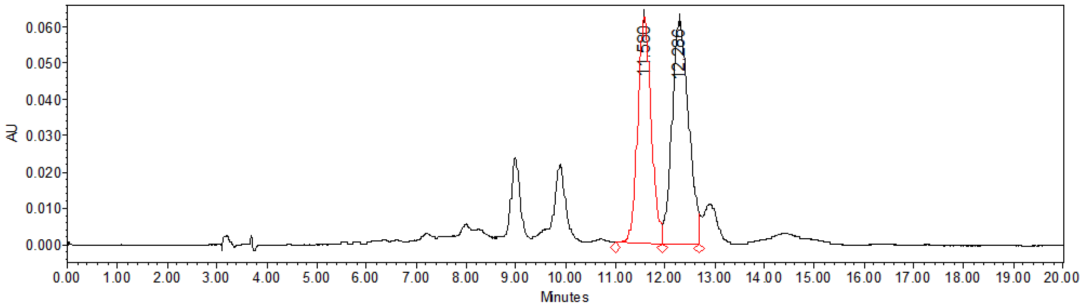

Peak	Retention Time	Area	% Area
1	11.580	1196856	49.41
2	12.286	1225390	50.59

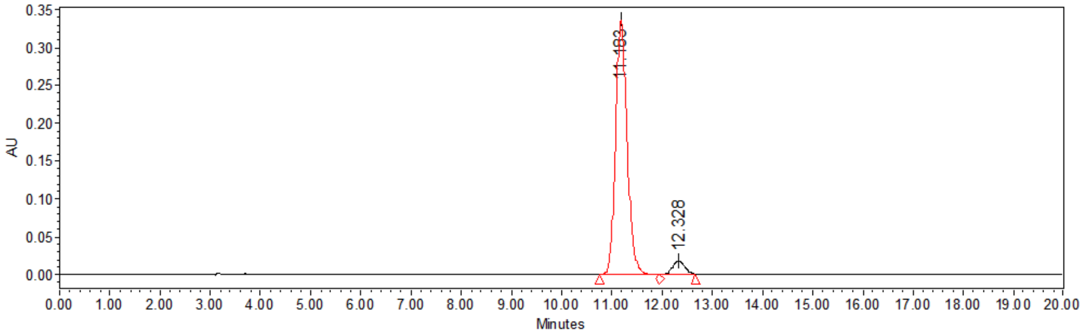

Peak	Retention Time	Area	% Area
1	11.183	5371677	94.91
2	12.328	287903	5.09

2-Isobutyryl-2-(1-phenylvinyl)-2,3-dihydro-1*H*-inden-1-one

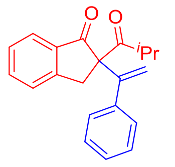

7aa

colorless oil; 96% yield; 95:5 e.r.; R_f = 0.45 (petroleum ether/ethyl acetate = 10/1); $[\alpha]^{22}_D$ = -318.1 (*c* = 0.50, in DCM).

HPLC (Chiralcel **IA**, hexane/*i*-PrOH = 98/2, flow rate 0.8 mL/min, λ = 254 nm) t_r (minor) = 8.45 min, t_r (major) = 11.11 min.

IR (neat): 2973, 2934, 2873, 1695, 1607, 1494, 1380, 1274, 1214, 1190, 1129, 1095, 1006, 983, 907, 768, 712 cm^{-1}.

^{1}H NMR (400 MHz, $CDCl_3$) δ 7.67 (d, *J* = 7.6 Hz, 1H), 7.52 (t, *J* = 7.6 Hz, 1H), 7.36 (d, *J* = 8.0 Hz, 1H), 7.28 (t, *J* = 7.2 Hz, 1H), 7.25 – 7.19 (m, 3H), 7.18 – 7.15 (m, 2H), 5.62 (s, 1H), 5.57 (s, 1H), 4.34 (d, *J* = 17.2 Hz, 1H), 3.53 (hept, *J* = 6.4 Hz, 1H), 3.00 (d, *J* = 17.2 Hz, 1H), 0.88 (d, *J* = 6.4 Hz, 3H), 0.62 (d, *J* = 6.8 Hz, 3H).

^{13}C{^{1}H} NMR (101 MHz, $CDCl_3$) δ 210.1, 200.6, 152.9, 146.6, 140.7, 135.6, 134.9, 128.5, 128.0, 127.7, 127.1, 126.3, 124.6, 118.0, 74.4, 37.7, 36.4, 21.5, 20.4.

HRMS (ESI-TOF) calcd for $C_{21}H_{21}O_2^+$ ($[M]+H^+$) = 305.1536, found 305.1537.

Chiral HPLC spectrum **7aa**:

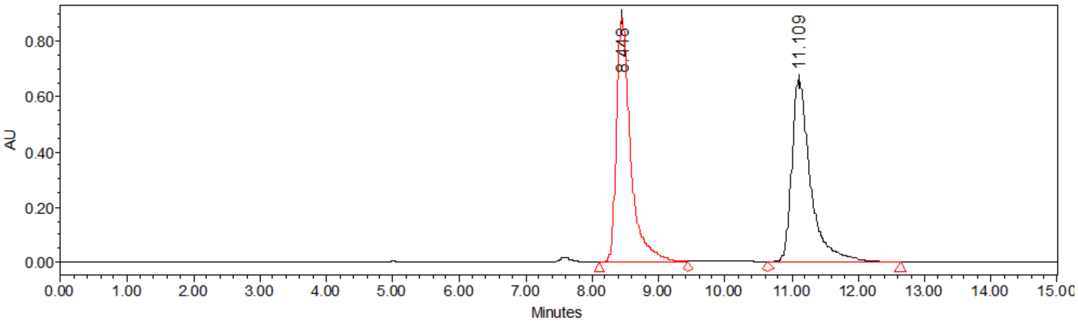

Peak	Retention Time	Area	% Area
1	8.45	13174249	50.15
2	11.11	13094160	49.85

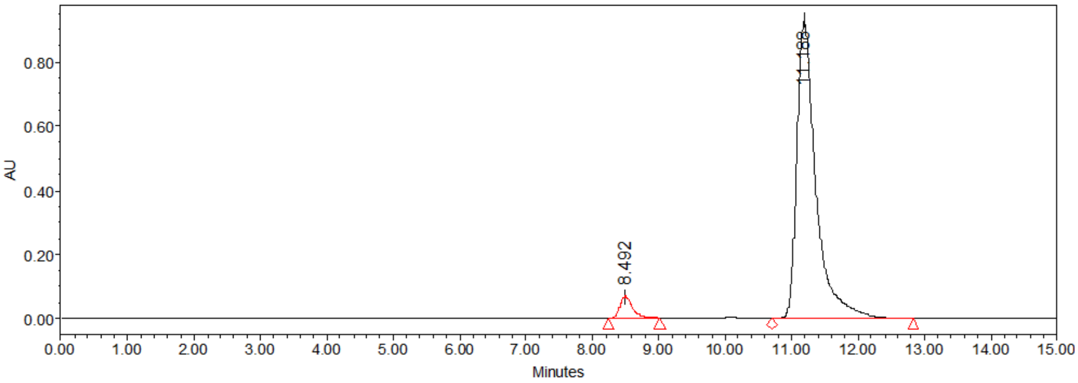

Peak	Retention Time	Area	% Area
1	8.49	936643	4.79
2	11.19	18617389	95.21

5-Bromo-2-isobutyryl-2-(1-phenylvinyl)-2,3-dihydro-1*H*-inden-1-one

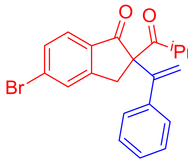

7ba

colorless oil; 95% yield; 91:9 e.r.; R_f = 0.45 (petroleum ether/ethyl acetate = 10/1); $[\alpha]^{23}_{D}$ = -241.7 (c = 0.68, in DCM).
HPLC (Chiralcel **IA**, hexane/*i*-PrOH = 98/2, flow rate 1.0 mL/min, λ = 254 nm) t_r (minor) = 7.19 min, t_r (major) = 8.20 min.
IR (neat): 2972, 2932, 2873, 1699, 1650, 1596, 1464, 1418, 1311, 1262, 1207, 983, 903, 777, 705 cm^{-1}.
^{1}H NMR (400 MHz, $CDCl_3$) δ 7.60 (d, *J* = 8.4 Hz, 2H), 7.51 (d, *J* = 8.4 Hz, 1H), 7.33 – 7.28 (m, 3H), 7.22 – 7.20 (m, 2H), 5.67 (d, *J* = 13.2 Hz, 2H), 4.39 (d, *J* = 17.2 Hz, 1H), 3.60 (hept, *J* = 6.8 Hz, 1H), 3.05 (d, *J* = 17.2 Hz, 1H), 0.95 (d, *J* = 6.4 Hz, 3H), 0.72 (d, *J* = 6.8 Hz, 3H).
^{13}C{^{1}H} NMR (101 MHz, $CDCl_3$) δ 209.7, 199.4, 154.4, 146.3, 140.5, 133.8, 131.4, 131.2, 129.6, 128.6, 128.1, 127.1, 125.8, 118.2, 74.4, 37.3, 36.4, 21.5, 20.4.
HRMS (ESI-TOF) calcd for $C_{21}H_{19}Br^{78.9183}NaO_2^+$ ([M]+Na$^+$) = 405.0461, found 405.0468, and calcd for $C_{21}H_{19}Br^{80.9163}NaO_2^+$ ([M]+Na$^+$) = 407.0440, found 407.0446.

Chiral HPLC spectrum **7ba**:

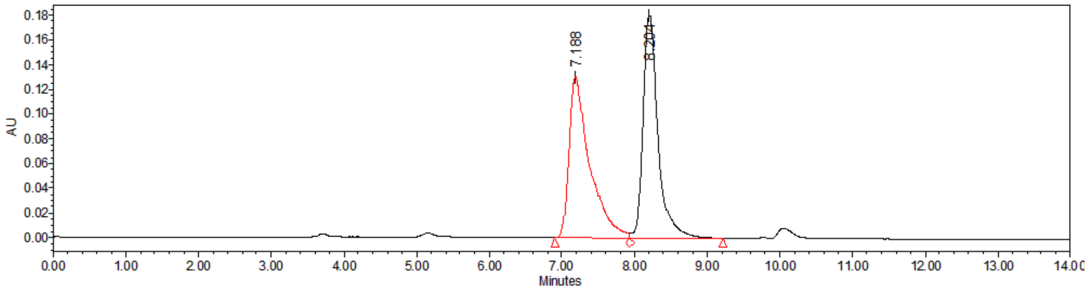

Peak	Retention Time	Area	% Area
1	7.188	2603857	50.22
2	8.204	2580848	49.78

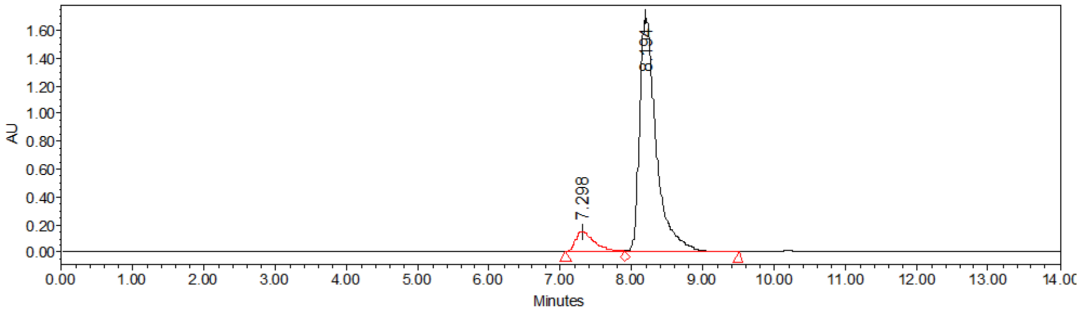

Peak	Retention Time	Area	% Area
1	7.298	2793877	9.14
2	8.194	27786197	90.86

2-Isobutyryl-5-methoxy-2-(1-phenylvinyl)-2,3-dihydro-1*H*-inden-1-one

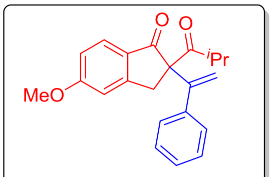

7ca

colorless oil; 89% yield; 96.5:3.5 e.r.; R_f = 0.40 (petroleum ether/ethyl acetate = 10/1); $[\alpha]^{23}_D$ = -312.7 (c = 0.29, in DCM).
HPLC (Chiralcel **IA**, hexane/*i*-PrOH = 95/5, flow rate 1.0 mL/min, λ = 254 nm) t_r (minor) = 9.22 min, t_r (major) = 10.38 min.
IR (neat): 2970, 2934, 2360, 1691, 1595, 1491, 1464, 1445, 1259, 1091, 1024, 982, 842, 804, 778, 710, 606, 546 cm^{-1}.
^{1}H NMR (400 MHz, $CDCl_3$) δ 7.61 (d, J = 8.4 Hz, 1H), 7.26 – 7.16 (m, 5H), 6.84 – 6.79 (m, 2H), 5.60 (d, J = 24.4 Hz, 2H), 4.30 (d, J = 17.2 Hz, 1H), 3.80 (s, 3H), 3.53 (hept, J = 6.4 Hz, 1H), 2.95 (d, J = 17.2 Hz, 1H), 0.89 (d, J = 6.6 Hz, 3H), 0.60 (d, J = 6.4 Hz, 3H).
^{13}C{^{1}H} NMR (101 MHz, $CDCl_3$) δ 209.7 , 197.4, 165.0, 154.9, 145.8, 139.8, 127.5, 127.1, 126.9, 125.3, 116.9, 115.0, 108.1, 73.4, 54.7, 36.7, 35.4, 20.5, 19.3.
HRMS (ESI-TOF) calcd for $C_{22}H_{22}NaO_3^+$ ([M]+Na^+) = 357.1461, found 357.1464.
Chiral HPLC spectrum **7ca**:

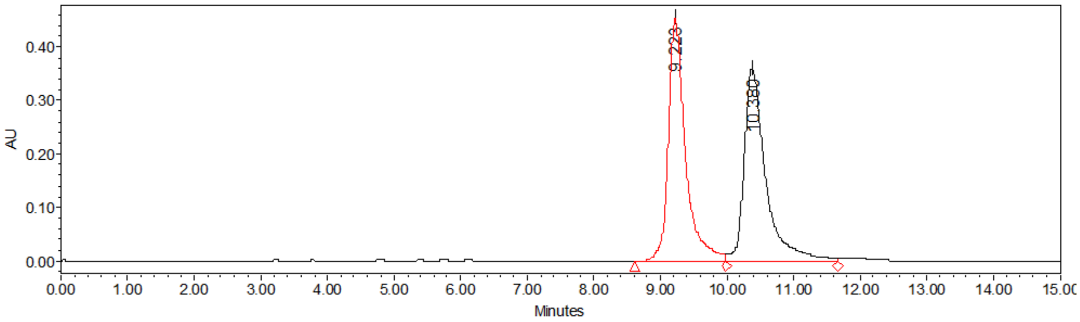

Peak	Retention Time	Area	% Area
1	9.223	8094778	50.07
2	10.380	8071947	49.93

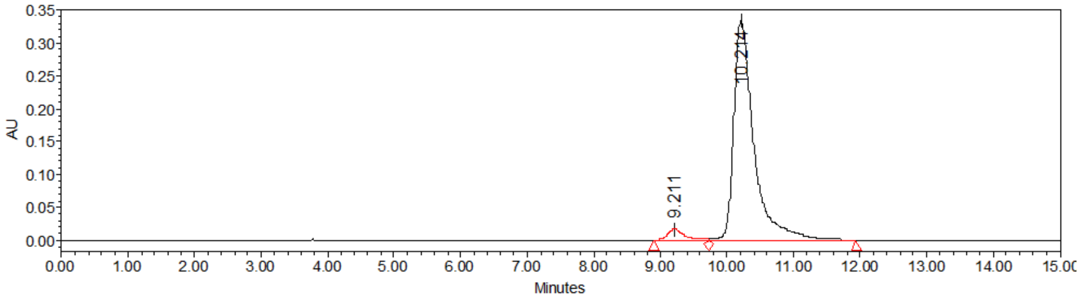

Peak	Retention Time	Area	% Area
1	9.211	256305	3.54
2	10.214	6975151	96.46

6-Chloro-2-isobutyryl-2-(1-phenylvinyl)-2,3-dihydro-1*H*-inden-1-one

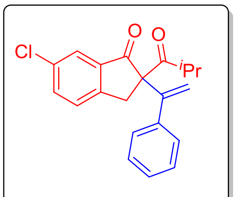

7da

colorless oil; 89% yield; 94:6 e.r.; R_f = 0.55 (petroleum ether/ethyl acetate = 10/1); $[\alpha]^{23}_D$ = -249.6 (*c* = 0.58, in DCM).
HPLC (Chiralcel **IA**, hexane/*i*-PrOH = 98/2, flow rate 1.0 mL/min, λ = 254 nm) t_r (major) = 5.44 min, t_r (minor) = 6.29 min.
IR (neat): 2974, 2933, 2874, 1699, 1607, 1470, 1422, 1249, 1204, 1184, 983, 923, 822, 777, 717 cm^{-1}.
^{1}H NMR (400 MHz, $CDCl_3$) δ 7.63 (d, *J* = 2.0 Hz, 1H), 7.48 (dd, *J* = 8.4, 2.0 Hz, 1H), 7.30 (d, *J* = 8.0 Hz, 1H), 7.25 – 7.21 (m, 3H), 7.15 – 7.12 (m, 2H), 5.60 (d, *J* = 13.2 Hz, 2H), 4.29 (d, *J* = 17.6 Hz, 1H), 3.52 (hept, *J* = 6.8 Hz, 1H), 2.96 (d, *J* = 17.2 Hz, 1H), 0.88 (d, *J* = 6.8 Hz, 3H), 0.65 (d, *J* = 6.8 Hz, 3H).
^{13}C{^{1}H} NMR (101 MHz, $CDCl_3$) δ 208.5, 198.3, 149.9, 145.2, 139.4, 135.4, 134.6, 133.0, 127.6, 127.1, 126.5, 126.0, 123.3, 117.1, 73.9, 36.2, 35.3, 20.4, 19.4.
HRMS (ESI-TOF) calcd for $C_{21}H_{19}Cl^{34.9689}NaO_2^+$ ($[M]+Na^+$) = 361.0966, found 361.0969, and calcd for $C_{21}H_{19}Cl^{36.9659}NaO_2^+$ ($[M]+Na^+$) = 363.0936, found 363.0935.
Chiral HPLC spectrum **7da**:

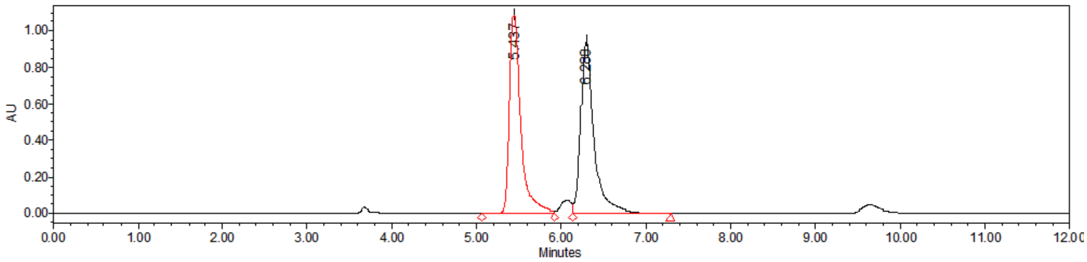

Peak	Retention Time	Area	% Area
1	5.437	9996373	49.35
2	6.288	10260514	50.65

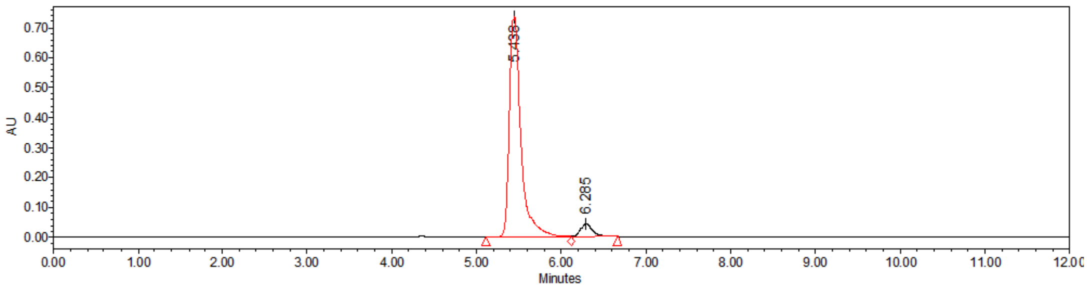

Peak	Retention Time	Area	% Area
1	5.438	6849351	93.88
2	6.285	446302	6.12

2-Isobutyryl-6-methyl-2-(1-phenylvinyl)-2,3-dihydro-1*H*-inden-1-one

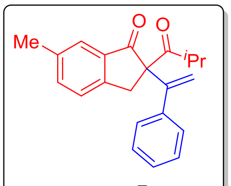

7ea

colorless oil; 76% yield; 96.5:3.5 e.r.; R_f = 0.45 (petroleum ether/ethyl acetate = 10/1); $[\alpha]^{22}_{D}$ = -268.8 (*c* = 0.44, in DCM).
HPLC (Chiralcel **IA**, hexane/*i*-PrOH = 98/2, flow rate 1.0 mL/min, λ = 254 nm) t_r (minor) = 7.34 min, t_r (major) = 8.86 min.
IR (neat): 2972, 2928, 2872, 1695, 1616, 1586, 1493, 1277, 1219, 1095, 1025, 983, 818, 777, 702 cm^{-1}.
^{1}H NMR (400 MHz, $CDCl_3$) δ 7.48 (s, 1H), 7.36 – 7.34 (m, 1H), 7.26 – 7.15 (m, 6H), 5.58 (d, *J* = 12.4 Hz, 2H), 4.28 (d, *J* = 17.2 Hz, 1H), 3.51 (hept, *J* = 6.4 Hz, 1H), 2.96 (d, *J* = 16.8 Hz, 1H), 2.32 (s, 3H), 0.88 (d, *J* = 6.4 Hz, 3H), 0.62 (d, *J* = 6.4 Hz, 3H).
^{13}C{^{1}H} NMR (101 MHz, $CDCl_3$) δ 210.2, 200.7, 150.3, 146.9, 140.8, 137.7, 137.0, 135.2, 128.5, 128.0, 127.1, 126.0, 124.5, 117.9, 74.8, 37.4, 36.5, 21.6, 21.1, 20.4.
HRMS (ESI-TOF) calcd for $C_{22}H_{22}NaO_2^+$ ($[M]+Na^+$) = 341.1512, found 341.1517.
Chiral HPLC spectrum **7ea**:

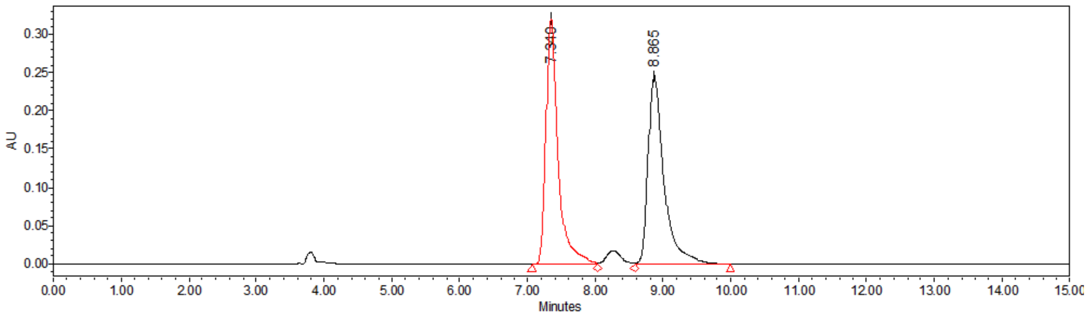

Peak	Retention Time	Area	% Area
1	7.340	4004758	49.87
2	8.865	4025284	50.13

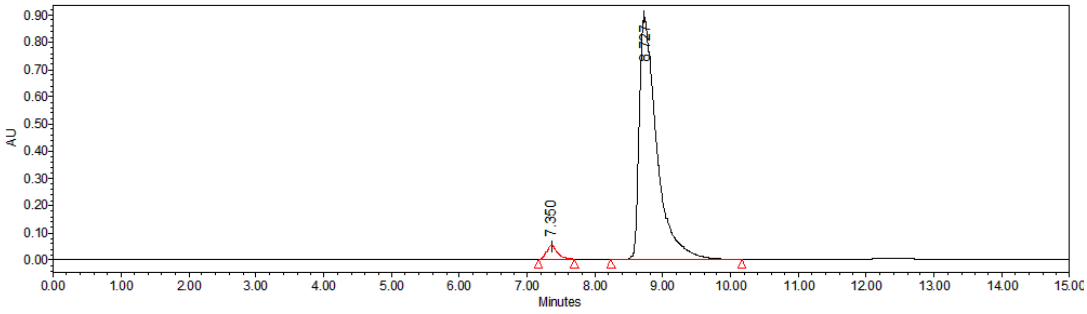

Peak	Retention Time	Area	% Area
1	7.350	585568	3.49
2	8.727	16177595	96.51

2-Isobutyryl-6-methoxy-2-(1-phenylvinyl)-2,3-dihydro-1*H*-inden-1-one

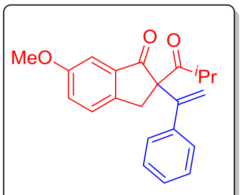

7fa

colorless oil; 87% yield; 96.5:3.5 e.r.; R_f = 0.40 (petroleum ether/ethyl acetate = 10/1); $[\alpha]^{22}_D$ = -301.6 (c = 0.55, in DCM).
HPLC (Chiralcel **IA**, hexane/*i*-PrOH = 95/5, flow rate 1.0 mL/min, λ = 254 nm) t_r (major) = 7.00 min, t_r (minor) = 7.92 min.
IR (neat): 2930, 2873, 1695, 1652, 1492, 1441, 1274, 1224, 1196, 1093, 1025, 983, 774, 701 cm^{-1}.
^{1}H NMR (400 MHz, $CDCl_3$) δ 7.34 – 7.17 (m, 8H), 5.65 (d, J = 7.2 Hz, 2H), 4.32 (d, J = 16.8 Hz, 1H), 3.83 (s, 3H), 3.58 (hept, J = 6.4 Hz, 1H), 3.02 (d, J = 16.8 Hz, 1H), 0.96 (d, J = 6.4 Hz, 3H), 0.70 (d, J = 6.8 Hz, 3H).
^{13}C{^{1}H} NMR (101 MHz, $CDCl_3$) δ 210.1, 200.6, 159.7, 146.8, 145.9, 140.7, 136.1, 128.6, 128.0, 127.1, 127.1, 125.2, 117.9, 105.6, 75.3, 55.6, 37.1, 36.5, 21.6, 20.3.
HRMS (ESI-TOF) calcd for $C_{22}H_{22}NaO_3^+$ ($[M]+Na^+$) = 357.1461, found 357.1463.
Chiral HPLC spectrum **7fa**:

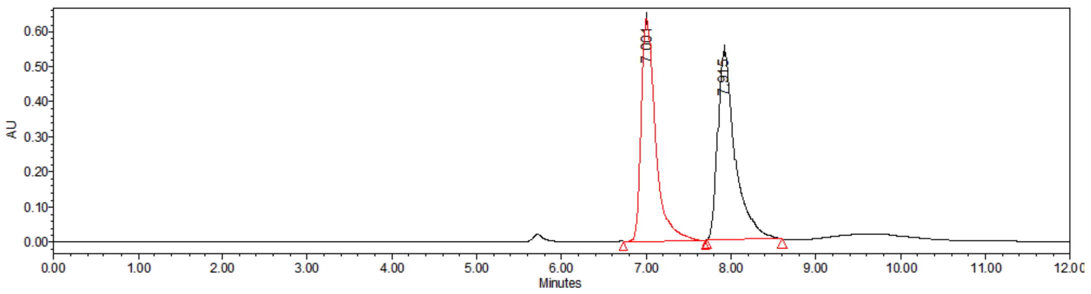

Peak	Retention Time	Area	% Area
1	7.001	7480674	49.21
2	7.915	7721241	50.79

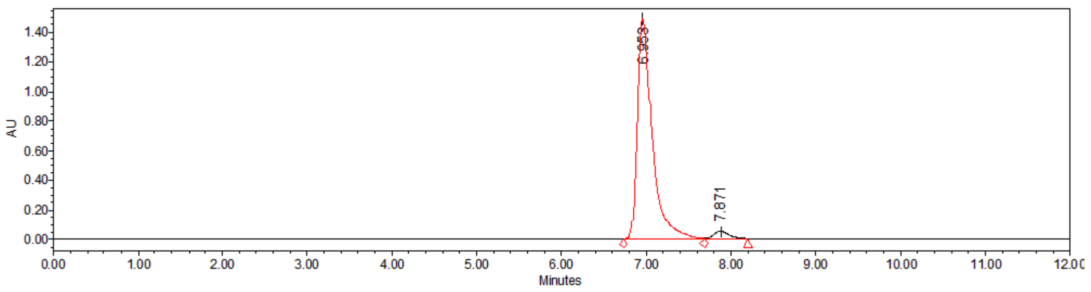

Peak	Retention Time	Area	% Area
1	6.953	18469618	96.54
2	7.871	661858	3.46

2-Isobutyryl-5,6-dimethoxy-2-(1-phenylvinyl)-2,3-dihydro-1*H*-inden-1-one

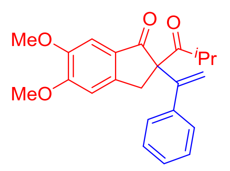

7ga

colorless oil; 74% yield; 95:5 e.r.; R_f = 0.25 (petroleum ether/ethyl acetate = 8/1); $[\alpha]^{26}_D$ = -321.7 (*c* = 0.50, in DCM).
HPLC (Chiralcel **IA**, hexane/*i*-PrOH = 90/10, flow rate 1.0 mL/min, λ = 254 nm) t_r (major) = 8.88 min, t_r (minor) = 11.83 min.
IR (neat): 2972, 2873, 1688, 1592, 1500, 1464, 1309, 1266, 1222, 1097, 1021, 985, 908, 864, 779, 712 cm^{-1}.
^{1}H NMR (400 MHz, $CDCl_3$) δ 7.33 – 7.24 (m, 5H), 7.16 (s, 1H), 6.87 (s, 1H), 5.66 (d, *J* = 10.0 Hz, 2H), 4.34 (d, *J* = 17.2 Hz, 1H), 3.96 (s, 3H), 3.91 (s, 3H), 3.58 (hept, *J* = 6.4 Hz, 1H), 3.00 (d, *J* = 16.8 Hz, 1H), 0.97 (d, *J* = 6.4 Hz, 3H), 0.66 (d, *J* = 6.4 Hz, 3H).
^{13}C{^{1}H} NMR (101 MHz, $CDCl_3$) δ 210.8, 198.9, 156.3, 149.8, 148.5, 147.0, 140.8, 128.5, 127.9, 127.6, 127.0, 117.8, 107.1, 104.8, 74.6, 56.3, 56.1, 37.6, 36.5, 21.6, 20.3.
HRMS (ESI-TOF) calcd for $C_{23}H_{24}NaO_4^+$ ([M]+Na^+) = 387.1567, found 387.1568.
Chiral HPLC spectrum **7ga**:

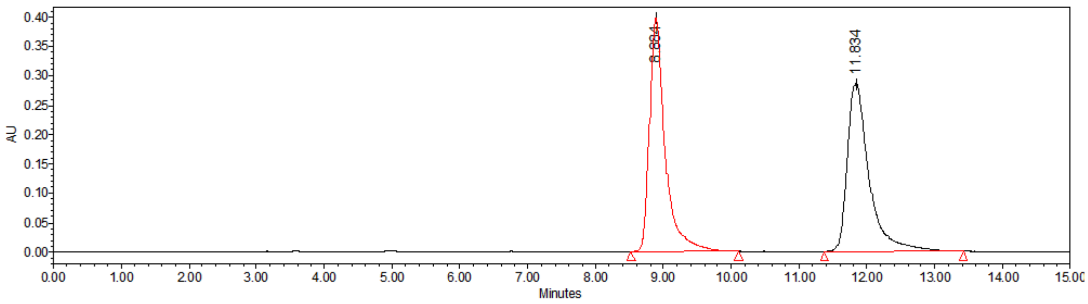

Peak	Retention Time	Area	% Area
1	8.884	6436643	50.03
2	11.834	6428590	49.97

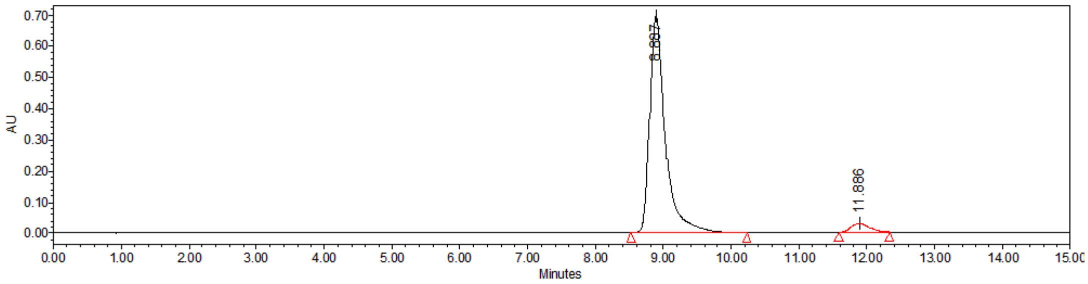

Peak	Retention Time	Area	% Area
1	8.887	11288526	95.15
2	11.886	575192	4.85

2-(1-(2-Fluorophenyl)vinyl)-2-isobutyryl-2,3-dihydro-1*H*-inden-1-one

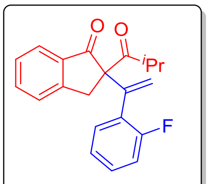

7ab

colorless oil; 84% yield; 93:7 e.r.; R_f = 0.45 (petroleum ether/ethyl acetate = 10/1); $[\alpha]^{23}_D$ = -21.2 (*c* = 0.51, in DCM).
HPLC (Chiralcel **IA**, hexane/*i*-PrOH = 98/2, flow rate 1.0 mL/min, λ = 254 nm) t_r (minor) = 8.17 min, t_r (major) = 9.21 min.
IR (neat): 2969, 2932, 2873, 1700, 1607, 1488, 1450, 1272, 1213, 1130, 1087, 985, 906, 761 cm^{-1}.
^{1}H NMR (400 MHz, $CDCl_3$) δ 7.62 (d, *J* = 7.6 Hz, 1H), 7.51 (td, *J* = 7.6, 1.2 Hz, 1H), 7.37 (dt, *J* = 7.6, 1.2 Hz, 1H), 7.29 – 7.24 (m, 1H), 7.22 – 7.12 (m, 2H), 7.05 (td, *J* = 7.2, 1.2 Hz, 1H), 6.93 – 6.88 (m, 1H), 5.59 (s, 1H), 5.47 (s, 1H), 4.21 (d, *J* = 16.8 Hz, 1H), 3.42 (hept, *J* = 6.8 Hz, 1H), 3.11 (d, *J* = 16.8 Hz, 1H), 0.94 (d, *J* = 6.8 Hz, 3H), 0.83 (d, *J* = 6.8 Hz, 3H).
^{13}C{^{1}H} NMR (101 MHz, $CDCl_3$) δ 207.4, 199.7, 159.4 (d, J_{C-F} = 244.8), 152.8, 142.2, 135.3, 135.1, 130.5 (d, J_{C-F} = 4.1), 129.72 (d, J_{C-F} = 8.5), 128.6 (d, J_{C-F} = 15.2), 127.6, 126.1, 124.5, 124.3 (d, J_{C-F} = 3.5), 122.0, 155.6, (d, J_{C-F} = 22.3), 74.4, 37.4 (d, J_{C-F} = 2.8), 36.7, 21.3, 21.1.
^{19}F{^{1}H} NMR (376 MHz, $CDCl_3$) δ –112.0 (s, 1F).
HRMS (ESI-TOF) calcd for $C_{21}H_{19}FNaO_2^+$ ([M]+Na^+) = 345.1261, found 345.1265.
Chiral HPLC spectrum **7ab**:

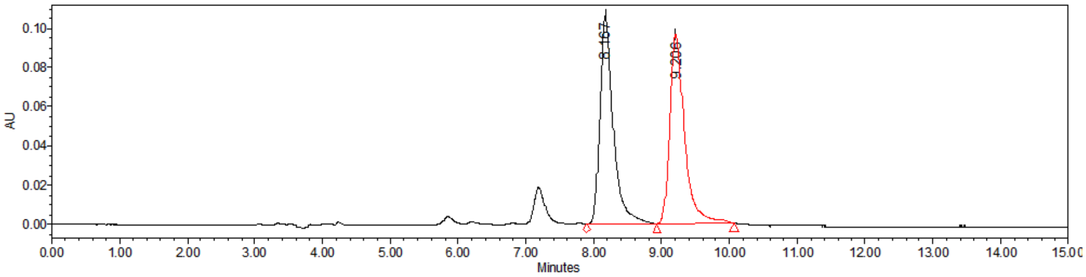

Peak	Retention Time	Area	% Area
1	8.167	1471037	49.94
2	9.206	1474500	50.06

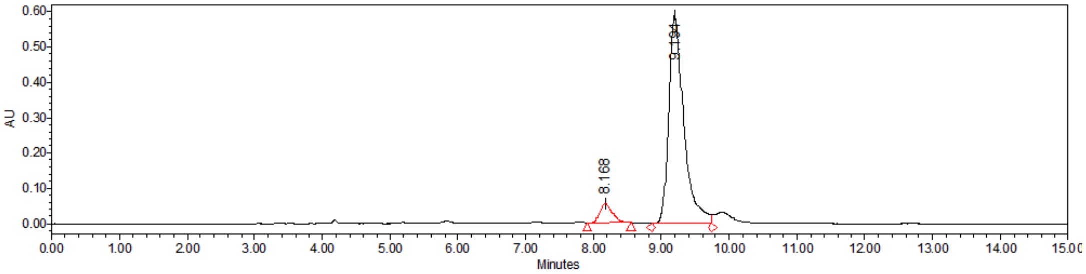

Peak	Retention Time	Area	% Area
1	8.168	688997	6.99
2	9.194	9165346	93.01

2-(1-(4-Fluorophenyl)vinyl)-2-isobutyryl-2,3-dihydro-1*H*-inden-1-one

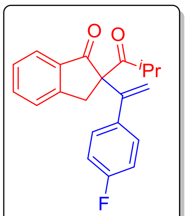

7ae

colorless oil; 73% yield; 96:4 e.r.; R_f = 0.45 (petroleum ether/ethyl acetate = 10/1); $[\alpha]^{22}_D$ = -258.1 (*c* = 0.47, in DCM).
HPLC (Chiralcel **IA**, hexane/*i*-PrOH = 98/2, flow rate 1.0 mL/min, λ = 254 nm) t_r (minor) = 6.79 min, t_r (major) = 10.61 min.
IR (neat): 2974, 2935, 2874, 1696, 1604, 1508, 1465, 1275, 1232, 1095, 1009, 983, 906, 843, 714 cm^{-1}.
^{1}**H NMR** (400 MHz, $CDCl_3$) δ 7.68 (d, *J* = 7.6 Hz, 1H), 7.56 – 7.52 (m, 1H), 7.37 (d, *J* = 7.6 Hz, 1H), 7.30 (t, *J* = 7.6 Hz, 1H), 7.16 – 7.12 (m, 2H), 6.95 – 6.90 (m, 2H), 5.57 (d, *J* = 36.0 Hz, 2H), 4.32 (d, *J* = 17.2 Hz, 1H), 3.52 (hept, *J* = 6.4 Hz, 1H), 2.99 (d, *J* = 16.8 Hz, 1H), 0.88 (d, *J* = 6.8 Hz, 3H), 0.67 (d, *J* = 6.8 Hz, 3H).
^{13}**C{^{1}H} NMR** (101 MHz, $CDCl_3$) δ 210.0, 200.4, 162.5 (d, $J_{C\text{-}F}$ =246.8), 152.7, 145.6, 136.8 (d, $J_{C\text{-}F}$ =3.6), 135.7, 135.0, 128.8 (d, $J_{C\text{-}F}$ =9.0), 127.8, 126.3, 124.7, 118.1, 115.4 (d, $J_{C\text{-}F}$ =21.1), 74.3, 37.5, 36.4, 21.4, 20.4.
^{19}**F{^{1}H} NMR** (376 MHz, $CDCl_3$) δ –113.9 (s, 1F).
HRMS (ESI-TOF) calcd for $C_{21}H_{19}FNaO_2^+$ ($[M]+H^+$) = 345.1261, found 345.1264.
Chiral HPLC spectrum **7ae**:

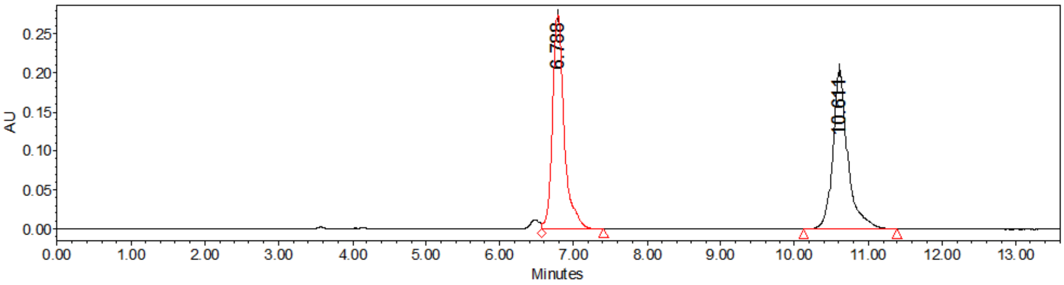

Peak	Retention Time	Area	% Area
1	6.788	2962752	50.19
2	10.611	2940270	49.81

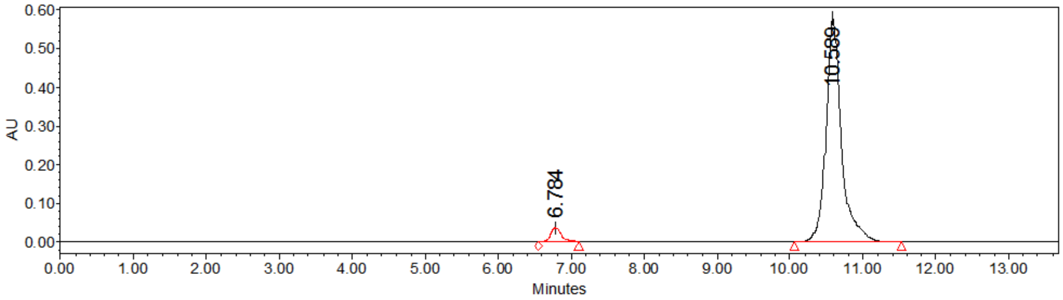

Peak	Retention Time	Area	% Area
1	6.784	363662	4.11
2	10.589	8478101	95.89

2-Isobutyryl-2-(1-(2-methoxyphenyl)vinyl)-2,3-dihydro-1*H*-inden-1-one

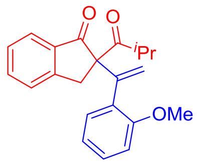

7af

colorless oil; 99% yield; 93.5:6.5 e.r.; R_f = 0.35 (petroleum ether/ethyl acetate = 10/1); $[\alpha]^{22}_D$ = +110.7 (*c* = 0.66, in DCM).
HPLC (Chiralcel **IA**, hexane/*i*-PrOH = 95/5, flow rate 1.0 mL/min, λ = 254 nm) t_r (minor) = 5.75 min, t_r (major) = 6.43 min.
IR (neat): 2971, 2935, 1698, 1600, 1487, 1462, 1435, 1294, 1241, 1087, 1021, 988, 906, 755 cm^{-1}.
^{1}H NMR (400 MHz, $CDCl_3$) δ 7.66 (d, *J* = 7.6 Hz, 1H), 7.57 (td, *J* = 7.6, 1.2 Hz, 1H), 7.49 – 7.46 (m, 1H), 7.34 – 7.28 (m, 3H), 7.03 (td, *J* = 7.6, 0.8 Hz, 1H), 6.72 (dd, *J* = 8.4, 1.2 Hz, 1H), 5.42 (s, 1H), 5.27 (s, 1H), 4.32 (d, *J* = 16.8 Hz, 1H), 3.41 (hept, *J* = 6.4 Hz, 1H), 3.29 (d, *J* = 16.8 Hz, 1H), 3.09 (s, 3H), 1.17 (d, *J* = 6.8 Hz, 3H), 0.86 (d, *J* = 6.8 Hz, 3H).
^{13}C{^{1}H} NMR (101 MHz, $CDCl_3$) δ 207.3, 196.4, 155.9, 152.8, 146.9, 135.8, 134.6, 130.3, 130.1 , 129.4, 127.1, 125.9, 123.9, 121.1, 120.0, 109.7, 74.3, 53.8, 37.8, 37.1, 22.0, 21.2.
HRMS (ESI-TOF) calcd for $C_{22}H_{22}NaO_3^+$ ($[M]+Na^+$) = 357.1461, found357.1463.
Chiral HPLC spectrum **7af**:

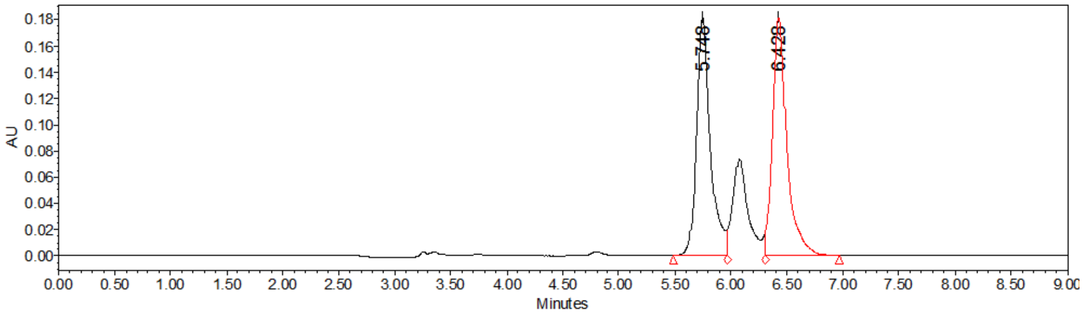

Peak	Retention Time	Area	% Area
1	5.748	1584799	49.53
2	6.428	1614713	50.47

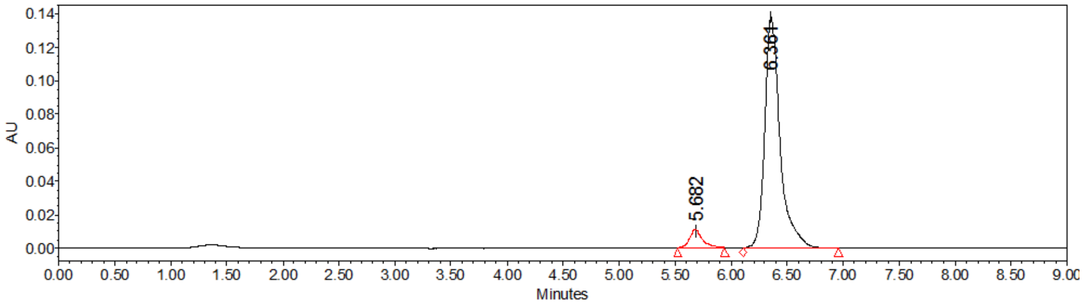

Peak	Retention Time	Area	% Area
1	5.682	90194	6.47
2	6.361	1303587	93.53

2-(1-(4-Chlorophenyl)vinyl)-2-isobutyryl-2,3-dihydro-1*H*-inden-1-one

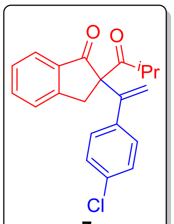

7ag

yellow oil; 62% yield; 87.5:12.5 e.r.; R_f = 0.50 (petroleum ether/ethyl acetate = 10/1); $[\alpha]^{23}_{D}$ = -190.8 (*c* = 0.37, in DCM).

HPLC (Chiralcel **IA**, hexane/*i*-PrOH = 98/2, flow rate 1.0 mL/min, λ = 254 nm) t_r (minor) = 6.87 min, t_r (major) = 9.97 min.

IR (neat): 2964, 2930, 2872, 2361, 1698, 1608, 1491, 1464, 1273, 1126, 1096, 1011, 983, 906, 838, 739 cm^{-1}.

^{1}H NMR (400 MHz, $CDCl_3$) δ 7.75 (d, *J* = 7.6 Hz, 1H), 7.61 (td, *J* = 7.6, 1.2 Hz, 1H), 7.44 (d, *J* = 7.6 Hz, 1H), 7.38 (t, *J* = 7.6 Hz, 1H), 7.29 – 7.26 (m, 2H), 7.18 – 7.16 (m, 2H), 5.74 (s, 1H), 5.63 (s, 1H), 4.38 (d, *J* = 17.6 Hz, 1H), 3.61 (hept, *J* = 6.8 Hz, 1H), 3.04 (d, *J* = 17.2 Hz, 1H), 0.96 (d, *J* = 6.8 Hz, 3H), 0.77 (d, *J* = 6.8 Hz, 3H).

^{13}C{^{1}H} NMR (101 MHz, $CDCl_3$) δ 209.8, 200.3, 152.7, 145.4, 139.2, 135.7, 134.9, 134.0, 128.7, 128.5, 127.8, 126.3, 124.7, 118.6, 74.1, 37.4, 36.3, 21.4, 20.5

HRMS (ESI-TOF) calcd for $C_{21}H_{19}Cl^{34.9689}NaO_2^+$ ([M]+Na$^+$) = 361.0966, found 361.0968, and calcd for $C_{21}H_{19}Cl^{36.9659}NaO_2^+$ ([M]+Na$^+$) = 363.0936, found 361.0934.
Chiral HPLC spectrum **7ag**:

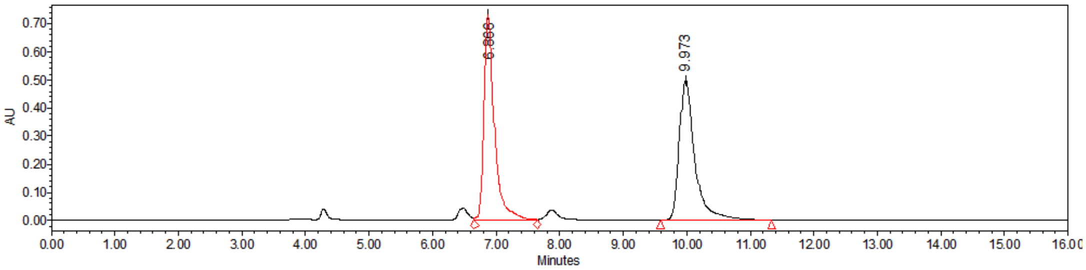

Peak	Retention Time	Area	% Area
1	6.866	8590008	50.14
2	9.973	8543667	49.86

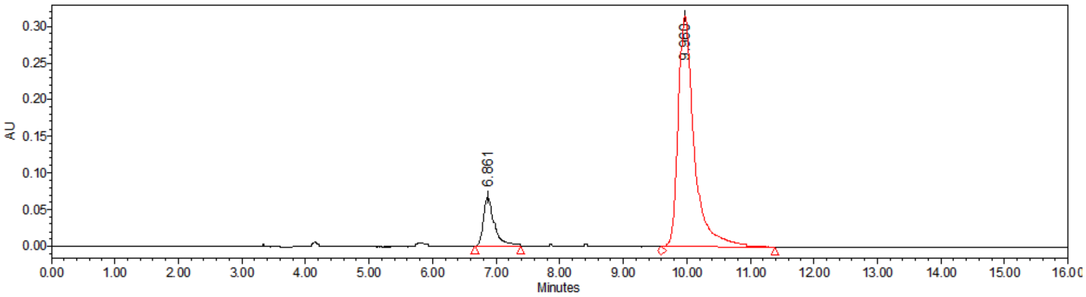

Peak	Retention Time	Area	% Area
1	6.861	784899	12.59
2	9.960	5447745	87.41

2-Isobutyryl-2-(1-(m-tolyl)vinyl)-2,3-dihydro-1*H*-inden-1-one

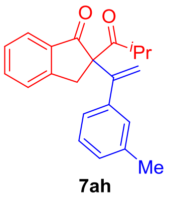

7ah

colorless oil; 99% yield; 94.5:5.5 e.r.; R_f = 0.50 (petroleum ether/ethyl acetate = 10/1); $[\alpha]^{23}_D$ = -273.1 (c = 0.62, in DCM).
HPLC (Chiralcel **IA**, hexane/*i*-PrOH = 98/2, flow rate 1.0 mL/min, λ = 254 nm) t_r (minor) = 6.36 min, t_r (major) = 7.37 min.
IR (neat): 2973, 2929, 2873, 1696, 1604, 1464, 1271, 1095, 1008, 983, 913, 886, 790, 726 cm^{-1}.
^{1}H NMR (400 MHz, $CDCl_3$) δ 7.76 (d, J = 7.6 Hz, 1H), 7.61 (td, J = 7.6, 1.2 Hz, 1H), 7.45 (d, J = 7.6 Hz, 1H), 7.38 (t, J = 7.6 Hz, 1H), 7.20 (t, J = 7.6 Hz, 1H), 7.12 – 7.08 (m, 2H), 7.02 (dt, J = 8.0, 1.6 Hz, 1H), 5.69 (s, 1H), 5.64 (s, 1H), 4.41 (d, J = 17.2 Hz, 1H), 3.62 (hept, J = 6.8 Hz, 1H), 3.09 (d, J = 17.2 Hz, 1H), 2.34 (s, 3H), 0.97 (d, J = 6.8 Hz, 3H), 0.73 (d, J = 6.8 Hz, 3H).
^{13}C{^{1}H} NMR (101 MHz, $CDCl_3$) δ 210.2, 200.7, 153.0, 146.8, 140.7, 138.2, 135.6, 135.0, 128.7, 128.4, 127.9, 127.7, 126.3, 124.6, 124.2, 117.8, 74.4, 37.8, 36.4, 21.5, 21.5, 20.4.
HRMS (ESI-TOF) calcd for $C_{22}H_{22}NaO_2^+$ ([M]+Na$^+$) = 341.1512, found 341.1516.
Chiral HPLC spectrum **7ah**:

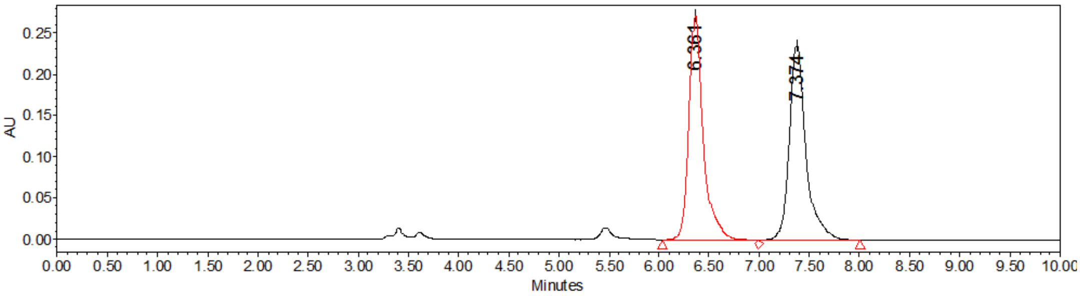

Peak	Retention Time	Area	% Area
1	6.361	2703406	50.09
2	7.374	2693950	49.91

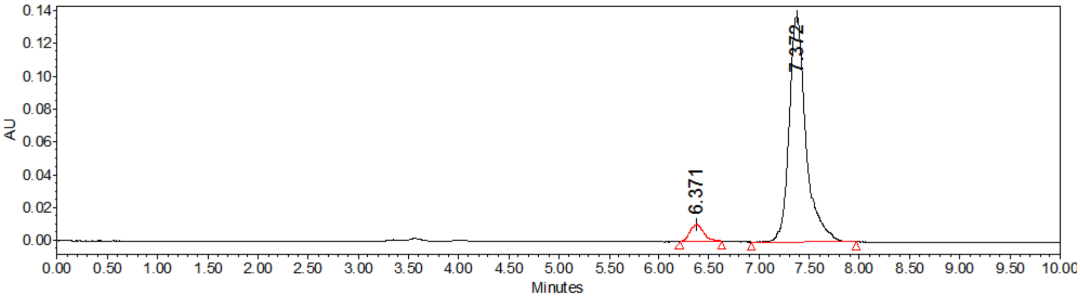

Peak	Retention Time	Area	% Area
1	6.371	93018	5.55
2	7.372	1584042	94.45

2-Isobutyryl-2-(1-(thiophen-2-yl)vinyl)-2,3-dihydro-1*H*-inden-1-one

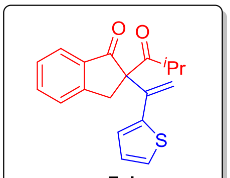

7ak

colorless oil; 99% yield; 96.5:3.5 e.r.; R_f = 0.50 (petroleum ether/ethyl acetate = 10/1); $[\alpha]^{23}_D$ = -460.3 (*c* = 0.54, in DCM).
HPLC (Chiralcel **IA**, hexane/*i*-PrOH = 98/2, flow rate 1.0 mL/min, λ = 254 nm) t_r (minor) = 7.23 min, t_r (major) = 8.79 min.
IR (neat): 3076, 2974, 2872, 1698, 1607, 1465, 1276, 1125, 1096, 983, 904, 790 cm^{-1}.
^{1}H NMR (400 MHz, $CDCl_3$) δ 7.69 (dt, *J* = 7.6, 1.2 Hz, 1H), 7.56 – 7.52 (m, 1H), 7.39 (dt, *J* = 7.6, 1.2 Hz, 1H), 7.32 – 7.28 (m, 1H), 7.10 (dd, *J* = 5.2, 1.2 Hz, 1H), 6.87 – 6.85 (m, 1H), 6.75 (dd, *J* = 3.6, 0.8 Hz, 1H), 5.69 (s, 1H), 5.46 (s, 1H), 4.41 (d, *J* = 17.2 Hz, 1H), 3.57 (hept, *J* = 6.4 Hz, 1H), 3.10 (d, *J* = 17.2 Hz, 1H), 0.91 (d, *J* = 6.4 Hz, 3H), 0.64 (d, *J* = 6.4 Hz, 3H).
^{13}C{^{1}H} NMR (101 MHz, $CDCl_3$) δ 210.4, 200.1, 153.1, 143.7, 140.0, 135.8, 134.9 , 127.8, 127.6, 126.4, 125.6, 125.0, 124.7, 115.9, 74.2, 37.1, 36.3, 21.6, 20.4.
HRMS (ESI-TOF) calcd for $C_{19}H_{18}NaO_2S^+$ ([M]+Na^+) = 333.0920, found 333.0924.
Chiral HPLC spectrum **7ak**:

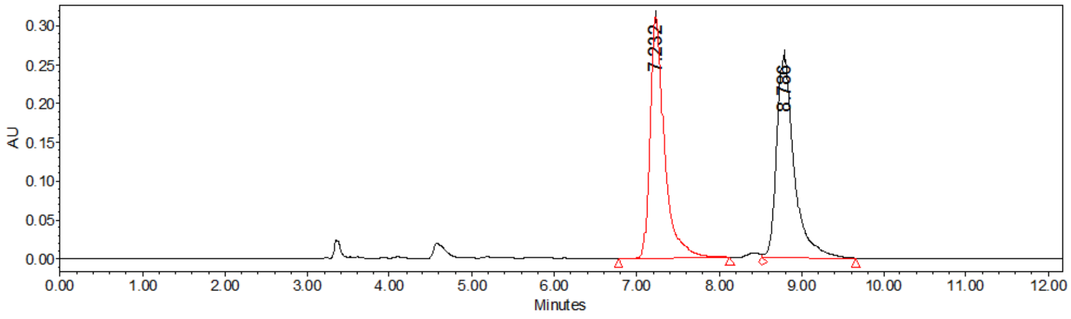

Peak	Retention Time	Area	% Area
1	7.232	3802702	49.84
2	8.786	3826673	50.16

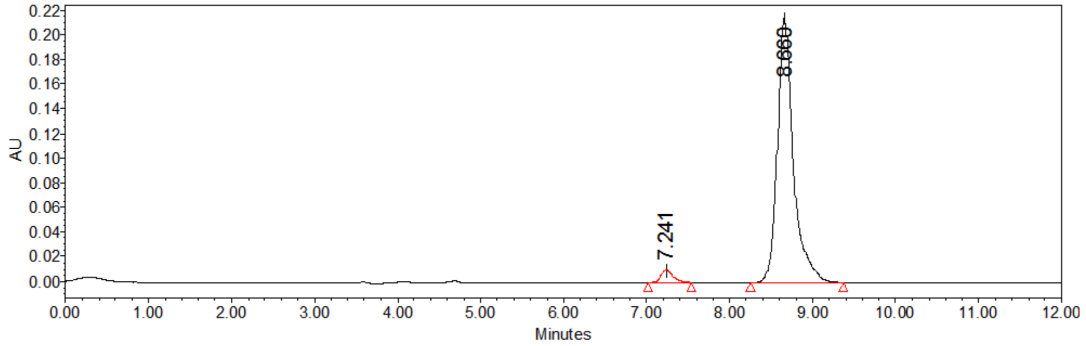

Peak	Retention Time	Area	% Area
1	7.241	106397	3.54
2	8.660	2902469	96.46

2-(1-Cyclopropylvinyl)-2-isobutyryl-2,3-dihydro-1*H*-inden-1-one

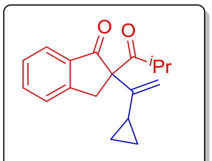

7am

colorless oil; 84% yield; 96.5:3.5 e.r.; R_f = 0.55 (petroleum ether/ethyl acetate = 10/1); $[\alpha]^{23}_D$ = -217.8 (*c* = 0.35, in DCM).
HPLC (Chiralcel **OJH**, hexane/*i*-PrOH = 95/5, flow rate 1.0 mL/min, λ = 254 nm) t_r (major) = 5.36 min, t_r (minor) = 7.25 min.
IR (neat): 3081, 2974, 2872, 1698, 1607, 1465, 1276, 1096, 1067, 983, 904, 790, 704 cm^{-1}.
^{1}H NMR (400 MHz, $CDCl_3$) δ 7.66 (d, *J* = 7.6 Hz, 1H), 7.52 (td, *J* = 7.6, 1.2 Hz, 1H), 7.39 (d, *J* = 7.6 Hz, 1H), 7.28 (t, *J* = 7.6 Hz, 1H), 5.04 (s, 1H), 4.74 (s, 1H), 4.18 (d, *J* = 17.6 Hz, 1H), 3.61 (hept, *J* = 6.8 Hz, 1H), 3.16 (d, *J* = 17.2 Hz, 1H), 1.10 – 1.06 (m, 1H), 1.05 (d, *J* = 6.8 Hz, 3H), 0.87 (d, *J* = 6.4 Hz, 3H), 0.80 – 0.74 (m, 1H), 0.65 – 0.58 (m, 1H), 0.54 – 0.48 (m, 1H), 0.44 – 0.38 (m, 1H).
^{13}C{^{1}H} NMR (101 MHz, $CDCl_3$) δ 209.8, 200.7, 152.6, 148.4, 135.7, 135.4, 127.6, 126.3, 124.4, 108.5, 75.6, 35.8, 35.7, 20.8, 20.6, 14.4, 10.4, 7.8.
HRMS (ESI-TOF) calcd for $C_{18}H_{20}KO_2^+$ ($[M]+K^+$) = 307.1095, found 307.1098.
Chiral HPLC spectrum **7am**:

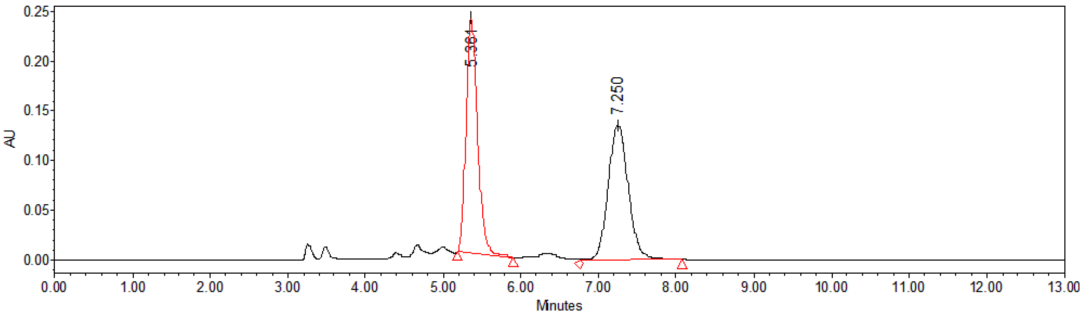

Peak	Retention Time	Area	% Area
1	5.361	2418554	49.99
2	7.250	2419650	50.01

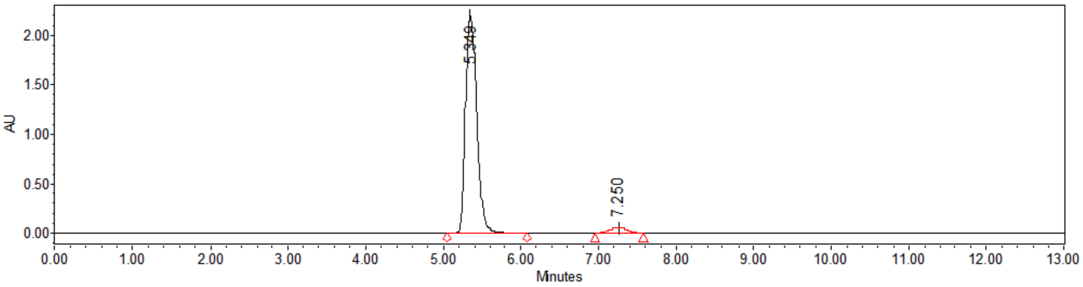

Peak	Retention Time	Area	% Area
1	5.355	3725527	96.48
2	7.247	135893	3.52

(*E*)-2-Benzoyl-*N*-(tert-butyl)-3-phenylbut-2-enamide

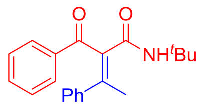

9aa

colorless oil; 96% yield; R_f = 0.20 (petroleum ether/ethyl acetate = 8/1). E/Z = > 19/1 (determined by ^{1}H NMR).

IR (neat): 3361, 2967, 2924, 1652, 1597, 1520, 1449, 1392, 1365, 1275, 1222, 1178, 1024, 917, 846, 822, 764, 696 cm^{-1}.

^{1}H NMR (400 MHz, $CDCl_3$) δ 7.73 – 7.70 (m, 2H), 7.36 – 7.31 (m, 1H), 7.22 – 7.18 (m, 2H), 7.07 – 7.02 (m, 5H), 6.60 (s, 1H), 2.43 (s, 3H), 1.40 (s, 9H).

^{13}C{^{1}H} NMR (101 MHz, $CDCl_3$) δ 198.2, 164.3, 147.4, 141.1, 137.2, 135.9, 133.0, 129.5, 128.1, 128.0, 127.6, 51.7, 28.7, 21.8, 21.8.

HRMS (ESI-TOF) calcd for $C_{21}H_{24}NO_2^+$ ([M]+H$^+$) = 322.1801, found 322.1797.

2-Benzoyl-*N*-(*tert*-butyl)-2-fluoro-3-phenylbut-3-enamide

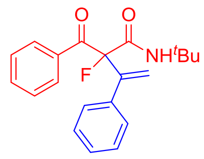

9ja

colorless oil; 46% yield; 91.5:8.5 e.r.; R_f = 0.45 (petroleum ether/ethyl acetate = 6/1); $[\alpha]^{26}_D$ = +38.8 (*c* = 0.33, in DCM).
HPLC (Chiralcel **IA**, hexane/*i*-PrOH = 90/10, flow rate 1.0 mL/min, λ = 254 nm) t_r (minor) = 6.28 min, t_r (major) = 7.79 min.
IR (neat): 3427, 3368, 2969, 2930, 1702, 1684, 1516, 1449, 1220, 1186, 1071, 920, 896, 841, 776 cm^{-1}.
^{1}H NMR (400 MHz, $CDCl_3$) δ 7.98 (dt, *J* = 8.0, 1.2 Hz, 2H), 7.56 – 7.51 (m, 1H), 7.42 – 7.38 (m, 2H), 7.36 – 7.27 (m, 5H), 6.38 (s, 1H), 5.71 (s, 1H), 5.60 (d, *J* = 3.6 Hz, 1H), 1.40 (s, 9H).
^{13}C{^{1}H} NMR (101 MHz, $CDCl_3$) δ 192.9 (d, J_{C-F} = 25.9), 164.1 (d, J_{C-F} = 21.7), 144.2, 144.0, 137.3, 134.1 (d, J_{C-F} = 2.7), 133.5, 130.3 (d, J_{C-F} = 4.3), 128.3, 128.2, 128.0 (d, J_{C-F} = 1.5), 121.4 (d, J_{C-F} = 7.0), 102.0, 52.2, 28.5.
^{19}F{^{1}H} NMR (376 MHz, $CDCl_3$) δ –143.5 (s, 1F).
HRMS (ESI-TOF) calcd for $C_{21}H_{22}FNNaO_2^+$ ([M]+Na^+) = 362.1527, found 362.1519.
Chiral HPLC spectrum **9ja**:

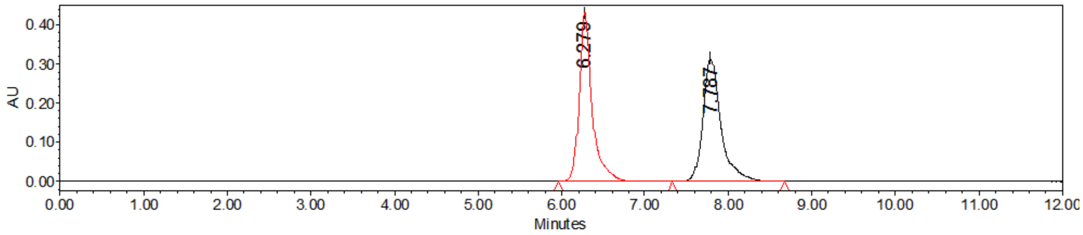

Peak	Retention Time	Area	% Area
1	6.279	4724864	50.01
2	7.787	4723028	49.99

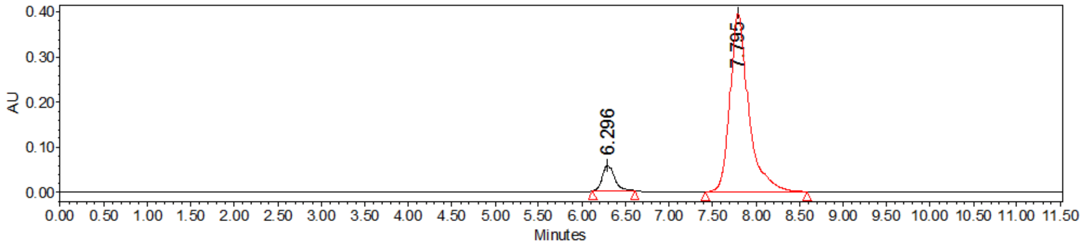

Peak	Retention Time	Area	% Area
1	6.296	564569	8.64
2	7.795	5969838	91.36

***N*-(*tert*-butyl)-2-fluoro-2-(4-fluorobenzoyl)-3-phenylbut-3-enamide**

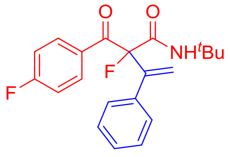

9ka

colorless oil; 54% yield; 89:11 e.r.; R_f = 0.45 (petroleum ether/ethyl acetate = 6/1); $[\alpha]^{27}_{D}$ = +40.6 (*c* = 0.33, in DCM).
HPLC (Chiralcel **IA**, hexane/*i*-PrOH = 90/10, flow rate 1.0 mL/min, λ = 254 nm) t_r (minor) = 6.08 min, t_r (major) = 7.09 min.
IR (neat): 3434, 3368, 2927, 2872, 1684, 1597, 1509, 1456, 1366, 1237, 1160, 1074, 921, 898, 854, 805, 776 cm^{-1}.
^{1}H NMR (400 MHz, $CDCl_3$) δ 8.07 – 8.03 (m, 2H), 7.34 – 7.26 (m, 5H), 7.08 – 7.03 (m, 2H), 6.40 (d, *J* = 3.6 Hz, 1H), 5.73 (s, 1H), 5.61 (d, *J* = 4.0 Hz, 1H), 1.40 (s, 9H).
^{13}C{^{1}H} NMR (101 MHz, $CDCl_3$) δ 191.2 (d, $J_{C\text{-}F}$ = 25.1), 165.8 (d, $J_{C\text{-}F}$ = 254.8), 164.1 (d, $J_{C\text{-}F}$ = 21.6), 144.1 (d, $J_{C\text{-}F}$ = 19.9), 137.2, 133.2 (dd, $J_{C\text{-}F}$ = 4.2, 9.4), 130.4 (t, $J_{C\text{-}F}$ = 2.8), 128.3, 128.3 , 127.8 (d, $J_{C\text{-}F}$ = 1.5), 121.6 (d, $J_{C\text{-}F}$ = 7.3), 115.4 (d, $J_{C\text{-}F}$ = 21.7), 100.9 (d, $J_{C\text{-}F}$ = 196.5), 52.2, 28.5.
^{19}F{^{1}H} NMR (376 MHz, $CDCl_3$) δ –103.8 (s, 1F), –142.2(s, 1F).
HRMS (ESI-TOF) calcd for $C_{21}H_{22}F_2NO_2^+$ ([M]+H^+) = 358.1613, found 358.1603.
Chiral HPLC spectrum **9ka**:

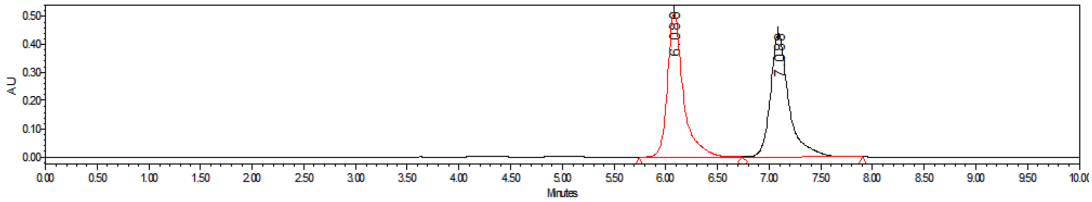

Peak	Retention Time	Area	% Area
1	6.080	5463054	50.03
2	7.088	5456982	49.97

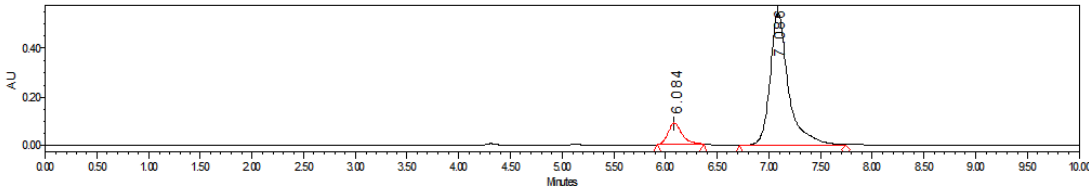

Peak	Retention Time	Area	% Area
1	6.084	825086	10.79
2	7.086	6822129	89.21

***N*-(*tert*-butyl)-2-fluoro-2-(4-methoxybenzoyl)-3-phenylbut-3-enamide**

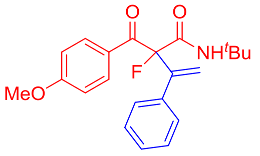

9la

colorless oil; 62% yield; 93:7 e.r.; R_f = 0.45 (petroleum ether/ethyl acetate = 6/1); $[\alpha]^{27}_D$ = +23.5 (*c* = 0.43, in DCM).
HPLC (Chiralcel **IA**, hexane/*i*-PrOH = 90/10, flow rate 1.0 mL/min, λ = 254 nm) t_r (minor) = 9.88 min, t_r (major) = 22.69 min.
IR (neat): 3431, 3368, 2968, 2929, 1696, 1599, 1512, 1457, 1313, 1254, 1223, 1176, 1074, 1027, 921, 850, 776 cm^{-1}.
^{1}H NMR (400 MHz, $CDCl_3$) δ 7.96 – 7.93 (m, 2H), 7.29 – 7.17 (m, 5H), 6.82 – 6.78 (m, 2H), 6.32 (d, *J* = 4.4 Hz, 1H), 5.62 (s, 1H), 5.51 (d, *J* = 3.6 Hz, 1H), 3.77 (s, 3H), 1.32 (s, 9H).
^{13}C{^{1}H} NMR (101 MHz, $CDCl_3$) δ 191.1 (d, $J_{C\text{-}F}$ = 24.9), 164.3 (d, $J_{C\text{-}F}$ = 21.7), 163.9, 144.4 (d, $J_{C\text{-}F}$ = 20.3), 137.4, 133.0 (d, $J_{C\text{-}F}$ = 4.4), 128.3, 128.2, 127.9 (d, $J_{C\text{-}F}$ = 1.4), 126.8 (d, $J_{C\text{-}F}$ = 2.8), 121.3 (d, $J_{C\text{-}F}$ = 7.2), 113.5, 101.0 (d, $J_{C\text{-}F}$ = 196.2), 55.5, 52.1, 28.5.
^{19}F{^{1}H} NMR (376 MHz, $CDCl_3$) δ –142.8 (s, 1F).
HRMS (ESI-TOF) calcd for $C_{22}H_{25}FNO_3^+$ ($[M+H]^+$) = 370.1813, found 370.1804.
Chiral HPLC spectrum **9la**:

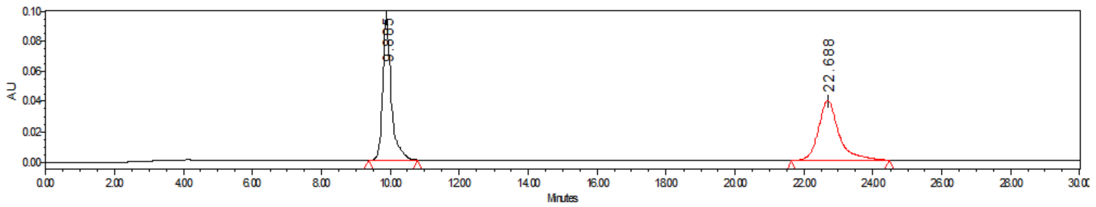

Peak	Retention Time	Area	% Area
1	9.885	1644776	50.17
2	22.688	1633592	49.83

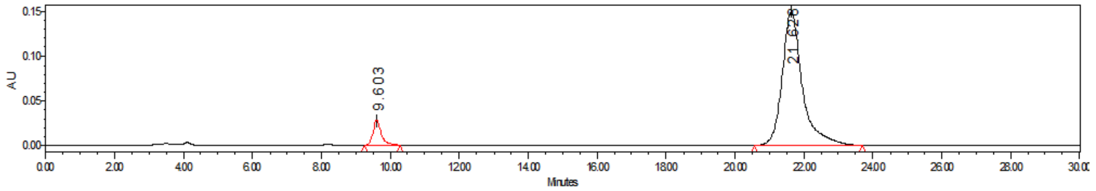

Peak	Retention Time	Area	% Area
1	9.603	467951	7.13
2	21.628	6093605	92.87

2-([1,1'-Biphenyl]-4-carbonyl)-*N*-(*tert*-butyl)-2-fluoro-3-phenylbut-3-enamide

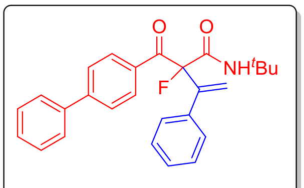

9ma

colorless oil; 43% yield; 89:11 e.r.; R_f = 0.42 (petroleum ether/ethyl acetate = 10/1). $[\alpha]^{27}_D$ = +19.7 (*c* = 0.32, in DCM).
HPLC (Chiralcel **IA**, hexane/*i*-PrOH = 90/10, flow rate 1.0 mL/min, λ = 254 nm) t_r (minor) = 9.26 min, t_r (major) = 19.36 min.
IR (neat): 3426, 2969, 2930, 1700, 1682, 1602, 1514, 1453, 1406, 1394, 1366, 1254, 1221, 1195, 1075, 920, 898, 857, 833, 774, 749, 735 cm^{-1}.
^{1}H NMR (400 MHz, $CDCl_3$) δ 8.08 (dd, *J* = 8.4, 1.6 Hz, 2H), 7.63 – 7.59 (m, 4H), 7.51 – 7.43 (m, 3H), 7.41 – 7.35 (m, 3H), 7.32 – 7.27 (m, 3H), 6.41 (d, *J* = 4.4 Hz, 1H), 5.73 (s, 1H), 5.63 (d, *J* = 3.6 Hz, 1H), 1.42 (s, 9H).

^{13}C{^{1}H} NMR (101 MHz, $CDCl_3$) δ 192.3 (d, $J_{C\text{-}F}$ = 25.6), 164.2 (d, $J_{C\text{-}F}$ = 21.6), 146.1, 144.1 (d, $J_{C\text{-}F}$ = 20.3), 139.7, 133.3, 132.7 (d, $J_{C\text{-}F}$ = 2.6), 130.9 (d, $J_{C\text{-}F}$ = 4.2), 128.9, 128.3, 128.3, 128.2, 128.0 (d, $J_{C\text{-}F}$ = 1.4), 127.3, 126.8, 121.5 (d, $J_{C\text{-}F}$ = 7.3), 101.0 (d, $J_{C\text{-}F}$ = 196.6), 52.1, 28.5.
^{19}F{^{1}H} NMR (376 MHz, $CDCl_3$) δ –143.2 (s, 1F).
HRMS (ESI-TOF) calcd for $C_{27}H_{27}FNO_2^+$ ([M]+H$^+$) = 416.2020, found 416.2022.
Chiral HPLC spectrum **9ma**:

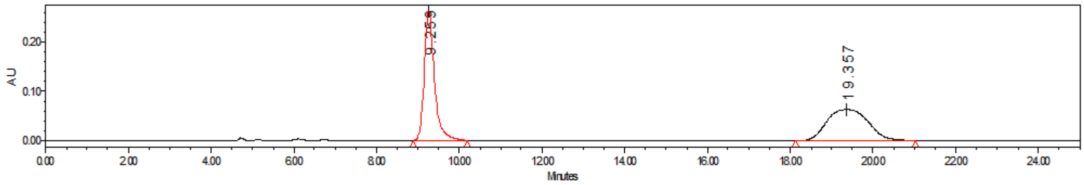

Peak	Retention Time	Area	% Area
1	9.259	4546418	50.28
2	19.357	4495258	49.72

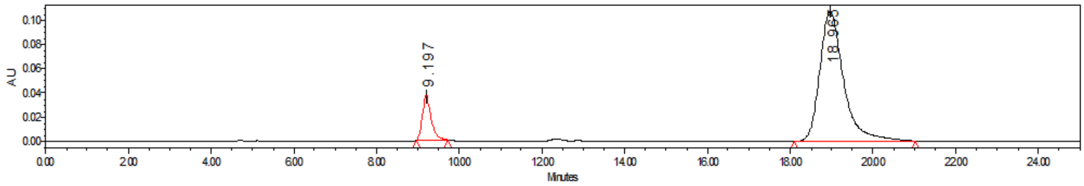

Peak	Retention Time	Area	% Area
1	9.197	540015	11.14
2	18.965	4305533	88.86

2-Benzoyl-*N*-(*tert*-butyl)-2-fluoro-3-(2-fluorophenyl)but-3-enamide

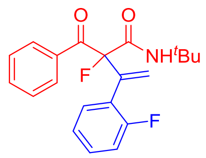

9jb

colorless oil; 40% yield; 91:9 e.r.; R_f = 0.45 (petroleum ether/ethyl acetate = 6/1); $[\alpha]^{27}_{D}$ = +15.2 (*c* = 0.27, in DCM).
HPLC (Chiralcel **IG**, hexane/*i*-PrOH = 90/10, flow rate 1.0 mL/min, λ = 254 nm) t_r (minor) = 12.21 min, t_r (major) = 16.68 min.
IR (neat): 3433, 3369, 2970, 2933, 2914, 1705, 1685, 1517, 1490, 1450, 1395, 1366, 1241, 1218, 1094, 1031, 933, 918, 841, 763, 749, 693 cm^{-1}.
^{1}H NMR (400 MHz, $CDCl_3$) δ 7.99 – 7.96 (m, 2H), 7.57 – 7.52 (m, 1H), 7.44 – 7.39 (m, 2H), 7.36 – 7.27 (m, 2H), 7.12 (td, *J* = 7.6, 1.2 Hz, 1H), 7.07 – 7.02 (m, 1H), 6.30 (s, 1H), 5.77 (d, *J* = 2.8 Hz, 1H), 5.66 (d, *J* = 2.0 Hz, 1H), 1.40 (s, 9H).
^{13}C{^{1}H} NMR (101 MHz, $CDCl_3$) δ 191.8 (d, $J_{C\text{-}F}$ = 26.5), 163.9 (d, $J_{C\text{-}F}$ = 20.9), 160.0 (d, $J_{C\text{-}F}$ = 246.1), 137.7 (d, $J_{C\text{-}F}$ = 23.7), 133.9 (d, $J_{C\text{-}F}$ = 3.0), 133.4, 131.1 (d, $J_{C\text{-}F}$ = 3.3), 130.1 (d, $J_{C\text{-}F}$ = 4.8), 130.0 (d, $J_{C\text{-}F}$ = 8.2), 128.2, 125.0 (d, $J_{C\text{-}F}$ = 15.6), 124.0 (d, $J_{C\text{-}F}$ = 3.6), 123.8 (d, $J_{C\text{-}F}$ = 6.0), 115.5 (d, $J_{C\text{-}F}$ = 22.4), 100.4 (d, $J_{C\text{-}F}$ = 198.4.9), 52.0, 28.4.
^{19}F{^{1}H} NMR (376 MHz, $CDCl_3$) δ –112.3 (d, $J_{F\text{-}F}$ = 9.8, 1F), δ –148.2 (d, $J_{F\text{-}F}$ = 9.8, 1F).
HRMS (ESI-TOF) calcd for $C_{21}H_{22}F_2NO_2^+$ ([M]+H$^+$) = 358.1613, found 358.1604.
Chiral HPLC spectrum **9jb**:

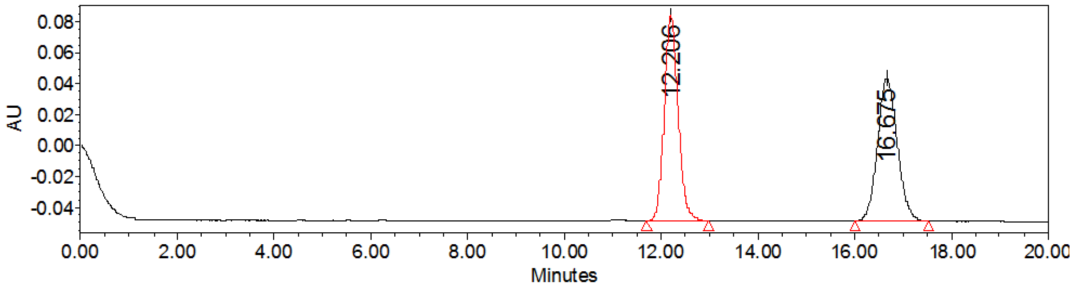

Peak	Retention Time	Area	% Area
1	12.206	2655608	50.29
2	16.675	2624564	49.71

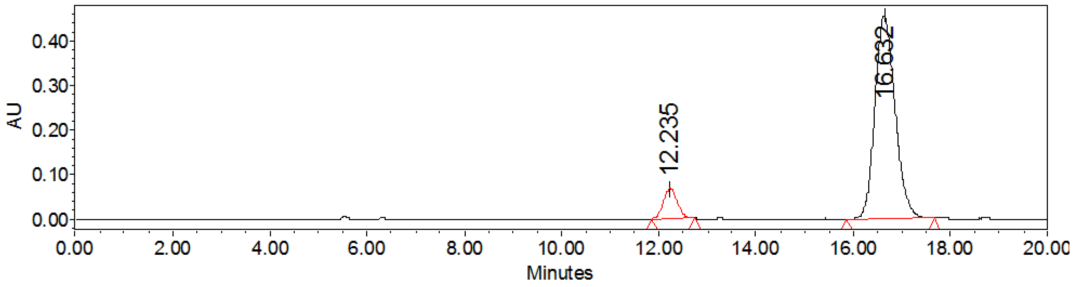

Peak	Retention Time	Area	% Area
1	12.235	1315517	9.13
2	16.632	13088086	90.87

2-Benzoyl-*N*-(*tert*-butyl)-2-fluoro-3-(4-fluorophenyl)but-3-enamide

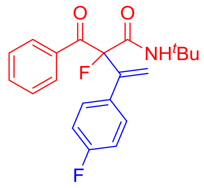

9je

colorless oil; 48% yield; 92:8 e.r.; R_f = 0.45 (petroleum ether/ethyl acetate = 6/1); $[\alpha]^{26}_D$ = +29.1 (*c* = 0.33, in DCM).
HPLC (Chiralcel **IA**, hexane/*i*-PrOH = 90/10, flow rate 1.0 mL/min, λ = 254 nm) t_r (minor) = 6.21 min, t_r (major) = 7.53 min.
IR (neat): 3429, 3367, 2967, 2925, 1702, 1684, 1597, 1517, 1492, 1451, 1395, 1366, 1278, 1252, 1091, 920, 897, 842 cm^{-1}.
^{1}H NMR (400 MHz, $CDCl_3$) δ 7.97 (dt, *J* = 8.4, 1.2 Hz, 2H), 7.56 – 7.51 (m, 1H), 7.42 – 7.38 (m, 2H), 7.35 – 7.29 (m, 2H), 6.99 – 6.94 (m, 2H), 6.38 (s, 1H), 5.67 (s, 1H), 5.61 (d, *J* = 3.6 Hz, 1H), 1.40 (s, 9H).
^{13}C{^{1}H} NMR (101 MHz, $CDCl_3$) δ 192.7 (d, J_{C-F} = 25.9), 164.0 (d, J_{C-F} = 21.5), 162.7 (d, J_{C-F} = 246.1), 143.2 (d, J_{C-F} = 20.1), 134.1 (d, J_{C-F} = 2.7), 133.6, 133.4 (d, J_{C-F} = 3.4), 130.2 (d, J_{C-F} = 4.2), 129.9 (d, J_{C-F} = 8.1), 128.3, 121.6 (d, J_{C-F} = 7.2), 115.2 (d, J_{C-F} = 21.4), 100.9 (d, J_{C-F} = 196.7), 52.2, 28.5.
^{19}F{^{1}H} NMR (376 MHz, $CDCl_3$) δ –113.7 (s, 1F), –143.2 (s, 1F).
HRMS (ESI-TOF) calcd for $C_{21}H_{21}F_2NNaO_2^+$ ([M]+Na^+) = 380.1433, found 380.1430.
Chiral HPLC spectrum **9je**:

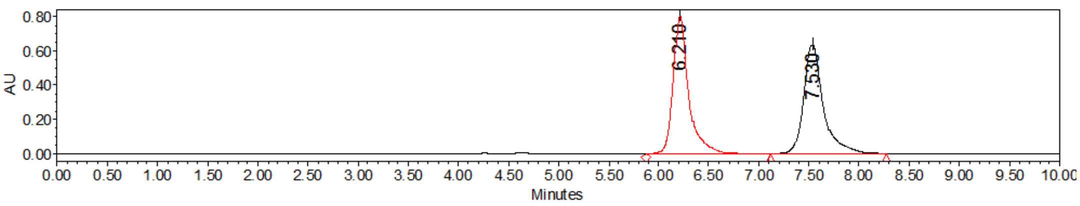

Peak	Retention Time	Area	% Area
1	6.210	8632490	50.10
2	7.530	8596858	49.90

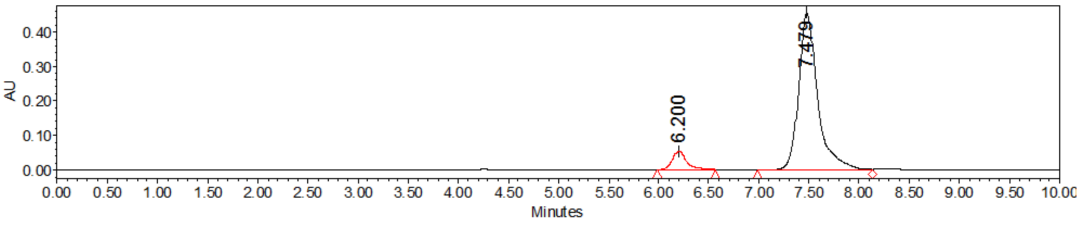

Peak	Retention Time	Area	% Area
1	6.200	533610	8.16
2	7.479	6008240	91.84

2-Benzoyl-*N*-(*tert*-butyl)-2-fluoro-3-(2-methoxyphenyl)but-3-enamide

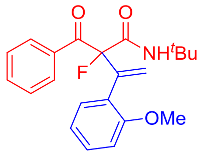

9jf

colorless oil; 80% yield; 92.5:7.5 e.r.; R_f = 0.40 (petroleum ether/ethyl acetate = 6/1); $[\alpha]^{26}_{D}$ = –3.9 (*c* = 0.77, in DCM).
HPLC (Chiralcel **IA**, hexane/*i*-PrOH = 95/5, flow rate 1.0 mL/min, λ = 254 nm) t_r (major) = 7.79 min, t_r (minor) = 8.82 min.
IR (neat): 3431, 2965, 2922, 2852, 1704, 1684, 1598, 1579, 1517, 1492, 1454, 1394, 1366, 1242, 1184, 1099, 1046, 1025, 922, 896, 837, 754, 694 cm^{-1}.
^{1}H NMR (400 MHz, $CDCl_3$) δ 7.98 (dt, *J* = 8.0, 1.2 Hz, 2H), 7.55 – 7.48 (m, 1H), 7.41 (t, *J* = 8.0 Hz, 2H), 7.31 (td, *J* = 8.0, 2.0 Hz, 1H), 7.22 (dd, *J* = 7.6, 1.6 Hz, 1H), 6.96 (td, *J* = 7.6, 0.8 Hz, 1H), 6.84 (d, *J* = 8.4 Hz, 1H), 6.30 (d, *J* = 3.2 Hz, 1H), 5.72 (d, *J* = 1.6 Hz, 1H), 5.54 (d, *J* = 3.2 Hz, 1H), 3.62 (s, 3H), 1.40 (s, 9H).
^{13}C{^{1}H} NMR (101 MHz, $CDCl_3$) δ 192.4 (d, $J_{C\text{-}F}$ = 27.2), 164.3 (d, $J_{C\text{-}F}$ = 21.9), 157.1, 141.0 (d, $J_{C\text{-}F}$ = 25.3), 134.4, 133.1, 131.1, 130.1 (d, $J_{C\text{-}F}$ = 5.4), 129.8, 128.1, 126.8 (d, $J_{C\text{-}F}$ = 2.7), 121.4 (d, $J_{C\text{-}F}$ = 6.2), 120.7, 110.6, 100.6 (d, $J_{C\text{-}F}$ = 198.9), 55.1, 51.7, 28.6.
^{19}F{^{1}H} NMR (376 MHz, $CDCl_3$) δ –151.5 (s, 1F).
HRMS (ESI-TOF) calcd for $C_{22}H_{25}FNO_3^+$ ([M]+H$^+$) = 370.1813, found 370.1822.
Chiral HPLC spectrum **9jf**:

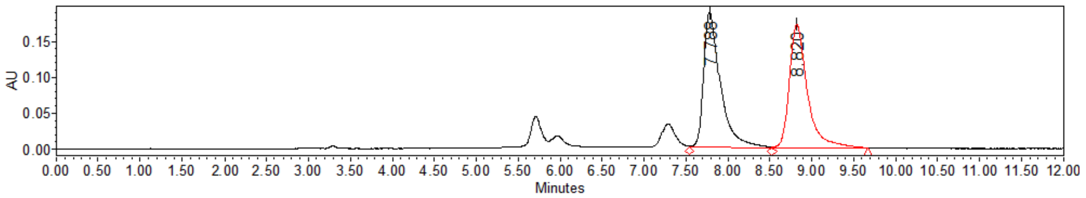

Peak	Retention Time	Area	% Area
1	7.788	2597995	49.80
2	8.820	2618682	50.20

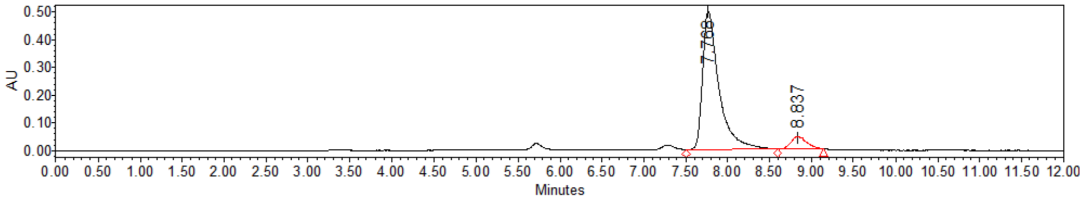

Peak	Retention Time	Area	% Area
1	7.768	6834356	92.57
2	8.837	548539	7.43

2-Benzoyl-*N*-(*tert*-butyl)-3-(4-chlorophenyl)-2-fluorobut-3-enamide

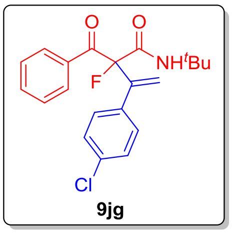

colorless oil; 33% yield; 90:10 e.r.; R_f = 0.45 (petroleum ether/ethyl acetate = 6/1); $[\alpha]^{25}_D$ = +21.5 (*c* = 0.27, in DCM).
HPLC (Chiralcel **IA**, hexane/*i*-PrOH = 90/10, flow rate 1.0 mL/min, λ = 254 nm) t_r (minor) = 6.77 min, t_r (major) = 8.42 min.
IR (neat): ν (cm^{-1}) 3432, 3367, 2969, 2927, 1702, 1684, 1600, 1510, 1452, 1225, 1072, 920, 897, 843 cm^{-1}.
^{1}H NMR (400 MHz, $CDCl_3$) δ 7.99 – 7.95 (m, 2H), 7.56 – 7.52 (m, 1H), 7.43 – 7.38 (m, 2H), 7.30 – 7.24 (m, 4H), 6.37 (s, 1H), 5.70 (s, 1H), 5.63 (d, *J* = 3.6 Hz, 1H), 1.40 (s, 9H).
^{13}C{^{1}H} NMR (101 MHz, $CDCl_3$) δ 192.6 (d, $J_{C\text{-}F}$ = 25.6), 164.0 (d, $J_{C\text{-}F}$ = 21.4), 143.1 (d, $J_{C\text{-}F}$ = 20.1), 135.8, 134.3, 134.0 (d, $J_{C\text{-}F}$ = 2.8), 133.7, 130.2 (d, $J_{C\text{-}F}$ = 4.3), 129.4 (d, $J_{C\text{-}F}$ = 1.5), 128.5, 128.3, 121.9 (d, $J_{C\text{-}F}$ = 7.3), 100.8 (d, $J_{C\text{-}F}$ = 197.0), 52.2, 28.5.
^{19}F{^{1}H} NMR (376 MHz, $CDCl_3$) δ –143.2 (s, 1F).
HRMS (ESI-TOF) calcd for $C_{21}H_{22}{}^{34.9689}ClFNO_2^+$ ($[M]+H^+$) = 374.1318, found 374.1328, and $C_{21}H_{22}{}^{36.9659}ClFNO_2^+$ ($[M]+H^+$) = 376.1288, found 376.1293.
Chiral HPLC spectrum **9jg**:

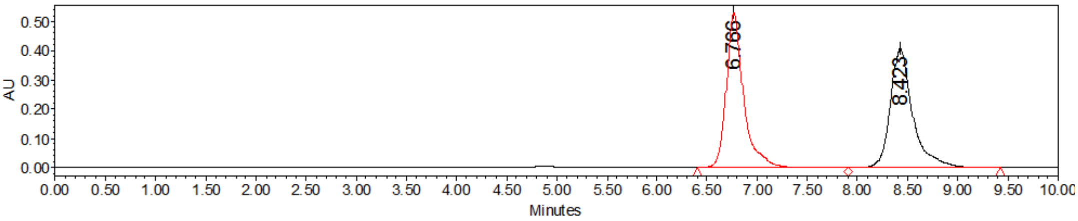

Peak	Retention Time	Area	% Area
1	6.766	6342387	50.00
2	8.423	6341845	50.00

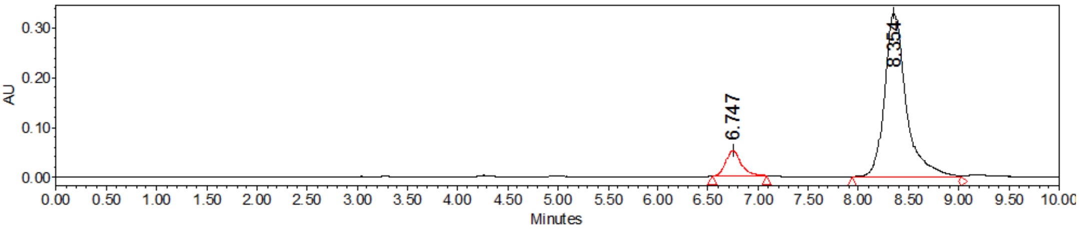

Peak	Retention Time	Area	% Area
1	6.747	557812	10.13
2	8.354	4949679	89.87

2-Benzoyl-*N*-(*tert*-butyl)-2-fluoro-3-(m-tolyl)but-3-enamide

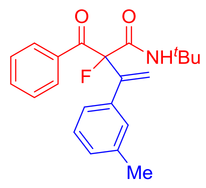

9jh

colorless oil; 68% yield; 88:12 e.r.; R_f = 0.45 (petroleum ether/ethyl acetate = 6/1); $[\alpha]^{27}_D$ = +26.2 (*c* = 0.48, in DCM).
HPLC (Chiralcel **IA**, hexane/*i*-PrOH = 90/10, flow rate 1.0 mL/min, λ = 254 nm) t_r (minor) = 5.74 min, t_r (major) = 7.39 min.
IR (neat): 3368, 2965, 2922, 2854, 1702, 1683, 1598, 1580, 1513, 1450, 1394, 1365, 1243, 1220, 1185, 1070, 1001, 921, 838, 791, 746, 692, 669 cm^{-1}.
^{1}H NMR (400 MHz, $CDCl_3$) δ 7.98 (dt, *J* = 8.0, 1.2 Hz, 2H), 7.55 – 7.51 (m, 1H), 7.42 – 7.38 (m, 2H), 7.20 – 7.08 (m, 4H), 6.37 (s, 1H), 5.69 (s, 1H), 5.56 (d, *J* = 3.6 Hz, 1H), 2.31 (s, 3H), 1.41 (s, 9H).
^{13}C{^{1}H} NMR (101 MHz, $CDCl_3$) δ 192.9 (d, $J_{C\text{-}F}$ = 26.0), 164.1 (d, $J_{C\text{-}F}$ = 21.8), 144.1 (d, $J_{C\text{-}F}$ = 20.7), 137.9, 137.2, 134.1 (d, $J_{C\text{-}F}$ = 2.8), 133.4, 130.3 (d, $J_{C\text{-}F}$ = 4.3), 129.0, 128.7, 128.2, 128.1, 125.0 (d, $J_{C\text{-}F}$ = 1.6), 121.3 (d, $J_{C\text{-}F}$ = 7.2), 101.0 (d, $J_{C\text{-}F}$ = 196.4), 52.1, 28.5, 21.5.
^{19}F{^{1}H} NMR (376 MHz, $CDCl_3$) δ–143.7 (s, 1F).
HRMS (ESI-TOF) calcd for $C_{22}H_{25}FNO_2^+$ ([M]+H^+) = 354.1864, found 354.1862.
Chiral HPLC spectrum **9jh**:

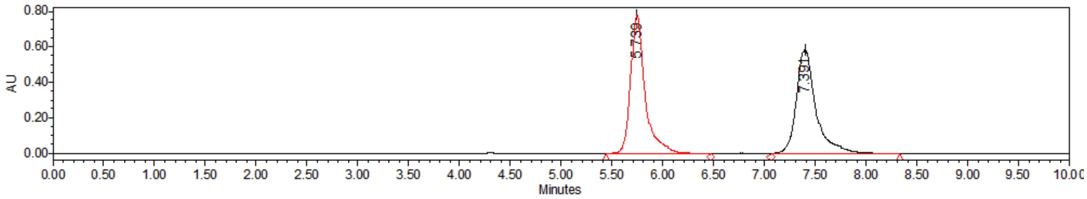

Peak	Retention Time	Area	% Area
1	5.739	7663390	50.02
2	7.391	7657323	49.98

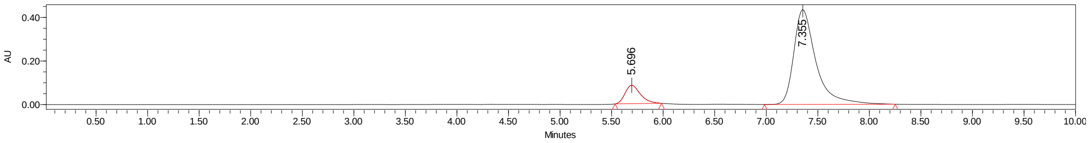

Peak	Retention Time	Area	% Area
1	5.696	894397	11.99
2	7.355	6563255	88.01

2-Benzoyl-*N*-(*tert*-butyl)-2-fluoro-3-(thiophen-3-yl)but-3-enamide

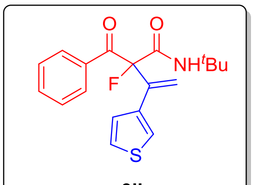

9jl

colorless oil; 74% yield; 92:8 e.r.; R_f = 0.45 (petroleum ether/ethyl acetate = 6/1); $[\alpha]^{26}_D$ = +50.8 (*c* = 0.51, in DCM).
HPLC (Chiralcel **IA**, hexane/*i*-PrOH = 90/10, flow rate 1.0 mL/min, λ = 254 nm) t_r (minor) = 7.40 min, t_r (major) = 9.41 min.
IR (neat): 3423, 2968, 2926, 1701, 1682, 1517, 1451, 1220, 1186, 1070, 928, 832, 791 cm^{-1}.
^{1}H NMR (400 MHz, $CDCl_3$) δ 8.00 (dt, *J* = 8.0, 1.2 Hz, 2H), 7.56 – 7.51 (m, 1H), 7.42 – 7.38 (m, 2H), 7.29 – 7.26 (m, 1H), 7.23 – 7.19 (m, 1H), 7.15 – 7.11 (m, 1H), 6.38 (s, 1H), 5.81 (s, 1H), 5.57 (d, *J* = 3.6 Hz, 1H), 1.41 (s, 9H).
^{13}C{^{1}H} NMR (101 MHz, $CDCl_3$) δ 193.0 (d, $J_{C\text{-}F}$ = 25.6), 164.1 (d, $J_{C\text{-}F}$ = 21.4), 139.0 (d, $J_{C\text{-}F}$ = 20.3), 137.4, 134.3 (d, $J_{C\text{-}F}$ = 2.6), 133.5, 130.2 (d, $J_{C\text{-}F}$ = 4.0), 128.2, 127.0, 125.5, 123.4 (d, $J_{C\text{-}F}$ = 3.3), 119.5 (d, $J_{C\text{-}F}$ = 7.6), 100.8 (d, $J_{C\text{-}F}$ = 196.3), 52.2, 28.5.
^{19}F{^{1}H} NMR (376 MHz, $CDCl_3$) δ –143.8 (s, 1F).
HRMS (ESI-TOF) calcd for $C_{19}H_{20}FSNNaO_2^+$ ($[M]+Na^+$) = 368.1091, found 368.1092.
Chiral HPLC spectrum **9jl**:

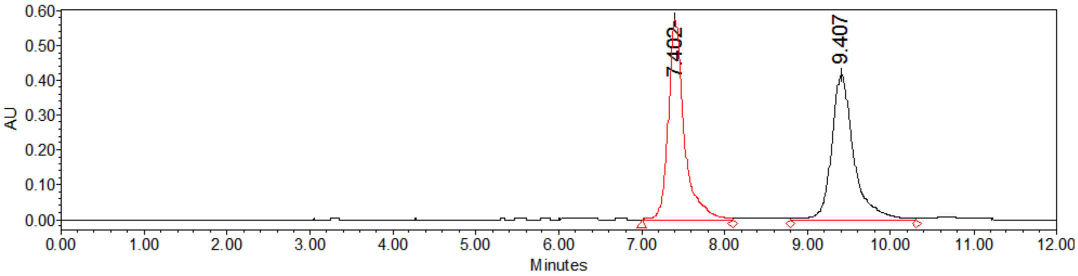

Peak	Retention Time	Area	% Area
1	7.402	7607935	50.07
2	9.407	7587878	49.93

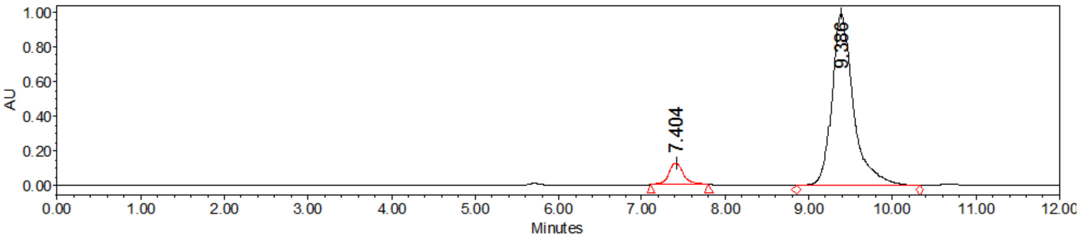

Peak	Retention Time	Area	% Area
1	7.404	1520944	7.82
2	9.386	17916514	92.18

2-Benzoyl-*N*-(*tert*-butyl)-3-cyclopropyl-2-fluorobut-3-enamide

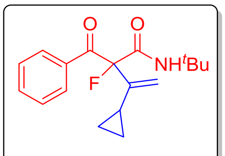

9jm

colorless oil; 60% yield; 90:10 e.r.; R_f = 0.45 (petroleum ether/ethyl acetate = 6/1); $[\alpha]^{27}_D$ = +39.1 (*c* = 0.33, in DCM).
HPLC (Chiralcel **IG**, hexane/*i*-PrOH = 90/10, flow rate 1.0 mL/min, λ = 254 nm) t_r (minor) = 11.56 min, t_r (major) = 16.06 min.
IR (neat): 3368, 2967, 2924, 2854, 1701, 1683, 1598, 1517, 1451, 1394, 1366, 1256, 1222, 1186, 1054, 1024, 889, 841, 692, 666 cm^{-1}.
^{1}H NMR (400 MHz, $CDCl_3$) δ 8.03 – 8.00 (m, 2H), 7.56 – 7.54 (m, 1H), 7.45 – 7.41 (m, 2H), 6.34 (s, 1H), 5.24 (d, *J* = 2.0 Hz, 1H), 5.08 (dd, *J* = 2.0, 1.2 Hz, 1H), 1.58 – 1.51 (m, 1H), 1.41 (s, 9H), 0.77 – 0.65 (m, 2H), 0.59 – 0.48 (m, 2H).
^{13}C{^{1}H} NMR (101 MHz, $CDCl_3$) δ 193.0 (d, $J_{C\text{-}F}$ = 26.4), 164.5 (d, $J_{C\text{-}F}$ = 21.3), 145.9 (d, $J_{C\text{-}F}$ = 19.5), 134.6 (d, $J_{C\text{-}F}$ = 2.9), 133.4, 130.1 (d, $J_{C\text{-}F}$ = 4.5), 128.2, 112.4 (d, $J_{C\text{-}F}$ = 8.6), 101.4 (d, $J_{C\text{-}F}$ = 196.8), 51.9 , 28.6 , 12.5 (d, $J_{C\text{-}F}$ = 4.9), 7.6, 7.2.
^{19}F{^{1}H} NMR (376 MHz, $CDCl_3$) δ –148.4 (s, 1F).
HRMS (ESI-TOF) calcd for $C_{18}H_{23}FNO_2^+$ ([M]+H$^+$) = 304.1707, found 304.1704.
Chiral HPLC spectrum **9jm**:

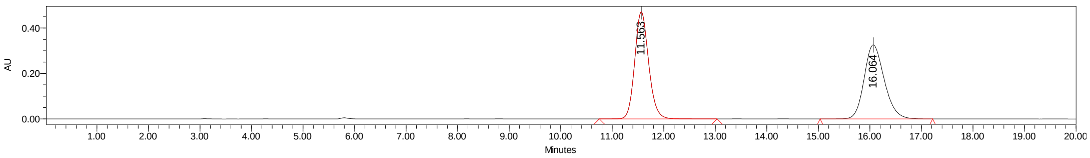

Peak	Retention Time	Area	% Area
1	11.563	8825319	50.04
2	16.064	8811441	49.96

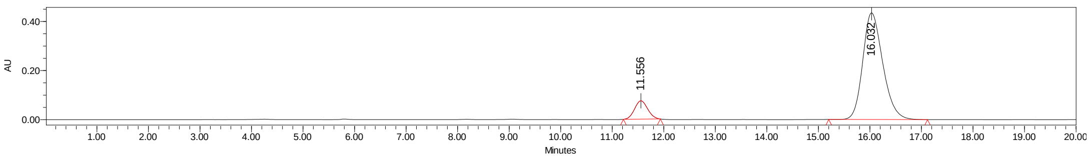

Peak	Retention Time	Area	% Area
1	11.556	1322794	10.14
2	16.032	11717211	89.86

2-Benzoyl-*N*-(*tert*-butyl)-2-fluoro-3-methylenehexanamide

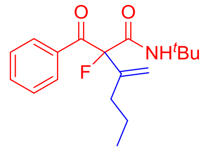

9jn

colorless oil; 33% yield; 87:13 e.r.; R_f = 0.45 (petroleum ether/ethyl acetate = 10/1); $[\alpha]26_D$ = +11.3 (*c* = 0.23, in DCM).
HPLC (Chiralcel **IG**, hexane/*i*-PrOH = 90/10, flow rate 1.0 mL/min, λ = 254 nm) t_r (minor) = 8.55 min, t_r (major) = 12.73 min.
IR (neat): 3436, 3368, 2963, 2930, 1701, 1684, 1518, 1452, 1366, 1256, 1220, 1062, 916, 837, 746 cm^{-1}.
^{1}H NMR (400 MHz, $CDCl_3$) δ 8.05 – 7.97 (m, 2H), 7.57 – 7.53(m, 1H), 7.43 (t, *J* = 7.6 Hz, 2H), 6.27 (s, 1H), 5.35 – 5.30 (m, 2H), 2.16 (t, *J* = 7.6 Hz, 2H), 1.56 – 1.49 (m, 2H), 1.40 (s, 9H), 0.93 (t, *J* = 7.6 Hz, 3H).
^{13}C{^{1}H} NMR (101 MHz, $CDCl_3$) δ 193.0 (d, J_{C-F} = 26.6), 164.5 (d, J_{C-F} = 21.7), 144.0 (d, J_{C-F} = 20.3), 134.4 (d, J_{C-F} = 3.1), 133.4, 130.1 (d, J_{C-F} = 4.4), 128.3, 116.0 (d, J_{C-F} = 8.6), 101.5 (d, J_{C-F} = 195.4), 51.9, 33.4 (d, J_{C-F} = 3.4), 28.5, 21.0, 13.8.
^{19}F{^{1}H} NMR (376 MHz, $CDCl_3$) δ –150.2 (s, 1F).
HRMS (ESI-TOF) calcd for $C_{18}H_{24}FNNaO_2^+$ ([M]+Na$^+$) = 368.1091, found 368.1092.
Chiral HPLC spectrum **9jn**:

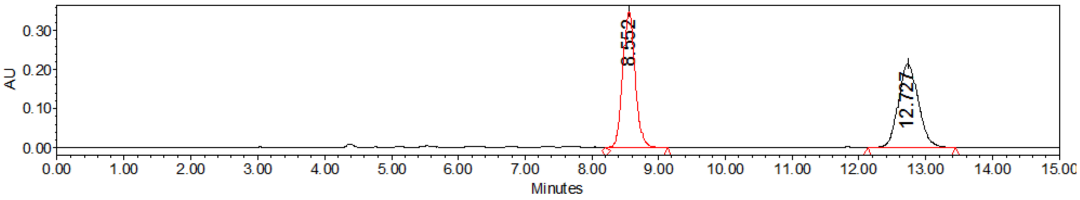

Peak	Retention Time	Area	% Area
1	8.552	4474751	50.06
2	12.727	4464916	49.94

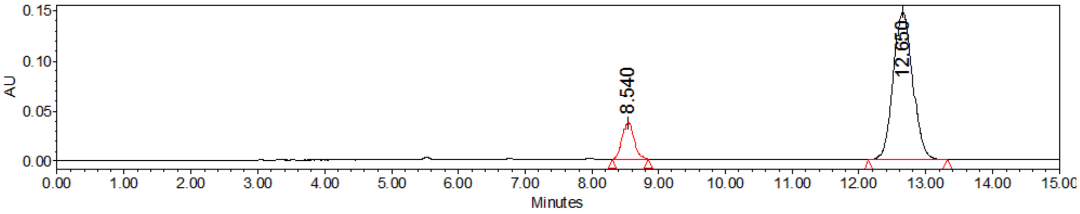

Peak	Retention Time	Area	% Area
1	8.540	458891	13.18
2	12.650	3024099	86.82

2-Benzoyl-*N*-(*tert*-butyl)-2-fluoro-3-(naphthalen-2-yl)but-3-enamide

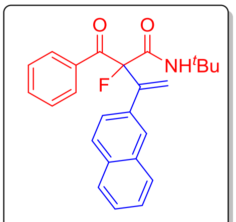

9js

colorless oil; 81% yield; 91:9 e.r.; R_f = 0.42 (petroleum ether/ethyl acetate = 6/1); [α]26$_D$ = +26.4 (*c* = 0.64, in DCM).
HPLC (Chiralcel **IA**, hexane/*i*-PrOH = 90/10, flow rate 1.0 mL/min, λ = 254 nm) t_r (minor) = 6.21 min, t_r (major) = 7.53 min.
IR (neat): 3423, 2965, 2924, 1702, 1682, 1517, 1450, 1221, 1186, 1070, 917, 895, 840 cm^{-1}.
^{1}H NMR (400 MHz, $CDCl_3$) δ 8.00 (dt, *J* = 8.0, 1.2 Hz, 2H), 7.83 – 7.75 (m, 4H), 7.54 – 7.50 (m, 1H), 7.48 – 7.43 (m, 3H), 7.41 – 7.37 (m, 2H), 6.43 (s, 1H), 5.83 (s, 1H), 5.69 (d, *J* = 3.6 Hz, 1H), 1.42 (s, 9H).
^{13}C{^{1}H} NMR (101 MHz, $CDCl_3$) δ 192.9 (d, J_{C-F} = 25.9), 164.2 (d, J_{C-F} = 21.5), 144.0 (d, J_{C-F} = 20.4), 134.7, 134.1 (d, J_{C-F} = 2.9), 133.5, 133.1, 132.9, 130.3 (d, J_{C-F} = 4.2), 128.3, 128.2, 127.9, 127.6, 127.2 (d, J_{C-F} = 2.6), 126.4, 126.3, 125.8 (d, J_{C-F} = 1.3), 121.9 (d, J_{C-F} = 7.1), 101.1 (d, J_{C-F} = 196.6), 52.2, 28.5.
^{19}F{^{1}H} NMR (376 MHz, $CDCl_3$) δ –143.3 (s, 1F).
HRMS (ESI-TOF) calcd for $C_{25}H_{24}FNNaO_2^+$ ([M]+Na^+) = 412.1683, found 412.1680.
Chiral HPLC spectrum **9js**:

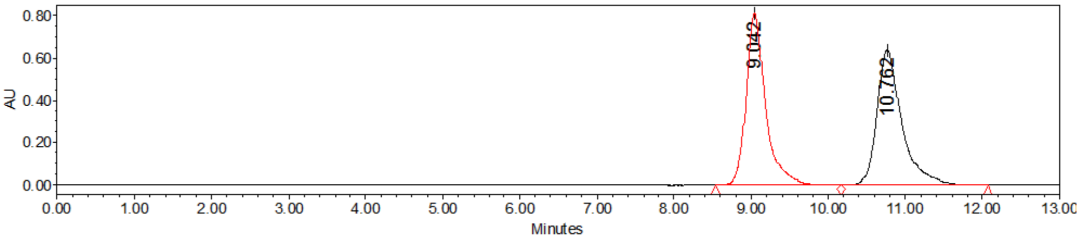

Peak	Retention Time	Area	% Area
1	9.042	14258229	49.98
2	10.762	14269723	50.02

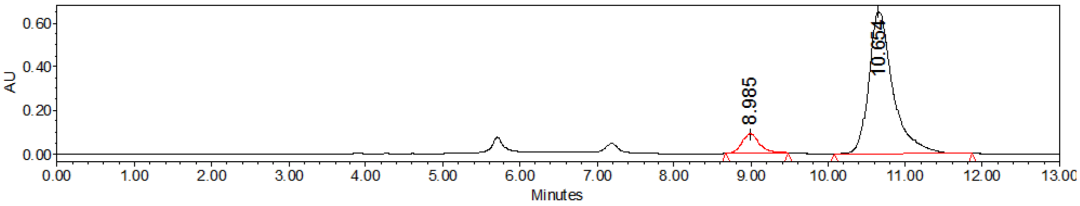

Peak	Retention Time	Area	% Area
1	8.985	1420486	9.12
2	10.654	14153448	90.88

2-Benzoyl-3-(4-bromophenyl)-*N*-(*tert*-butyl)-2-fluorobut-3-enamide

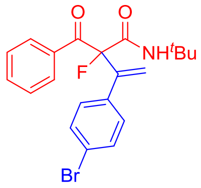

9jt

colorless oil; 35% yield; 87:13 e.r.; R_f = 0.45 (petroleum ether/ethyl acetate = 6/1); $[\alpha]^{25}_D$ = +19.3 (*c* = 0.28, in DCM).
HPLC (Chiralcel **IA**, hexane/*i*-PrOH = 90/10, flow rate 1.0 mL/min, λ = 254 nm) t_r (minor) = 7.14 min, t_r (major) = 8.95 min.
IR (neat): 3425, 3368, 2968, 2926, 1701, 1684, 1516, 1488, 1451, 1394, 1366, 1219, 1186, 1072, 1010, 842 cm^{-1}.
^{1}H NMR (400 MHz, $CDCl_3$) δ 7.96 (dt, *J* = 8.6, 1.2 Hz, 2H), 7.56 – 7.52 (m, 1H), 7.42 – 7.39 (m, 4H), 7.23 – 7.20 (m, 2H), 6.36 (s, 1H), 5.70 (s, 1H), 5.64 (d, *J* = 3.6 Hz, 1H), 1.40 (s, 9H).
^{13}C{^{1}H} NMR (101 MHz, $CDCl_3$) δ 192.5 (d, $J_{C\text{-}F}$ = 25.6), 164.0 (d, $J_{C\text{-}F}$ = 21.4), 143.1 (d, $J_{C\text{-}F}$ = 20.1), 136.3, 134.0 (d, $J_{C\text{-}F}$ = 2.8), 133.7, 131.4, 130.2 (d, $J_{C\text{-}F}$ = 4.2), 129.7 (d, $J_{C\text{-}F}$ = 1.5), 128.3, 122.5, 122.0 (d, $J_{C\text{-}F}$ = 7.3), 100.8 (d, $J_{C\text{-}F}$ = 197.0), 52.2, 28.5.
^{19}F{^{1}H} NMR (376 MHz, $CDCl_3$) δ –143.2 (s, 1F).
HRMS (ESI-TOF) calcd for $C_{21}H_{22}{}^{78.9183}BrFNO_2^+$ ($[M]+H^+$) = 418.0812, found 418.0819, and $C_{21}H_{22}{}^{80.9163}BrFNO_2^+$ ($[M]+H^+$) = 420.0792, found 420.0800.
Chiral HPLC spectrum **9jt**:

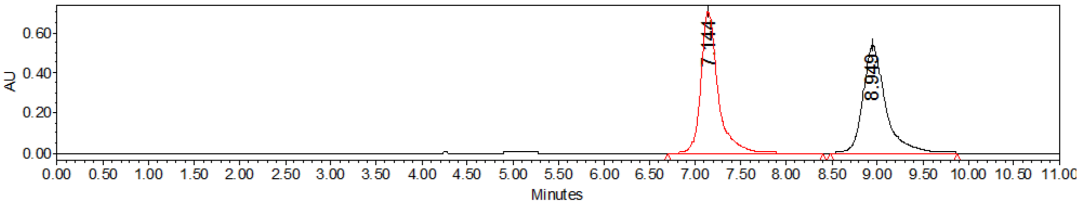

Peak	Retention Time	Area	% Area
1	7.144	9068199	50.12
2	8.949	9025756	49.88

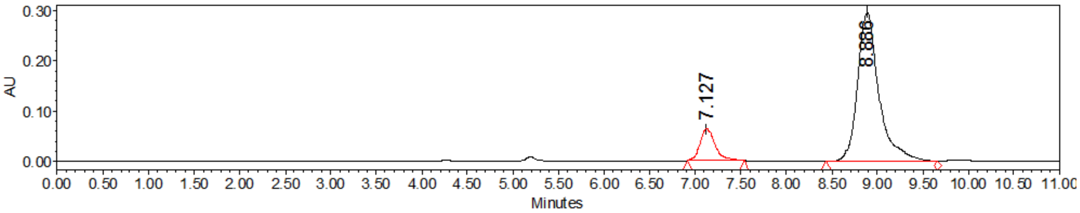

Peak	Retention Time	Area	% Area
1	7.127	731994	13.14
2	8.886	4837127	86.86

(1*S*,2*R*)-*N*-(*tert*-Butyl)-1-hydroxy-2-(1-phenylvinyl)-2,3-dihydro-1*H*-indene-2-carboxamide

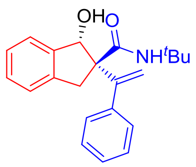

10aa

white soild, m.p.133.3 – 138.7; 90% yield; 94.5:5.5 e.r., d.r. = 92:8; R_f = 0.30 (petroleum ether/ethyl acetate = 4/1); $[\alpha]^{19}_D$ = +105.5 (c = 0.54, in DCM).
HPLC (Chiralcel **IA**, hexane/*i*-PrOH = 95/5, flow rate 1.0 mL/min, λ = 254 nm) t_r (minor-major) = 9.15 min, t_r (minor-minor) = 9.82 min, t_r (major-major) = 13.90, t_r (major-minor) = 22.78 min.
IR (neat): 2278, 2963, 2925, 1655, 1530, 1223, 1069, 1029, 910, 701 cm^{-1}.
^{1}H NMR (400 MHz, $CDCl_3$) δ 7.41 – 7.26 (m, 6H), 7.24 – 7.16 (m, 3H), 5.78 (s, 1H), 5.61 – 5.60 (m, 1H), 5.52 (s, 1H), 5.40 (s, 1H), 3.59 (dd, *J* = 15.6, 2.8 Hz, 1H), 3.37 (d, *J* = 15.6 Hz, 1H), 1.94 (d, *J* = 7.2 Hz, 1H), 1.16 (s, 9H).
^{13}C{^{1}H} NMR (101 MHz, $CDCl_3$) δ 173.0, 150.2, 143.8, 141.2, 140.1, 128.5, 128.4, 127.9, 127.3, 127.2, 124.3, 124.2, 118.2, 78.6, 65.2, 51.0, 42.2, 28.3.
HRMS (ESI-TOF) calcd for $C_{22}H_{25}NNaO_2^+$ ([M]+Na^+) = 358.1777, found 358.1770.
Chiral HPLC spectrum **10aa**:

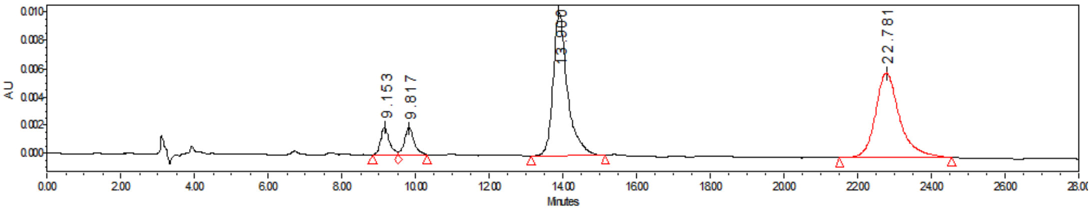

Peak	Retention Time	Area	% Area
1	9.153	33037	5.37
2	9.817	34195	5.56
3	13.900	274845	44.65
4	22.781	273457	44.43

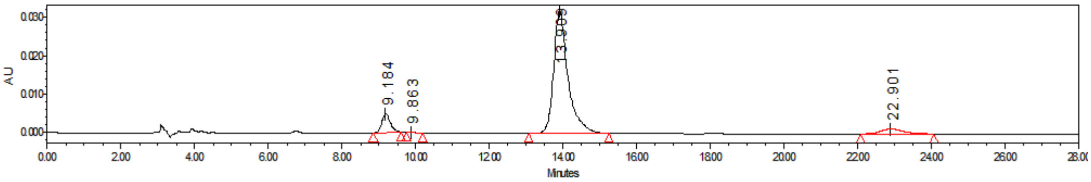

Peak	Retention Time	Area	% Area
1	9.184	79819	7.96
2	9.863	1520	0.15
3	13.909	861803	85.99
4	22.901	59104	5.90

(*R*)-*N*-Isopropyl-5-oxo-6-(1-phenylvinyl)-6,7,8,9-tetrahydro-5*H*-benzo[7]annulene-6-carboxamide

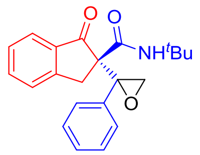

11aa

colorless oil; 98% yield; 94.5:5.5 e.r., d.r. = 90:10; R_f = 0.30 (petroleum ether/ethyl acetate = 10/1); $[\alpha]^{19}_D$ = –178.6 (c = 0.48, in DCM).
HPLC (Chiralcel **IA**, hexane/*i*-PrOH = 90/10, flow rate 1.0 mL/min, λ = 254 nm) t_r (minor-major) = 6.08 min, t_r (minor-major) = 7.36 min, t_r (major-major) = 7.76, t_r (major-minor) =13.2 min.
IR (neat): 2965, 2927, 1701, 1681, 1537, 1271, 1222, 769, 755, 700 cm^{-1}.
^{1}H NMR (400 MHz, $CDCl_3$) δ 7.72 (d, J = 7.6 Hz, 1H), 7.61 (td, J = 7.2, 1.2 Hz, 1H), 7.45 (dt, J = 8.0, 1.2 Hz, 1H), 7.40 – 7.35 (m, 3H), 7.32 – 7.28 (m, 3H), 7.13 (s, 1H), 4.09 (d, J = 18.0 Hz, 1H), 3.34 (d, J = 18.0 Hz, 1H), 3.00 (d, J = 4.8 Hz, 1H), 2.80 (d, J = 4.4 Hz, 1H), 1.31 (s, 9H).
^{13}C{^{1}H} NMR (101 MHz, $CDCl_3$) δ 201.2, 165.1, 153.2, 137.0, 135.9, 135.2, 128.4, 128.0, 127.7, 127.7, 126.4, 124.5, 63.6, 63.0, 51.8, 51.6, 34.8, 28.5.
HRMS (ESI-TOF) calcd for $C_{22}H_{23}NNaO_2^+$ ($[M+Na]^+$) = 372.1570, found 372.1570.
Chiral HPLC spectrum **11aa**:

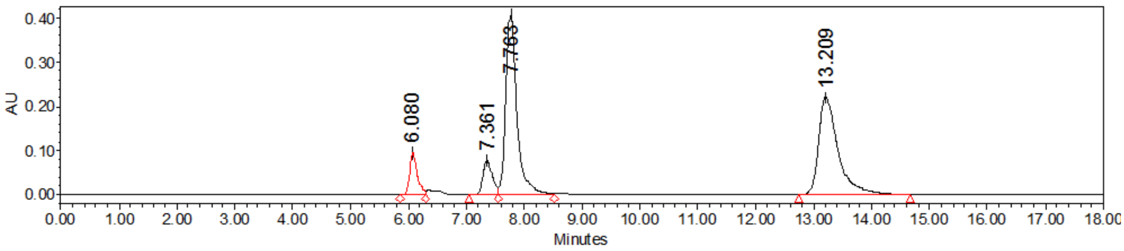

Peak	Retention Time	Area	% Area
1	6.080	902367	7.36
2	7.361	857825	6.99
3	7.763	5329033	43.44
4	13.209	5177618	42.21

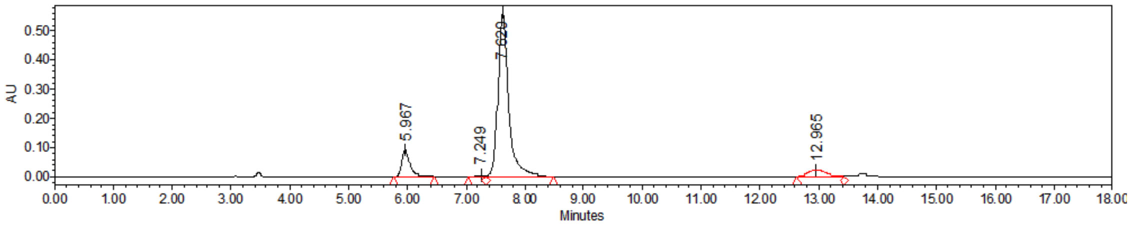

Peak	Retention Time	Area	% Area
1	5.967	864323	10.15
2	7.249	49138	0.43
3	7.620	7081826	83.96
4	12.965	439412	5.21

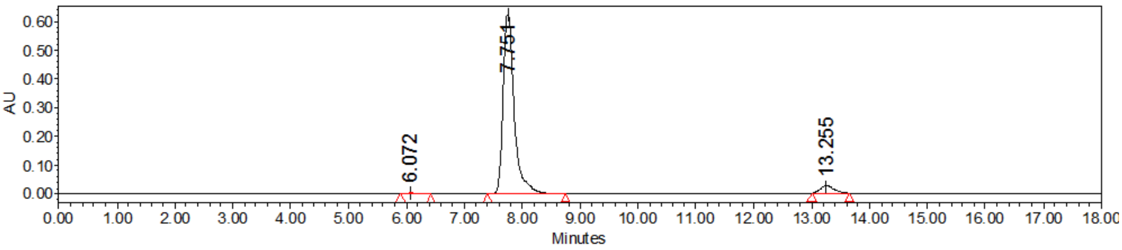

Peak	Retention Time	Area	% Area
1	6.072	49360	0.58
3	7.751	8085940	94.58
4	13.255	413809	4.84

NMR spectral characterization data of byproducts

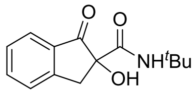

3bb

^{1}H NMR (400 MHz, $CDCl_3$) δ 7.76 (d, *J* = 7.6 Hz, 1H), 7.66 – 7.62 (m, 1H), 7.47 (d, *J* = 8.0 Hz, 1H), 7.39 (t, *J* = 7.6 Hz, 1H), 6.71 (s, 1H), 3.77 – 3.69 (m, 2H), 3.08 (d, *J* = 16.8 Hz, 1H), 1.33 (s, 9H).
^{13}C{^{1}H} NMR (101 MHz, $CDCl_3$) δ 203.8, 169.5, 153.1, 136.2, 134.0, 128.0, 126.4, 125.1, 82.3, 51.4, 40.8, 28.6.
HRMS (ESI-TOF) calcd for $C_{14}H_{17}NNaO_3^+$ ([M]+Na^+) = 270.1100, found 270.1096.

8. Copies of NMR spectra for products

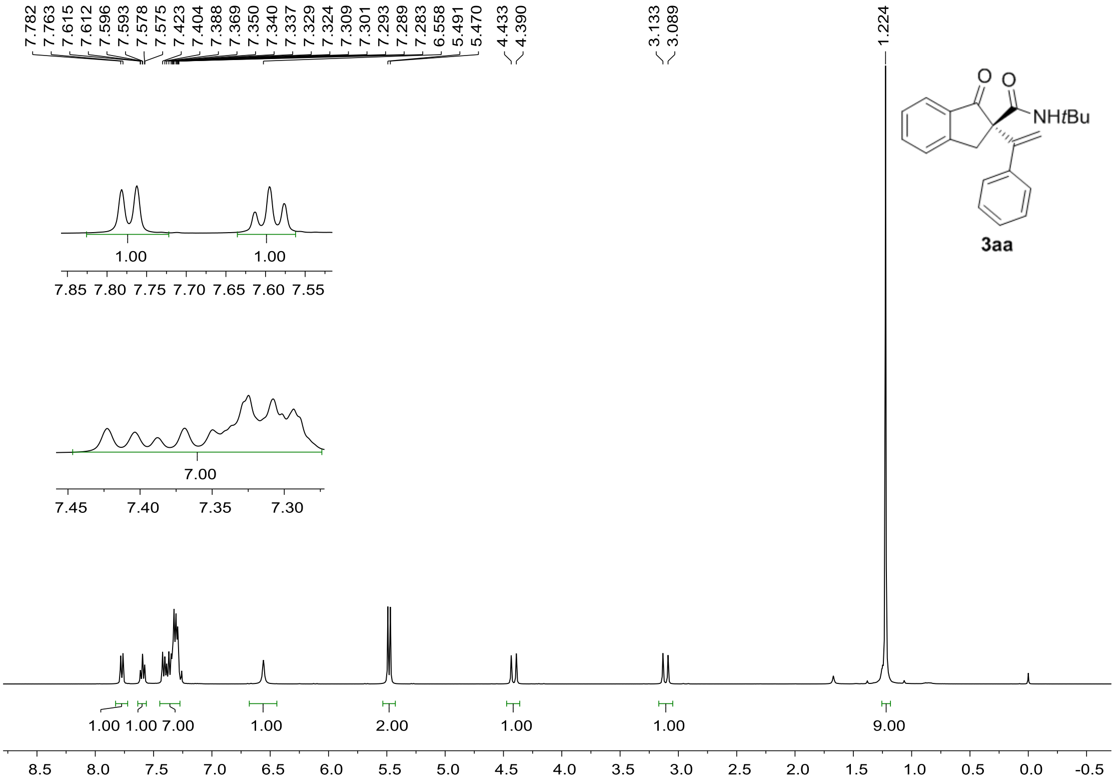

Supplementary Figure 6. ^{1}H NMR spectra for product **3aa**

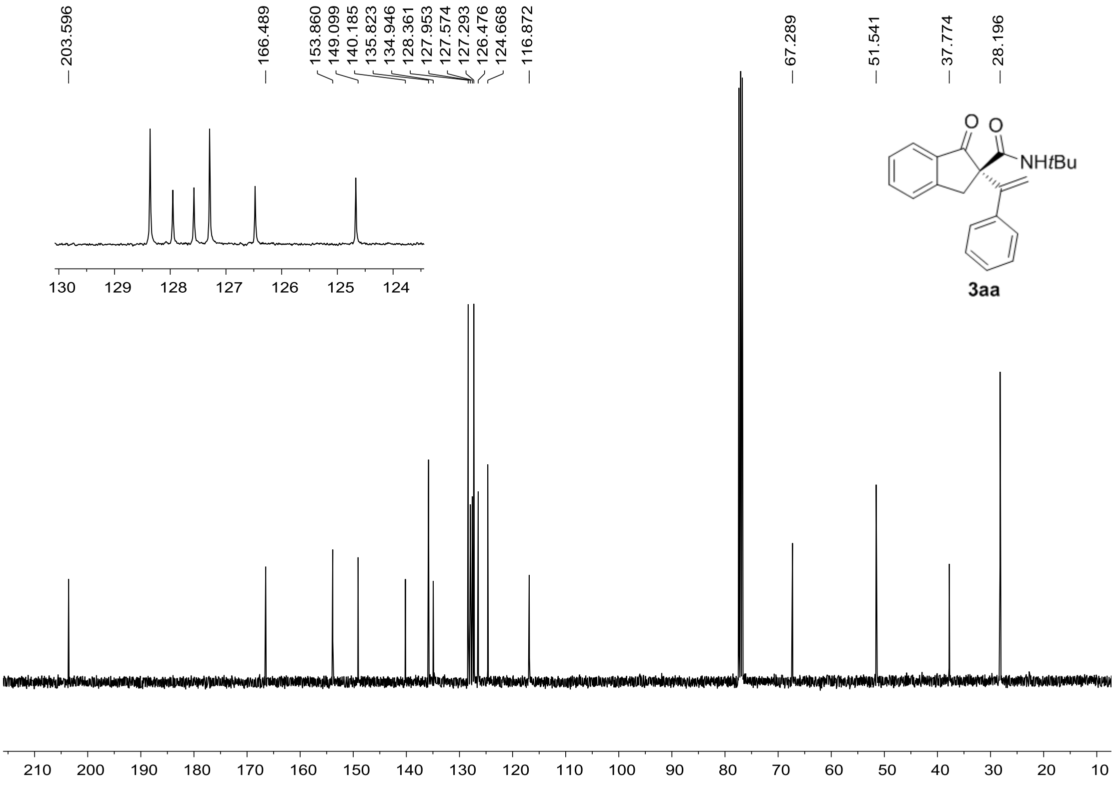

Supplementary Figure 7. ^{13}C NMR spectra for product **3aa**

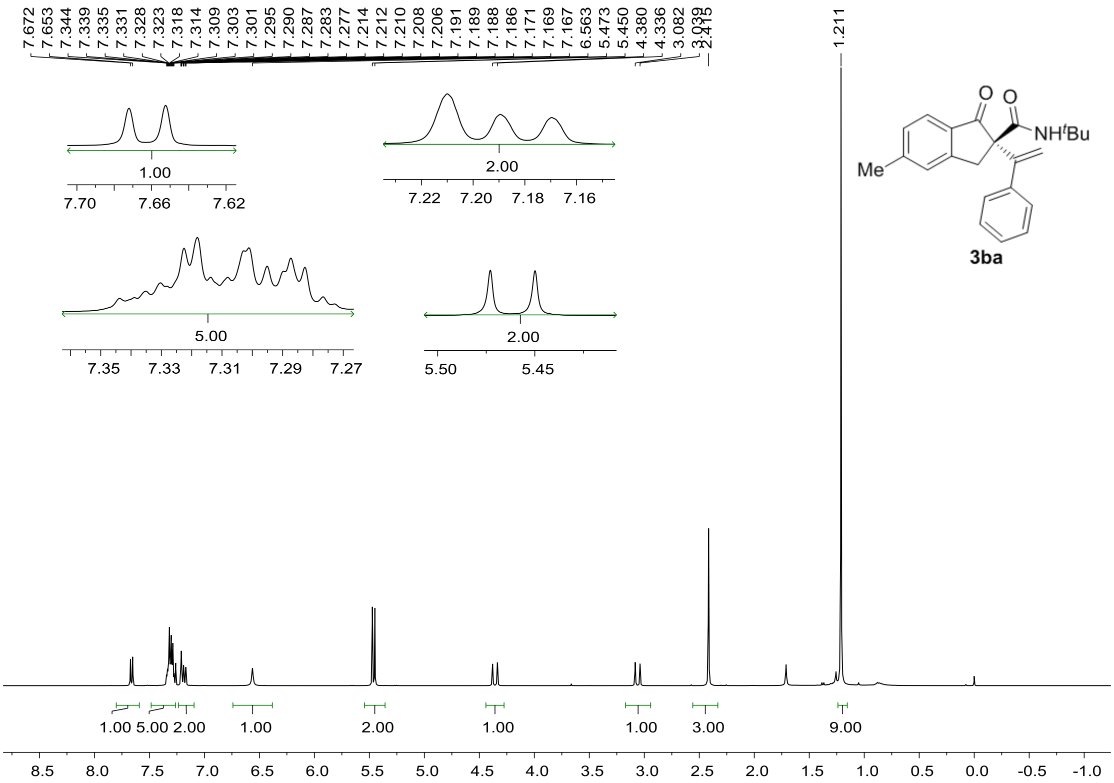

Supplementary Figure 8. ^{1}H NMR spectra for product **3ba**

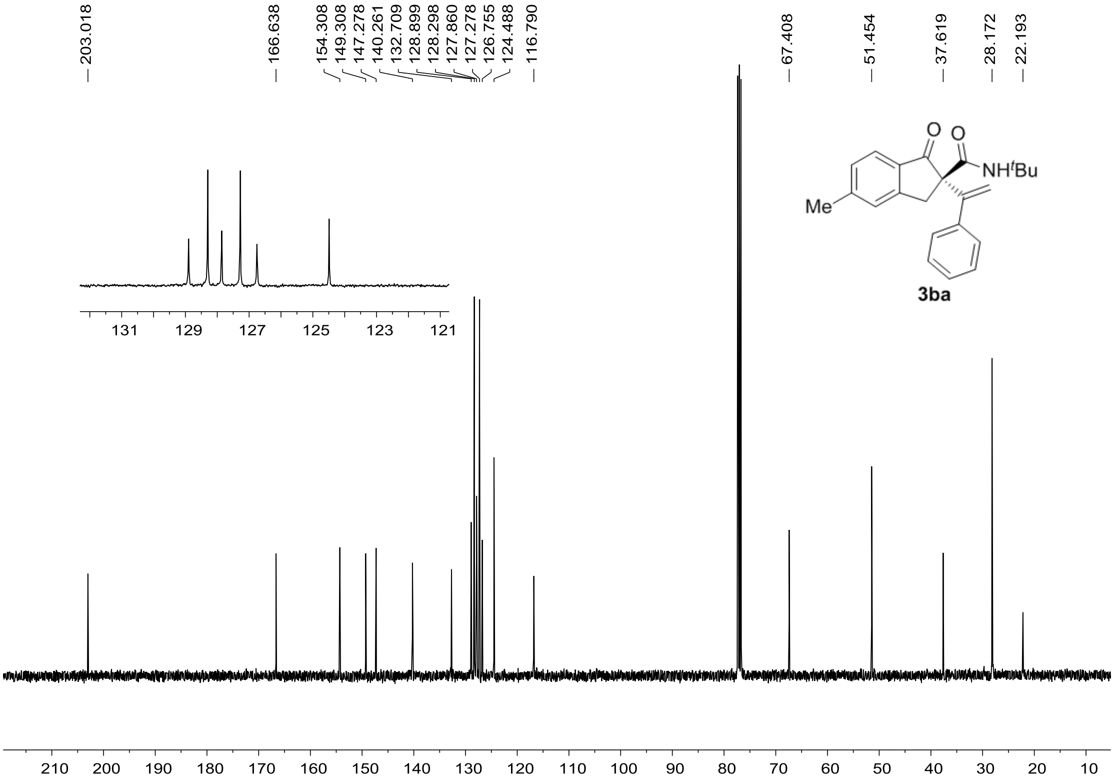

Supplementary Figure 9. ^{13}C NMR spectra for product **3ba**

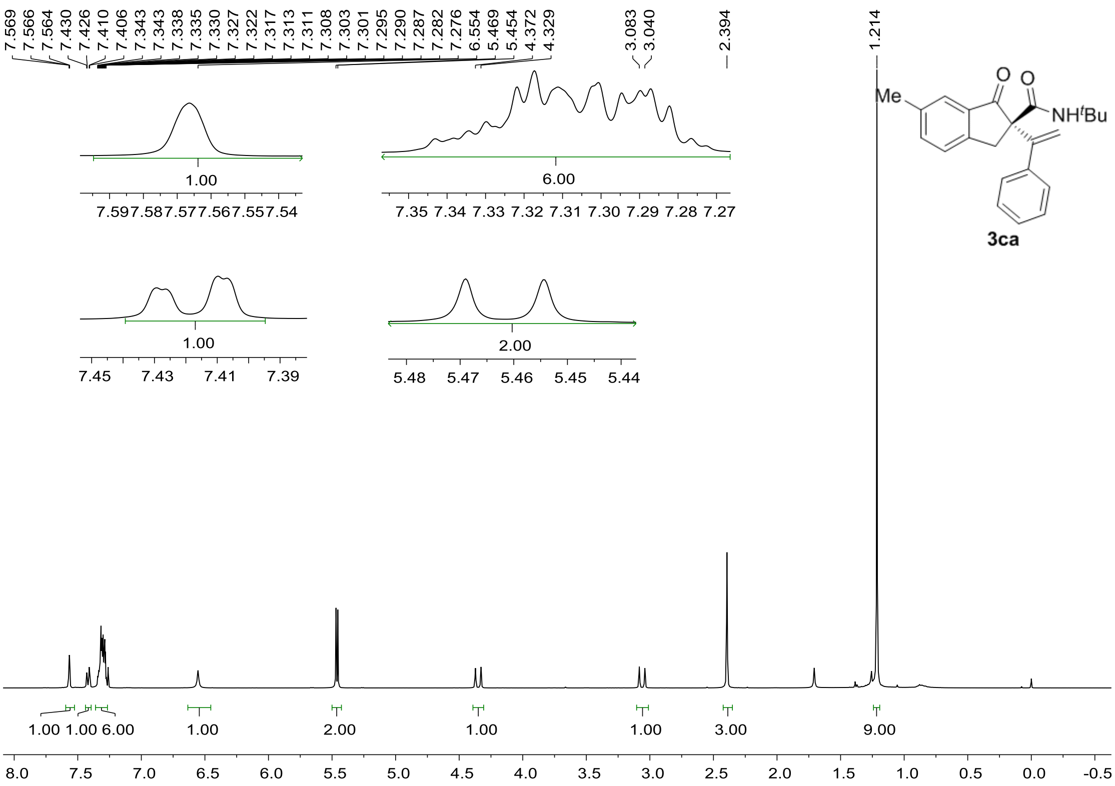

Supplementary Figure 10. ^{1}H NMR spectra for product **3ca**

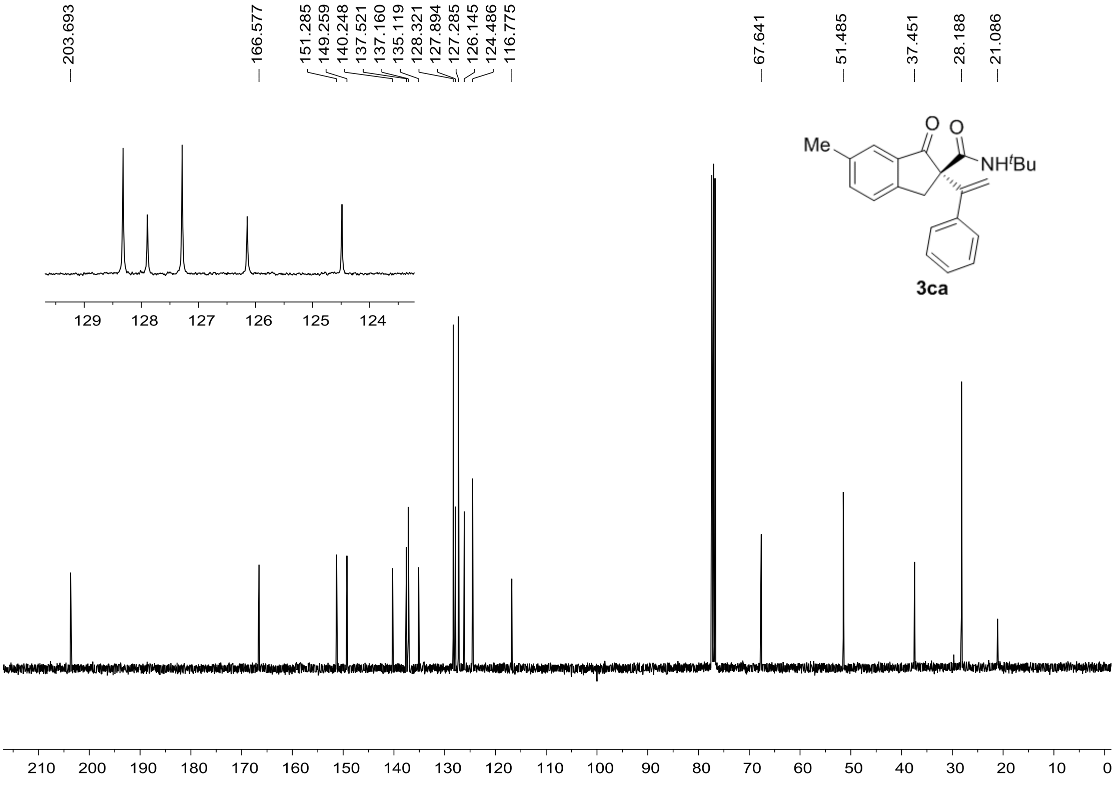

Supplementary Figure 11. ^{13}C NMR spectra for product **3ca**

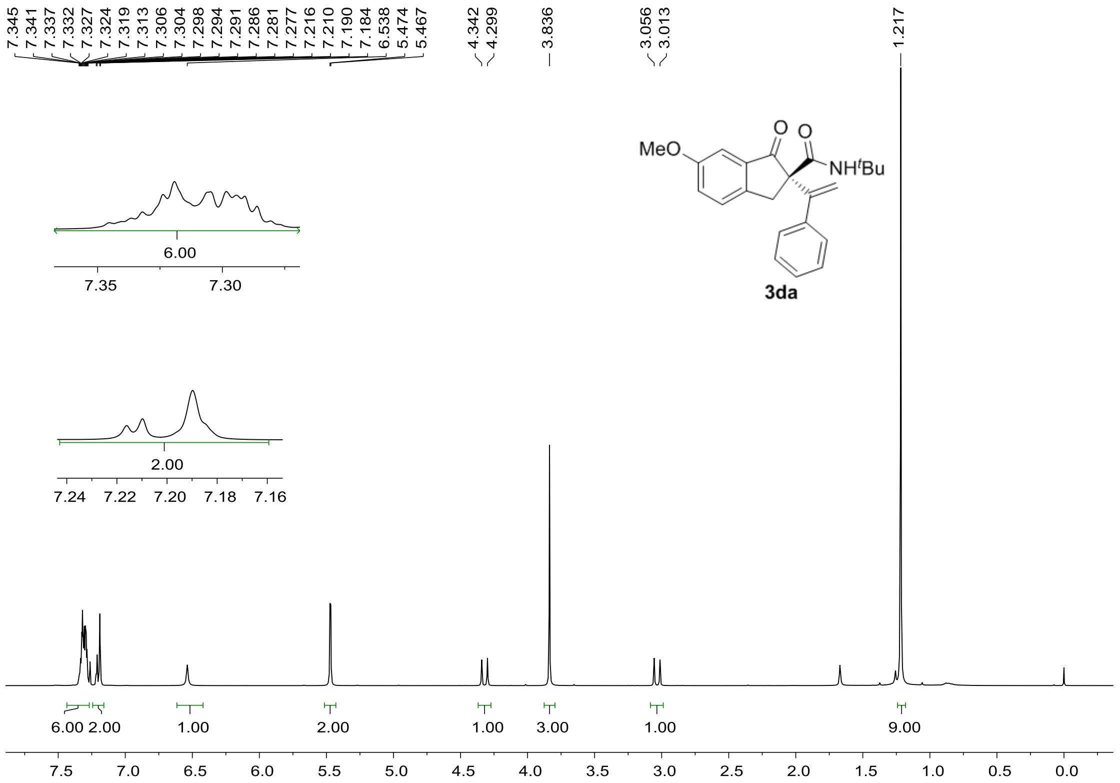

Supplementary Figure 12. ^{1}H NMR spectra for product **3da**

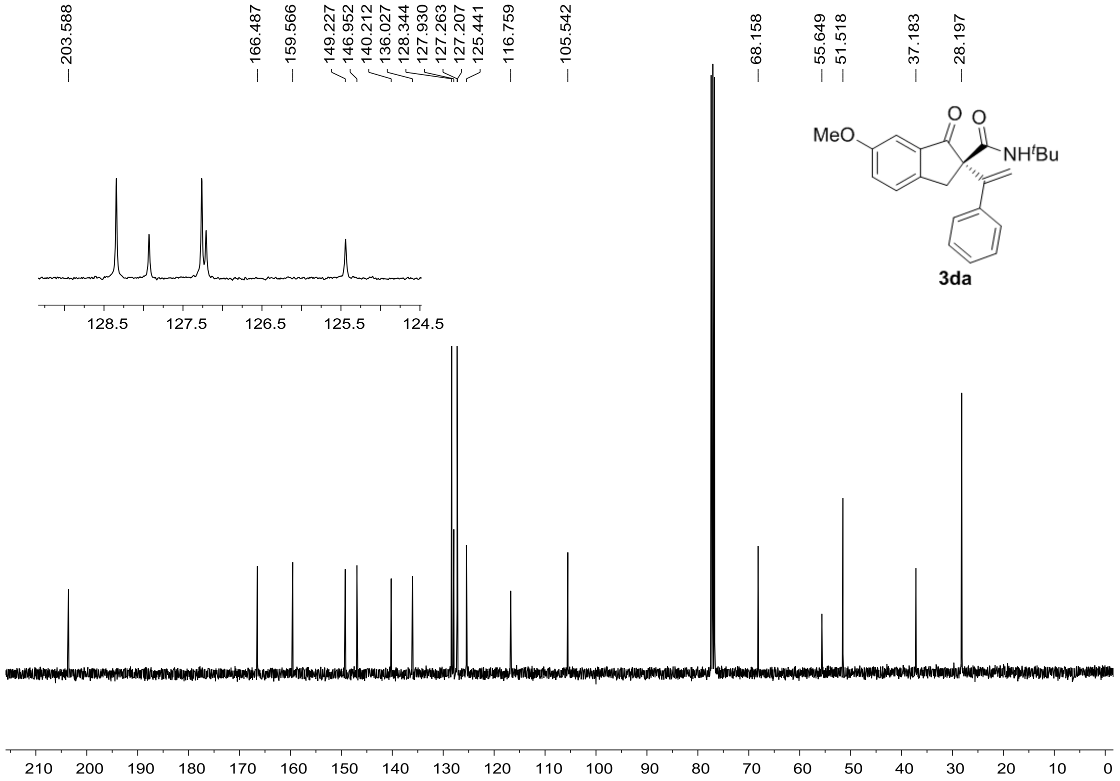

Supplementary Figure 13. ^{13}C NMR spectra for product **3da**

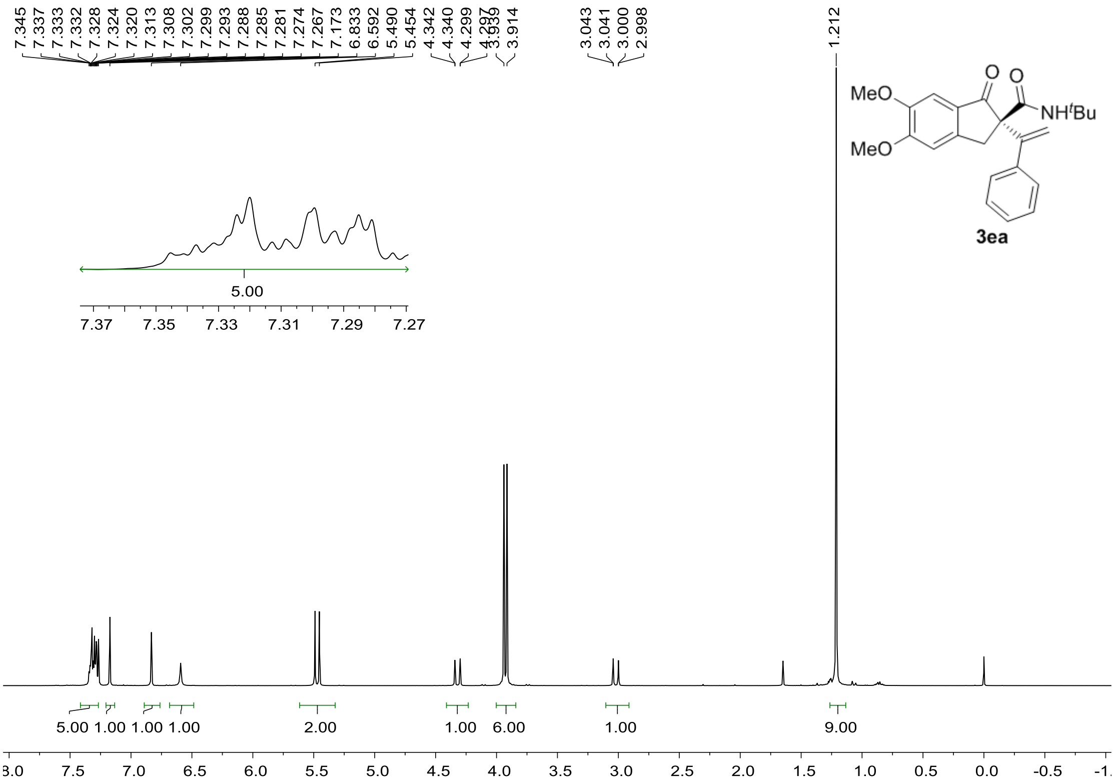

Supplementary Figure 14. ^{1}H NMR spectra for product **3ea**

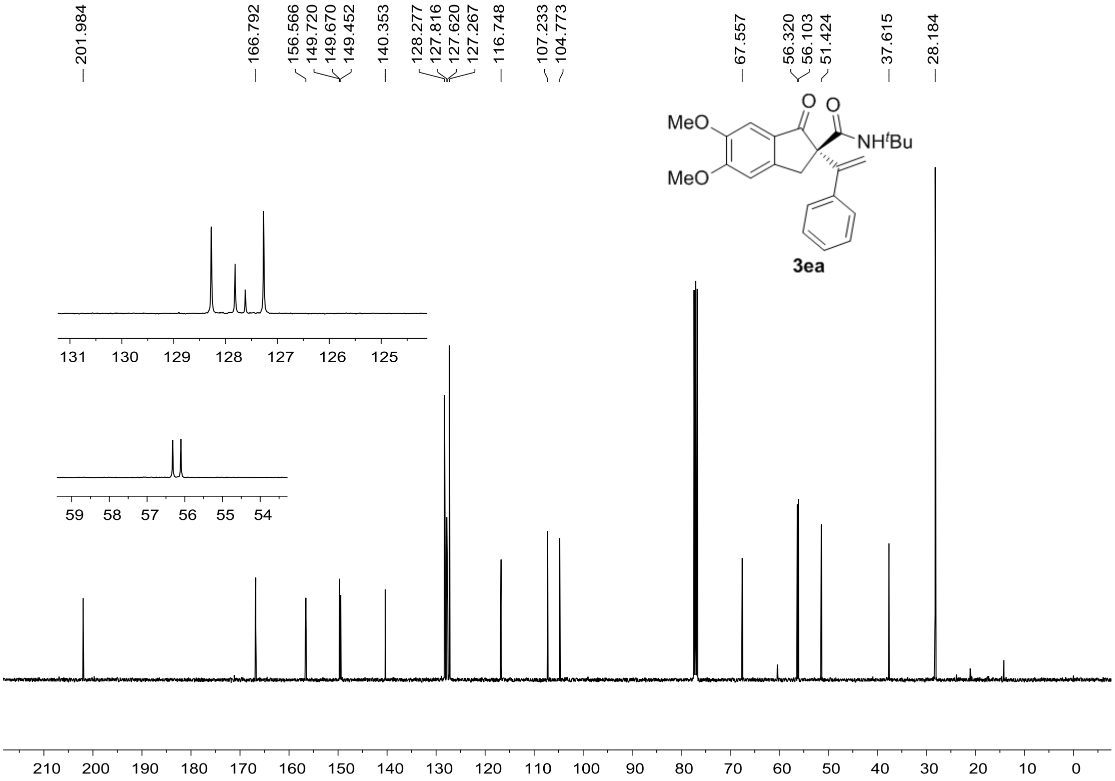

Supplementary Figure 15. ^{13}C NMR spectra for product **3ea**

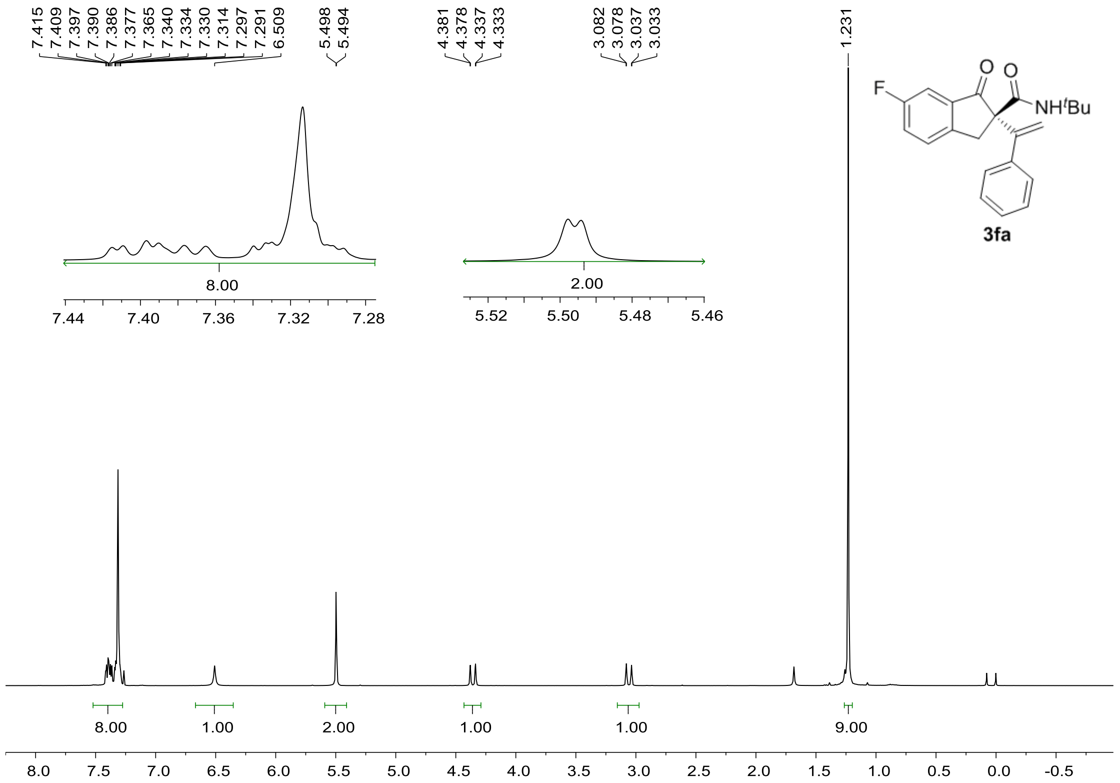

Supplementary Figure 16. ^{1}H NMR spectra for product **3fa**

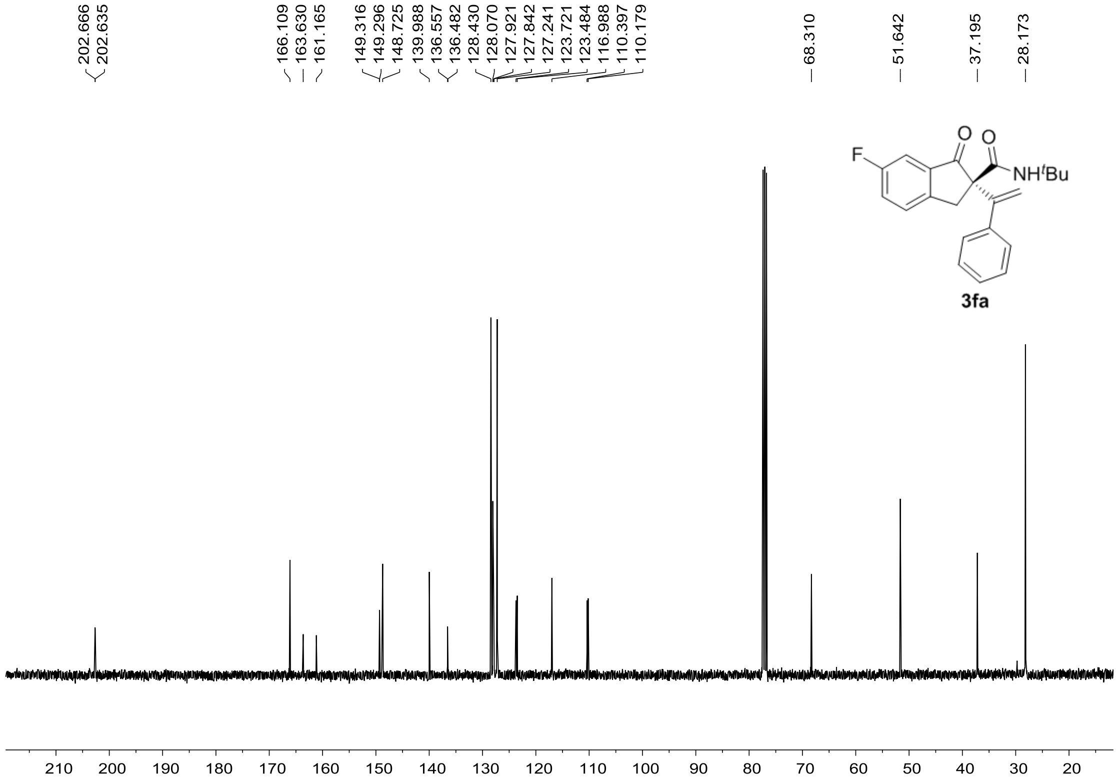

Supplementary Figure 17. ^{13}C NMR spectra for product **3fa**

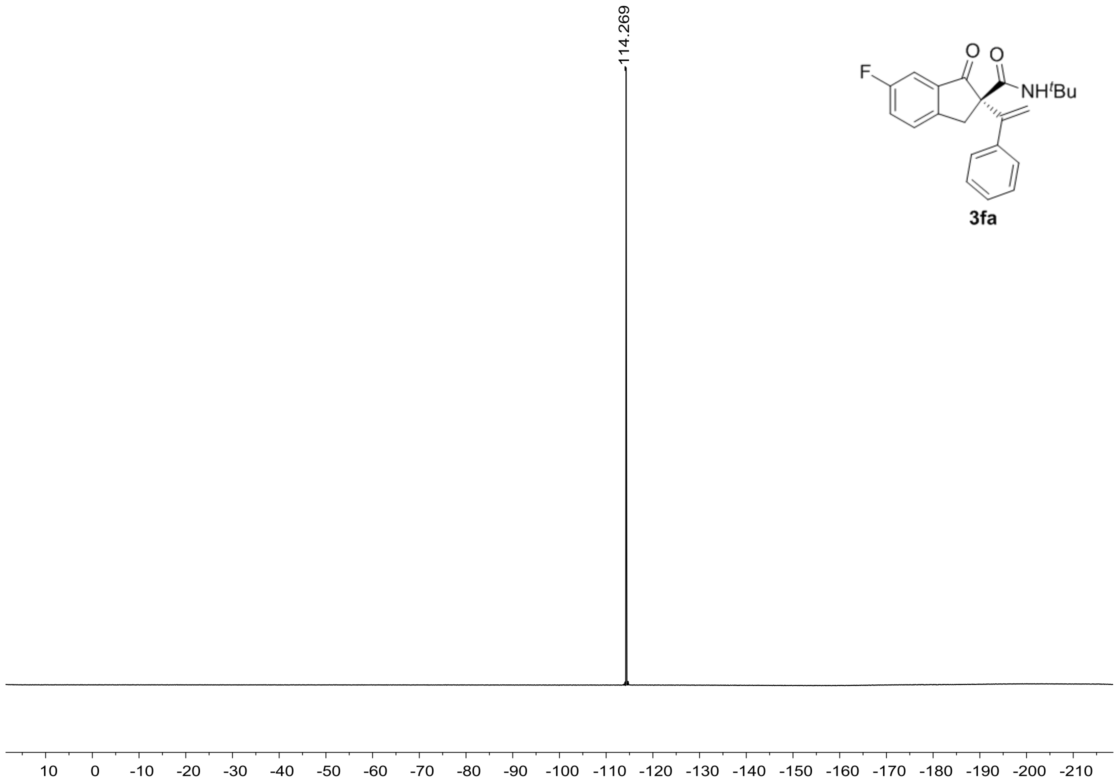

Supplementary Figure 18. ^{19}F NMR spectra for product **3fa**

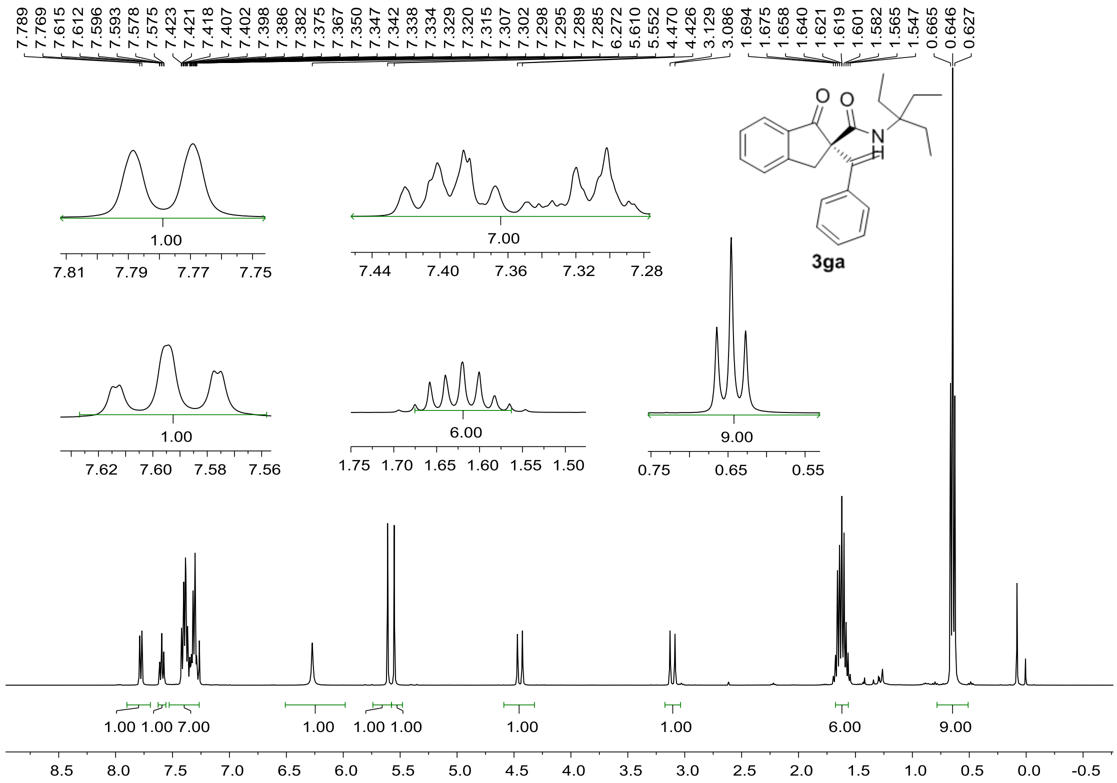

Supplementary Figure 19. ^{1}H NMR spectra for product **3ga**

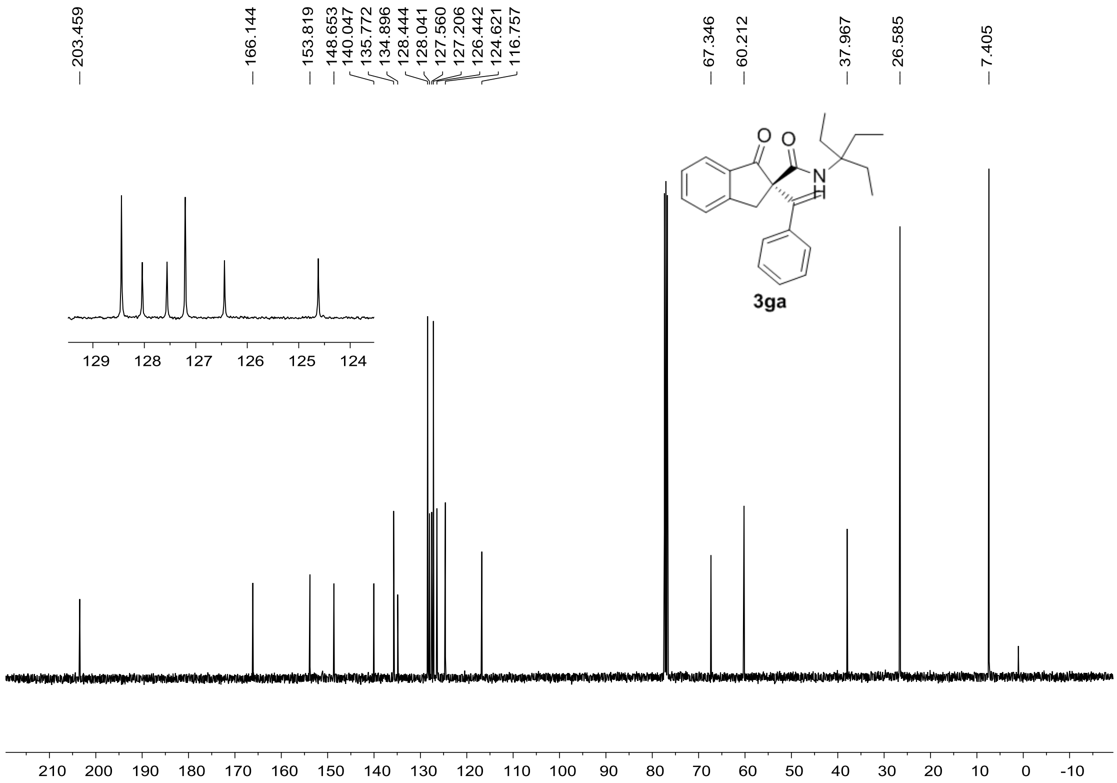

Supplementary Figure 20. ^{13}C NMR spectra for product **3ga**

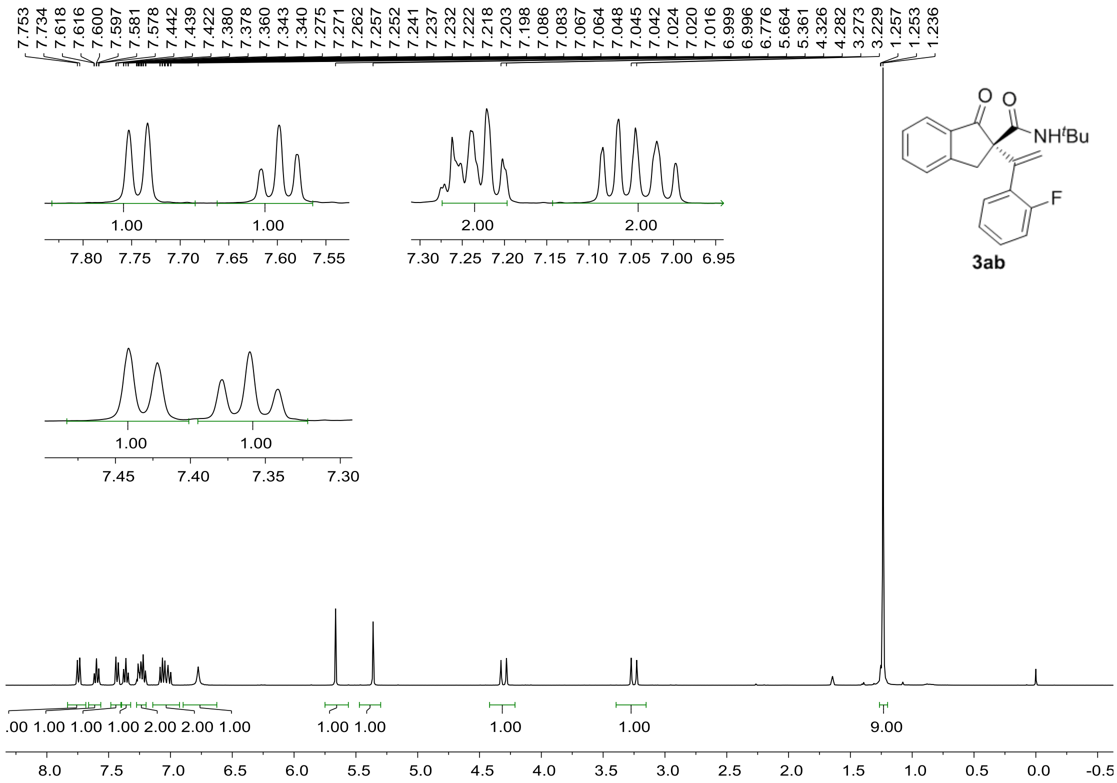

Supplementary Figure 21. ^{1}H NMR spectra for product **3ab**

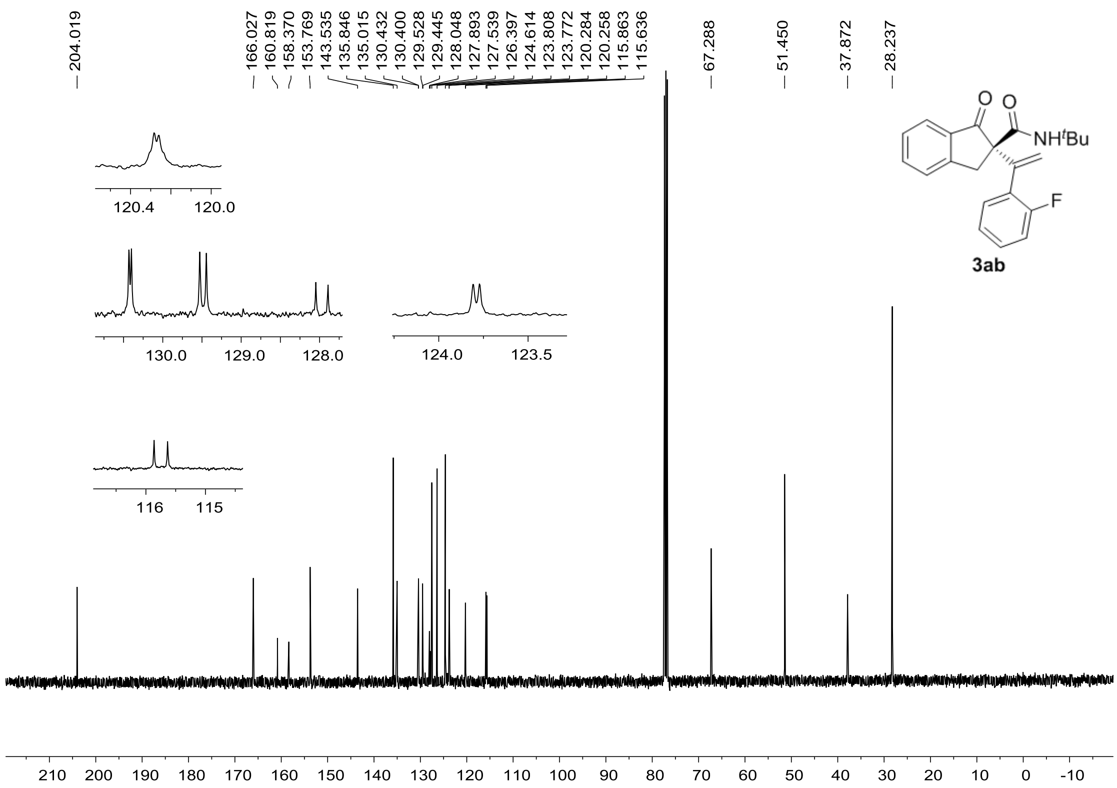

Supplementary Figure 22. ^{13}C NMR spectra for product **3ab**

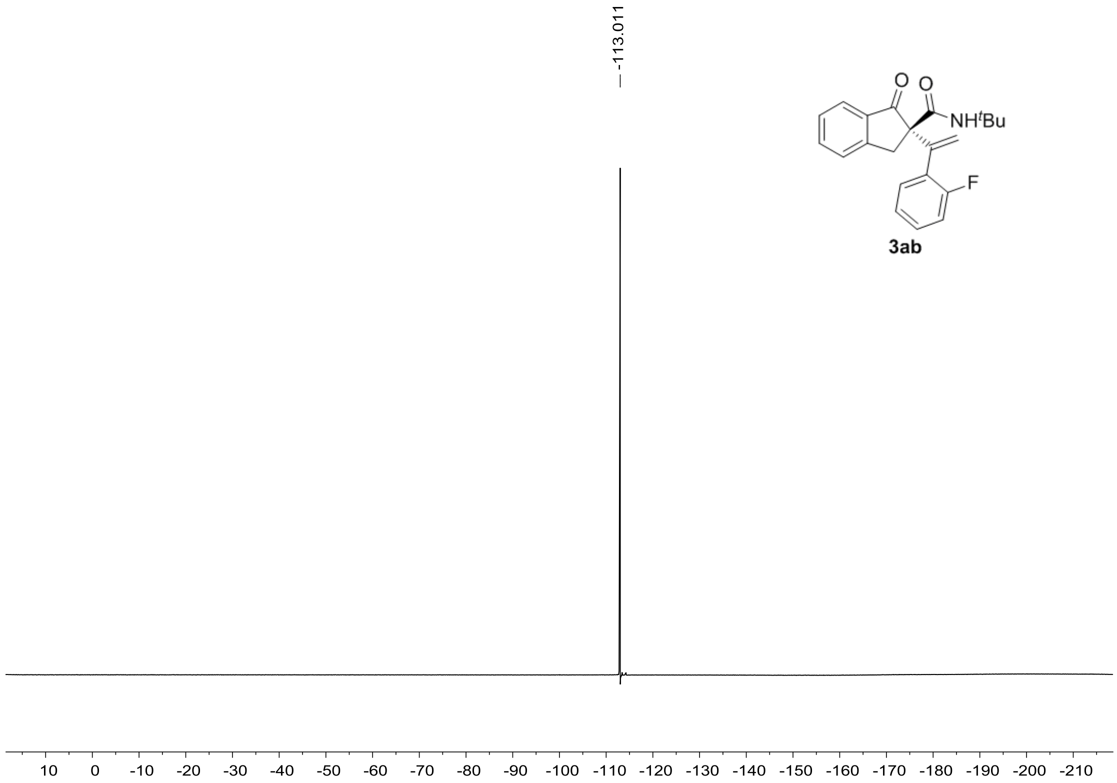

Supplementary Figure 23. ^{19}F NMR spectra for product **3ab**

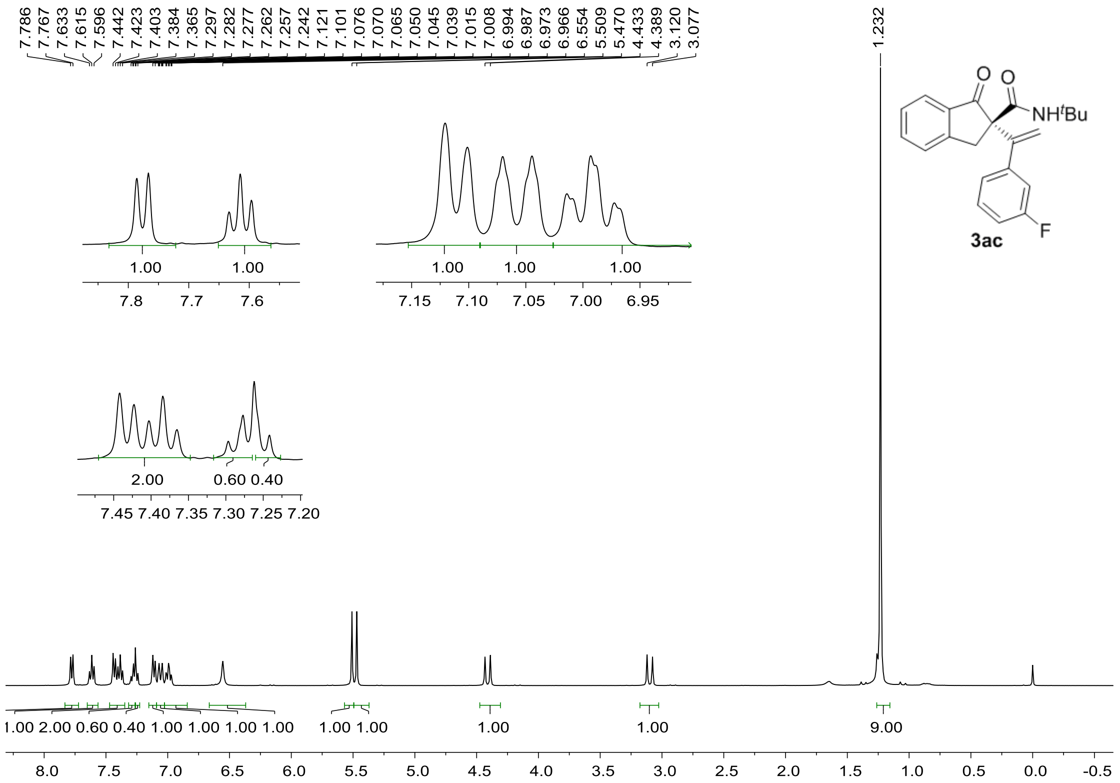

Supplementary Figure 24. ^{1}H NMR spectra for product 3**ac**

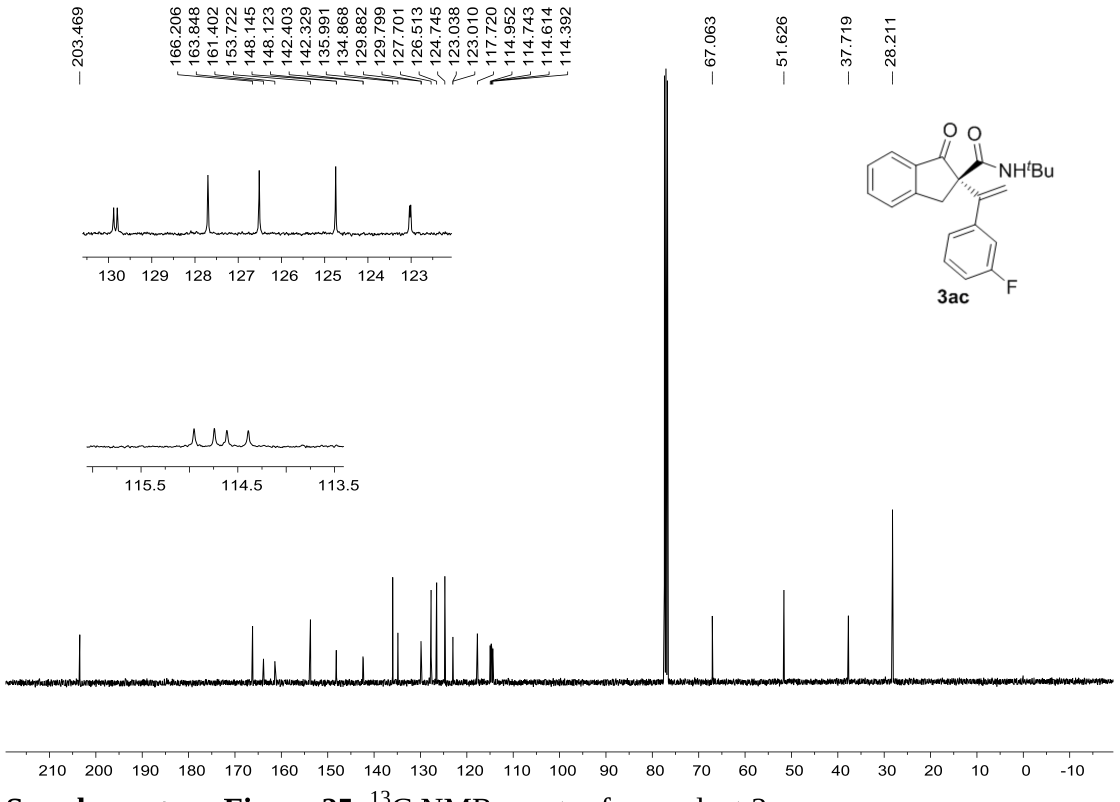

Supplementary Figure 25. ^{13}C NMR spectra for product 3**ac**

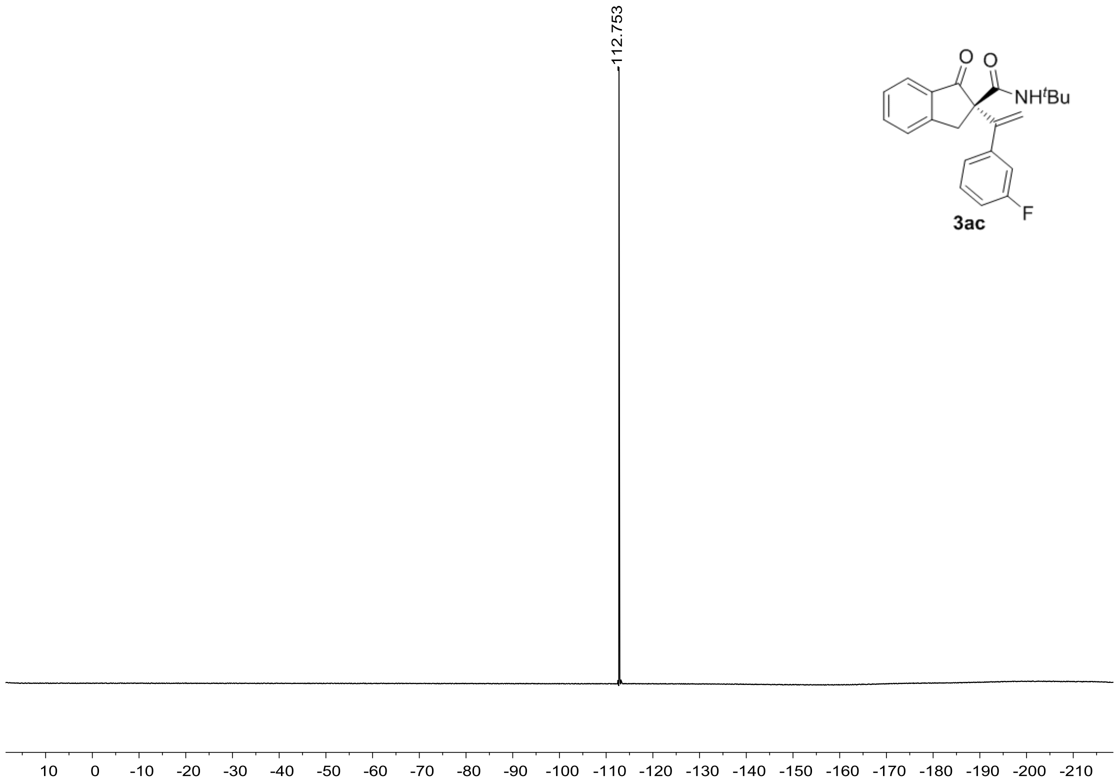

Supplementary Figure 26. ^{19}F NMR spectra for product **3ac**

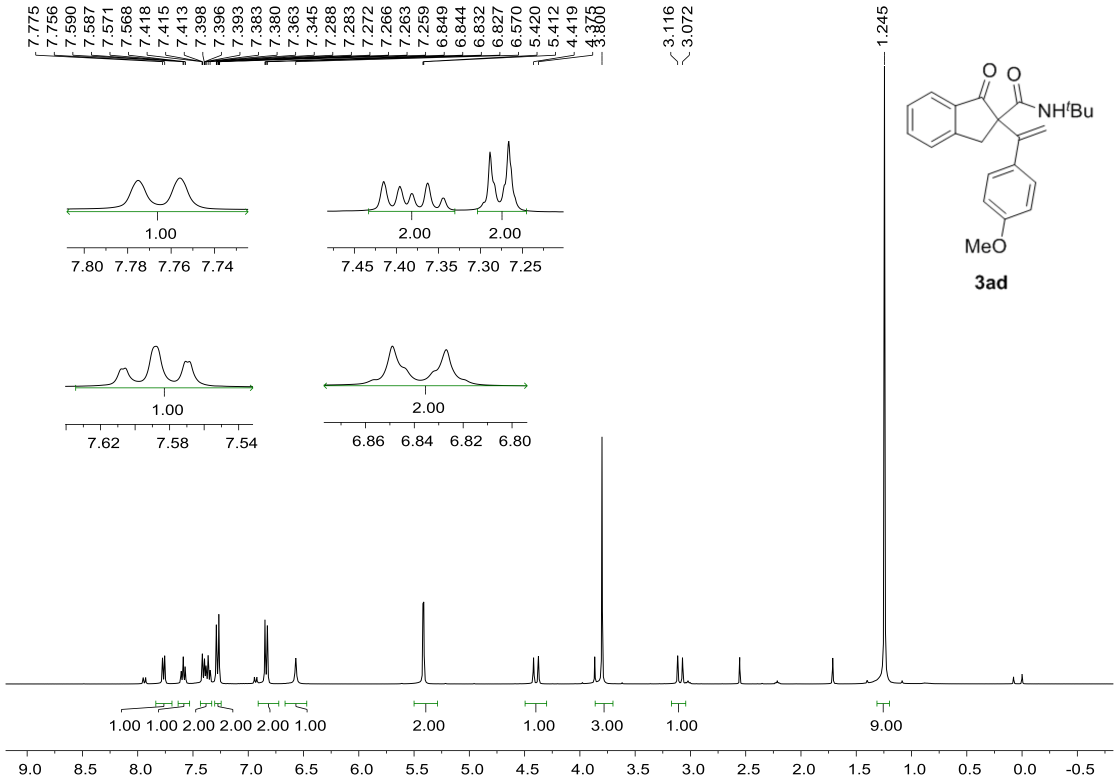

Supplementary Figure 27. ^{1}H NMR spectra for product **3ad**

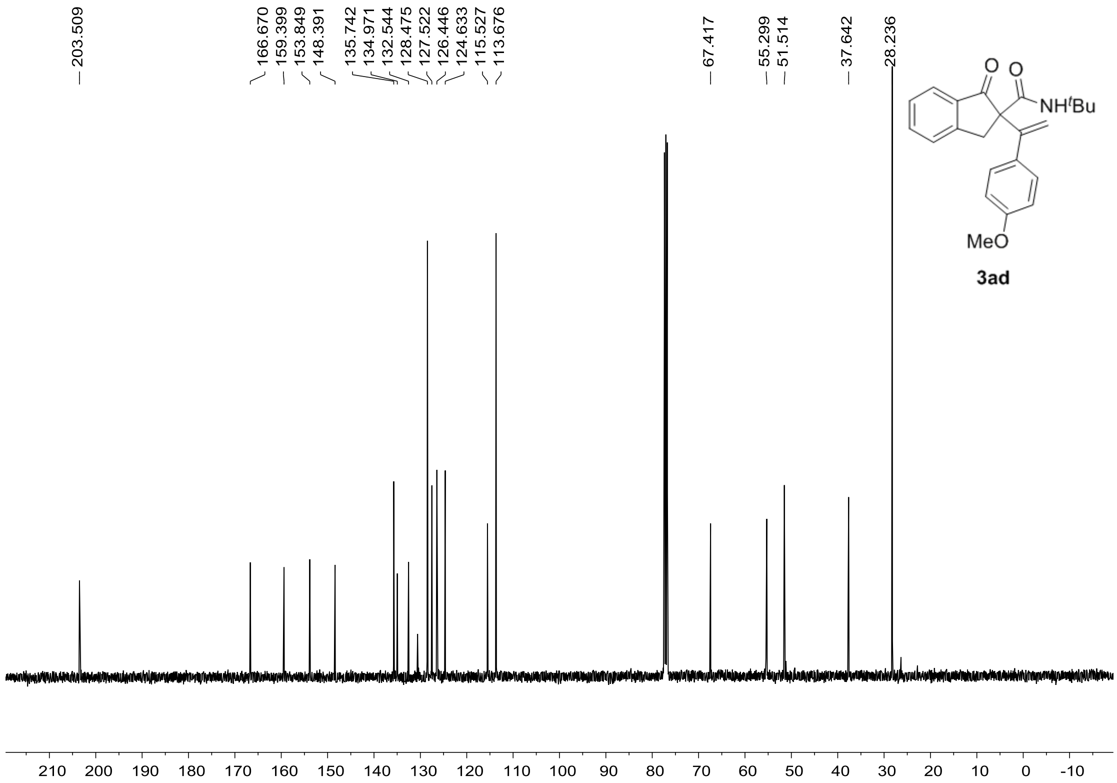

Supplementary Figure 28. ^{13}C NMR spectra for product **3ad**

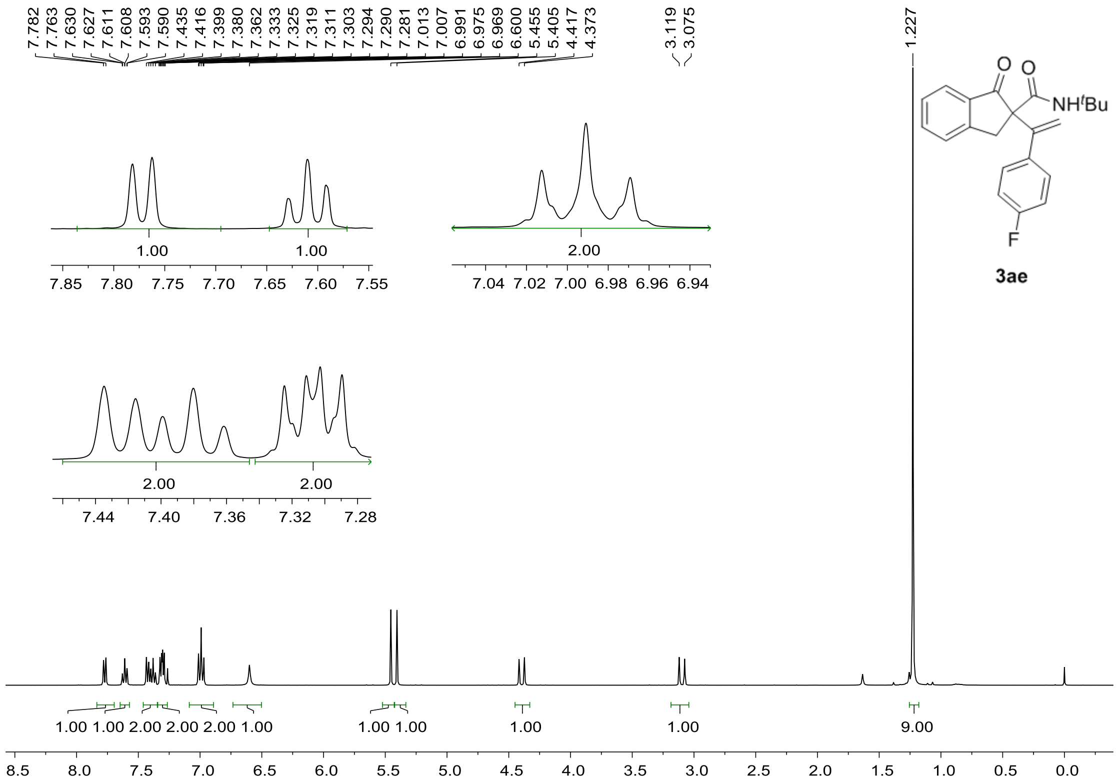

Supplementary Figure 29. ^{1}H NMR spectra for product **3ae**

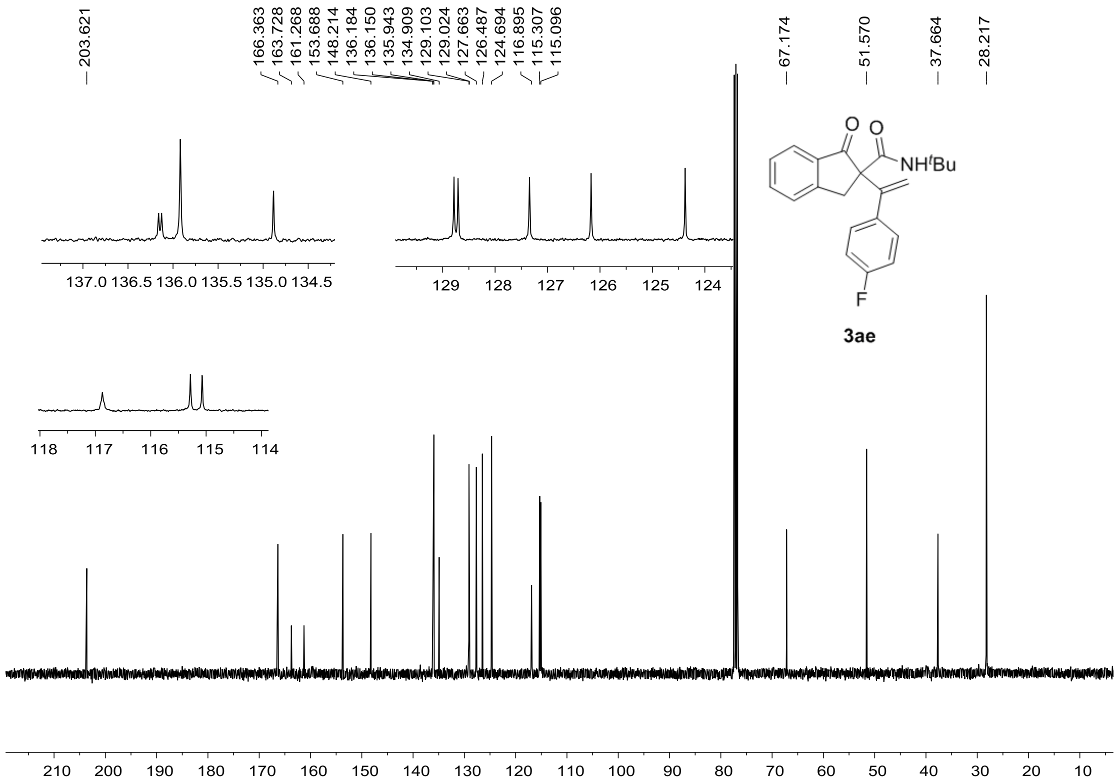

Supplementary Figure 30. ^{13}C NMR spectra for product **3ae**

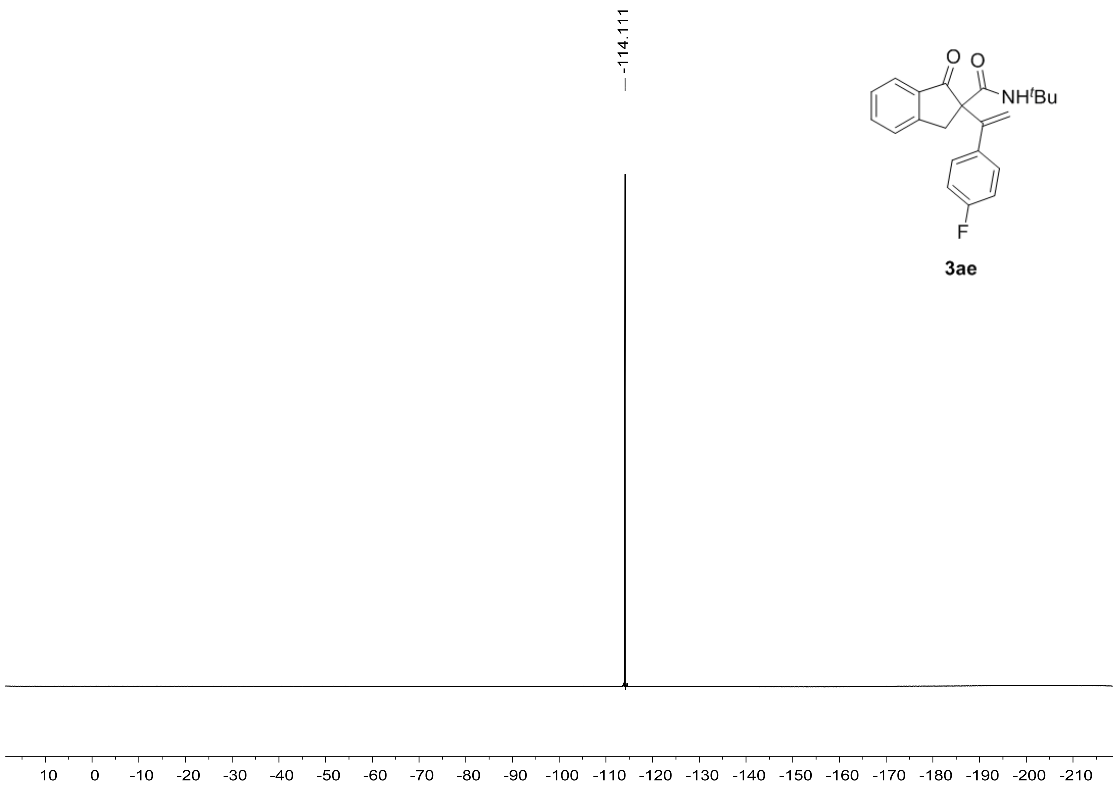

Supplementary Figure 31. ^{19}F NMR spectra for product **3ae**

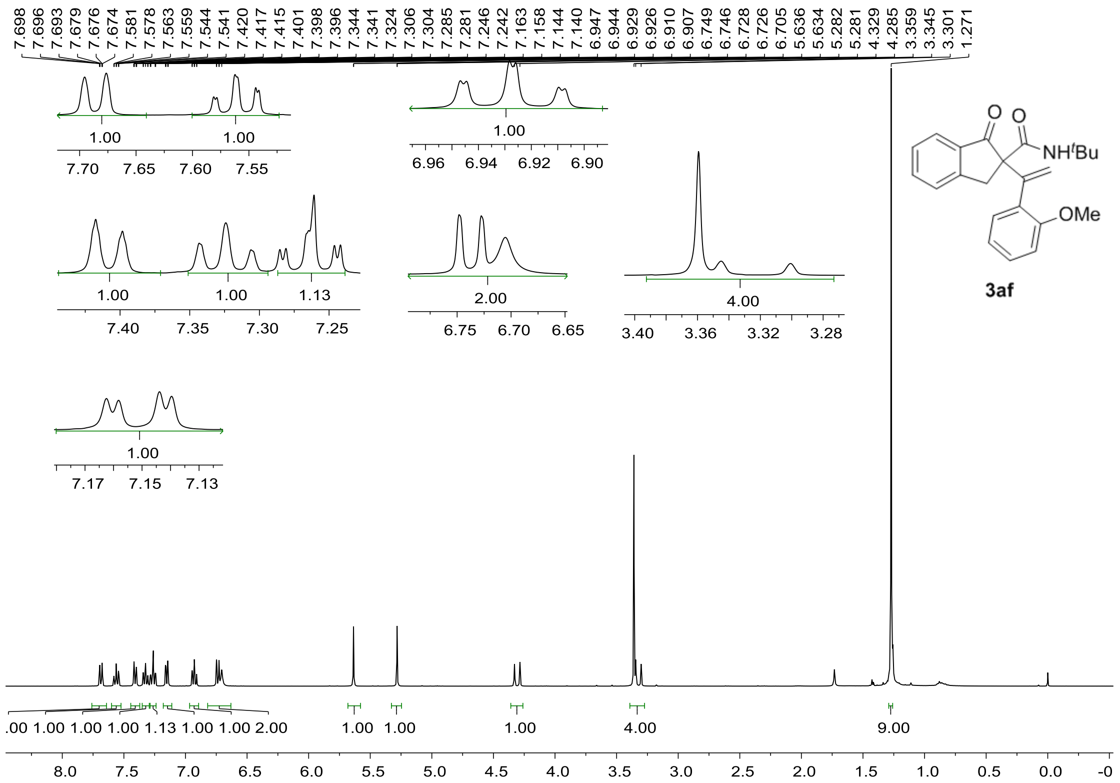

Supplementary Figure 32. ^{1}H NMR spectra for product **3af**

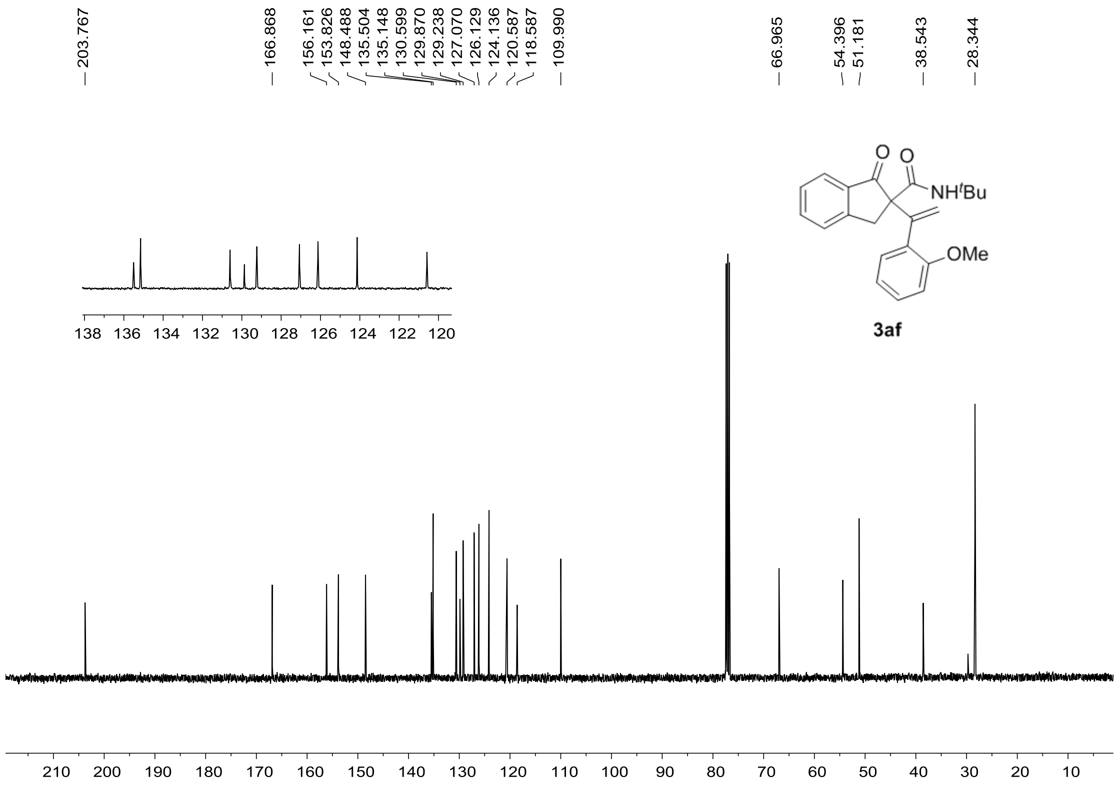

Supplementary Figure 33. ^{13}C NMR spectra for product **3af**

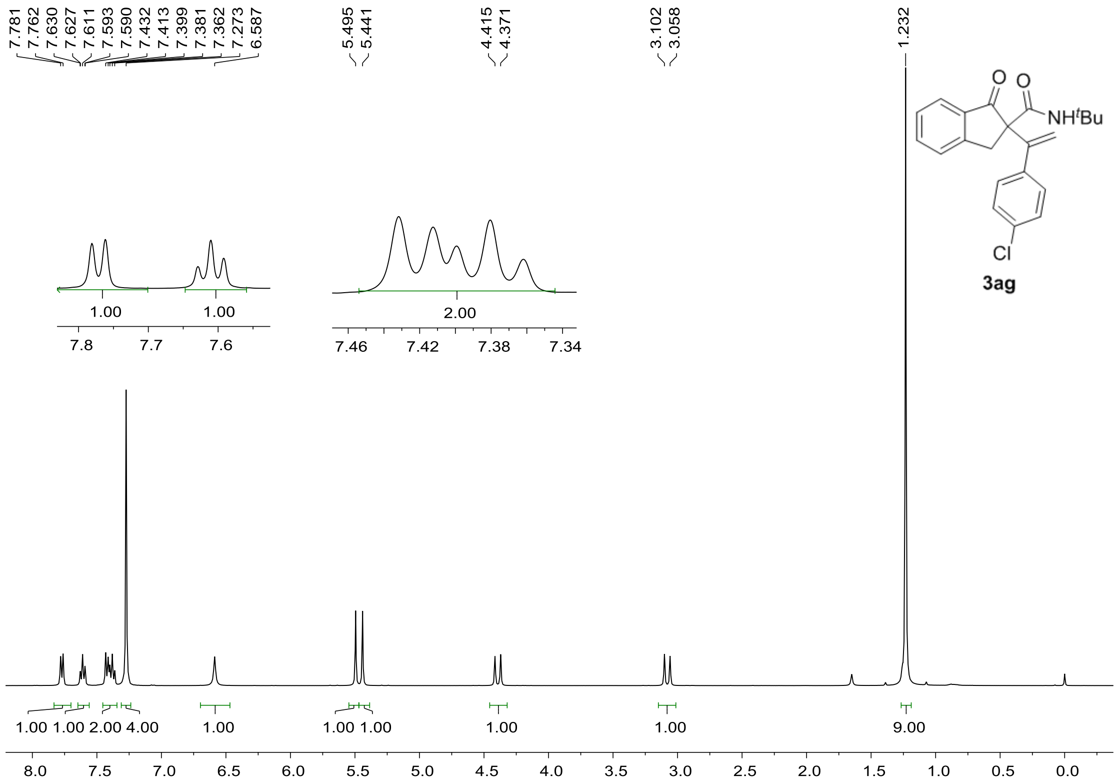

Supplementary Figure 34. ^{1}H NMR spectra for product **3ag**

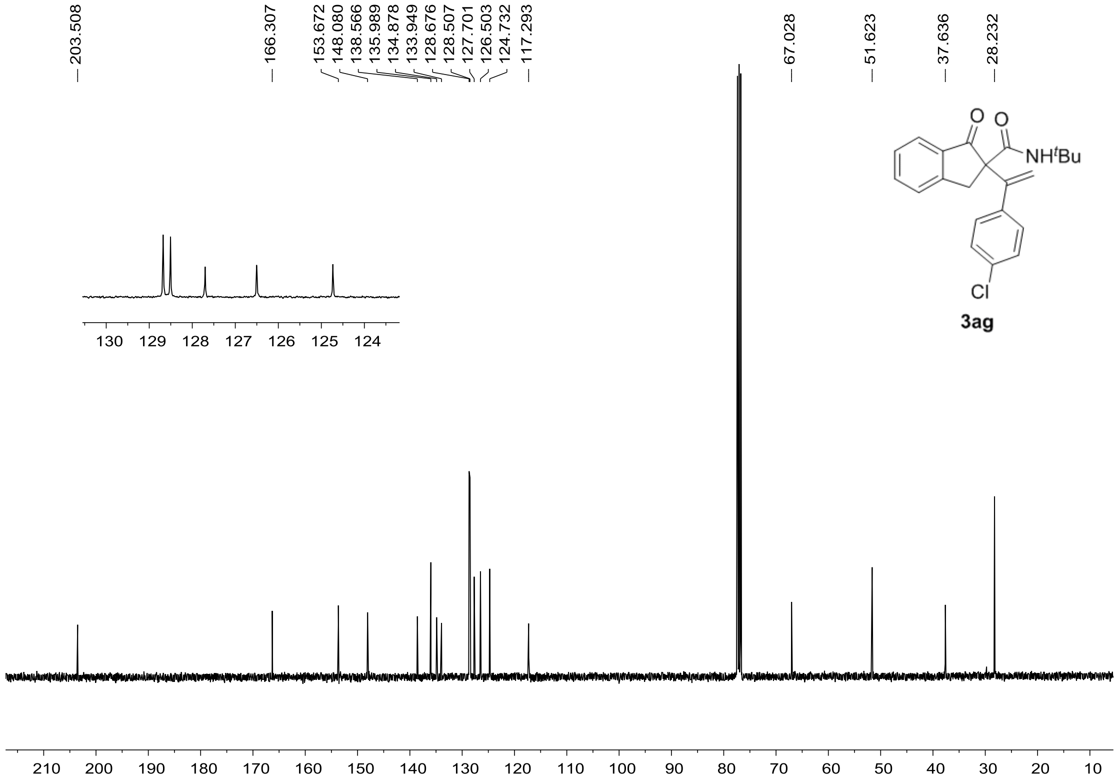

Supplementary Figure 35. ^{13}C NMR spectra for product **3ag**

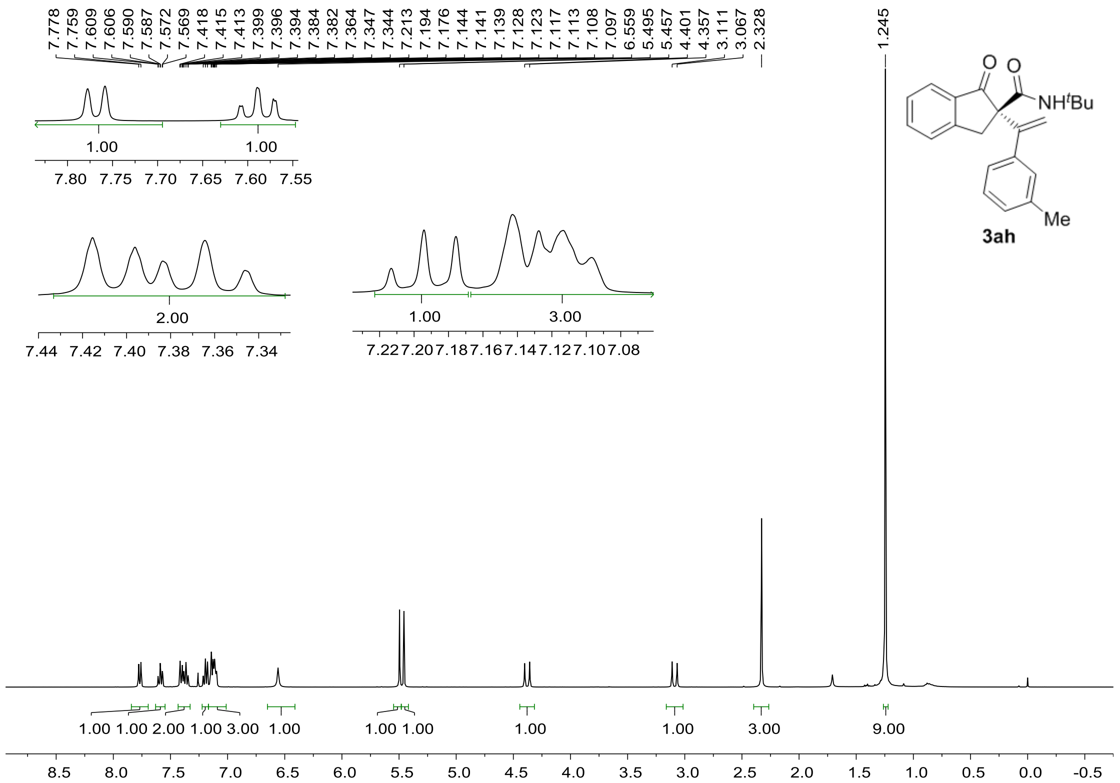

Supplementary Figure 36. ^{1}H NMR spectra for product **3ah**

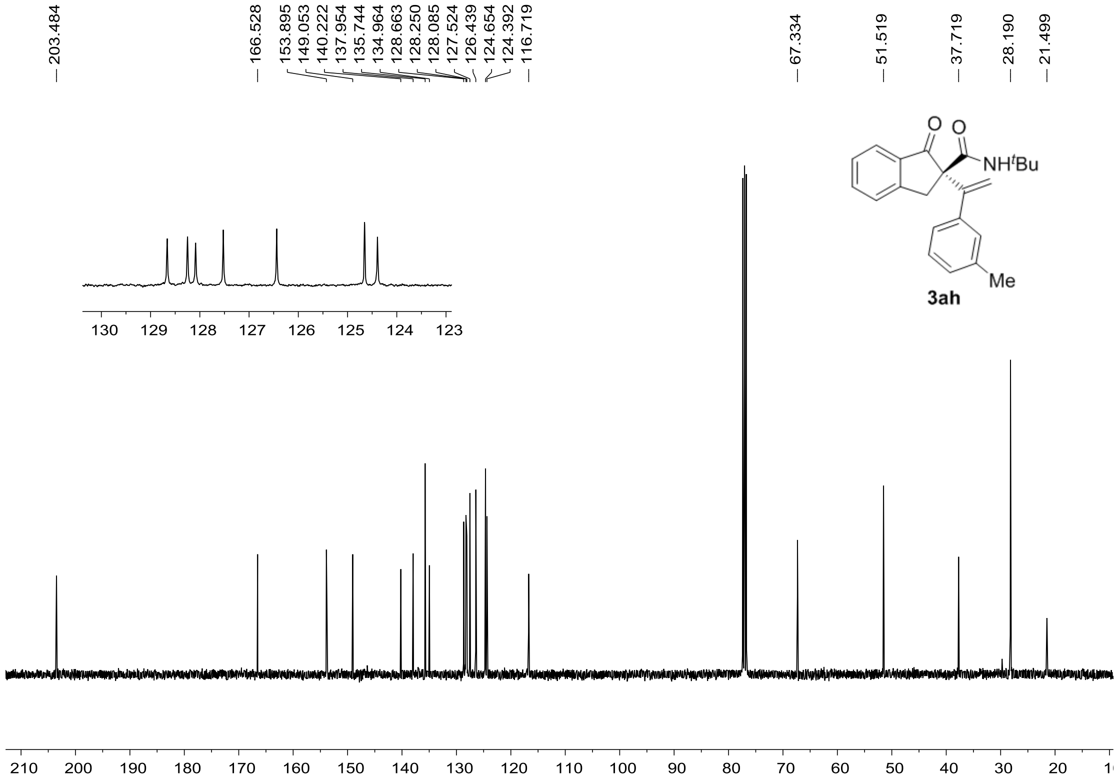

Supplementary Figure 37. ^{13}C NMR spectra for product **3ah**

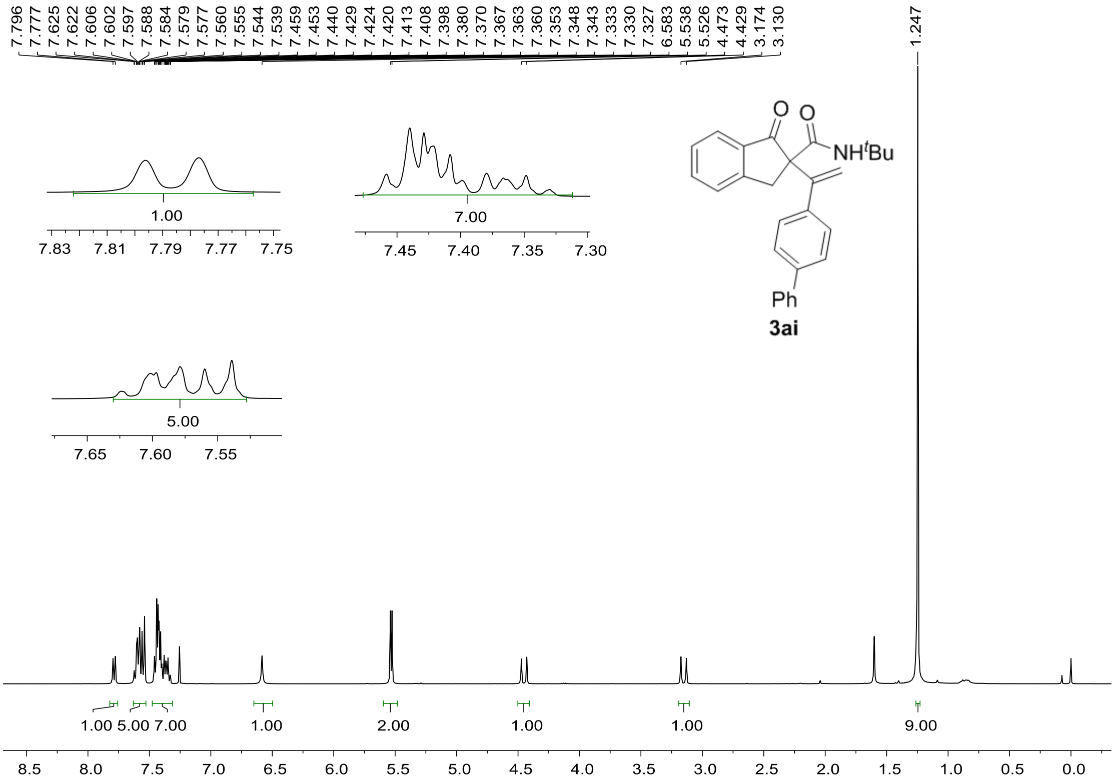

Supplementary Figure 38. ^{1}H NMR spectra for product **3ai**

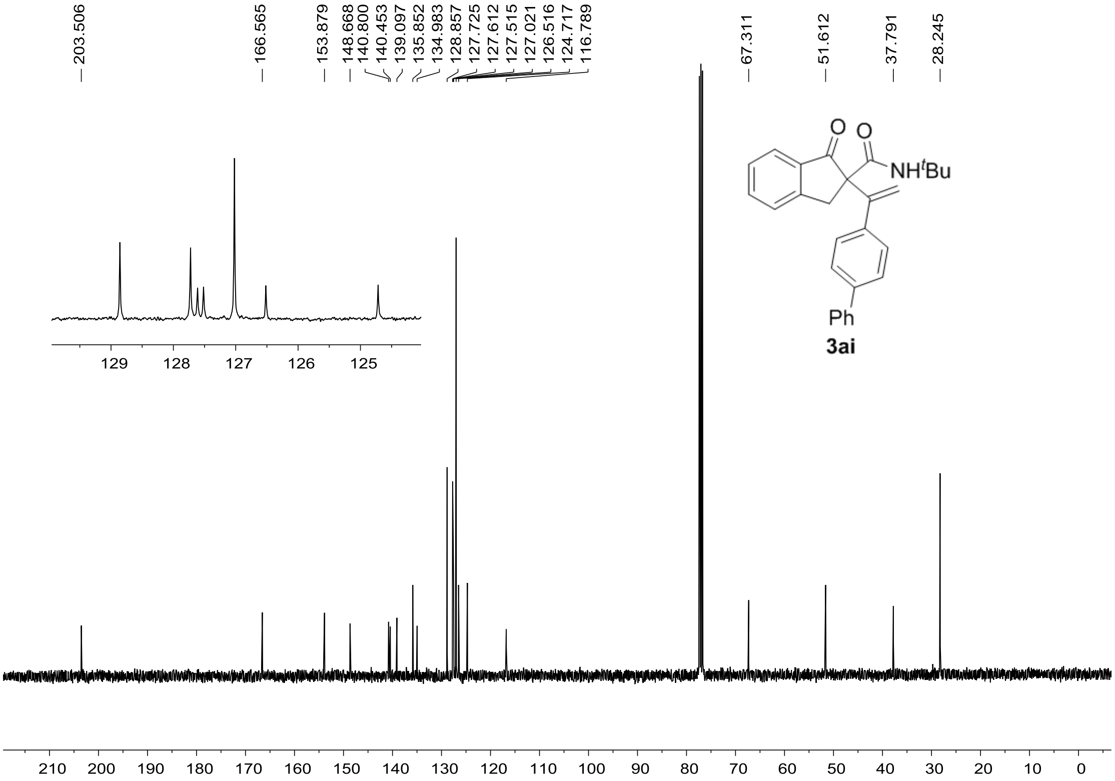

Supplementary Figure 39. ^{13}C NMR spectra for product **3ai**

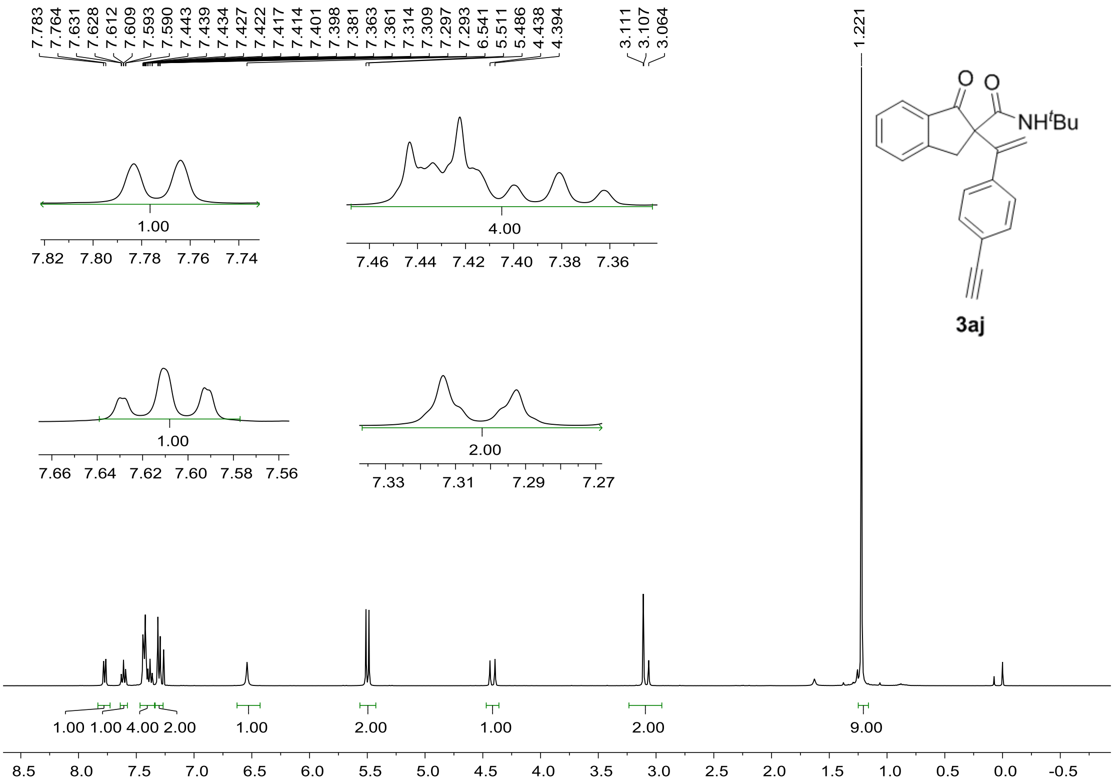

Supplementary Figure 40. ^{1}H NMR spectra for product **3aj**

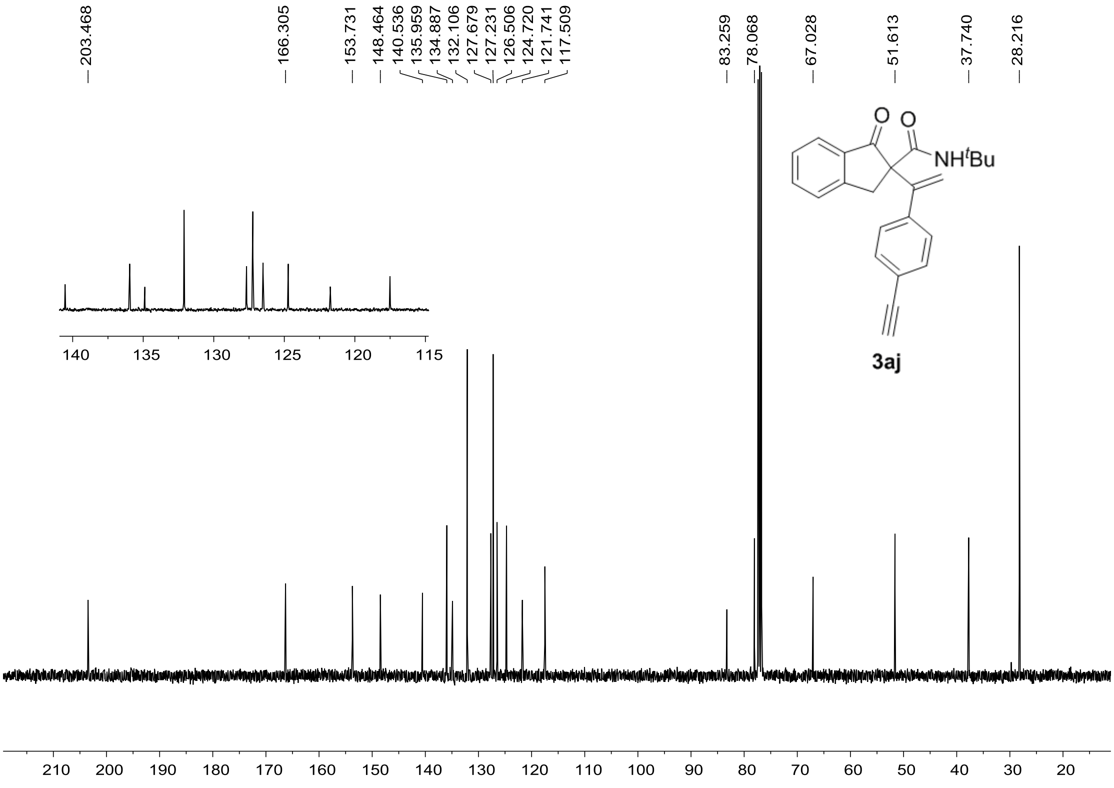

Supplementary Figure 41. ^{13}C NMR spectra for product **3aj**

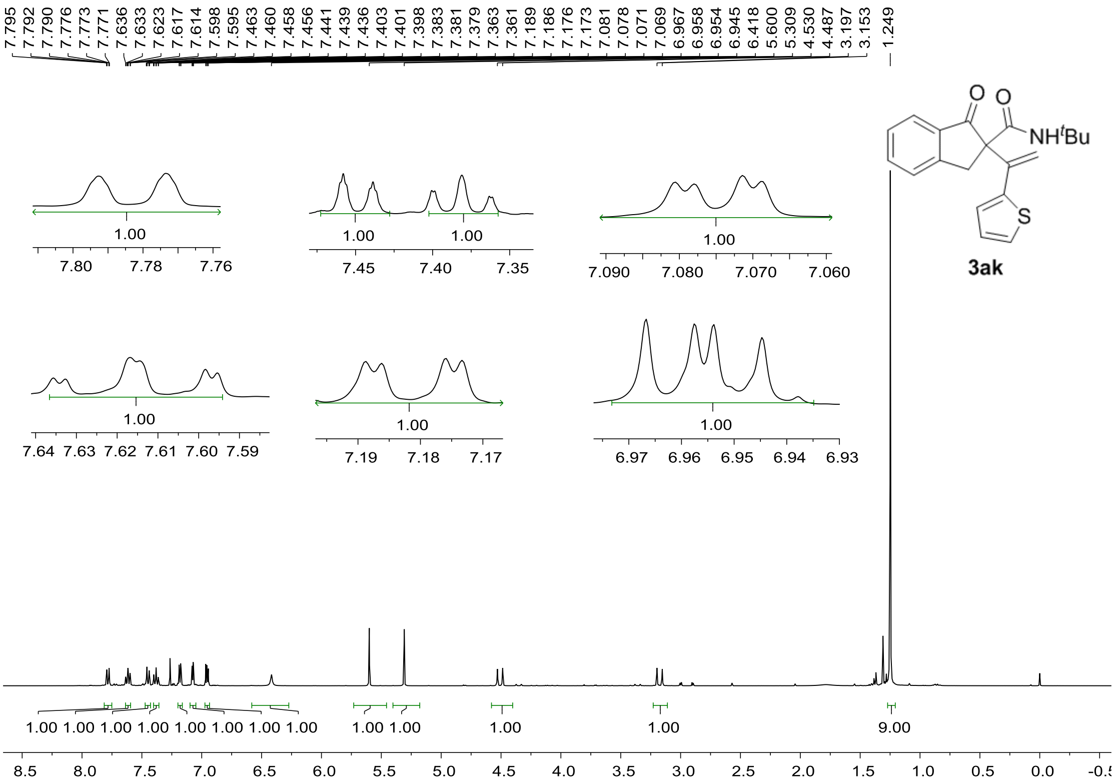

Supplementary Figure 42. ^{1}H NMR spectra for product **3ak**

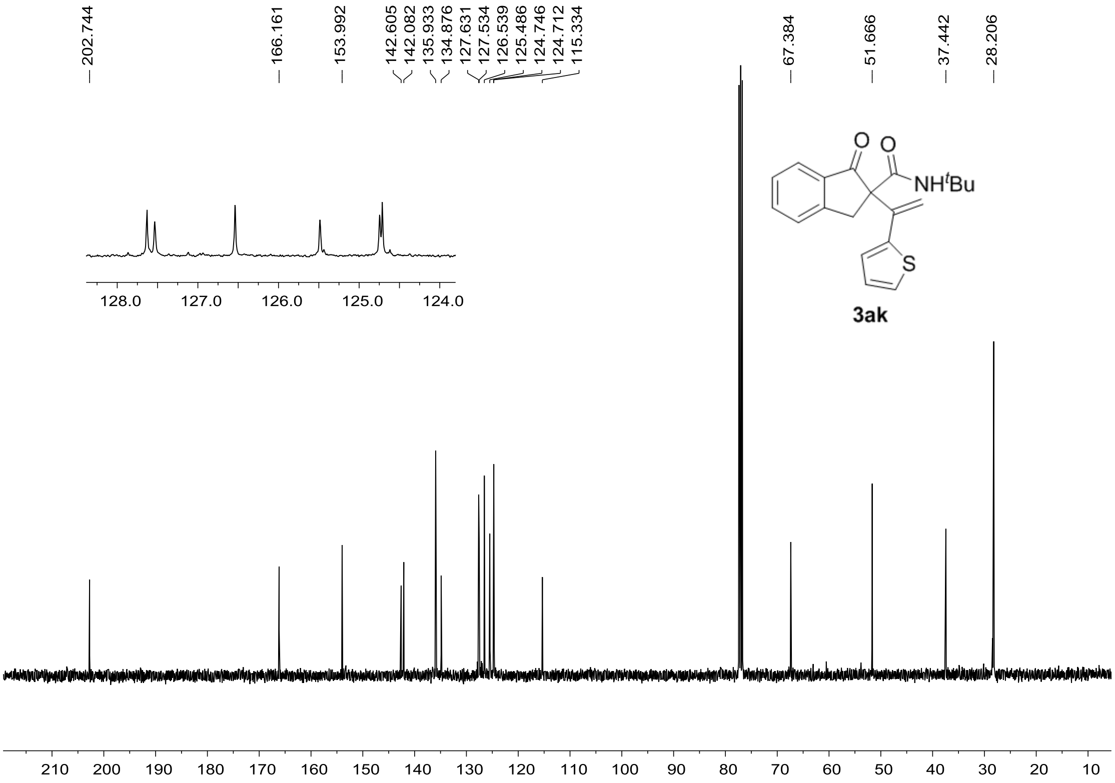

Supplementary Figure 43. ^{13}C NMR spectra for product **3ak**

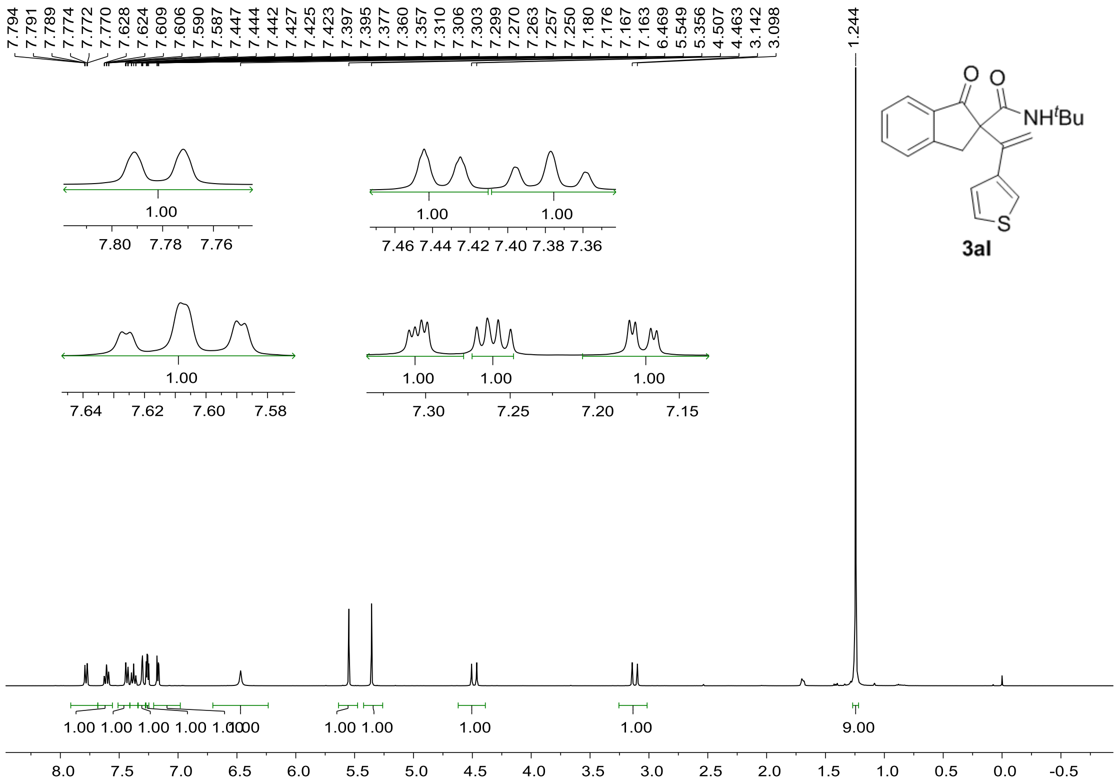

Supplementary Figure 44. ^{1}H NMR spectra for product **3al**

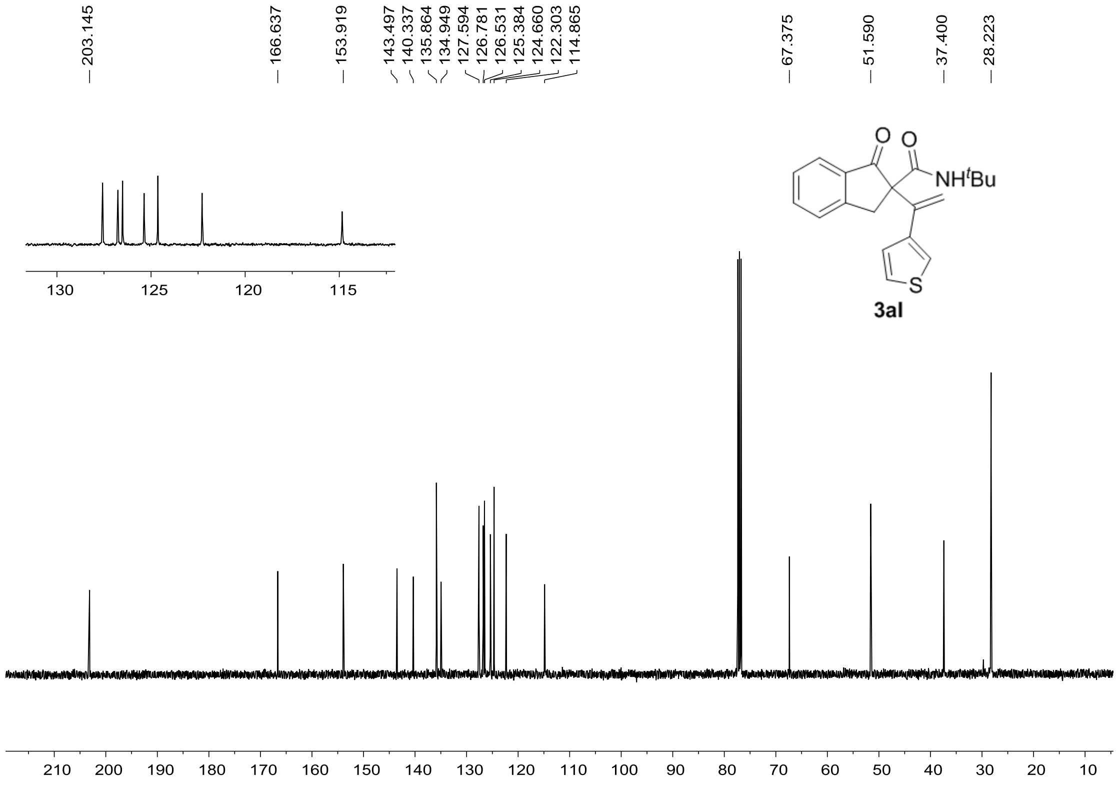

Supplementary Figure 45. ^{13}C NMR spectra for product **3al**

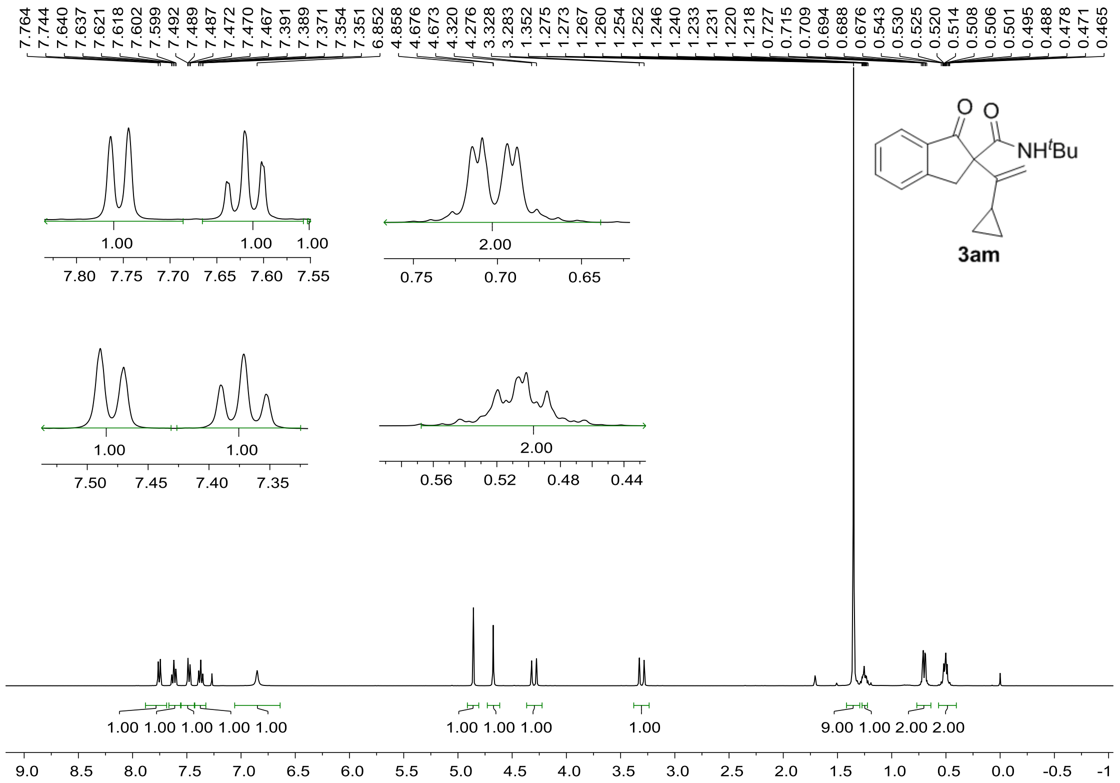

Supplementary Figure 46. ^{1}H NMR spectra for product **3am**

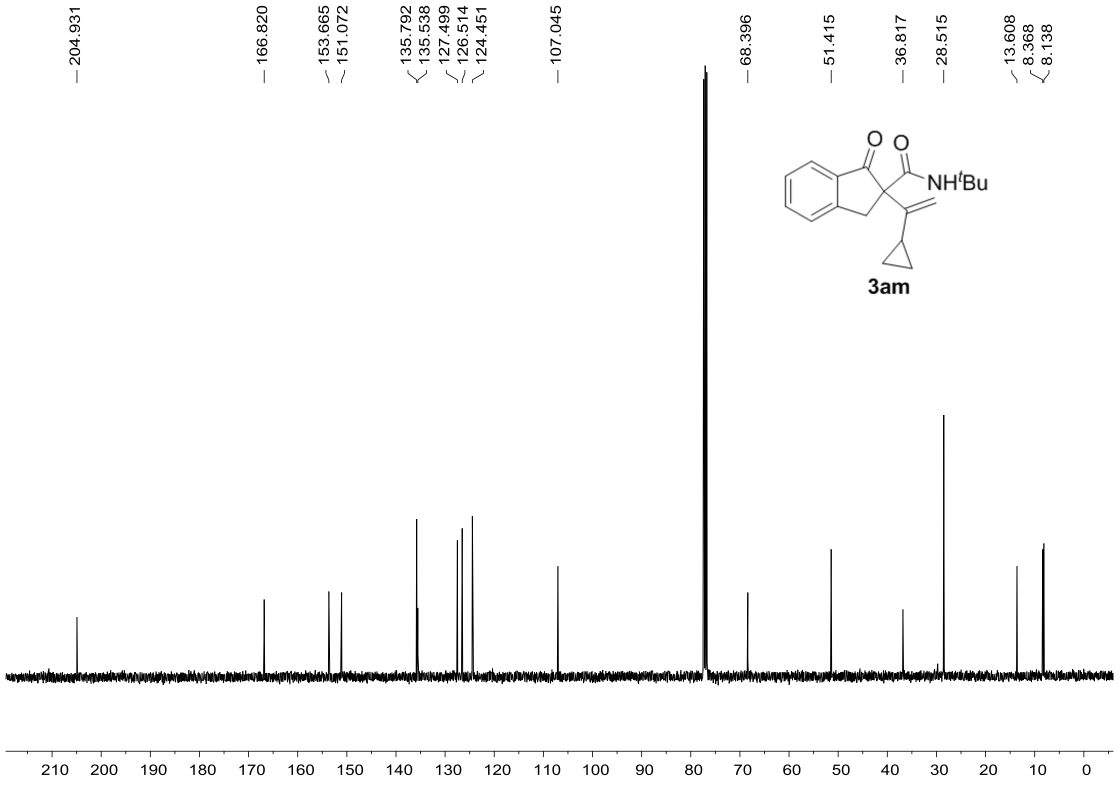

Supplementary Figure 47. ^{13}C NMR spectra for product **3am**

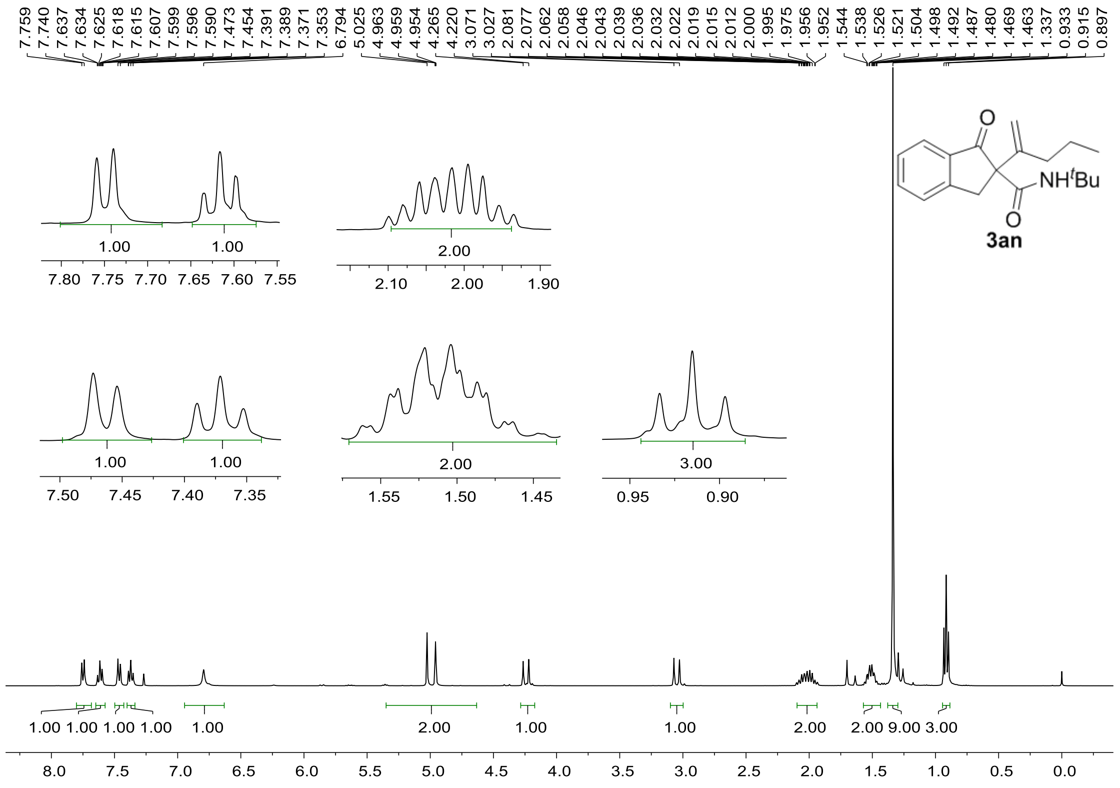

Supplementary Figure 48. ^{1}H NMR spectra for product **3an**

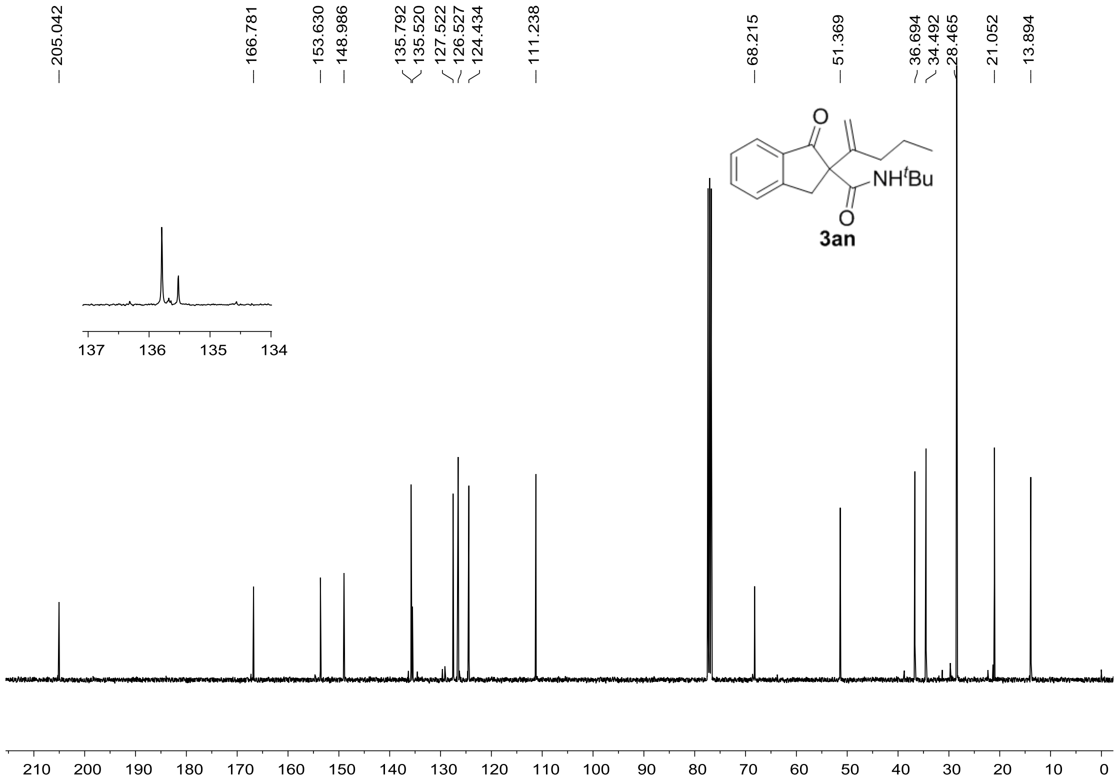

Supplementary Figure 49. ^{13}C NMR spectra for product **3an**

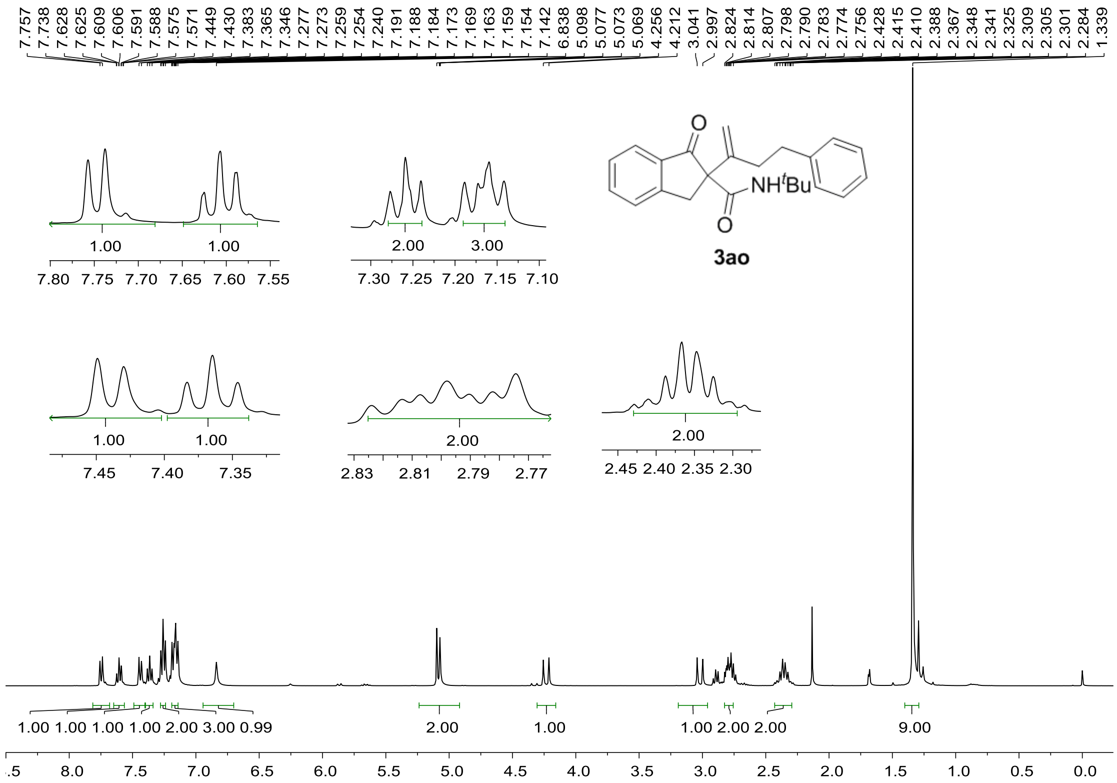

Supplementary Figure 50. ^{1}H NMR spectra for product **3ao**

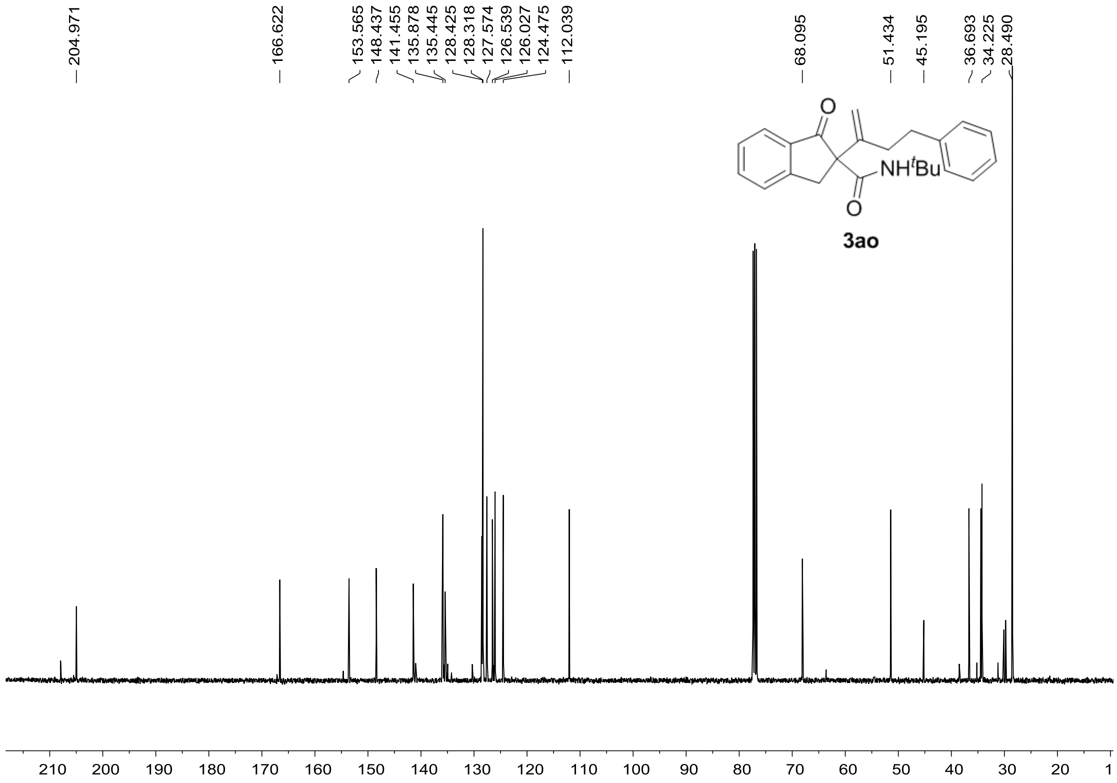

Supplementary Figure 51. ^{13}C NMR spectra for product **3ao**

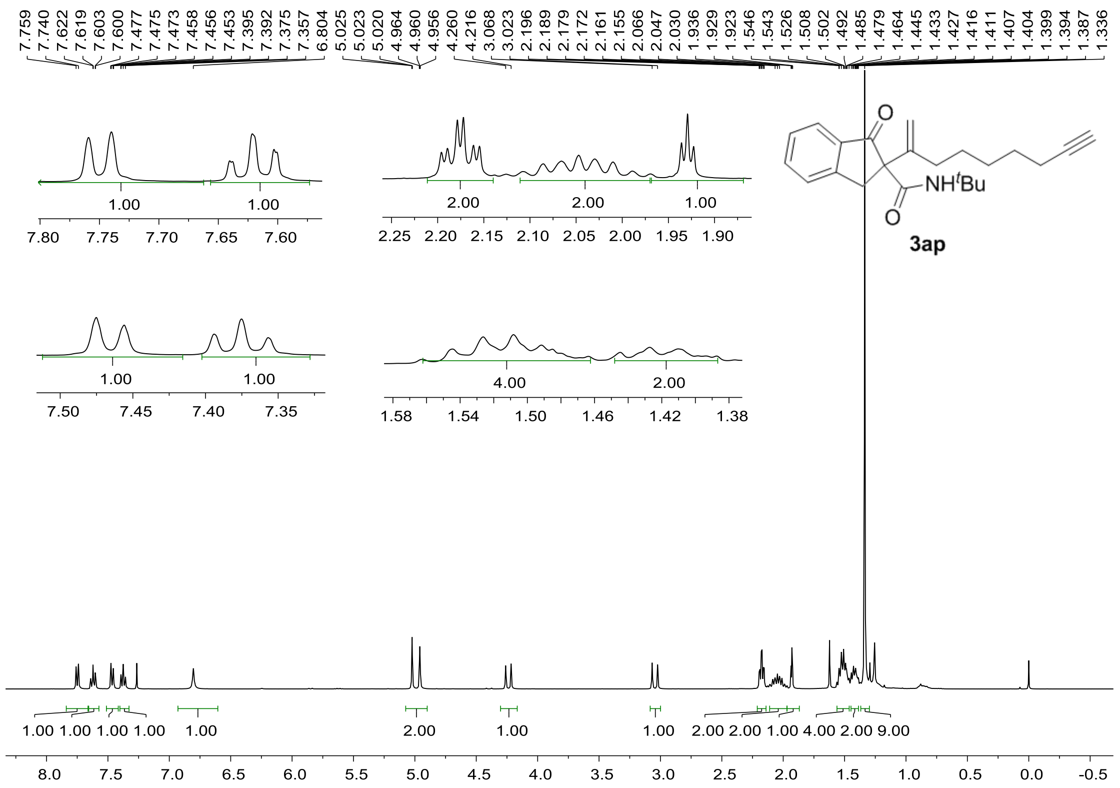

Supplementary Figure 52. ^{1}H NMR spectra for product **3ap**

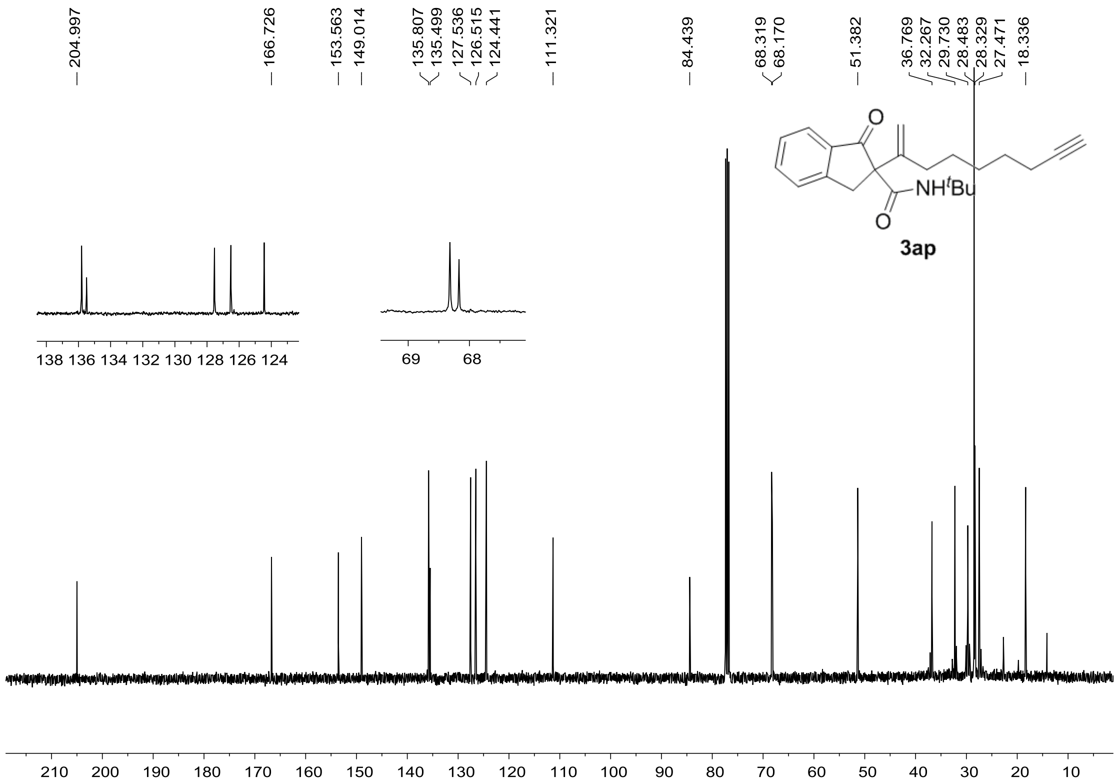

Supplementary Figure 53. ^{13}C NMR spectra for product **3ap**

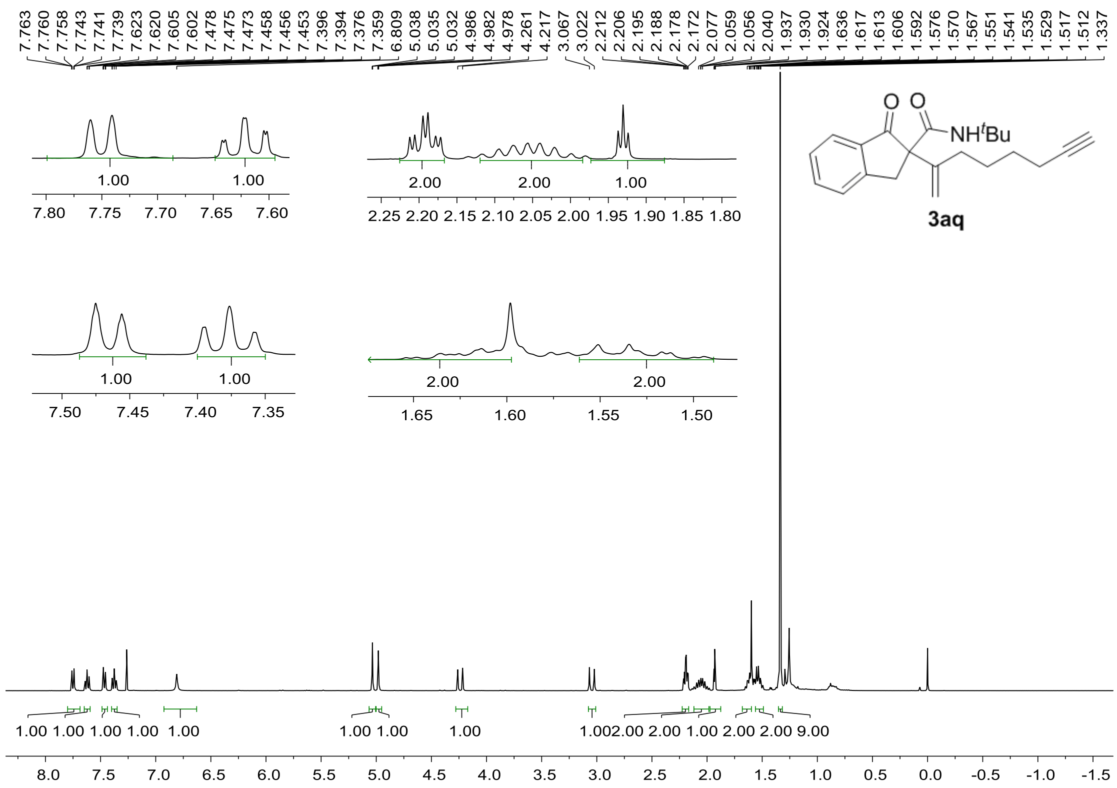

Supplementary Figure 54. ^{1}H NMR spectra for product **3aq**

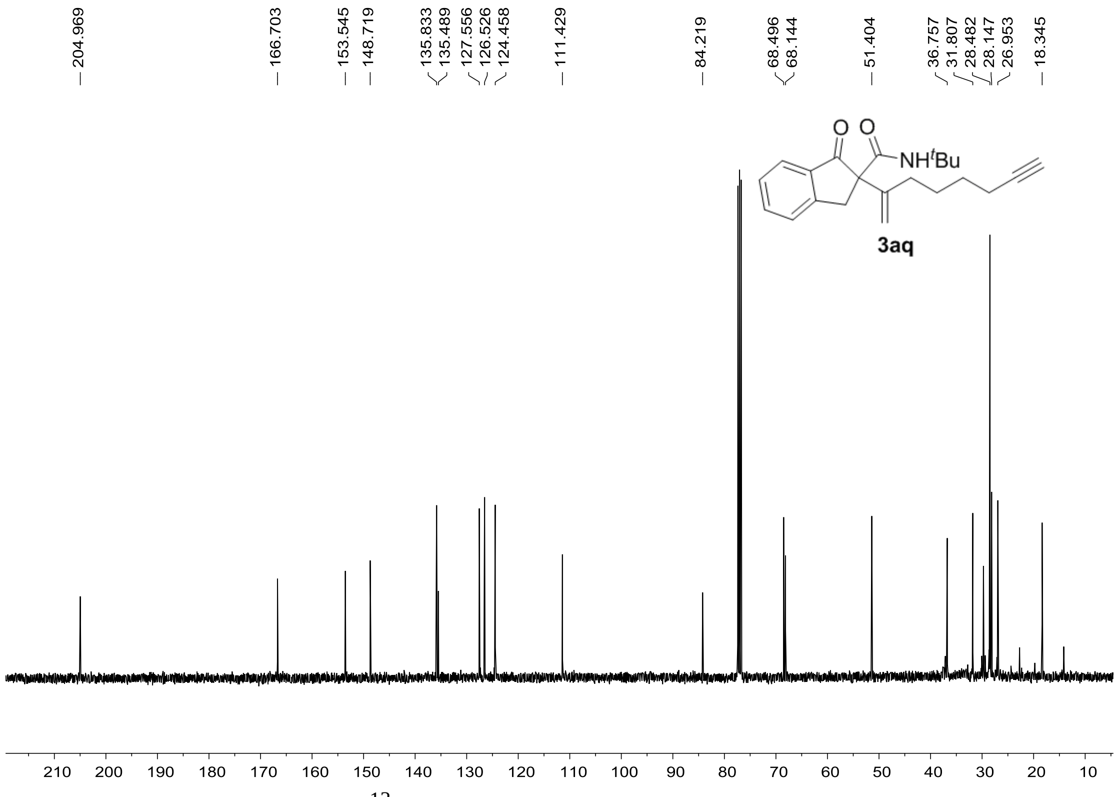

Supplementary Figure 55. ^{13}C NMR spectra for product **3aq**

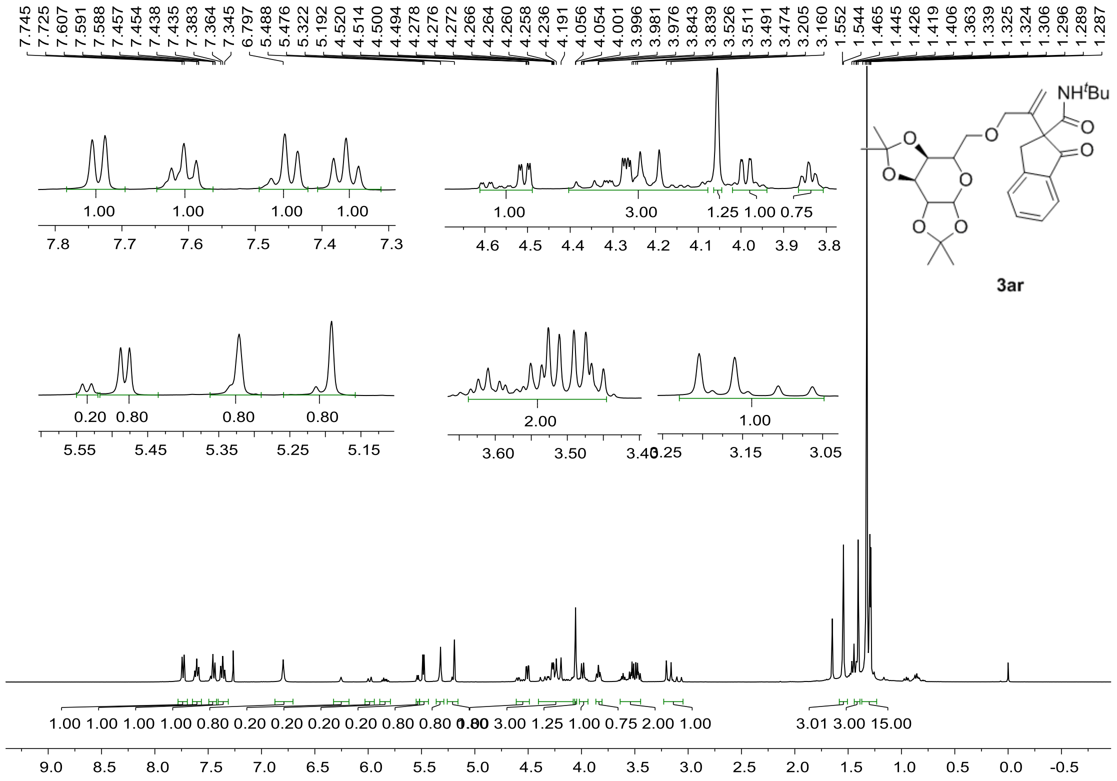

Supplementary Figure 56. ^{1}H NMR spectra for product **3ar**

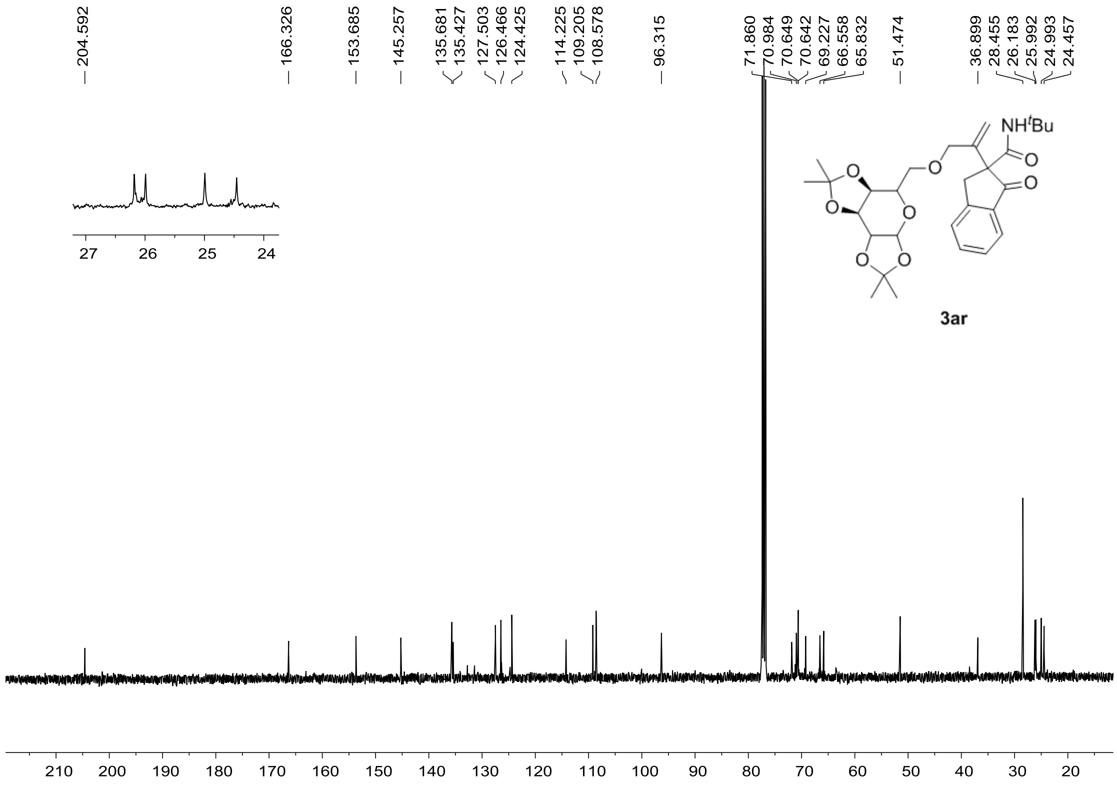

Supplementary Figure 57. ^{13}C NMR spectra for product **3ar**

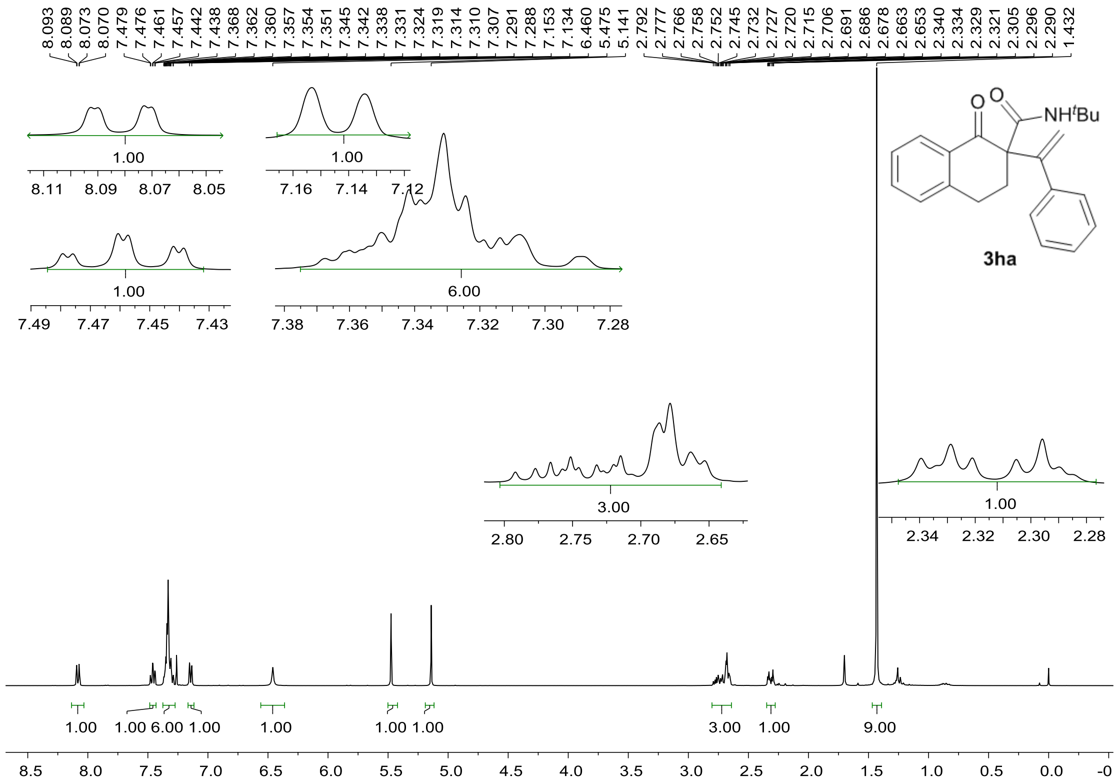

Supplementary Figure 58. ^{1}H NMR spectra for product **3ha**

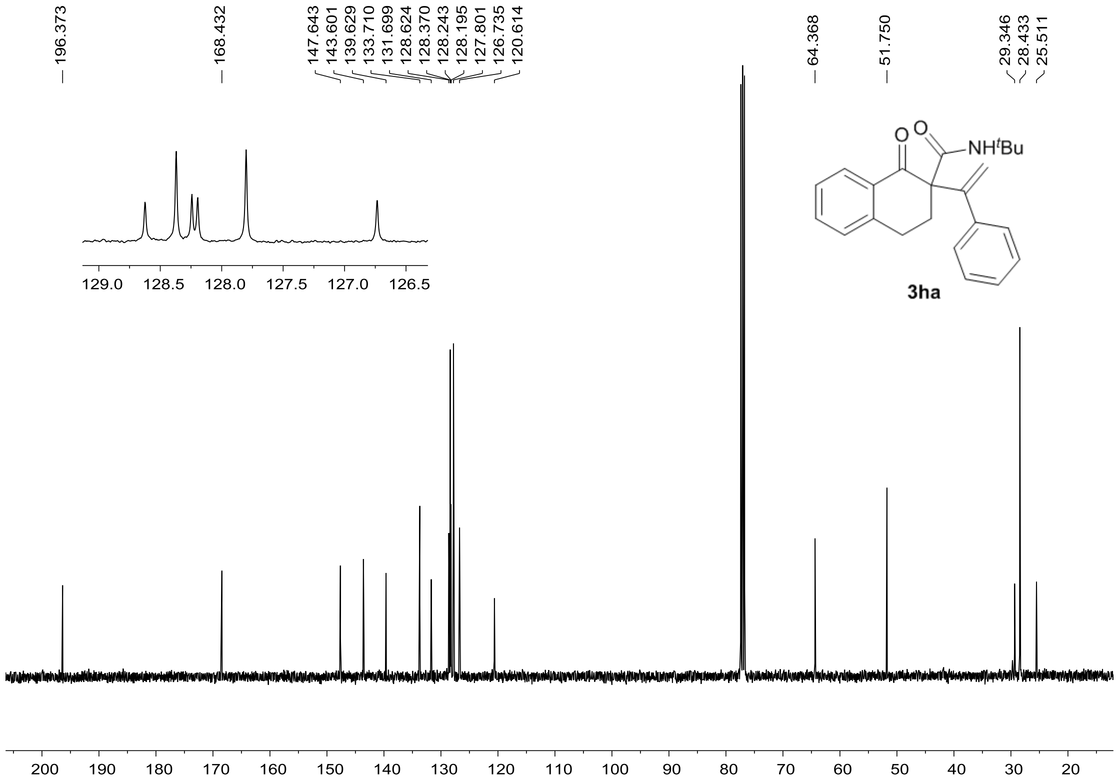

Supplementary Figure 59. ^{13}C NMR spectra for product **3ha**

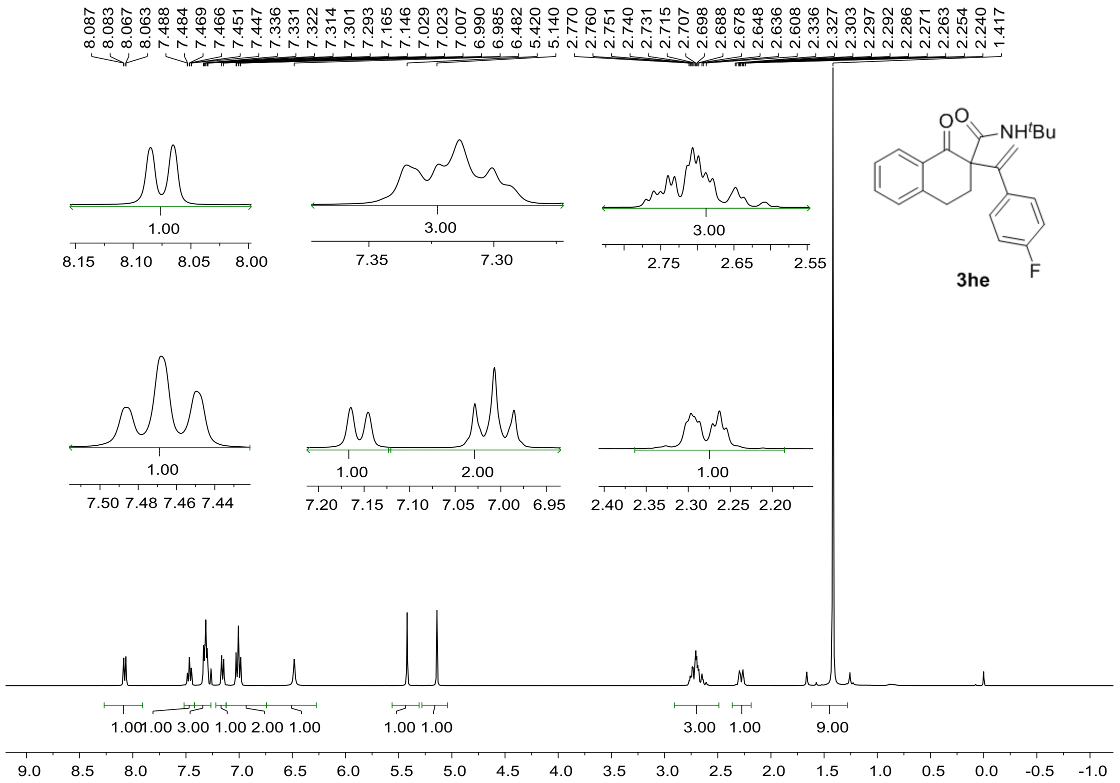

Supplementary Figure 60. ^{1}H NMR spectra for product **3he**

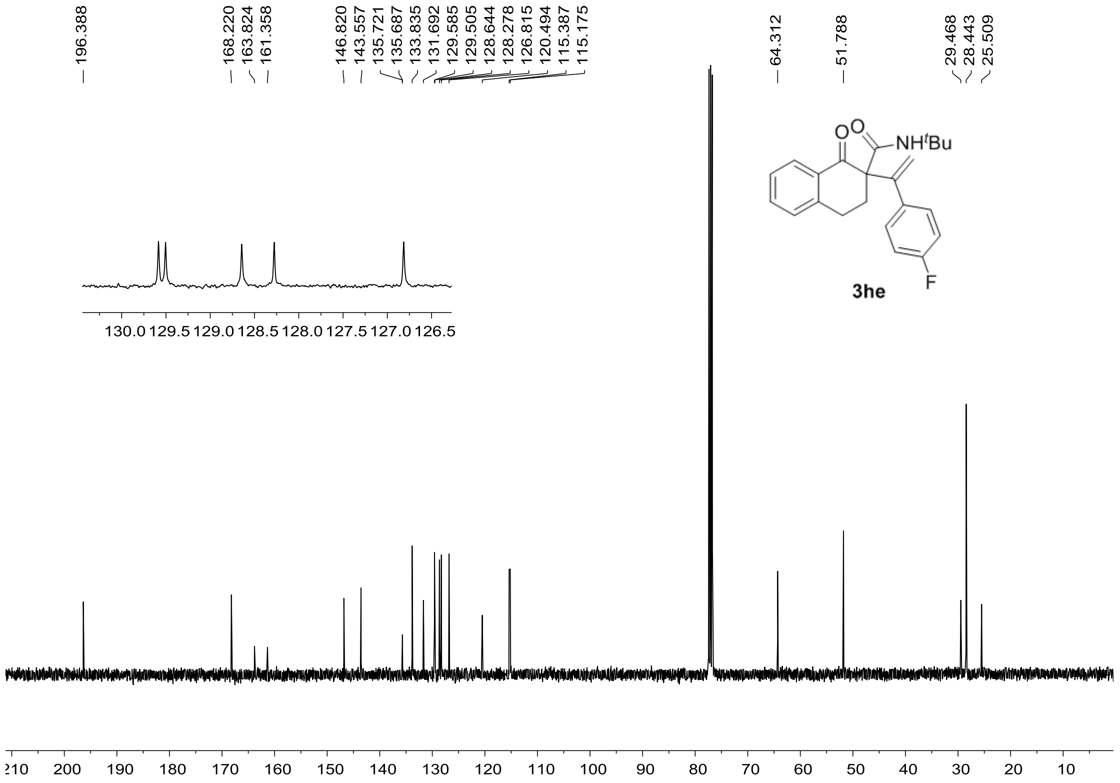

Supplementary Figure 61. ^{13}C NMR spectra for product **3he**

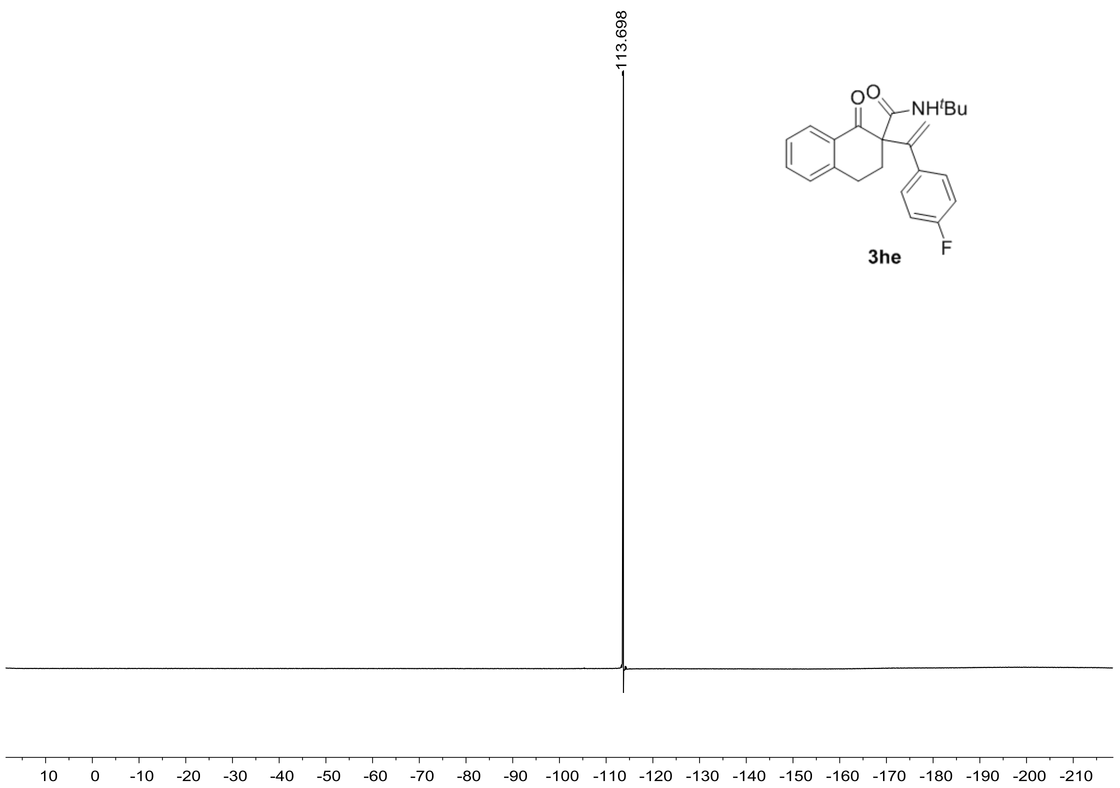

Supplementary Figure 62. ^{19}F NMR spectra for product **3he**

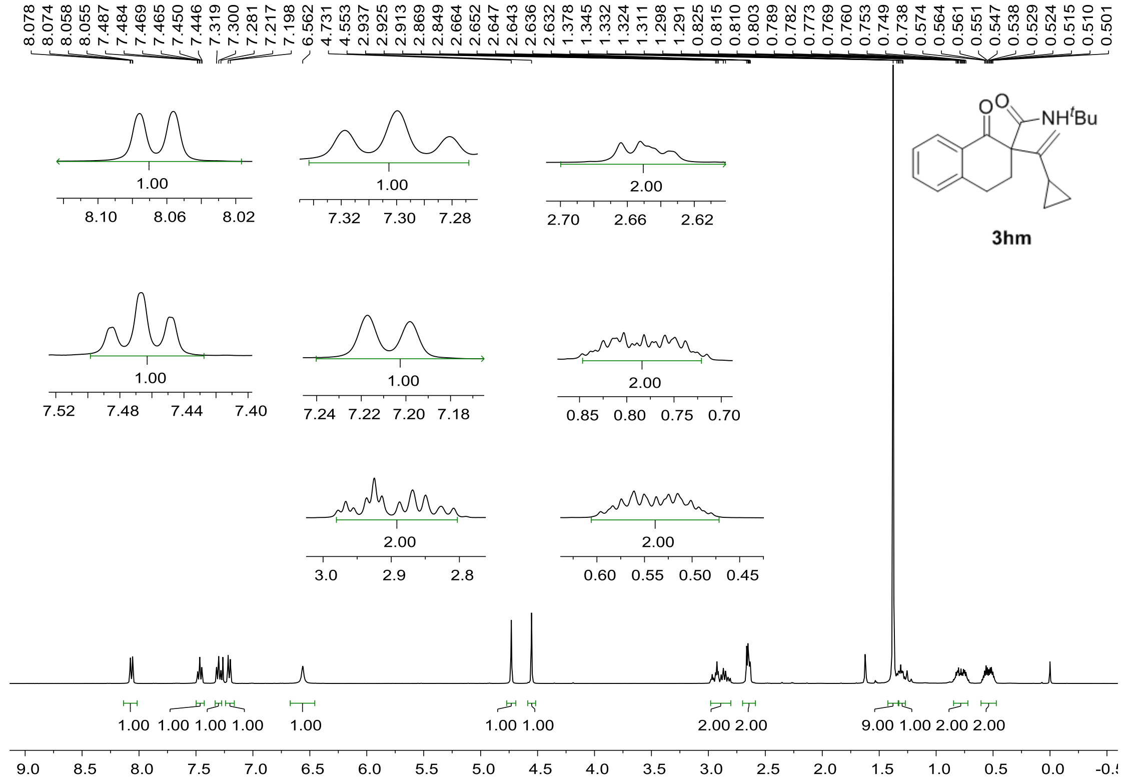

Supplementary Figure 63. ^{1}H NMR spectra for product **3hm**

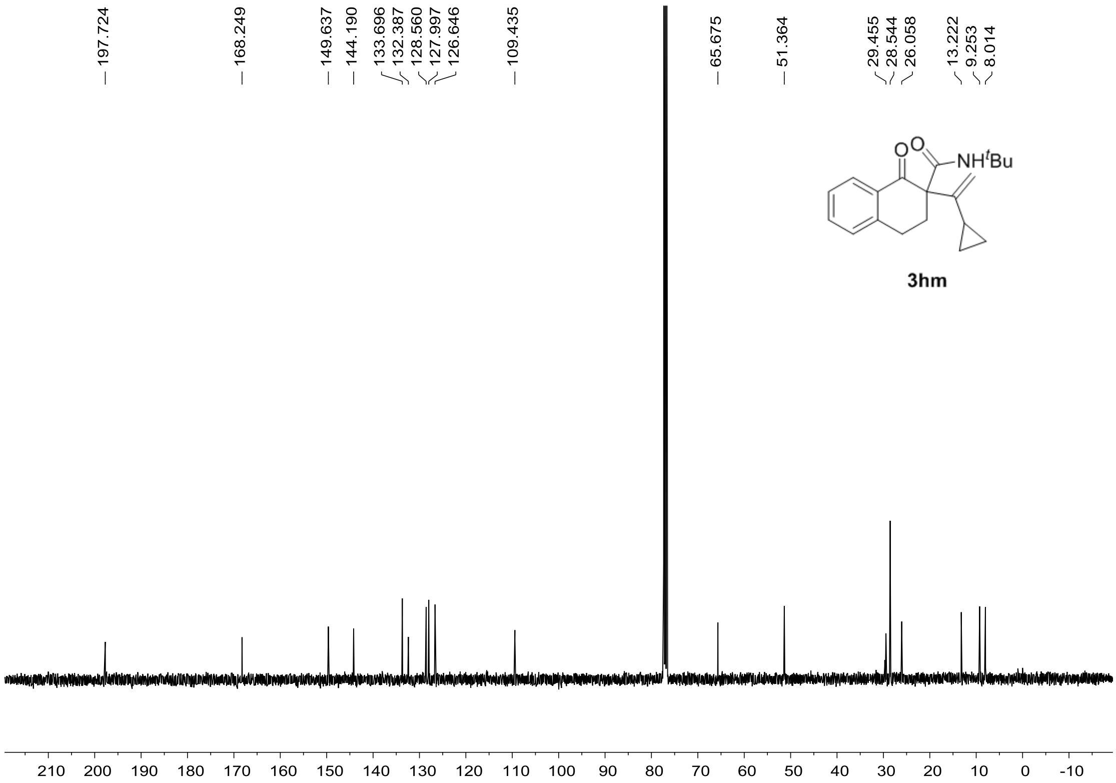

Supplementary Figure 64. ^{13}C NMR spectra for product **3hm**

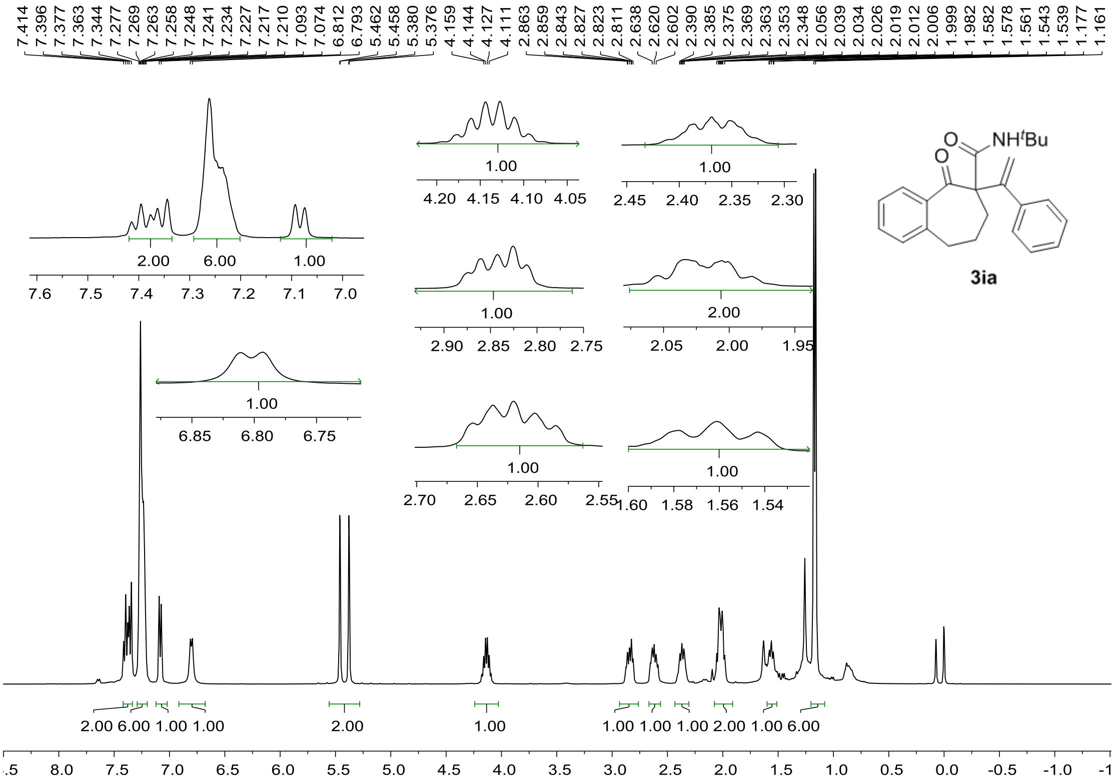

Supplementary Figure 65. ^{1}H NMR spectra for product **3ia**

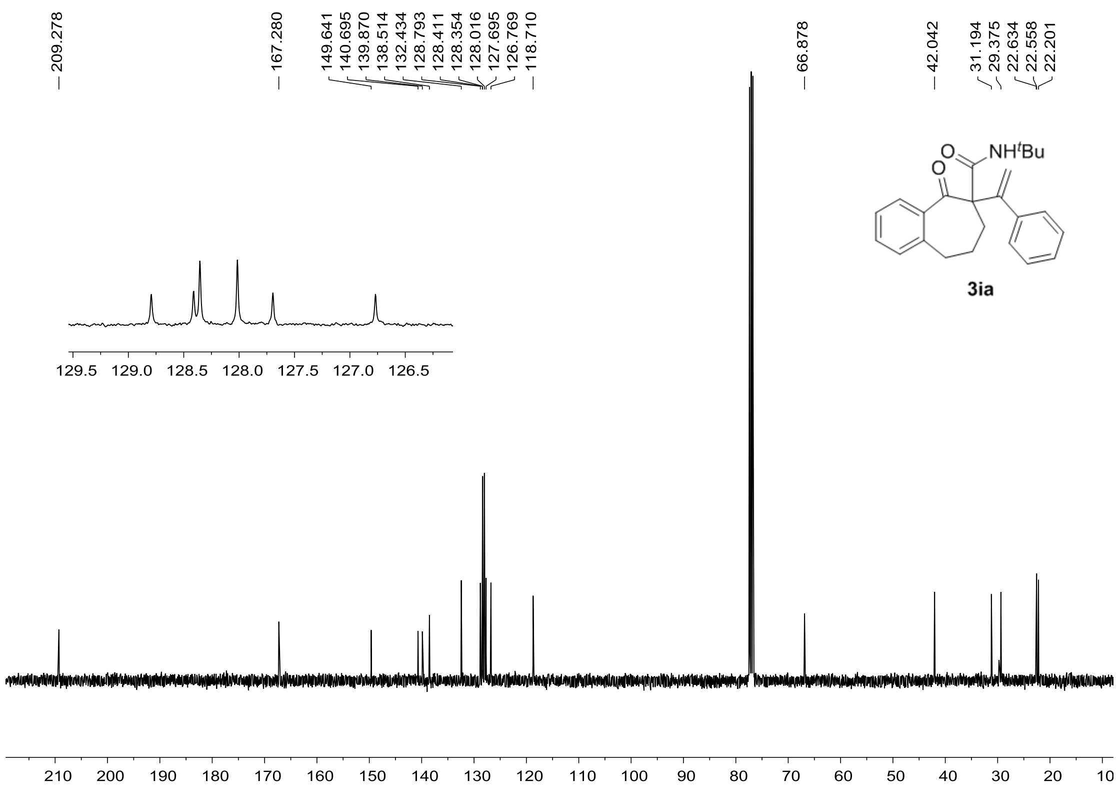

Supplementary Figure 66. ^{13}C NMR spectra for product **3ia**

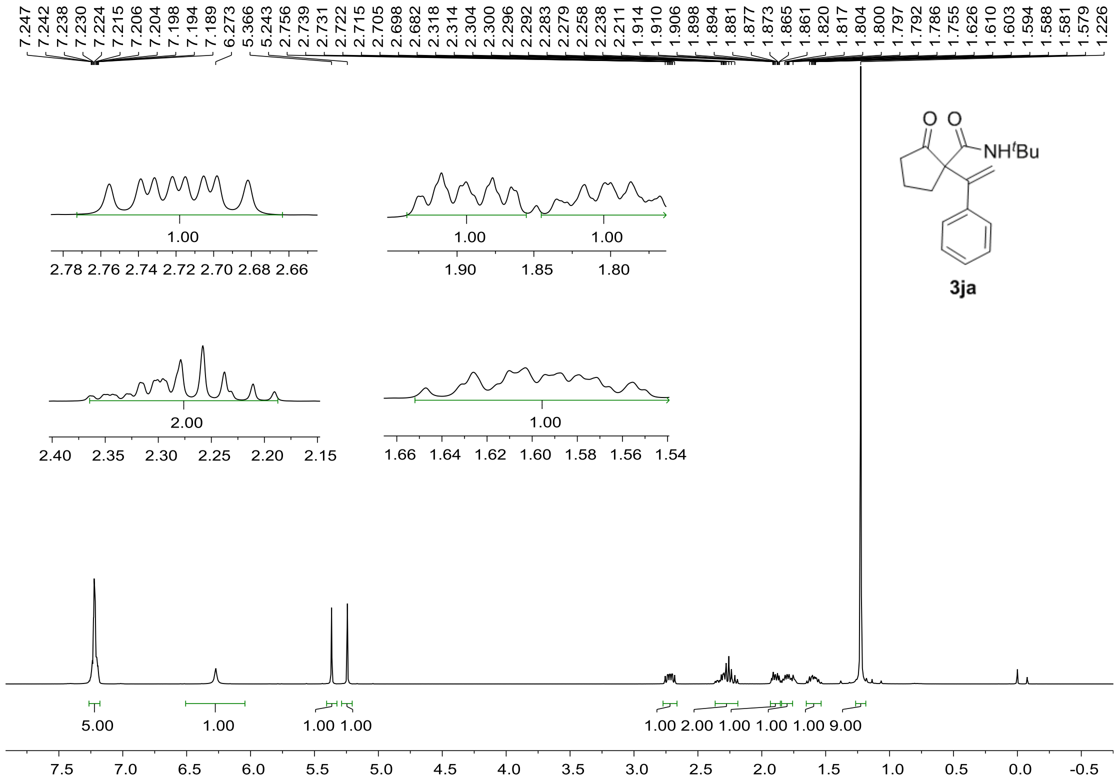

Supplementary Figure 67. ^{1}H NMR spectra for product **3ja**

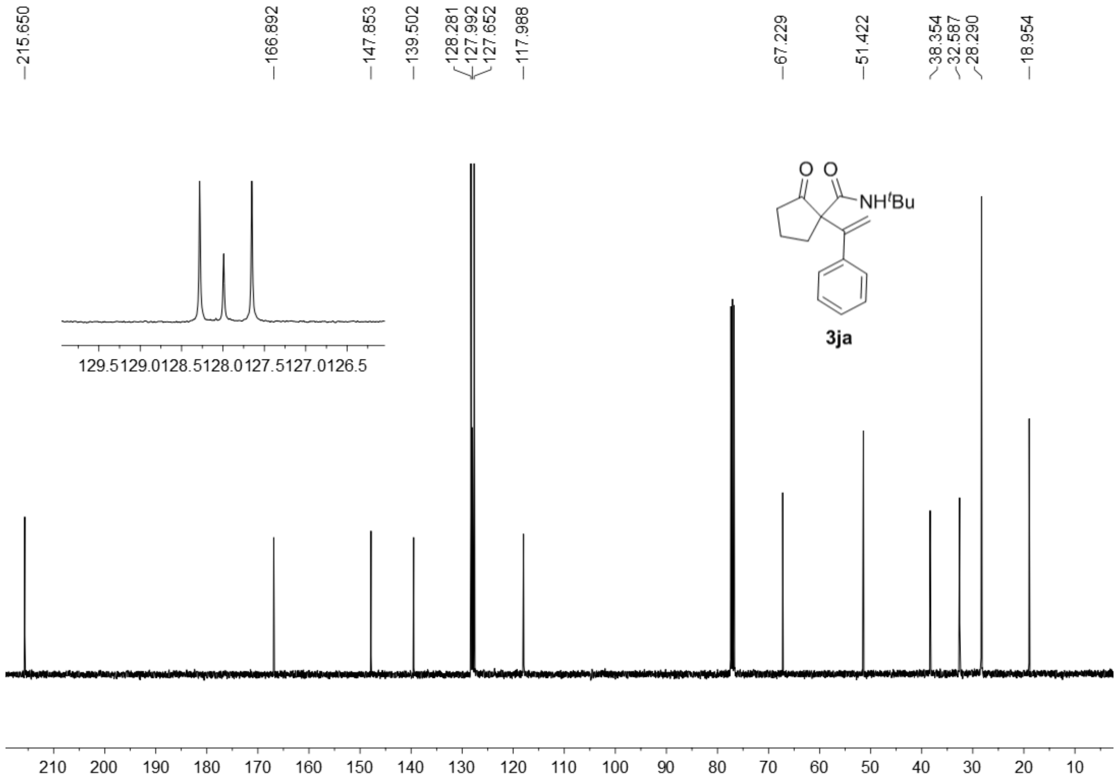

Supplementary Figure 68. ^{13}C NMR spectra for product **3ja**

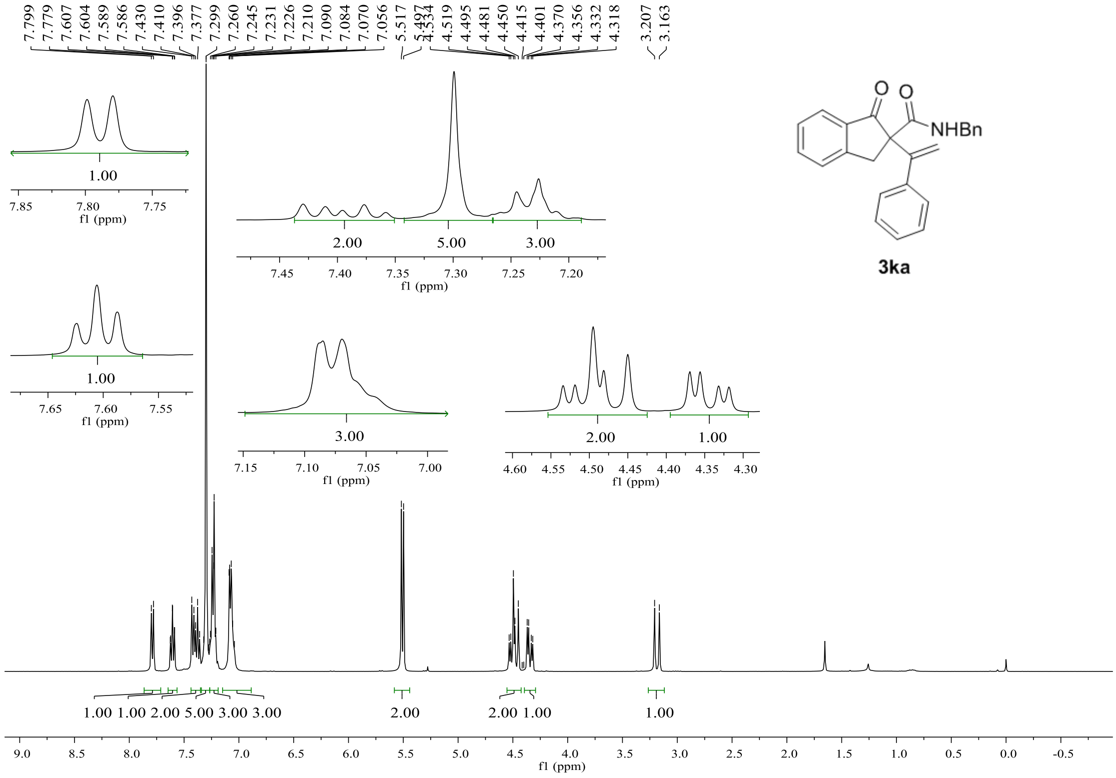

Supplementary Figure 69. ^{1}H NMR spectra for product **3ka**

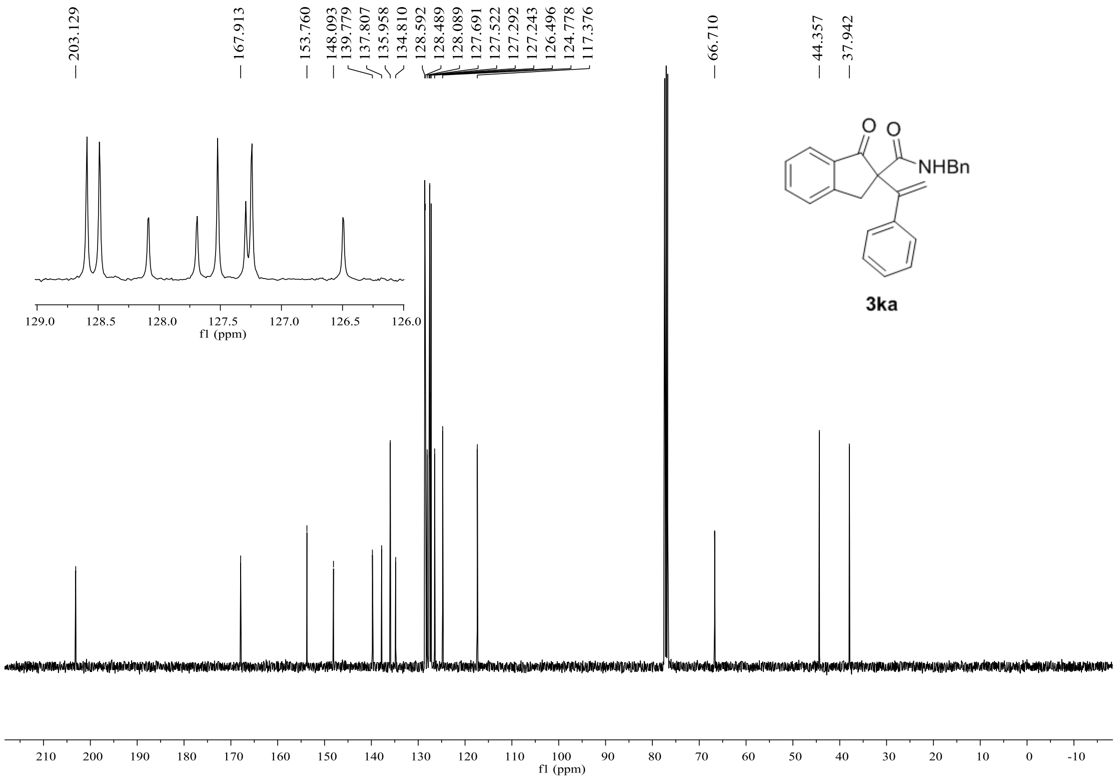

Supplementary Figure 70. ^{13}C NMR spectra for product **3ka**

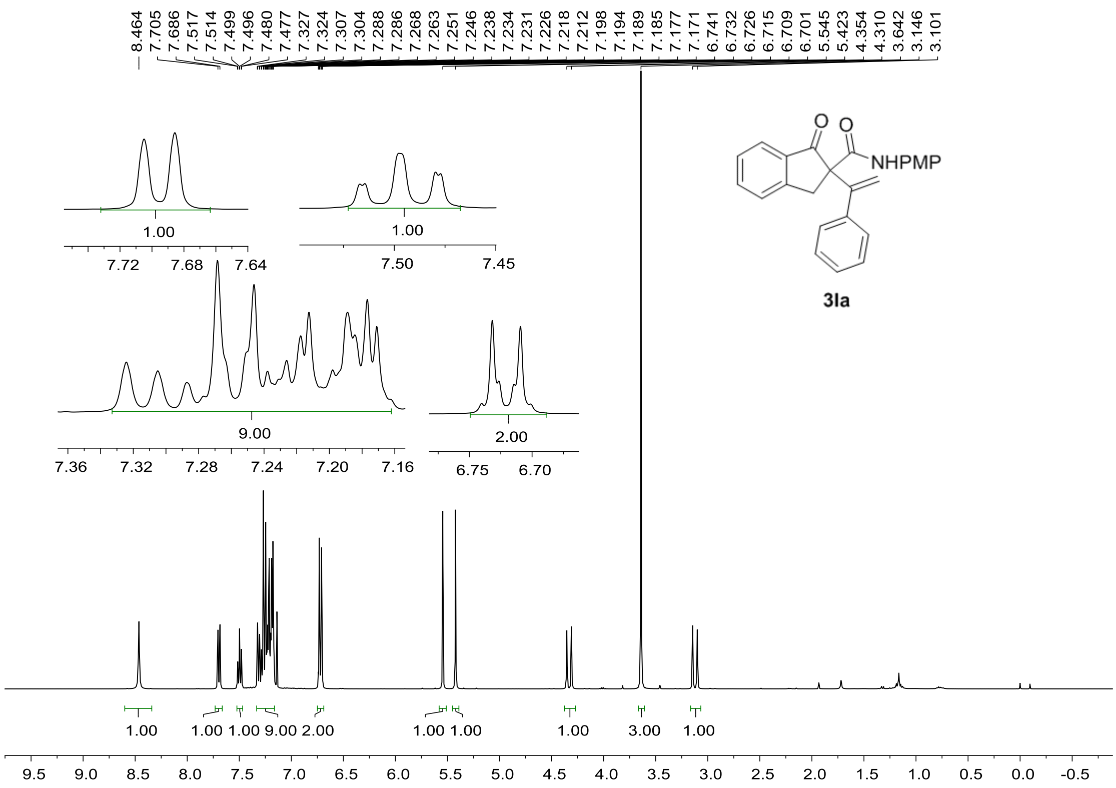

Supplementary Figure 71. ^{1}H NMR spectra for product **3la**

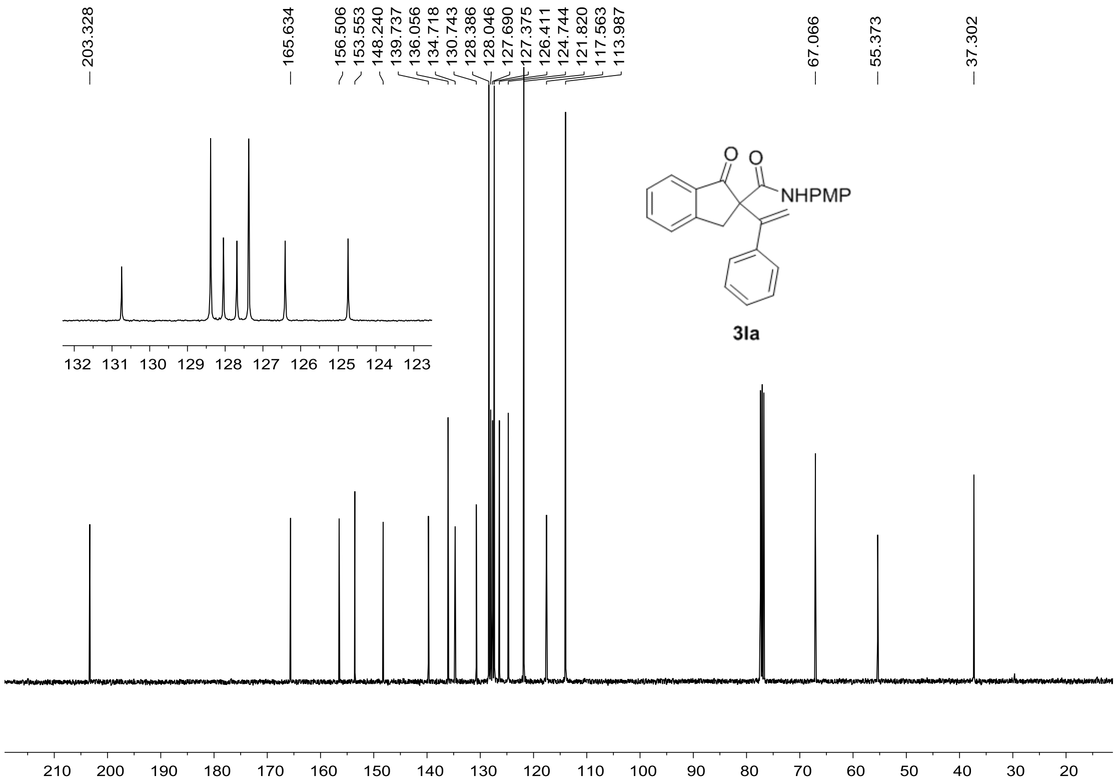

Supplementary Figure 72. ^{13}C NMR spectra for product **3la**

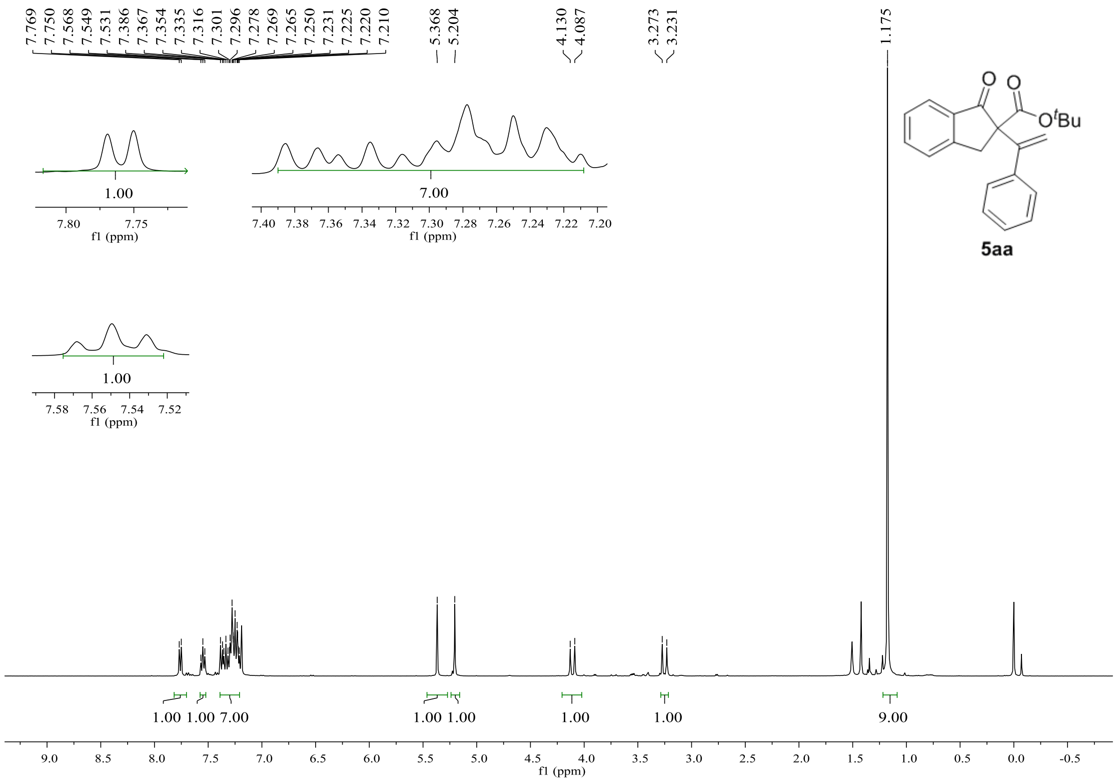

Supplementary Figure 73. ^{1}H NMR spectra for product **5aa**

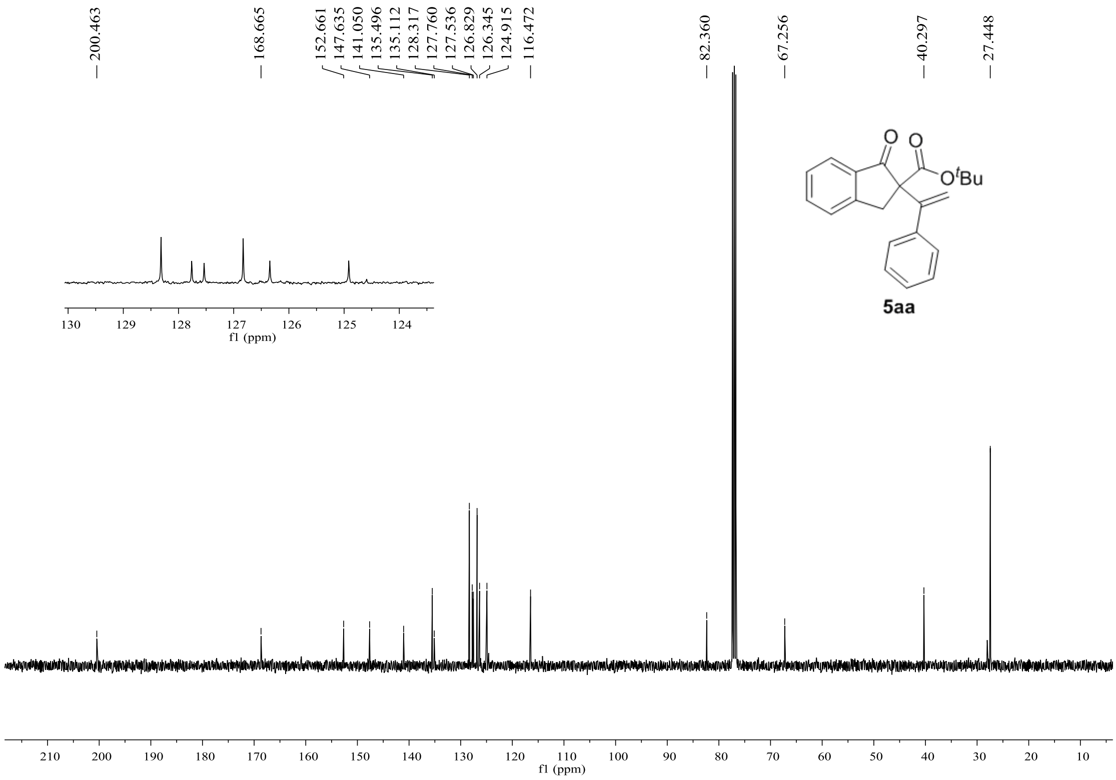

Supplementary Figure 74. ^{13}C NMR spectra for product **5aa**

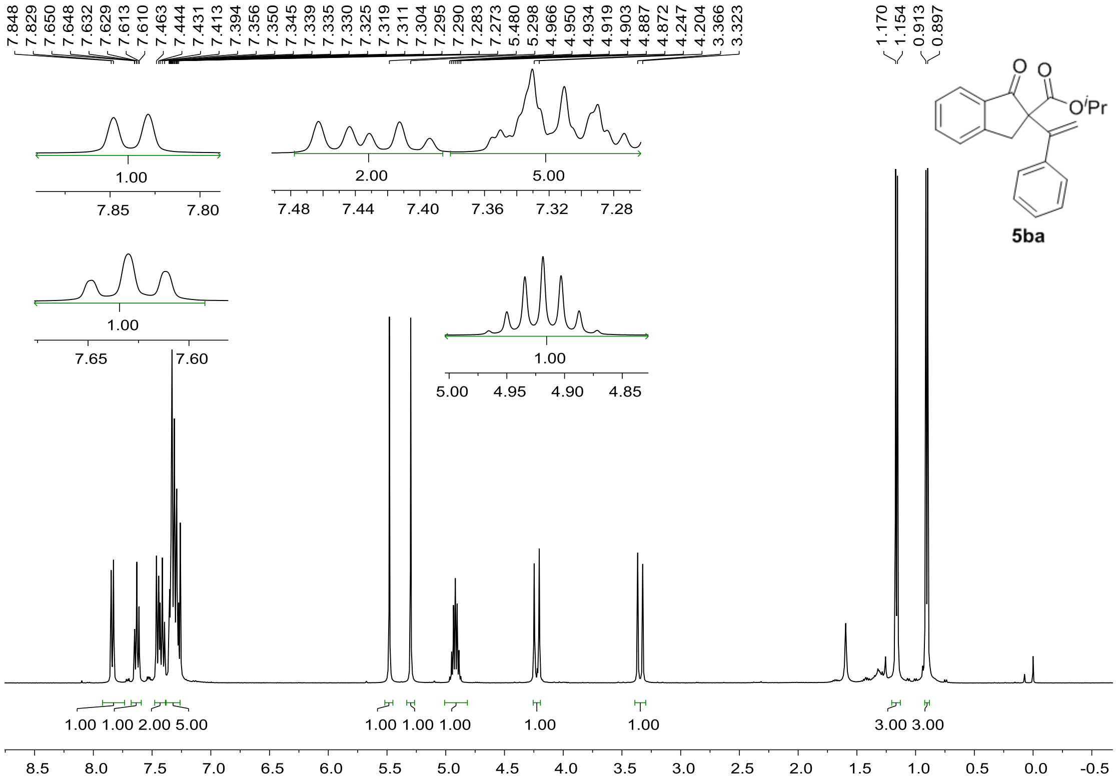

Supplementary Figure 75. ^{1}H NMR spectra for product **5ba**

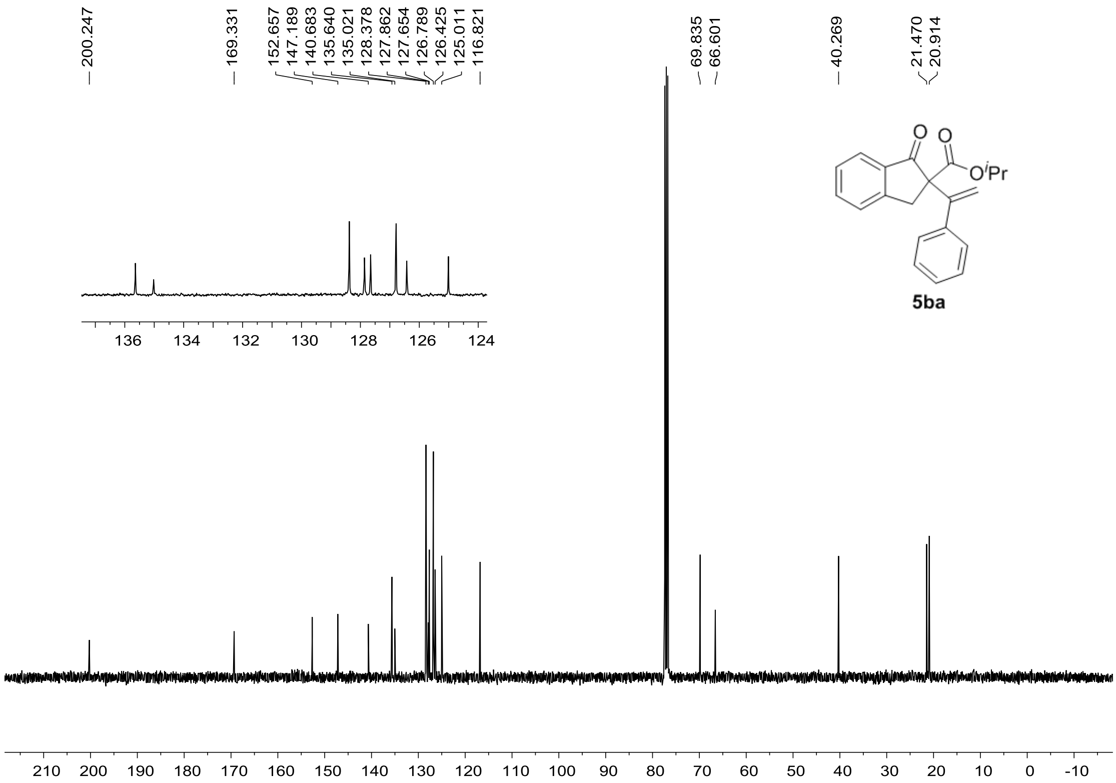

Supplementary Figure 76. ^{13}C NMR spectra for product **5ba**

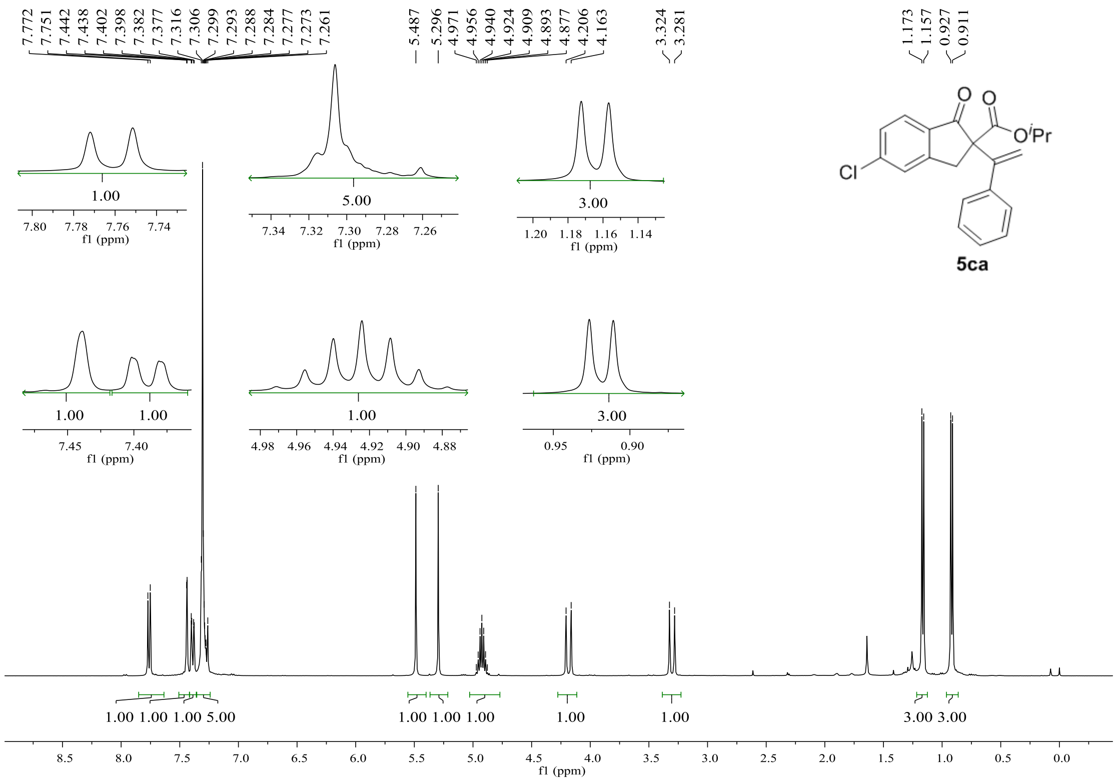

Supplementary Figure 77. ^{1}H NMR spectra for product **5ca**

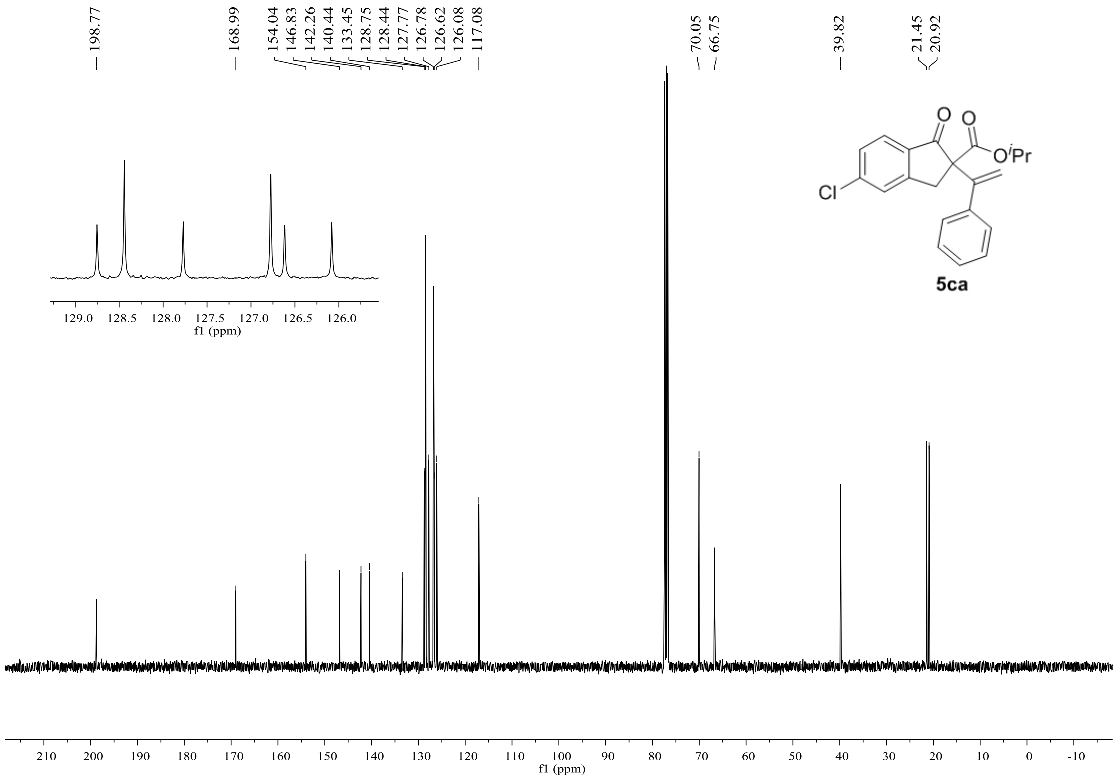

Supplementary Figure 78. ^{13}C NMR spectra for product **5ca**

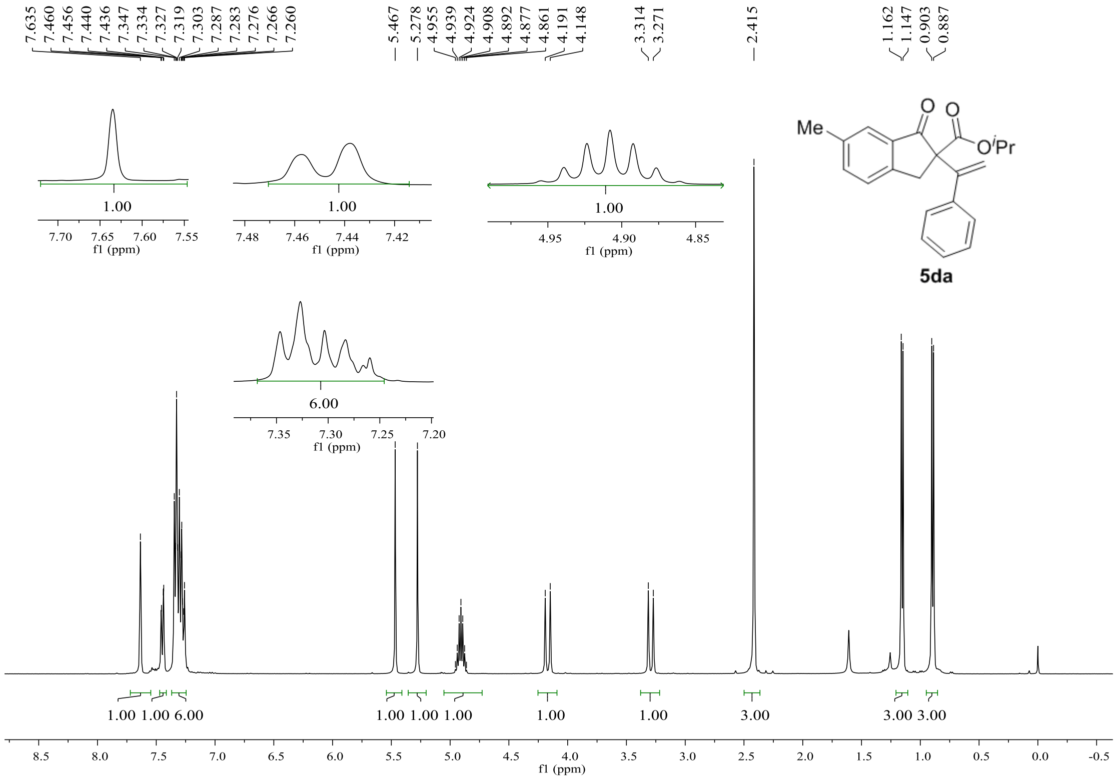

Supplementary Figure 79. ^{1}H NMR spectra for product **5da**

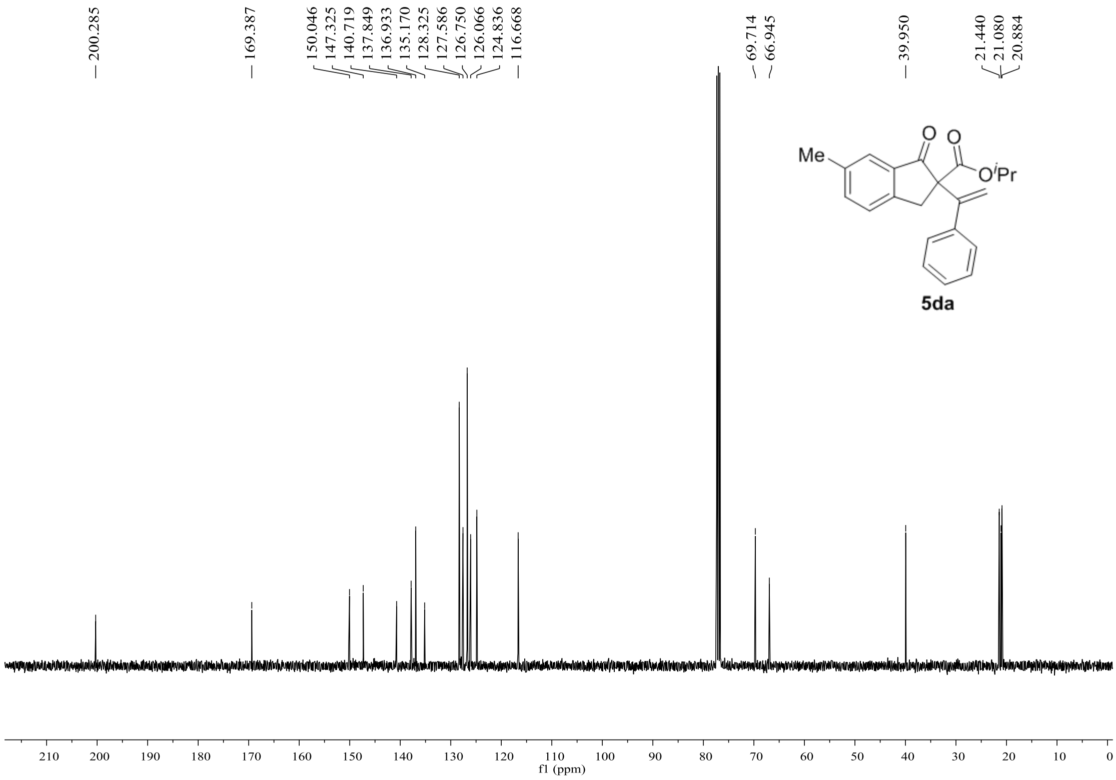

Supplementary Figure 80. ^{13}C NMR spectra for product **5da**

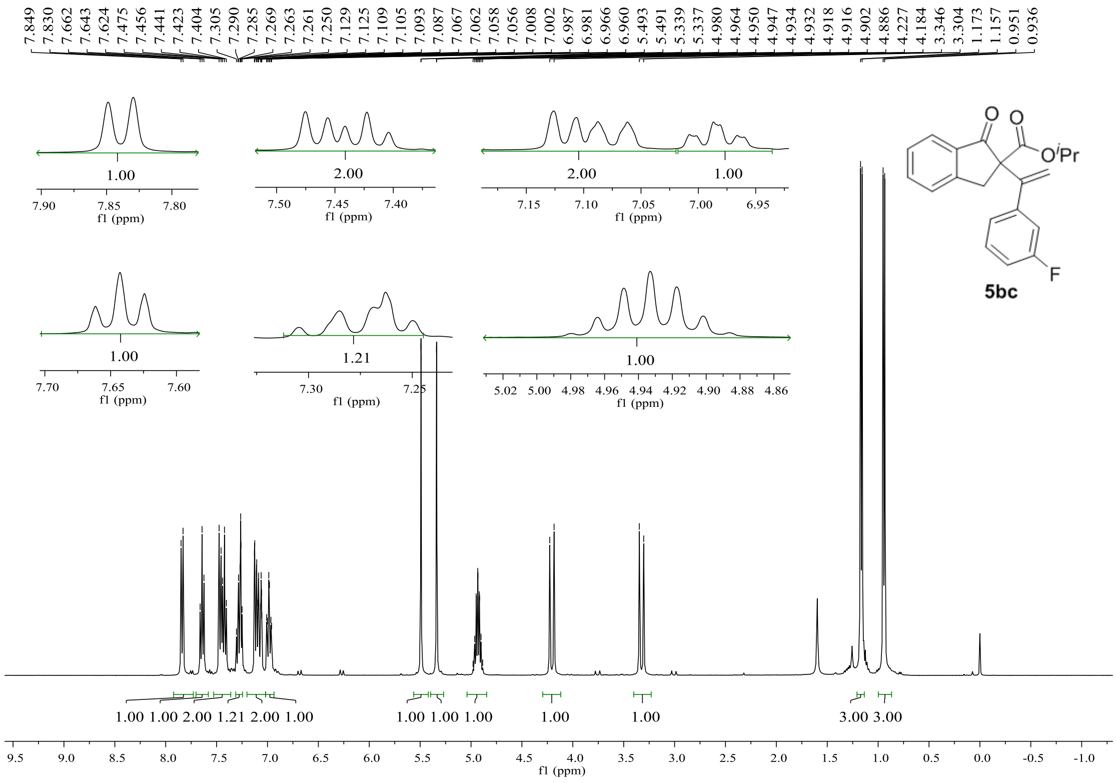

Supplementary Figure 81. ^{1}H NMR spectra for product **5bc**

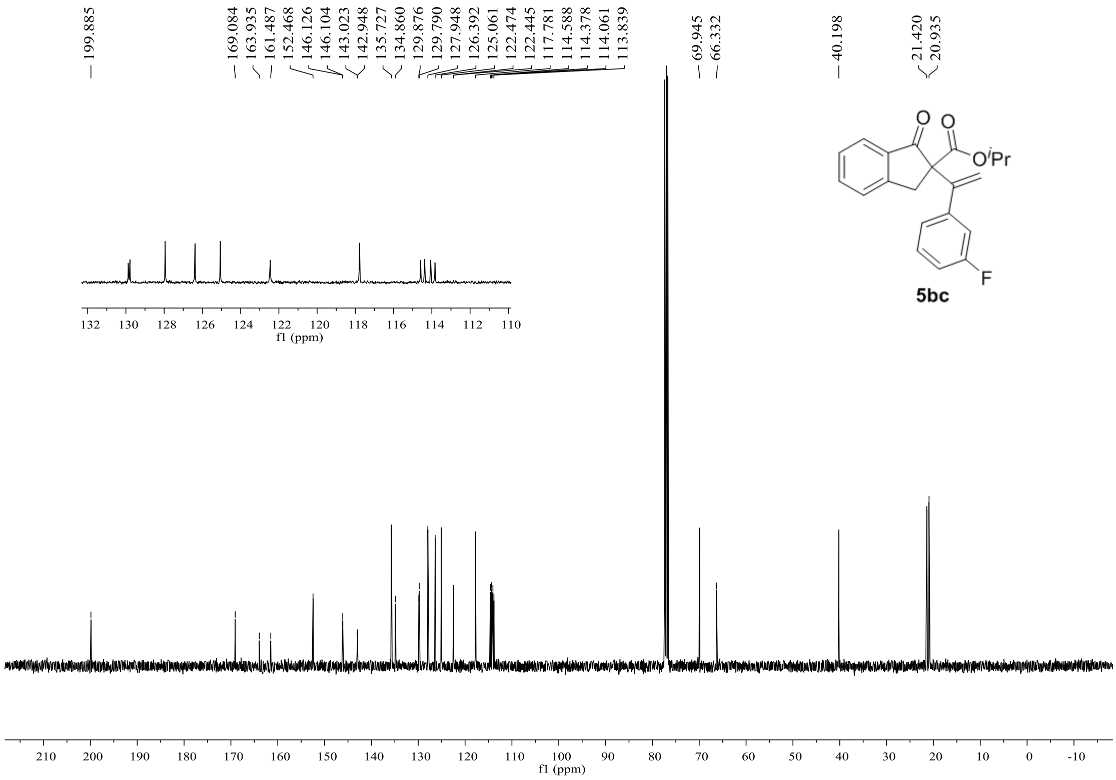

Supplementary Figure 82. ^{13}C NMR spectra for product **5bc**

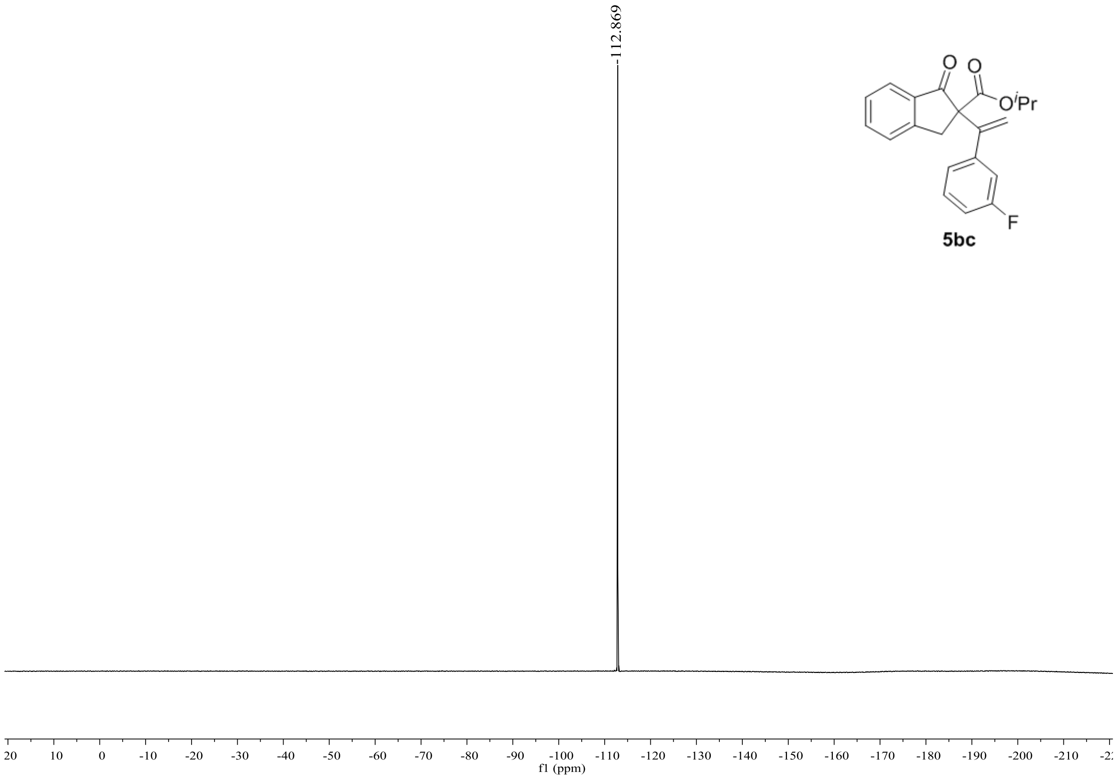

Supplementary Figure 83. ^{19}F NMR spectra for product **5bc**

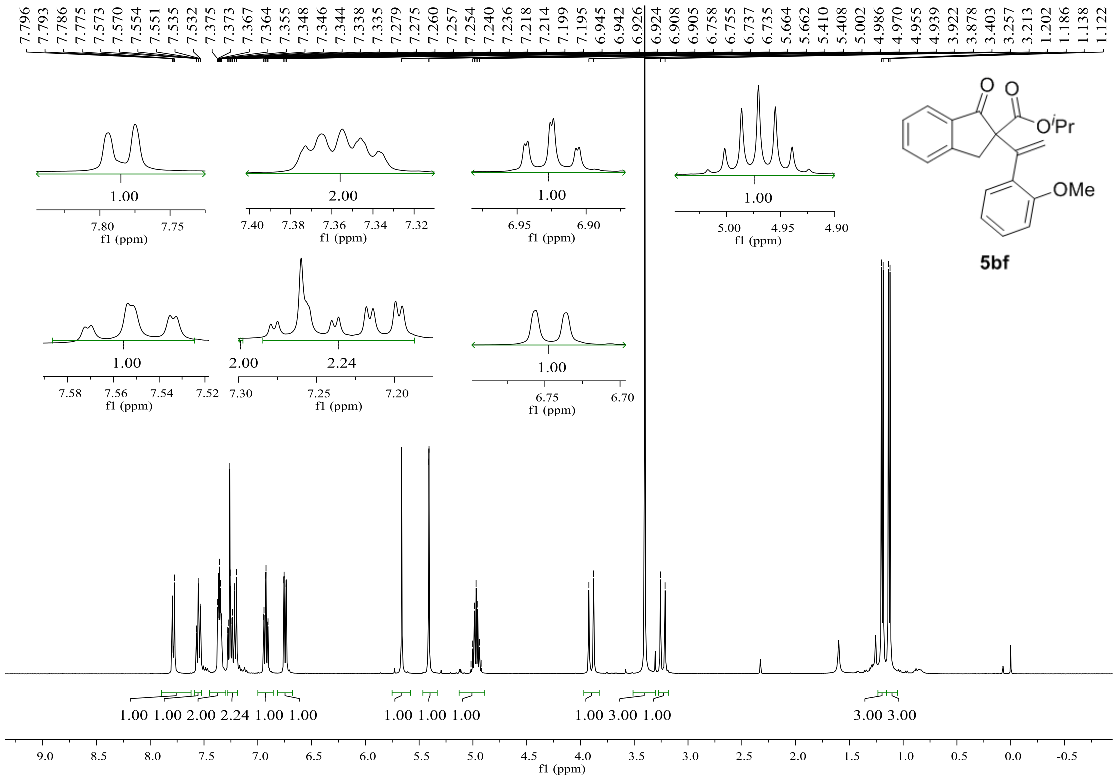

Supplementary Figure 84. ^{1}H NMR spectra for product **5bf**

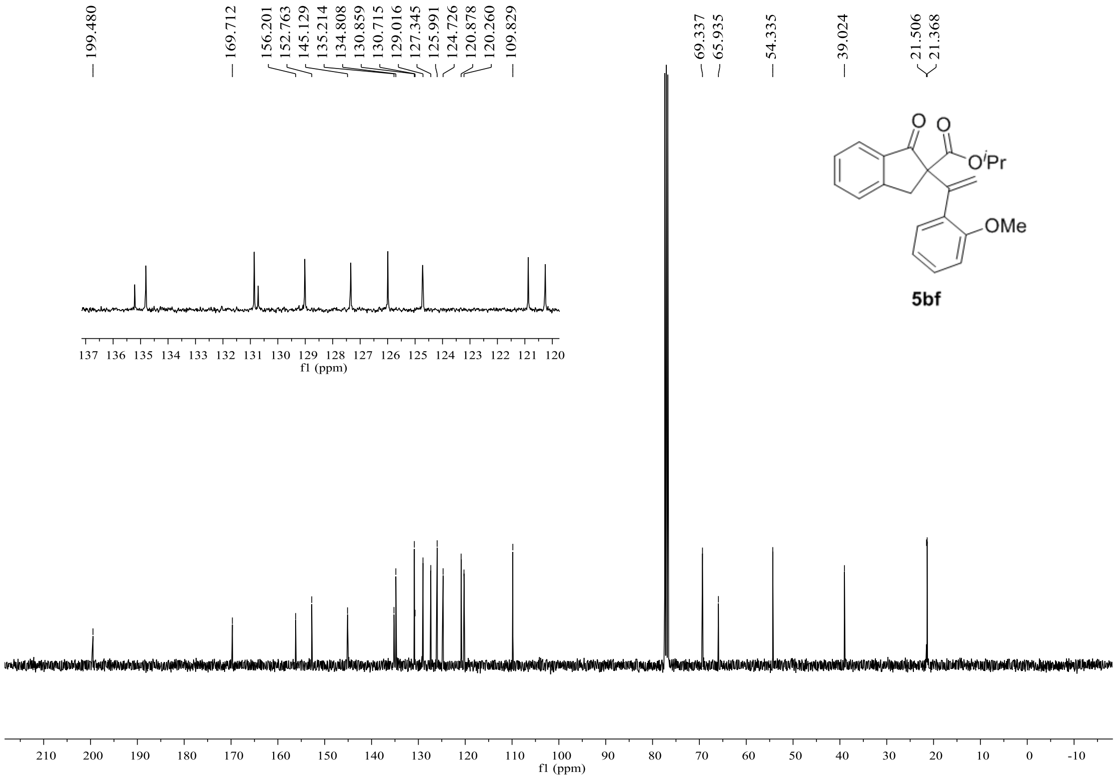

Supplementary Figure 85. ^{13}C NMR spectra for product **5bf**

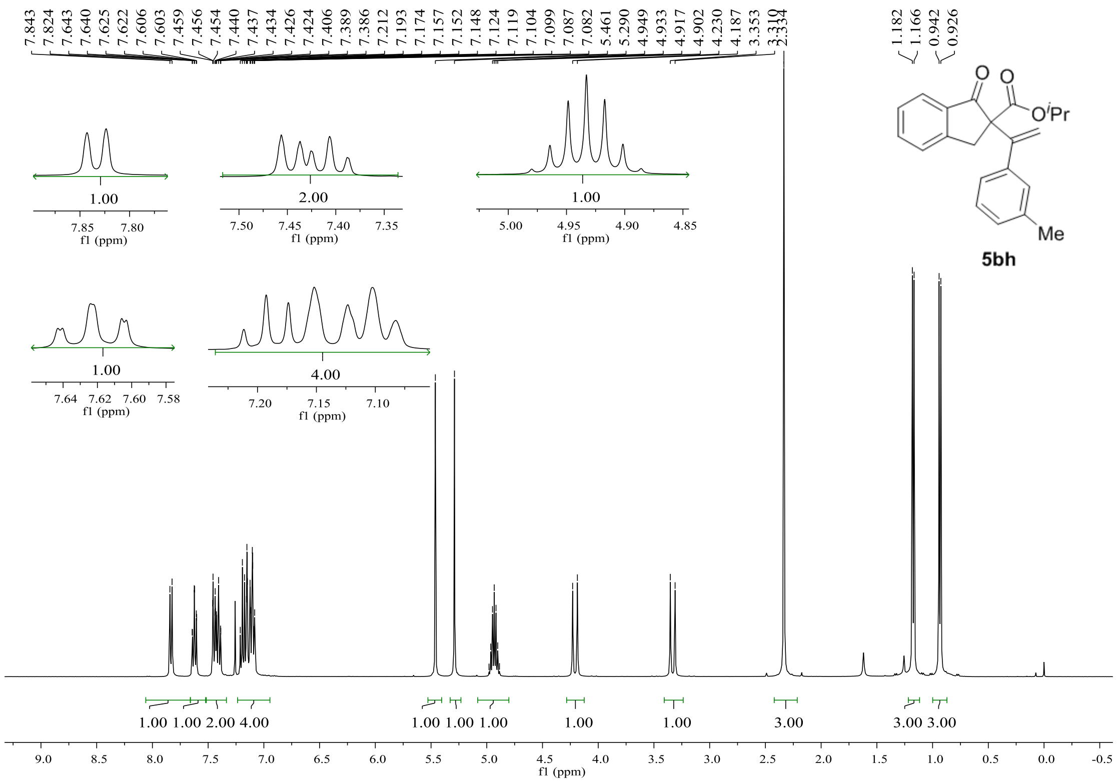

Supplementary Figure 86. ^{1}H NMR spectra for product **5bh**

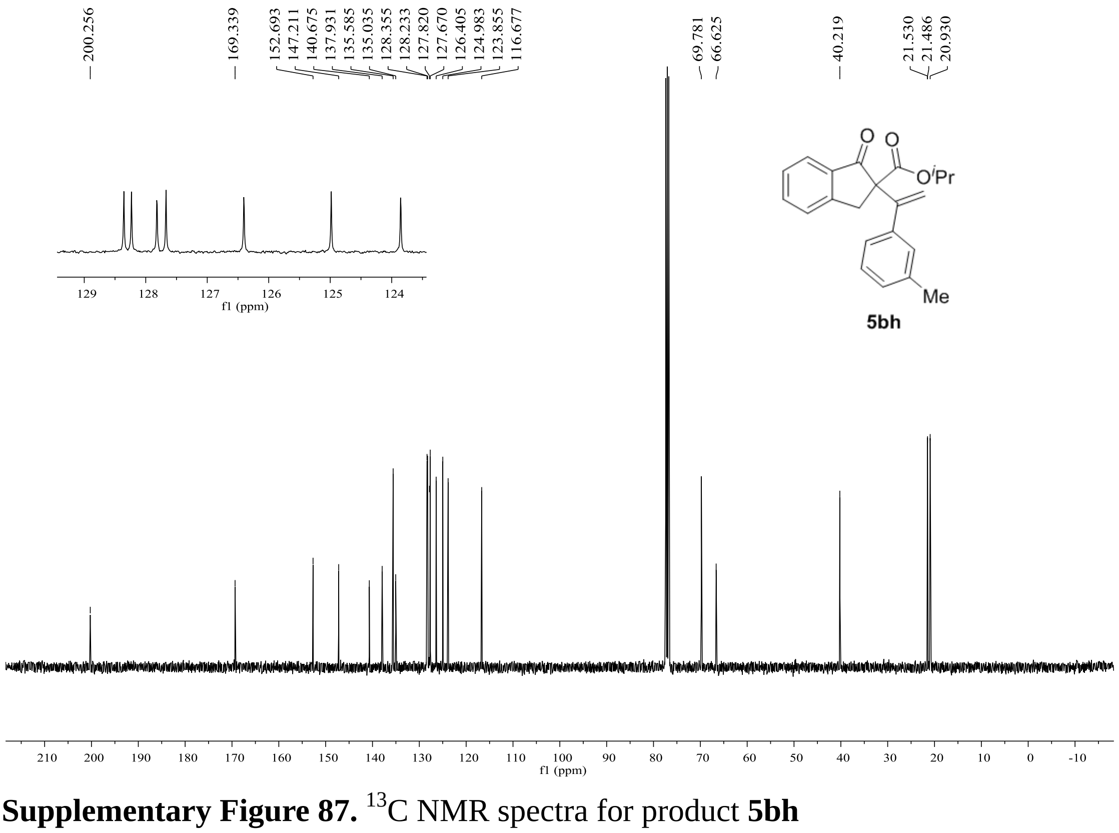

Supplementary Figure 87. ^{13}C NMR spectra for product **5bh**

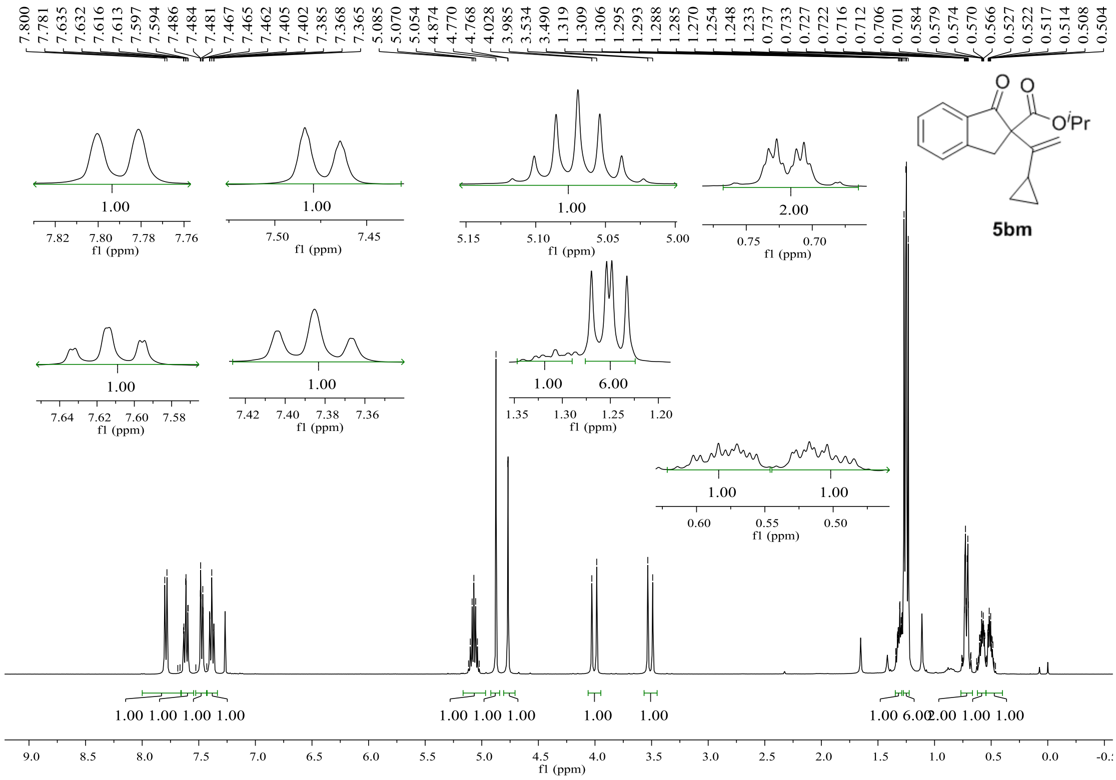

Supplementary Figure 88. ^{1}H NMR spectra for product **5bm**

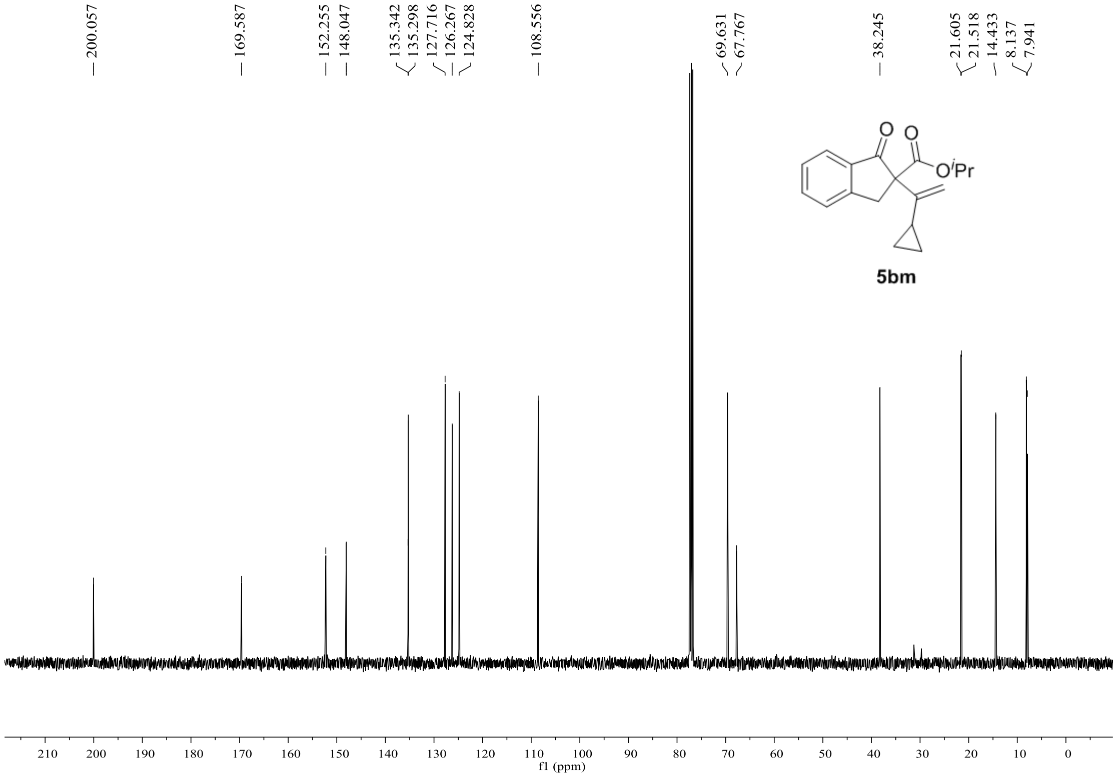

Supplementary Figure 89. ^{13}C NMR spectra for product **5bm**

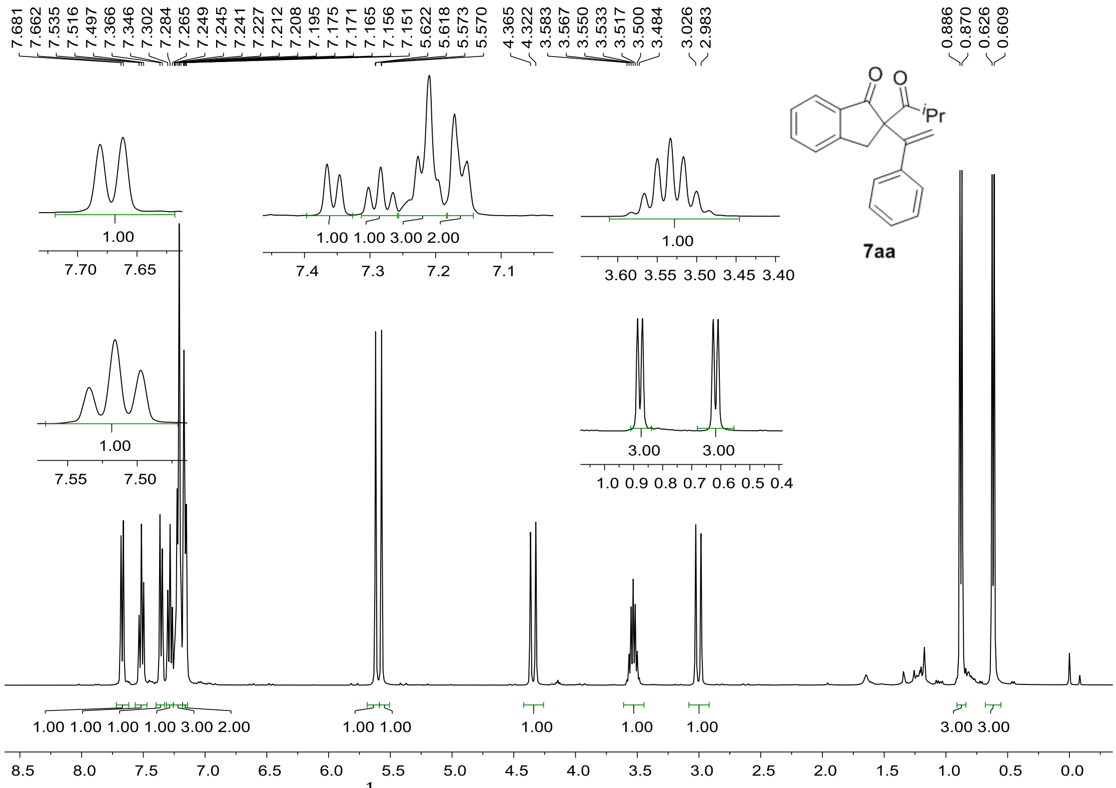

Supplementary Figure 90. ^{1}H NMR spectra for product **7aa**

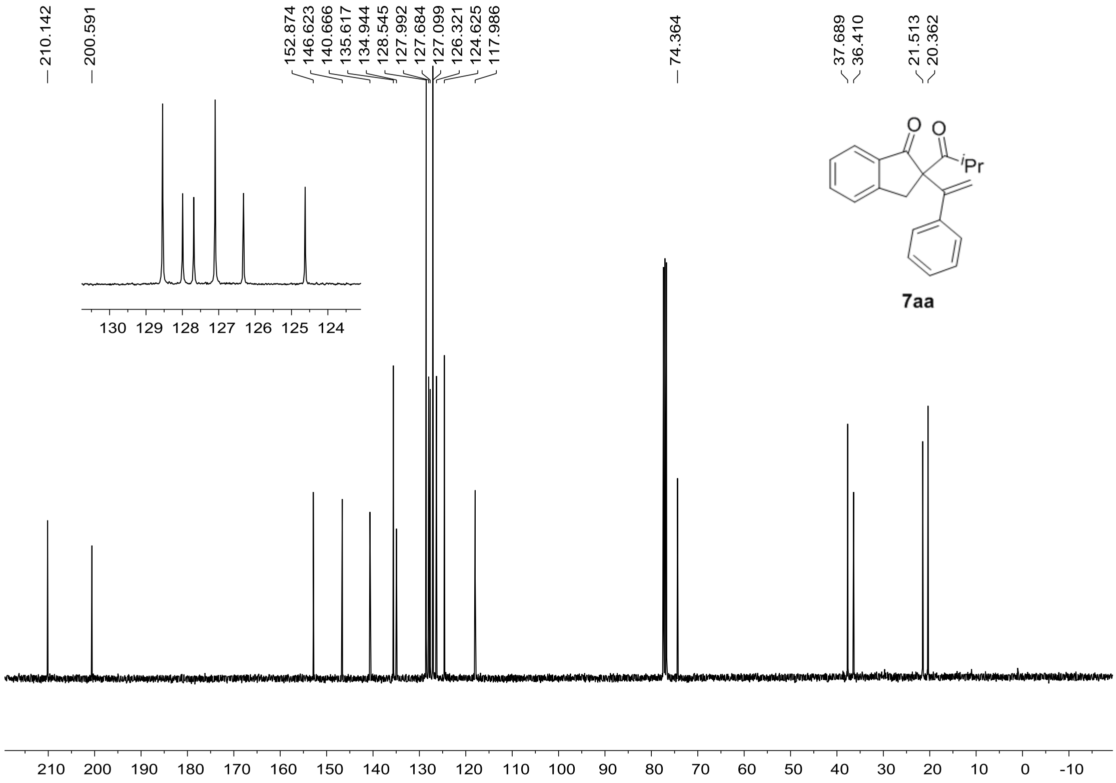

Supplementary Figure 91. ^{13}C NMR spectra for product **7aa**

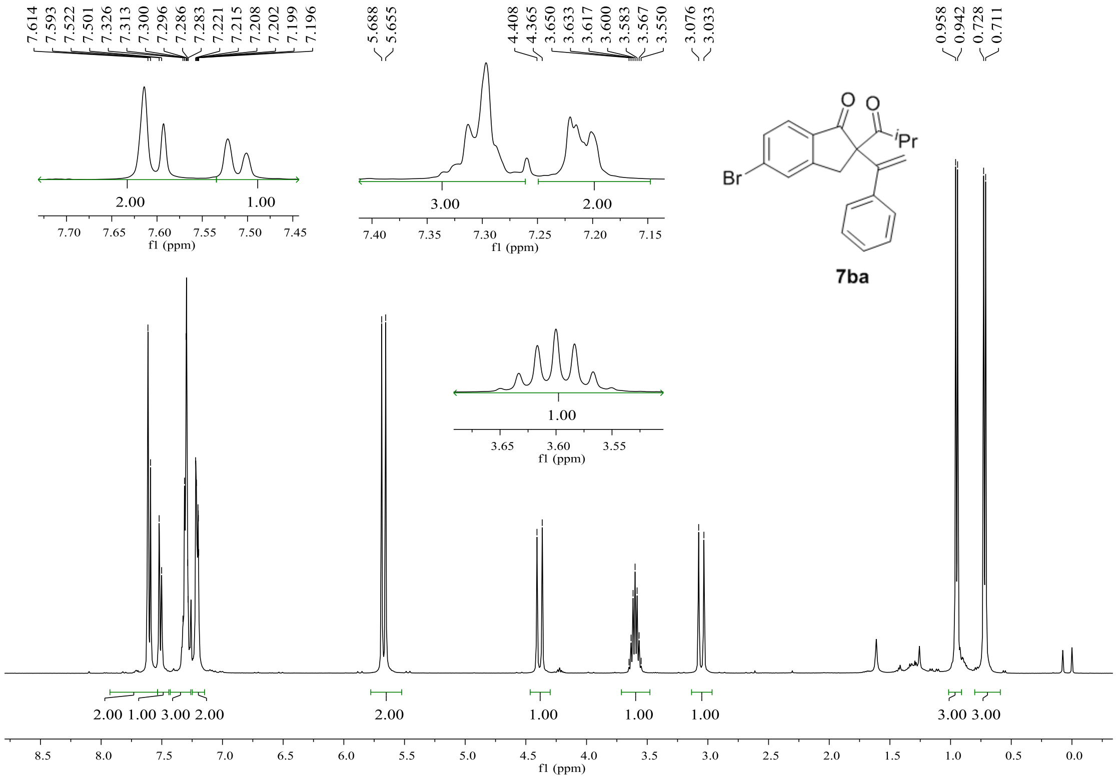

Supplementary Figure 92. ^{1}H NMR spectra for product **7ba**

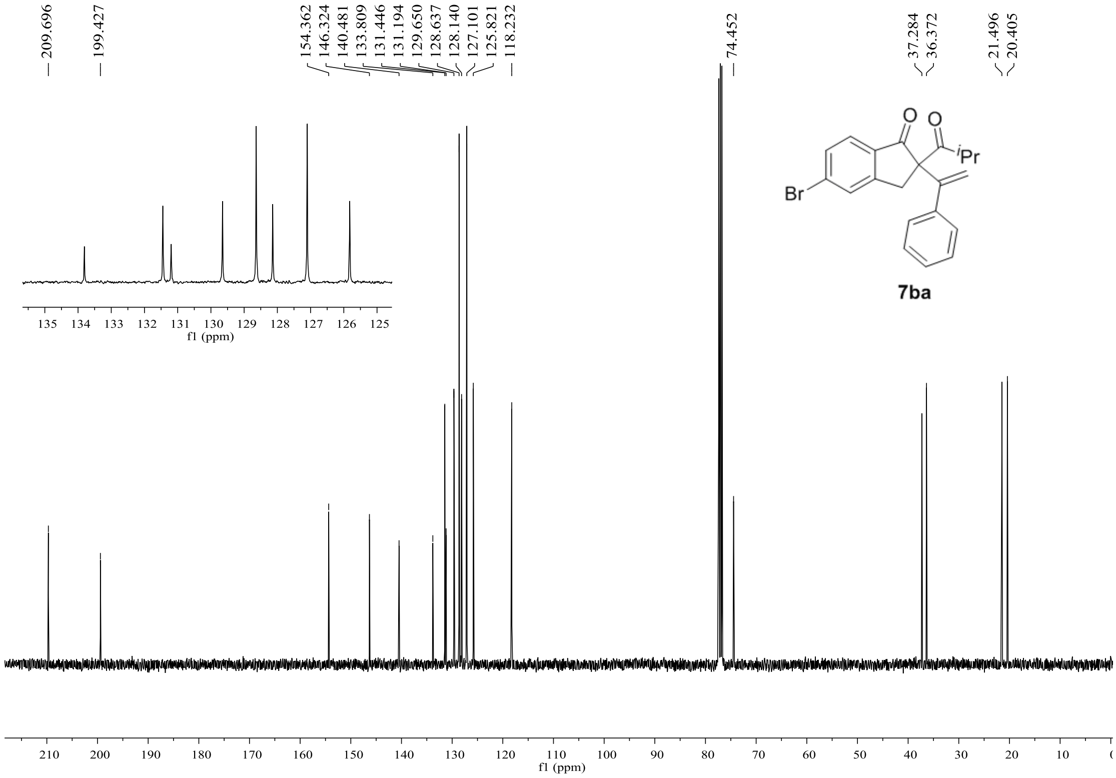

Supplementary Figure 93. ^{13}C NMR spectra for product **7ba**

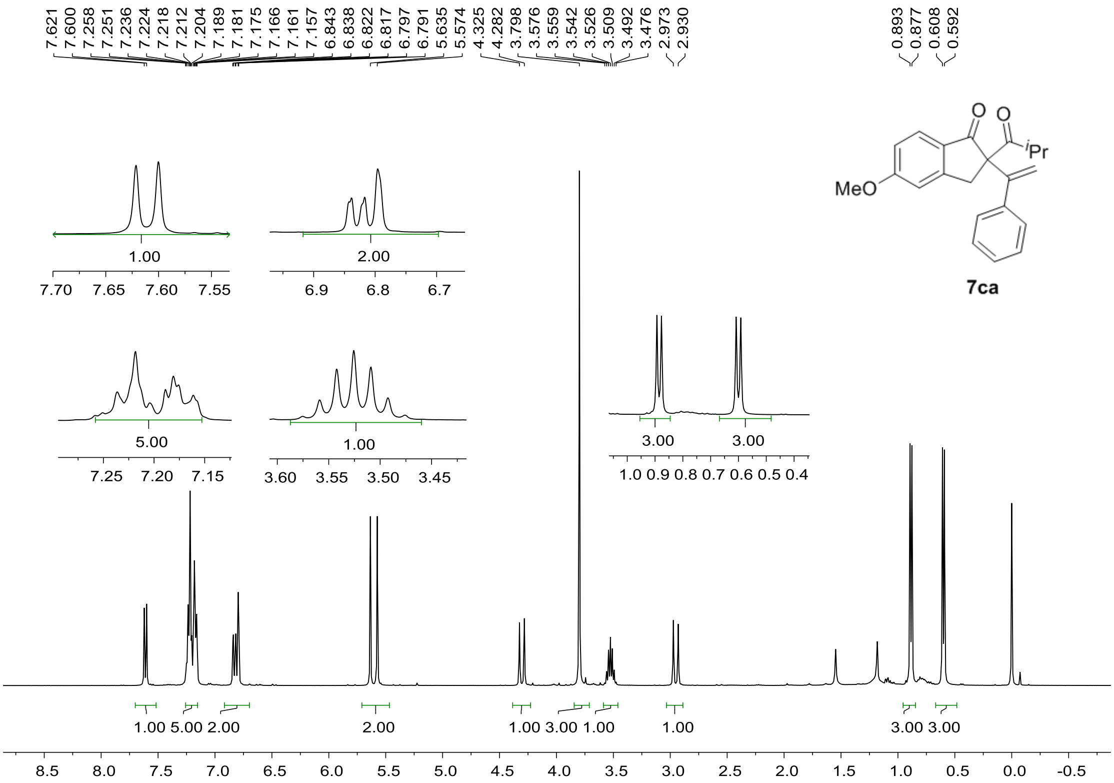

Supplementary Figure 94. ^{1}H NMR spectra for product **7ca**

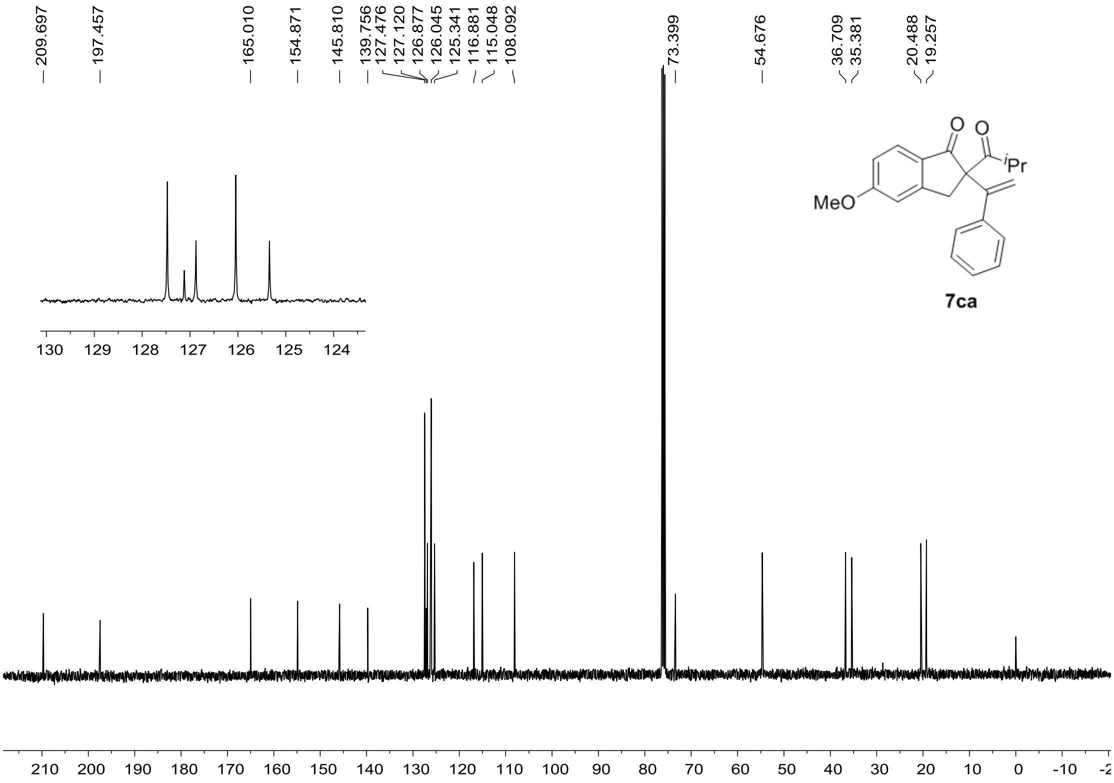

Supplementary Figure 95. ^{13}C NMR spectra for product **7ca**

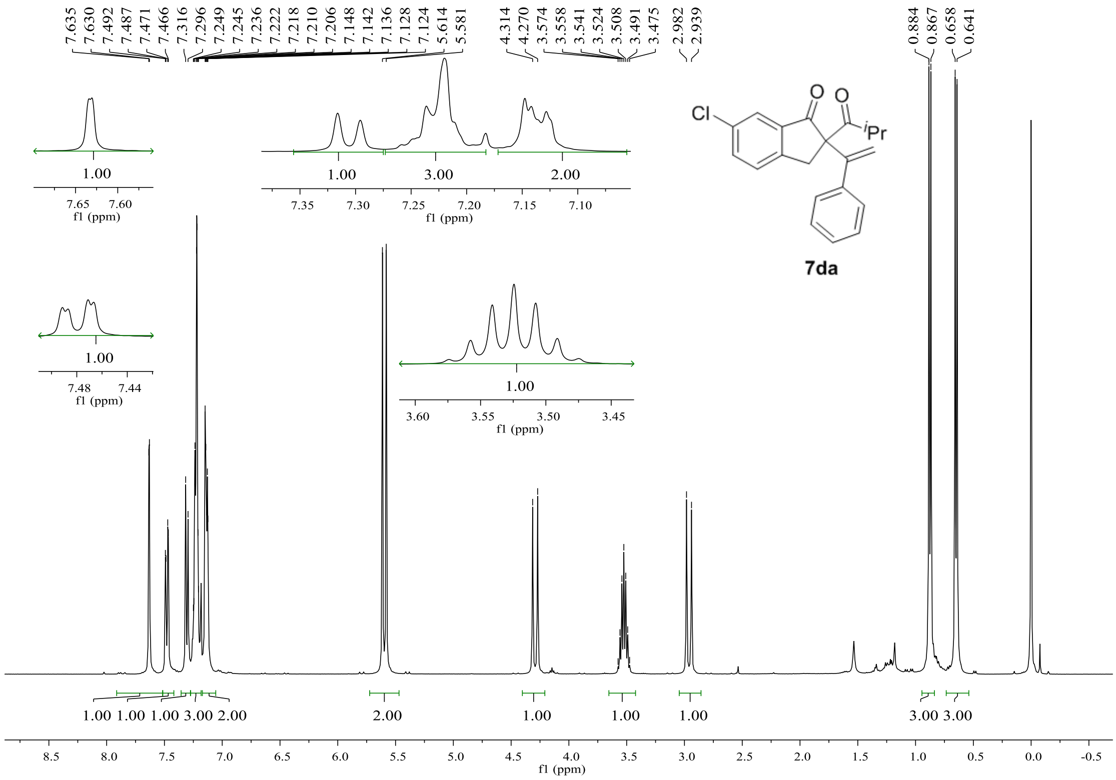

Supplementary Figure 96. ^{1}H NMR spectra for product **7da**

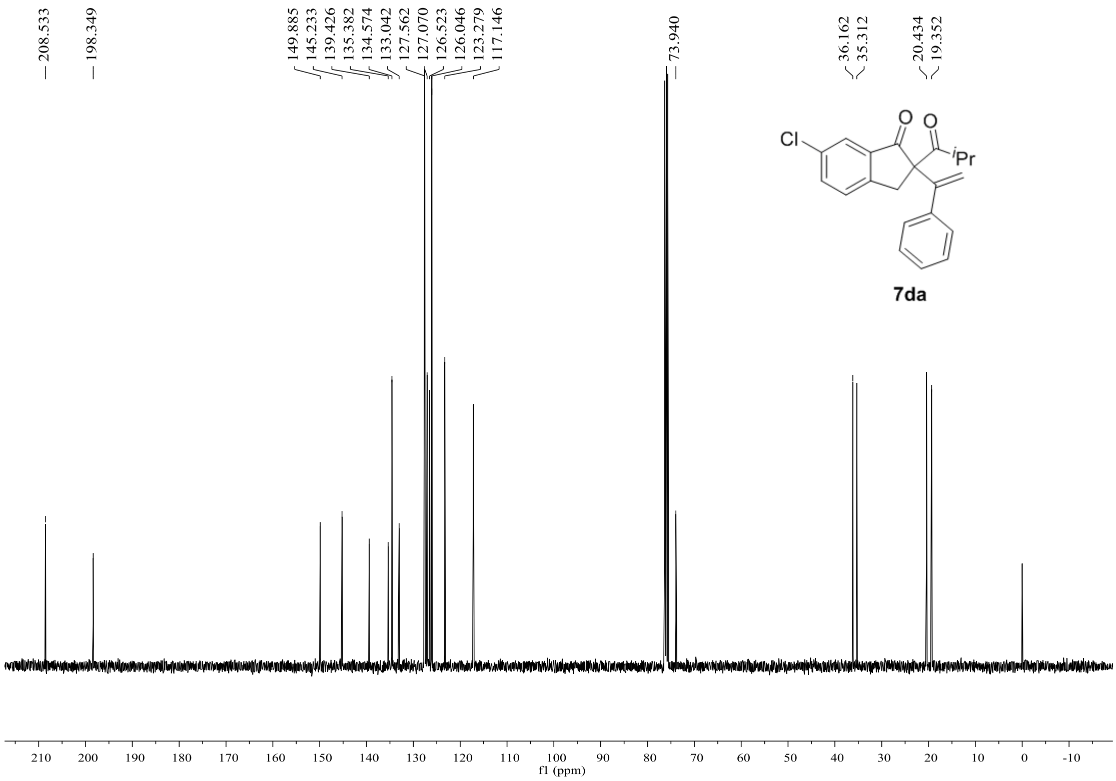

Supplementary Figure 97. ^{13}C NMR spectra for product **7da**

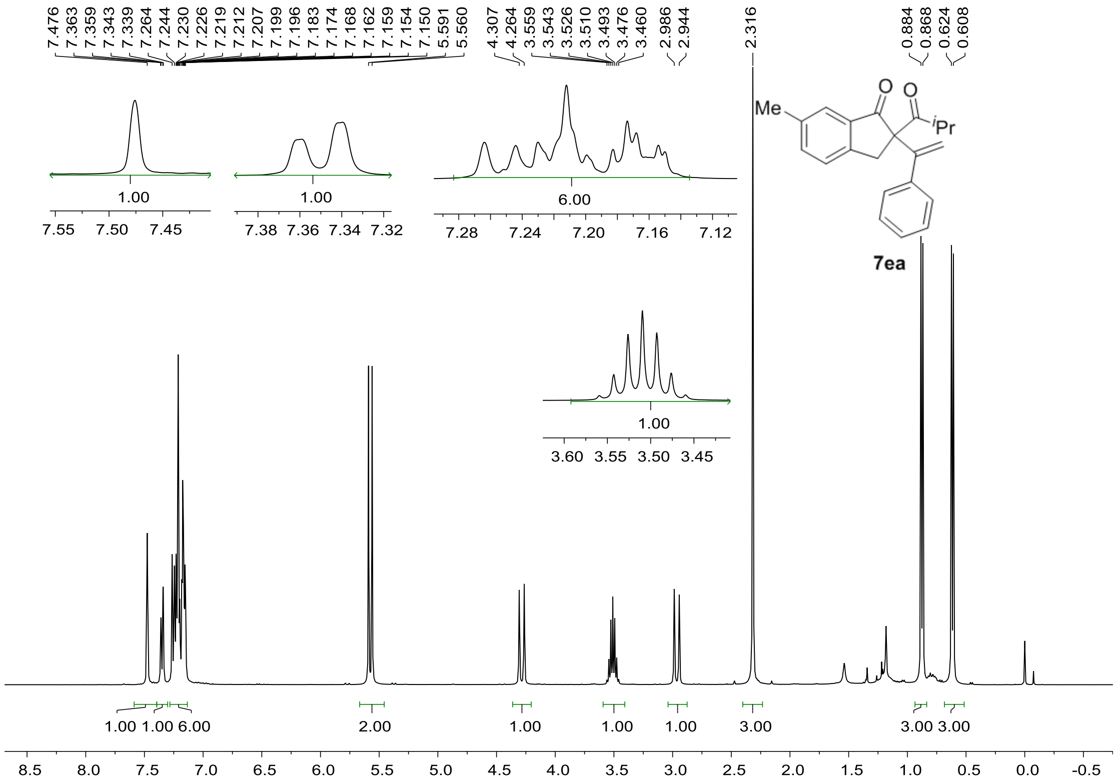

Supplementary Figure 98. ^{1}H NMR spectra for product **7ea**

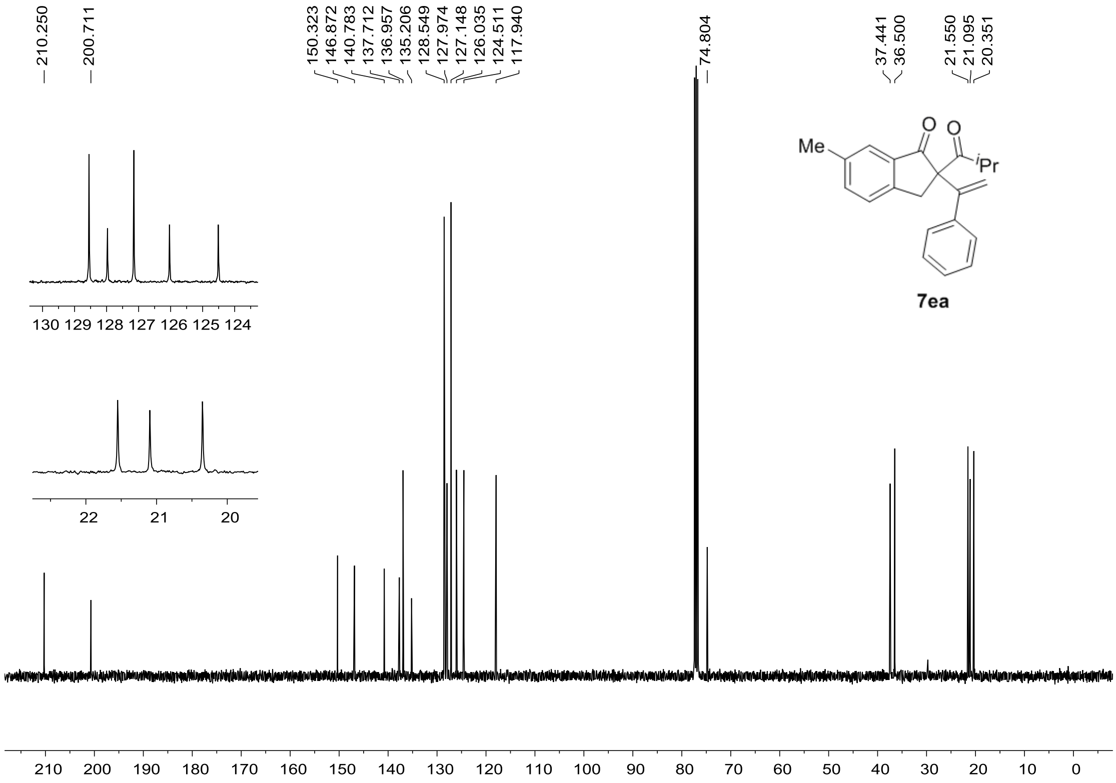

Supplementary Figure 99. ^{13}C NMR spectra for product **7ea**

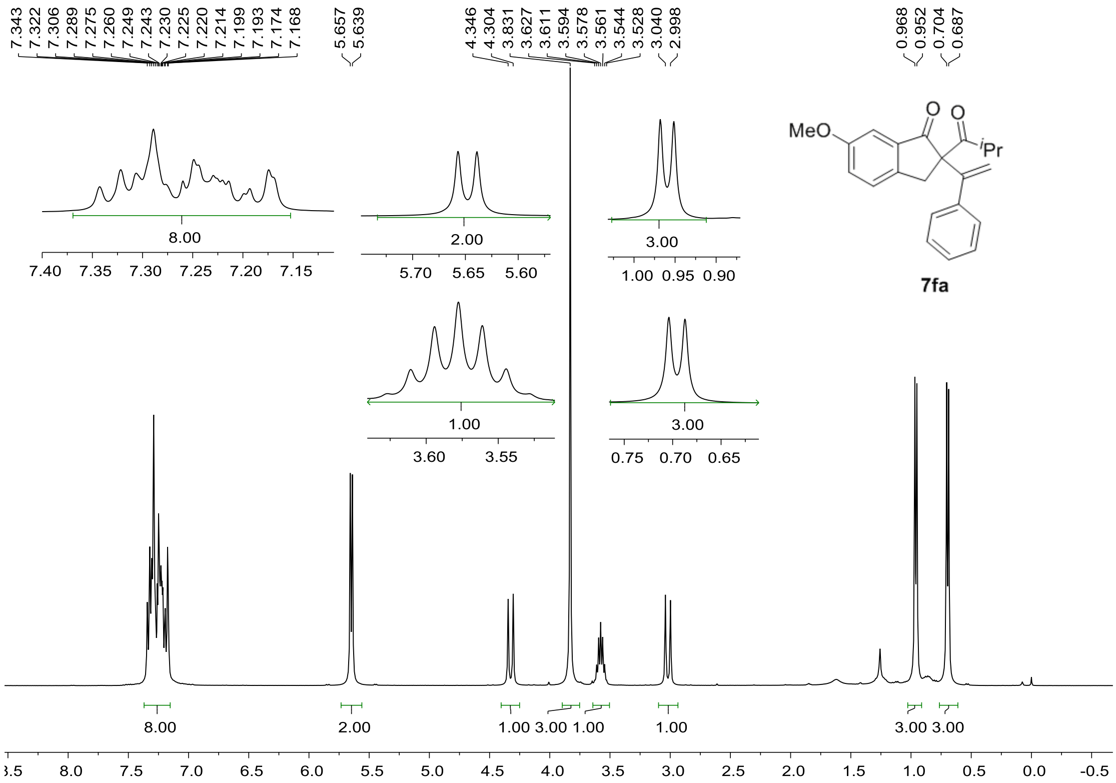

Supplementary Figure 100. ^{1}H NMR spectra for product **7fa**

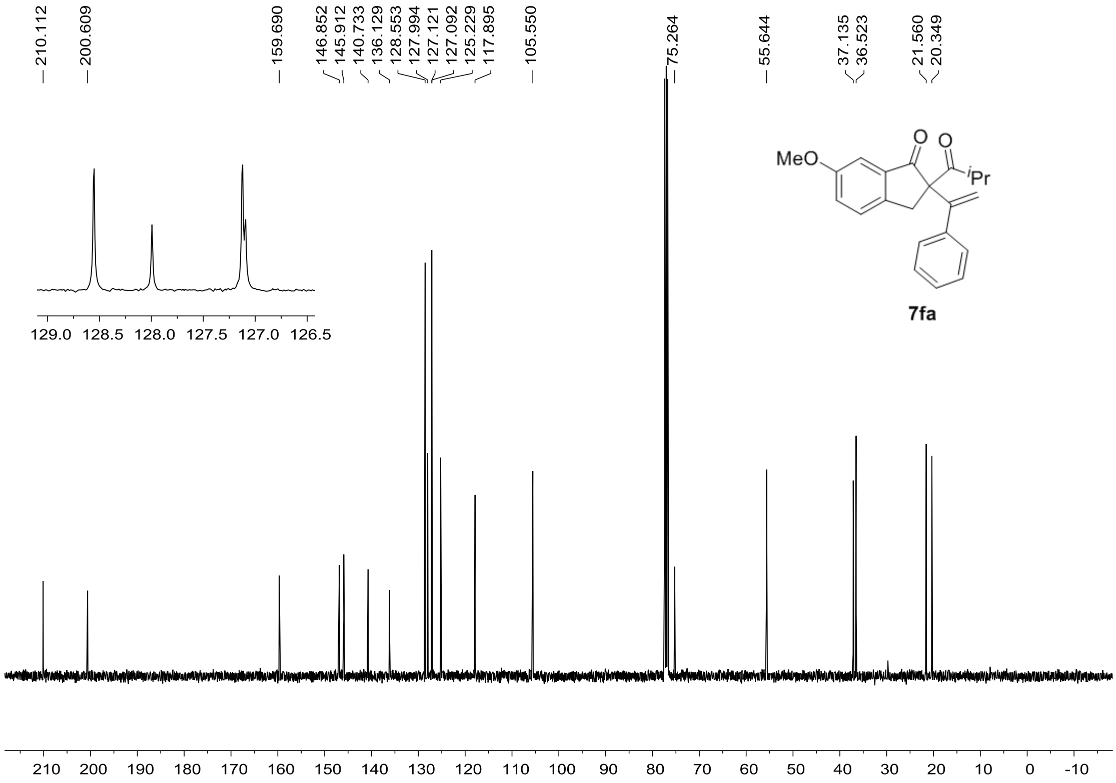

Supplementary Figure 101. ^{13}C NMR spectra for product **7fa**

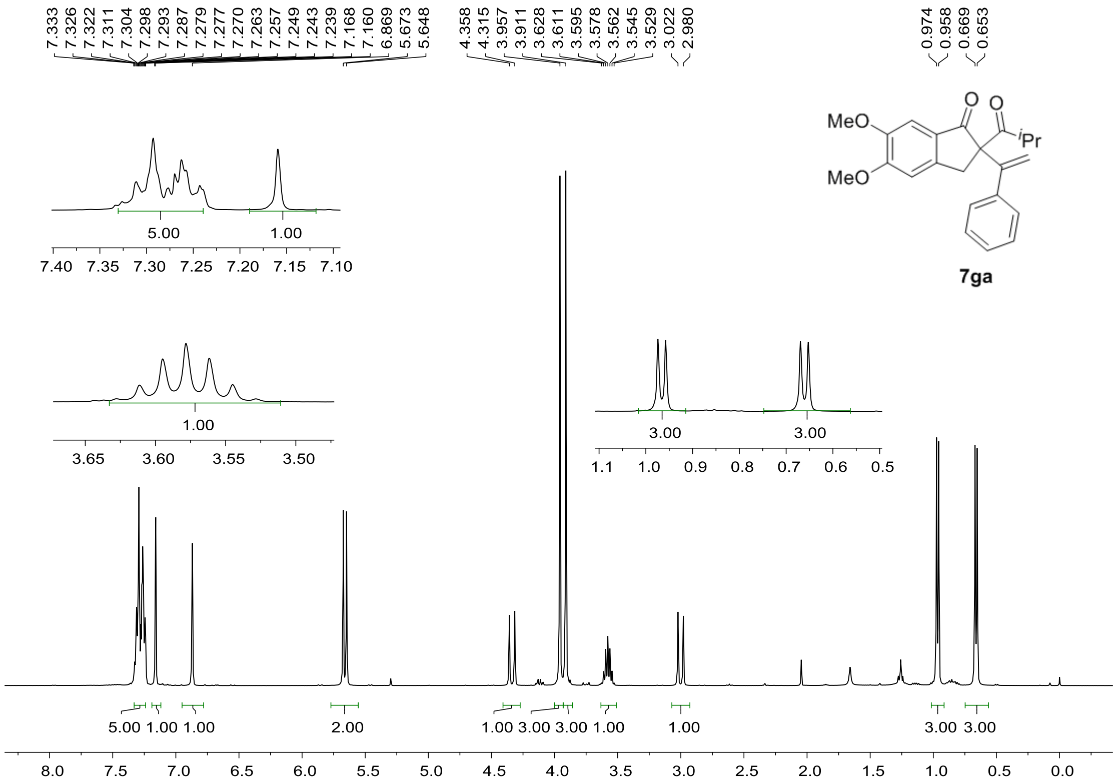

Supplementary Figure 102. ^{1}H NMR spectra for product **7ga**

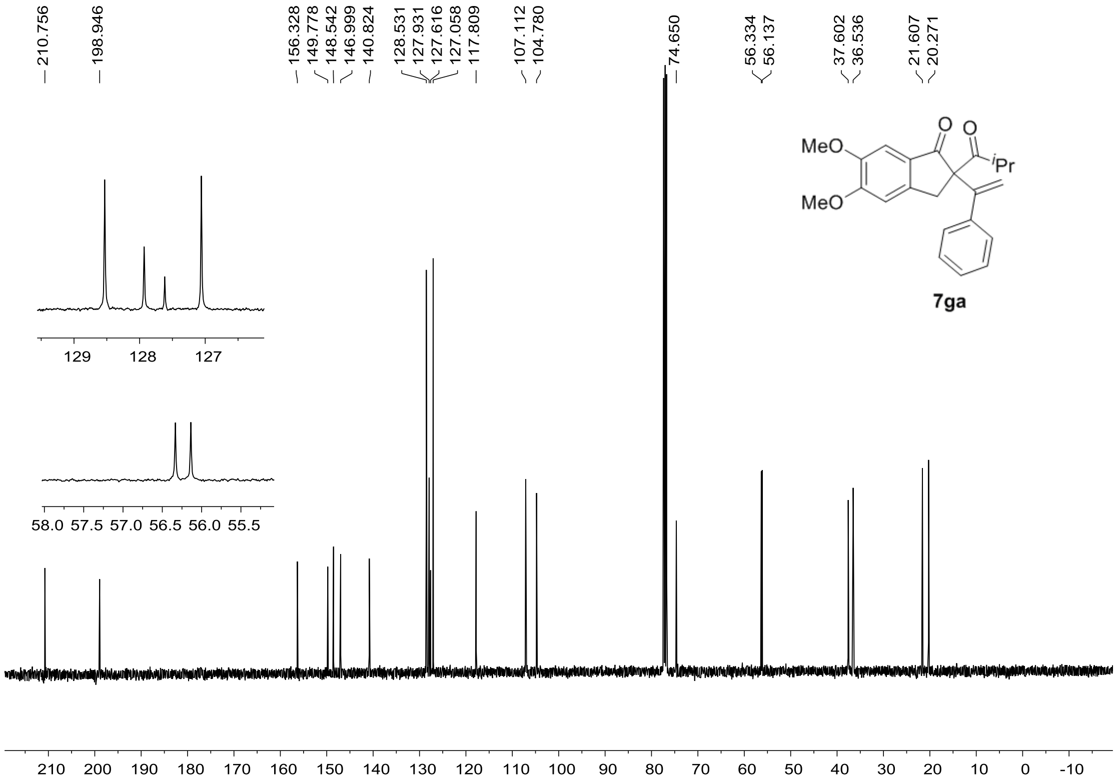

Supplementary Figure 103. ^{13}C NMR spectra for product **7ga**

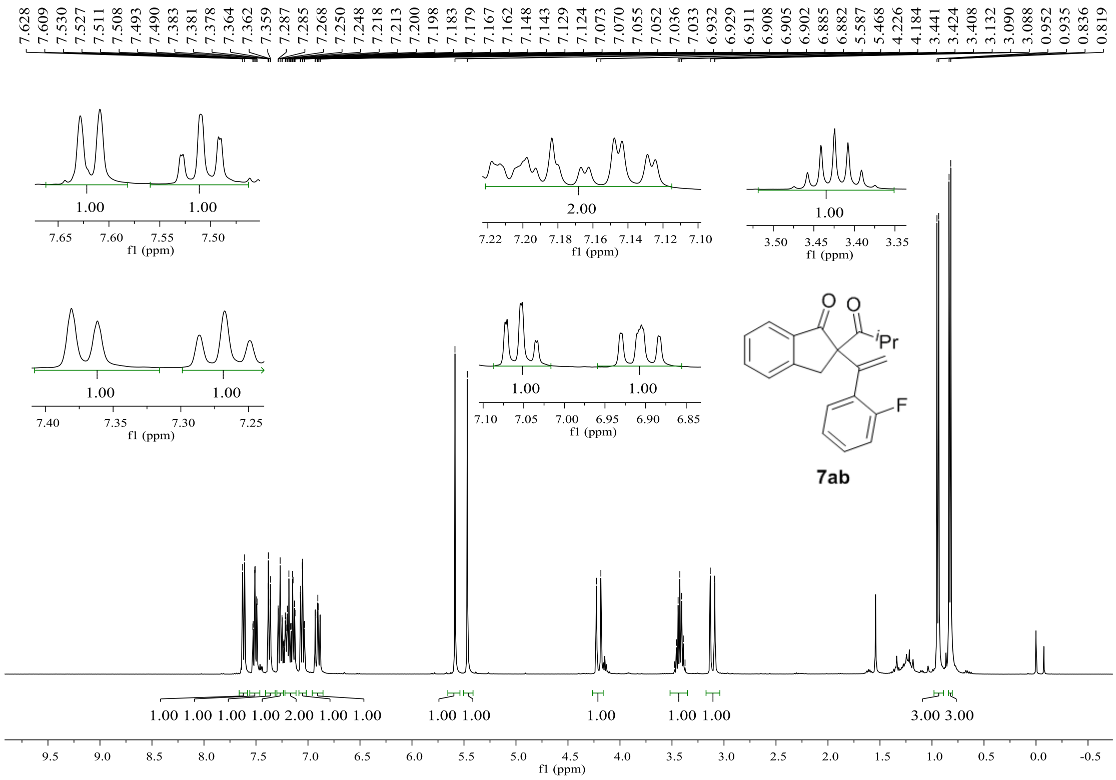

Supplementary Figure 104. ^{1}H NMR spectra for product **7ab**

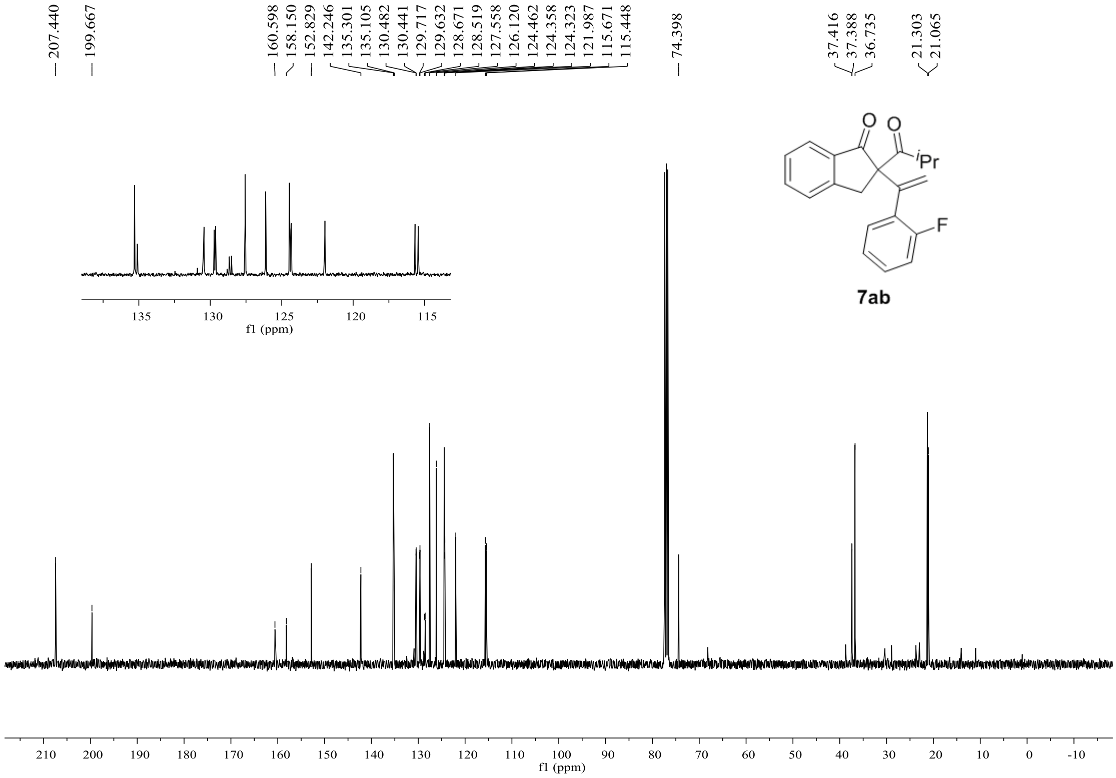

Supplementary Figure 105. ^{13}C NMR spectra for product **7ab**

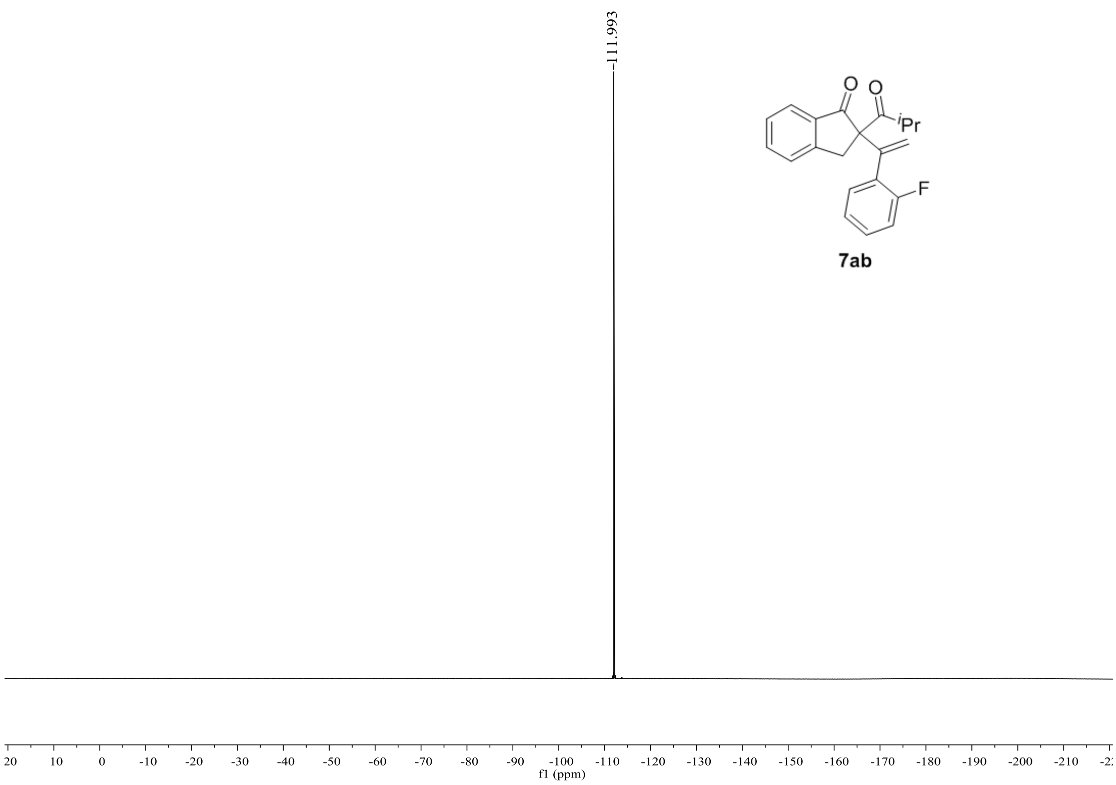

Supplementary Figure 106. ^{19}F NMR spectra for product **7ab**

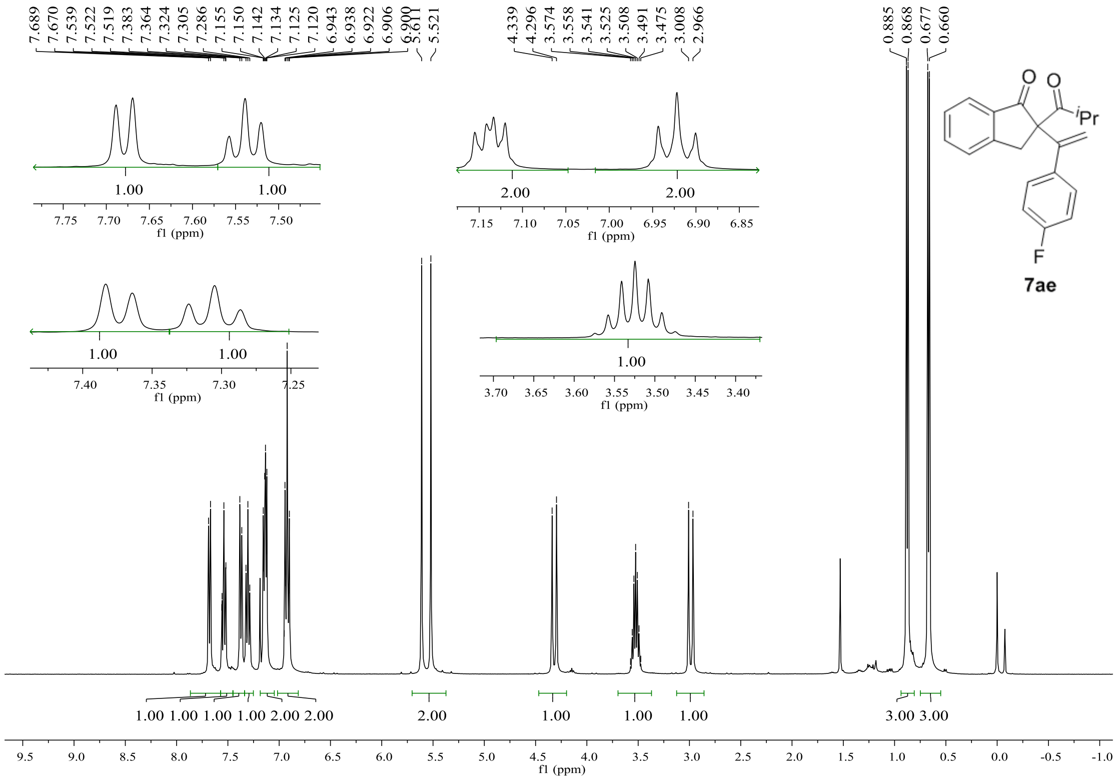

Supplementary Figure 107. ^{1}H NMR spectra for product **7ae**

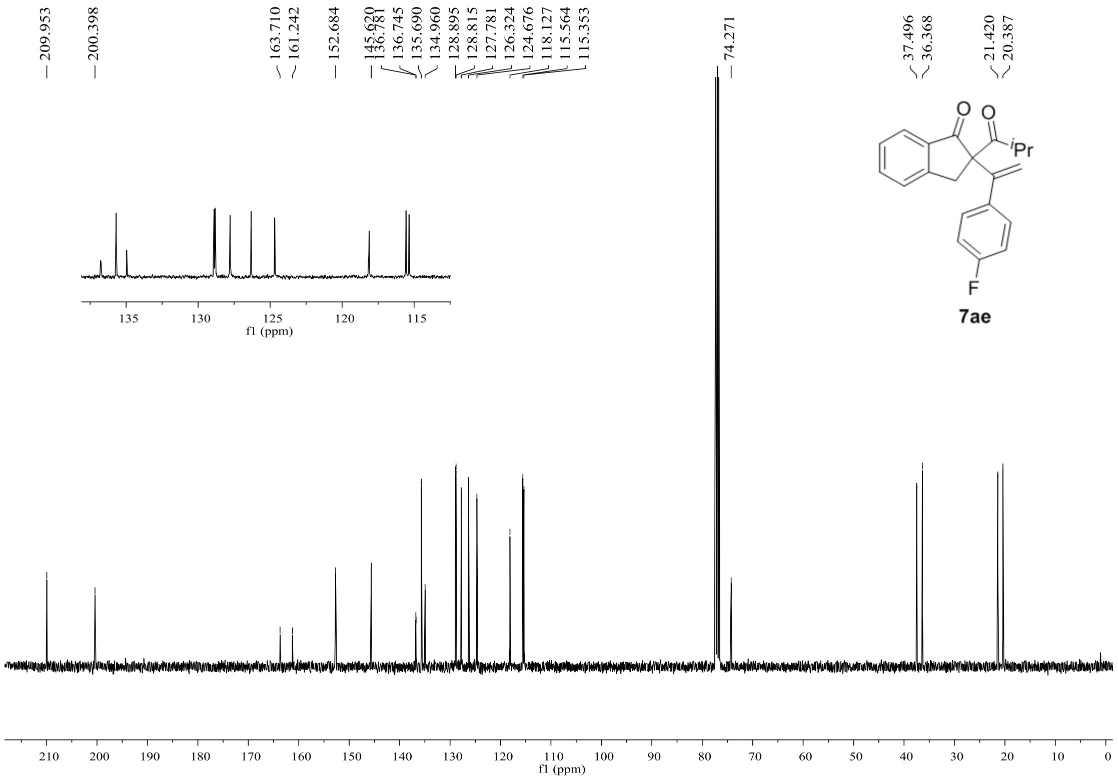

Supplementary Figure 108. ^{13}C NMR spectra for product **7ae**

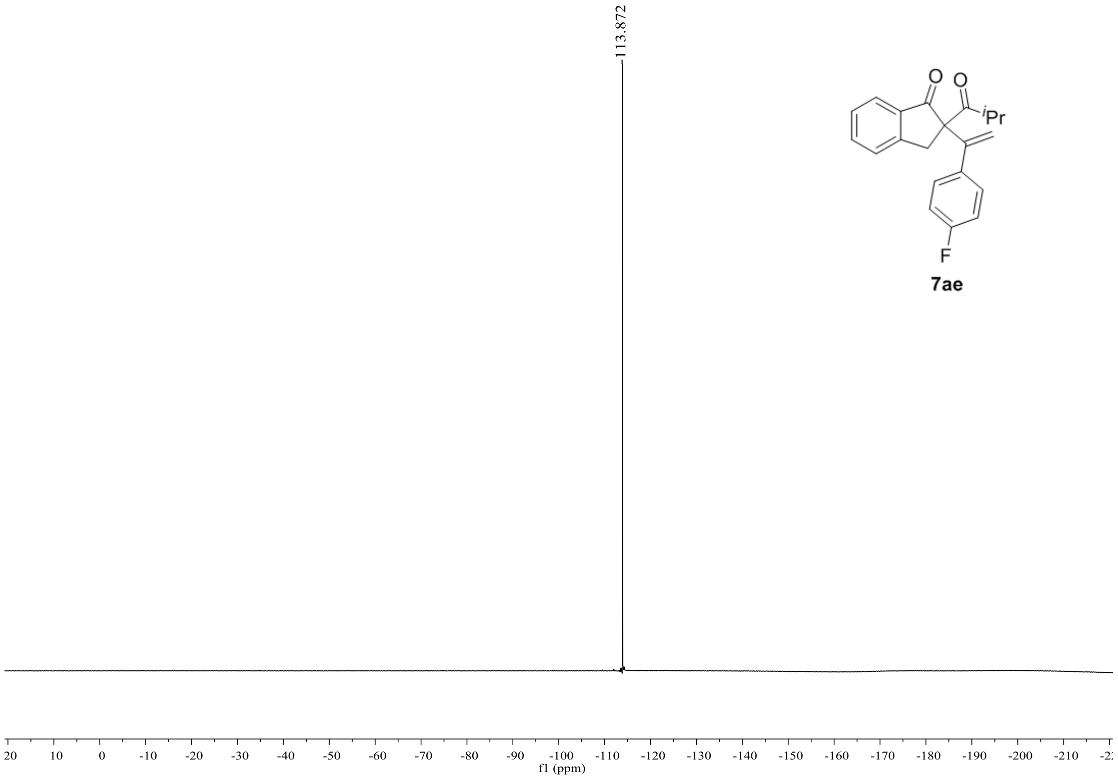

Supplementary Figure 109. ^{19}F NMR spectra for product **7ae**

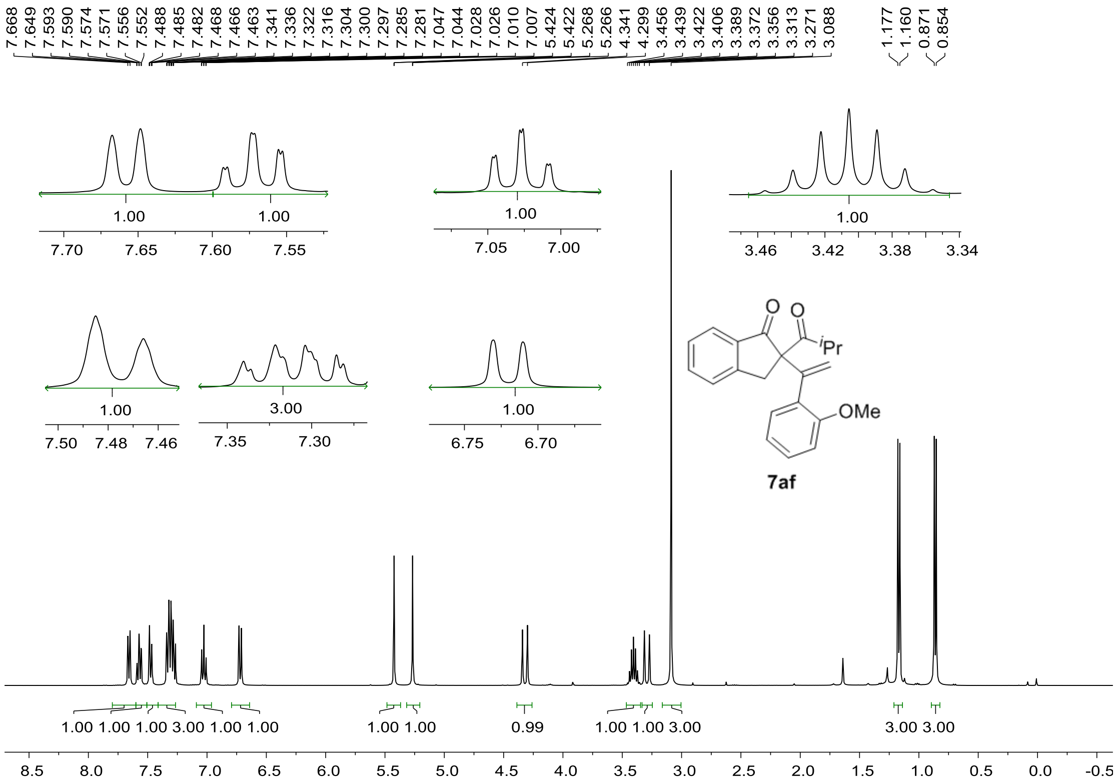

Supplementary Figure 110. ^{1}H NMR spectra for product **7af**

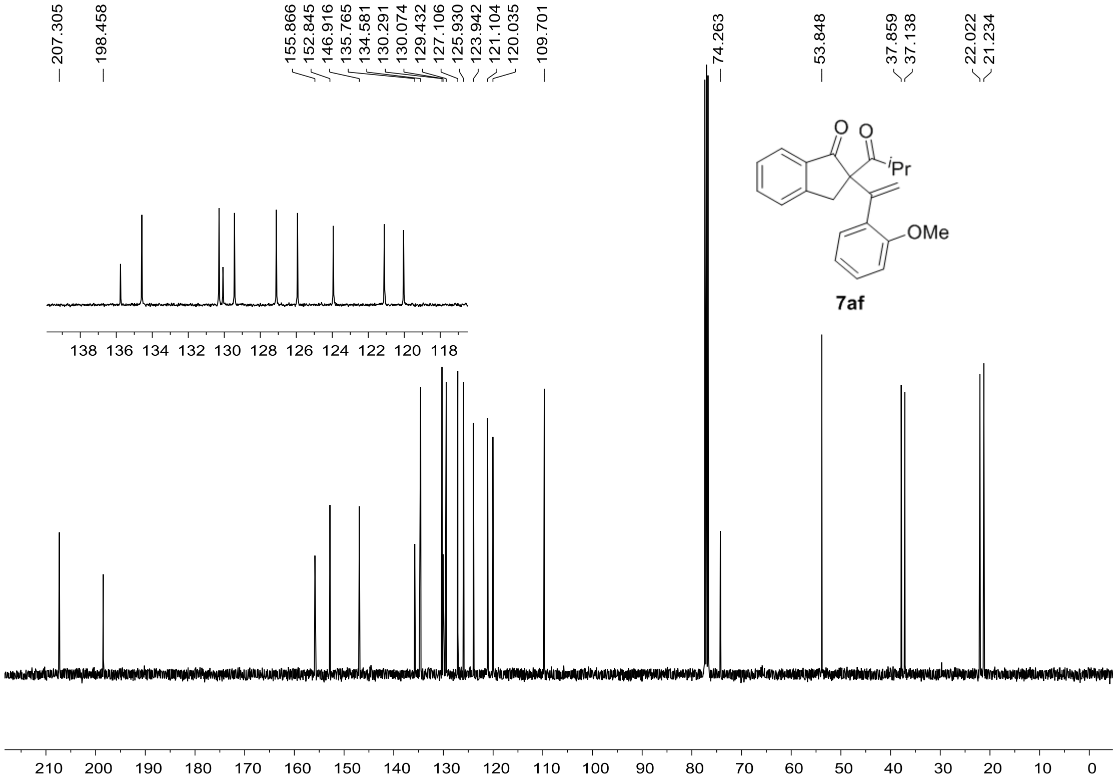

Supplementary Figure 111. ^{13}C NMR spectra for product **7af**

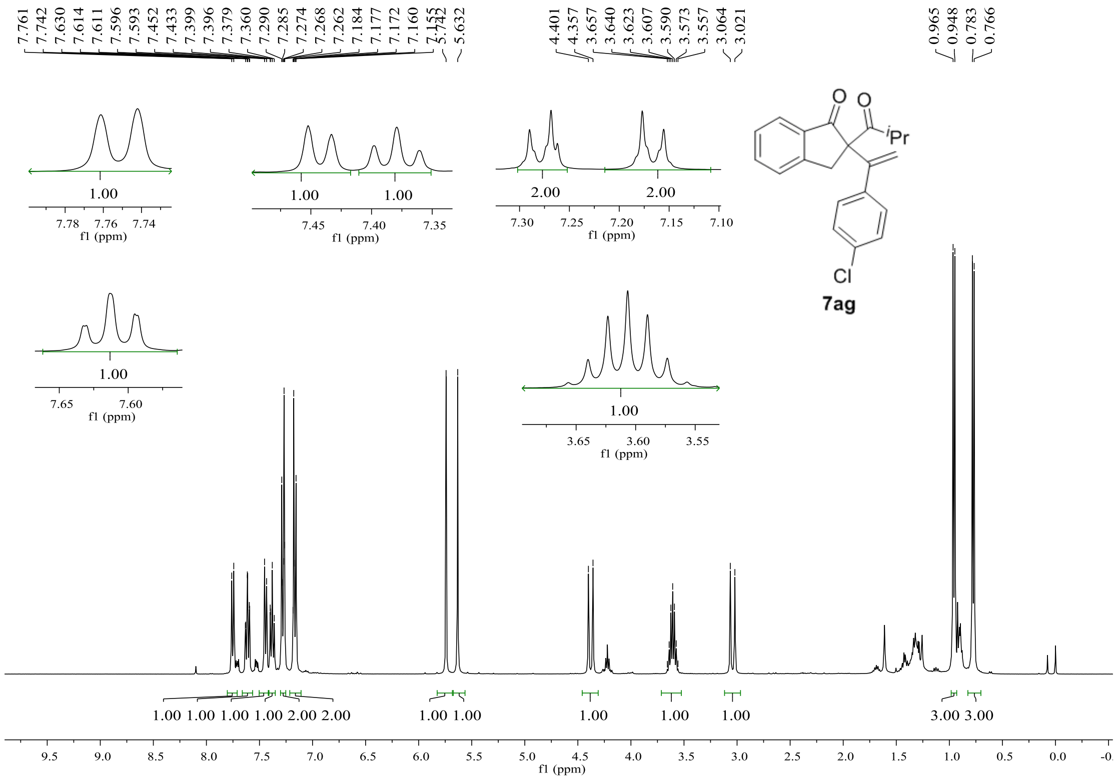

Supplementary Figure 112. ^{1}H NMR spectra for product **7ag**

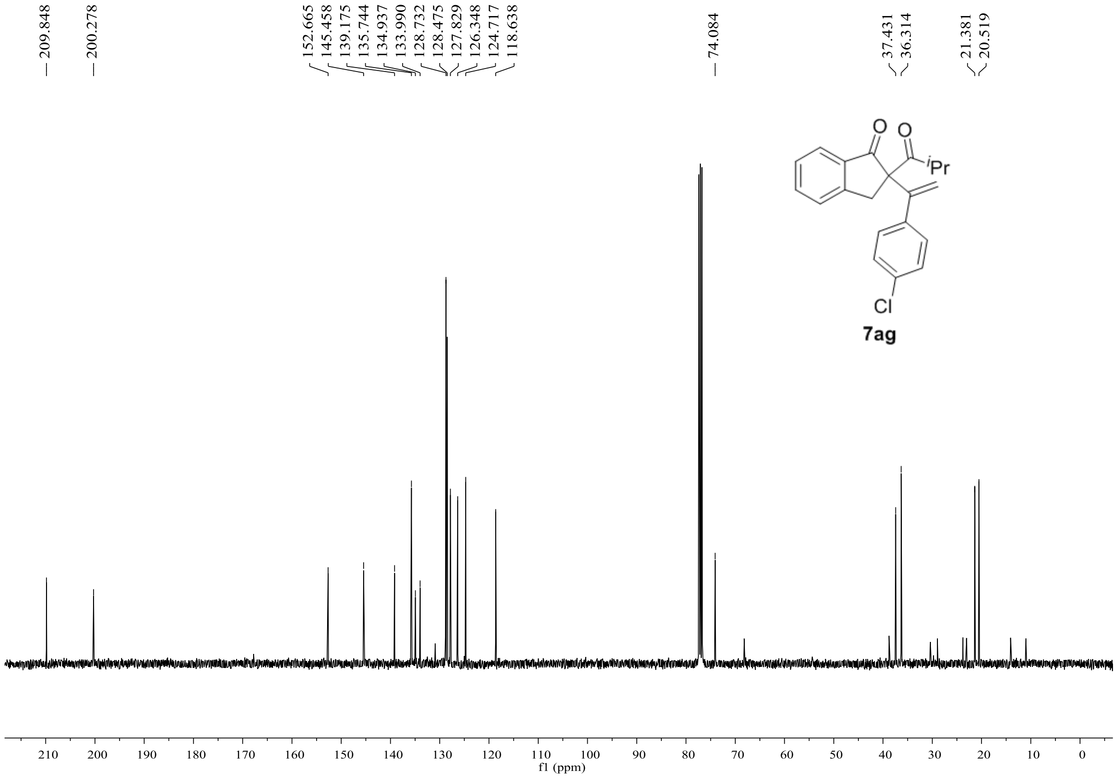

Supplementary Figure 113. ^{13}C NMR spectra for product **7ag**

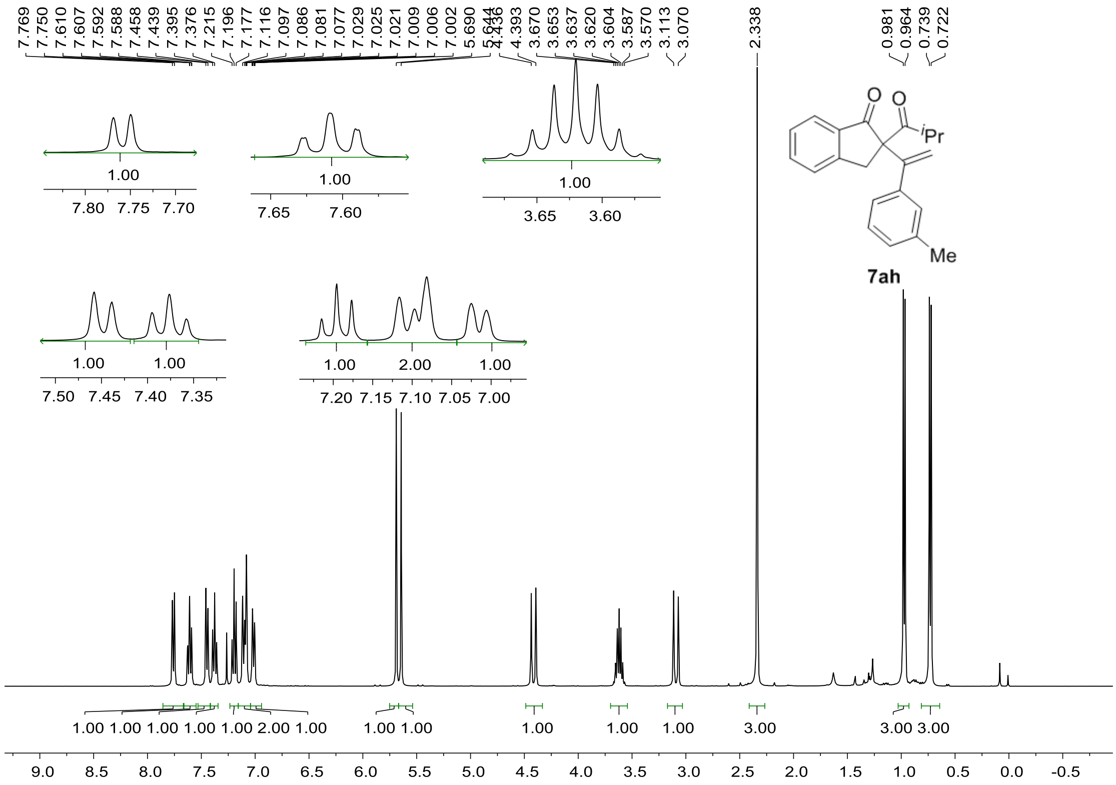

Supplementary Figure 114. ^{1}H NMR spectra for product **7ah**

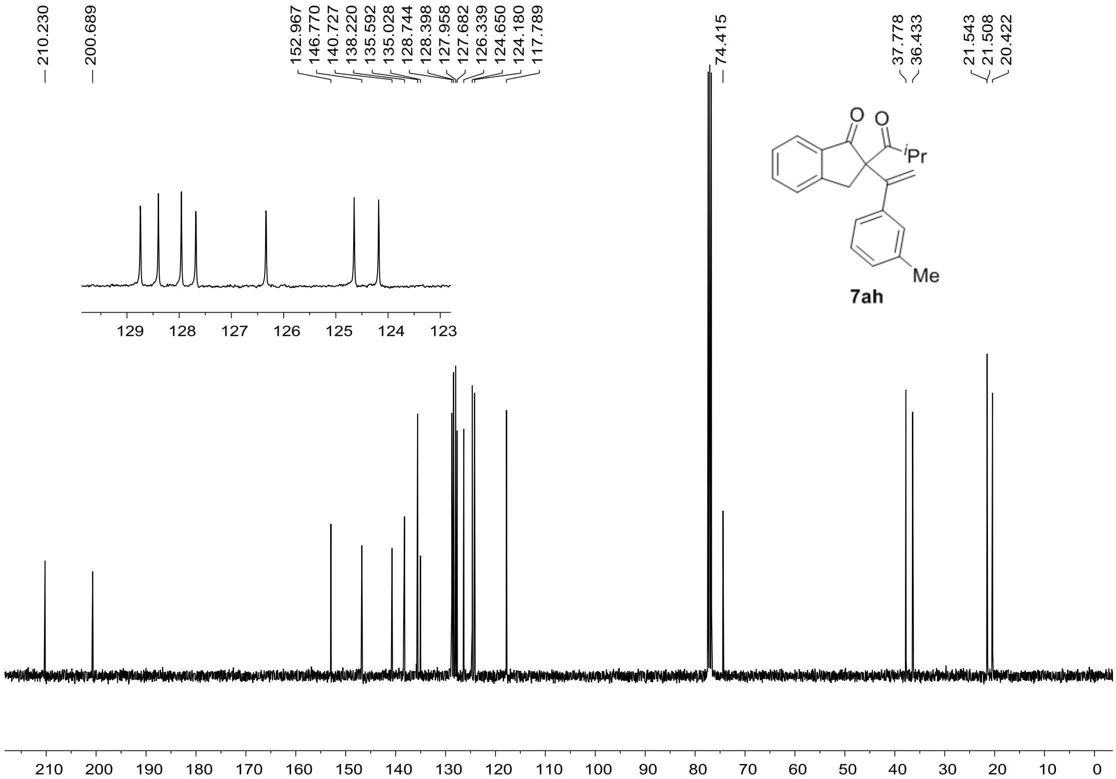

Supplementary Figure 115. ^{13}C NMR spectra for product **7ah**

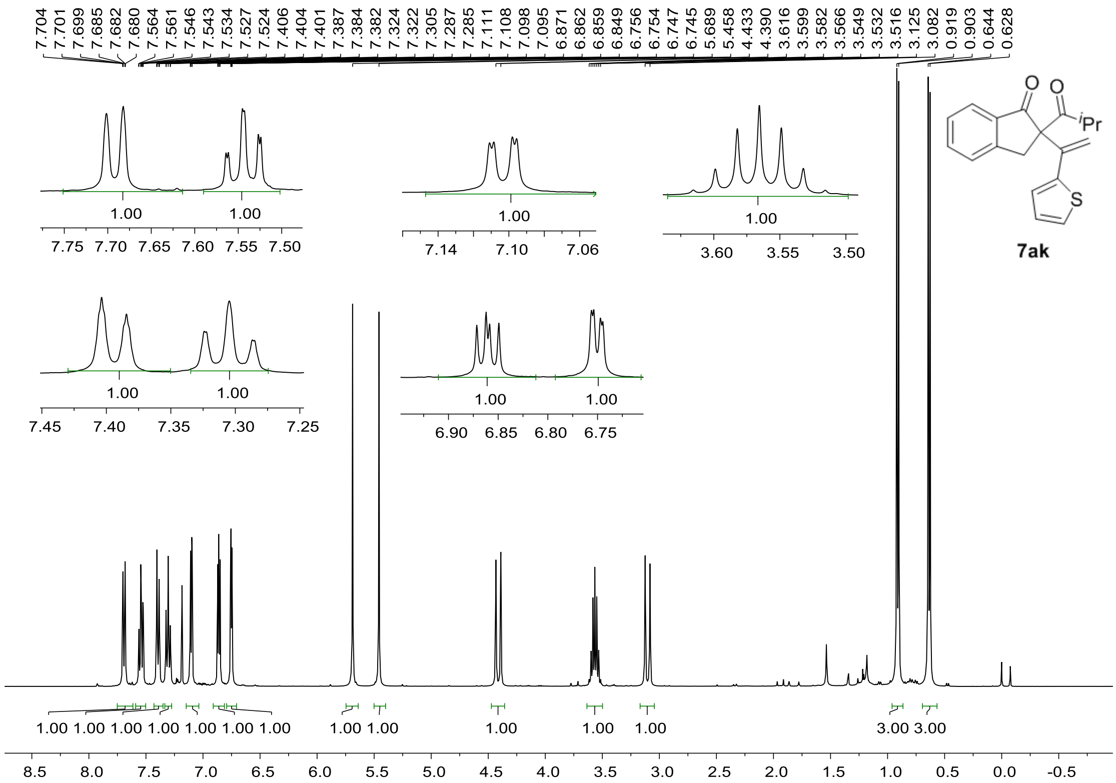

Supplementary Figure 116. ^{1}H NMR spectra for product **7ak**

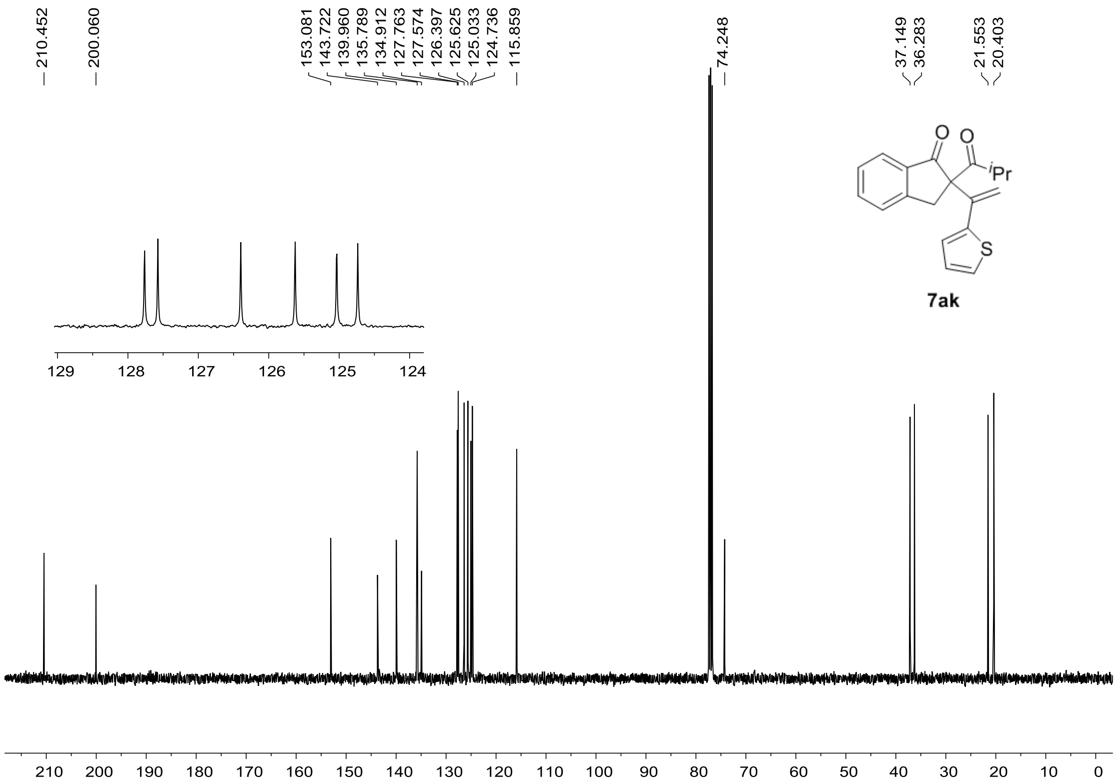

Supplementary Figure 117. ^{13}C NMR spectra for product **7ak**

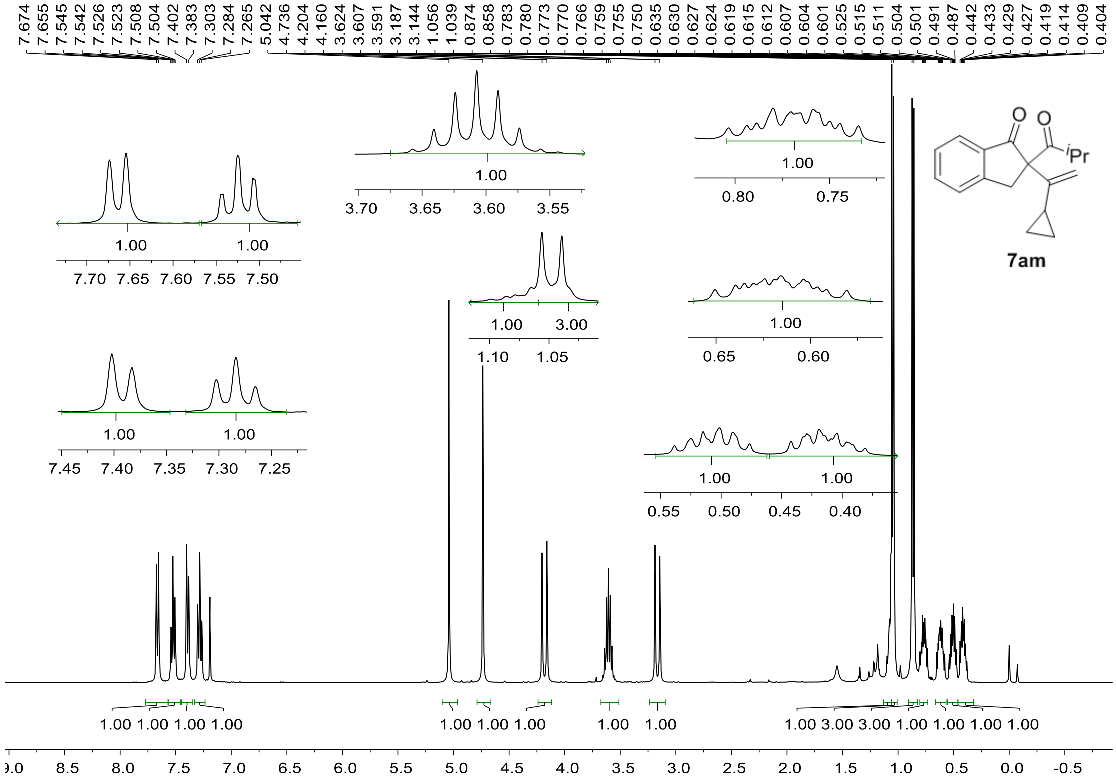

Supplementary Figure 118. ^{1}H NMR spectra for product **7am**

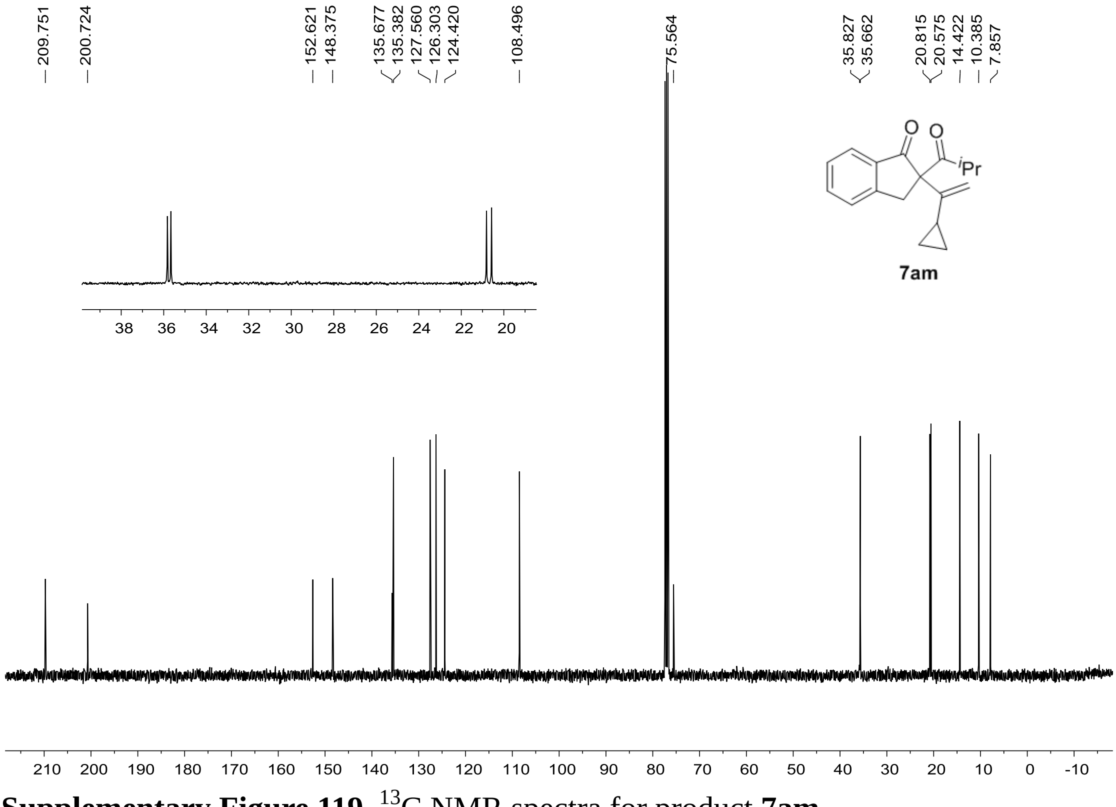

Supplementary Figure 119. ^{13}C NMR spectra for product **7am**

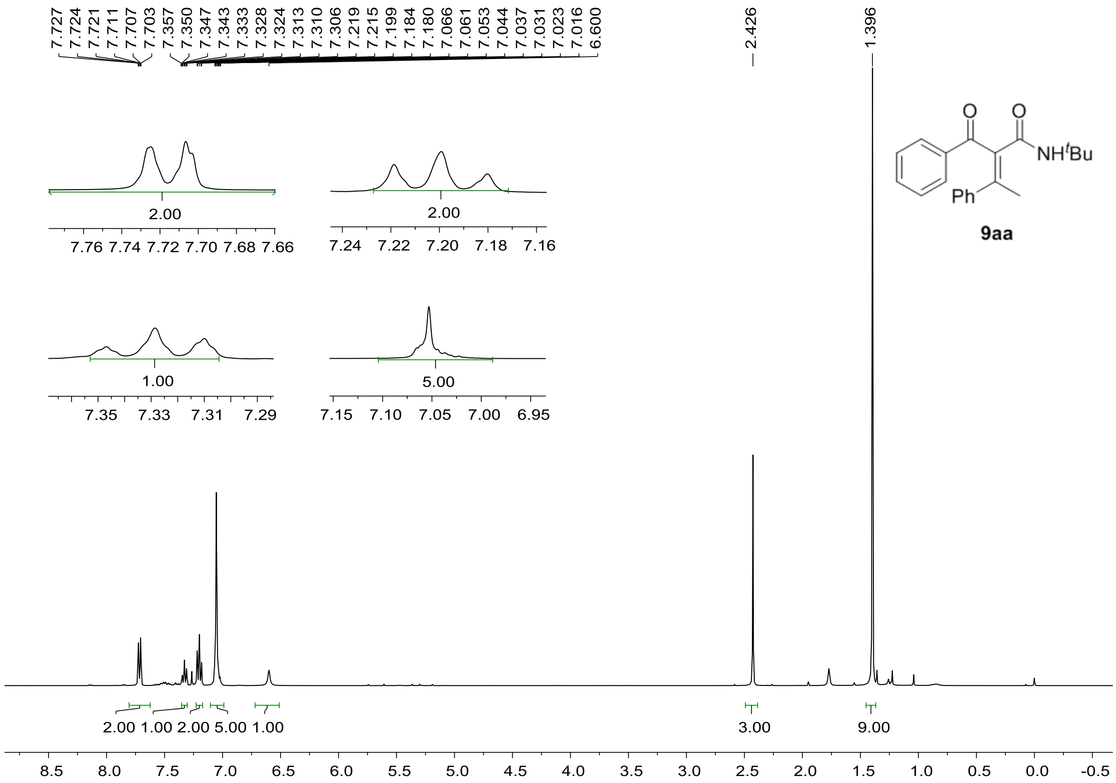

Supplementary Figure 120. ^{1}H NMR spectra for product **9aa**

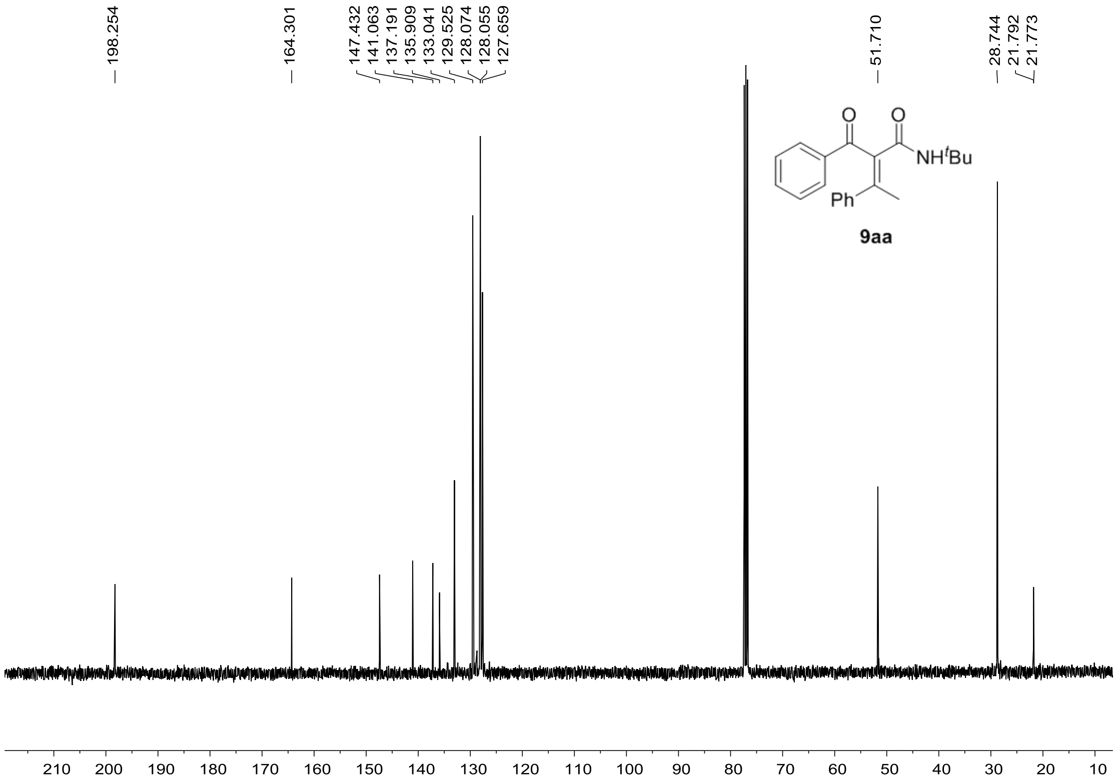

Supplementary Figure 121. ^{13}C NMR spectra for product **9aa**

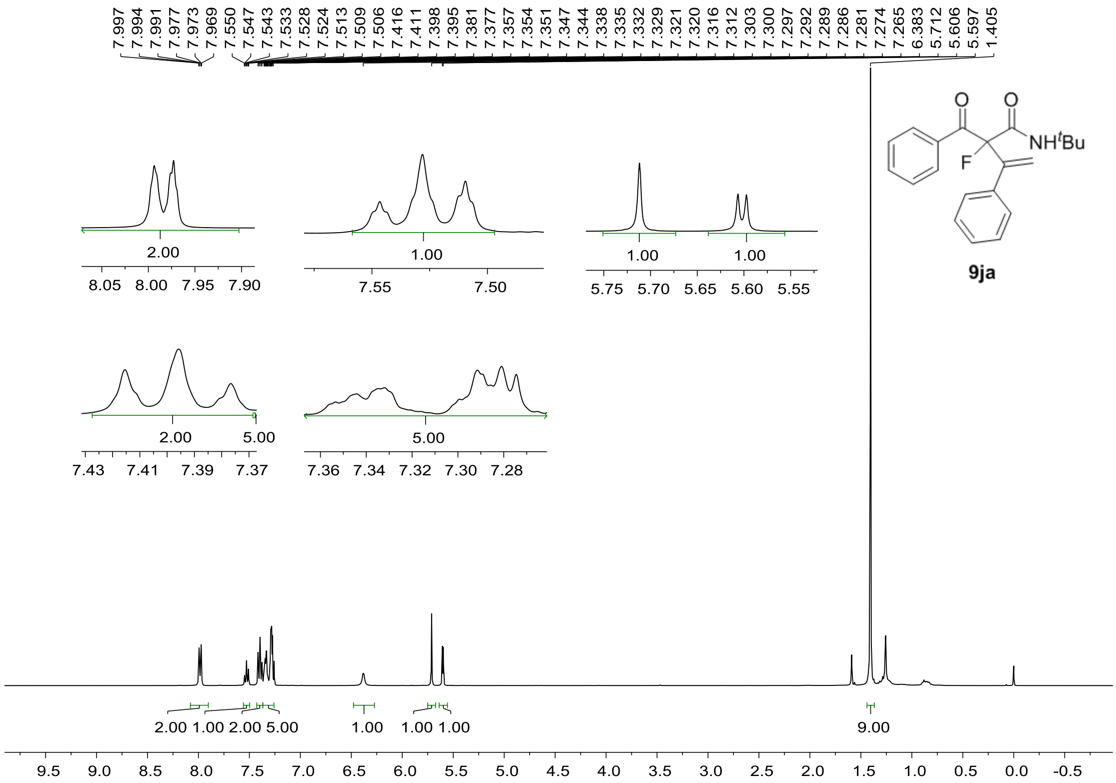

Supplementary Figure 122. ^{1}H NMR spectra for product **9ja**

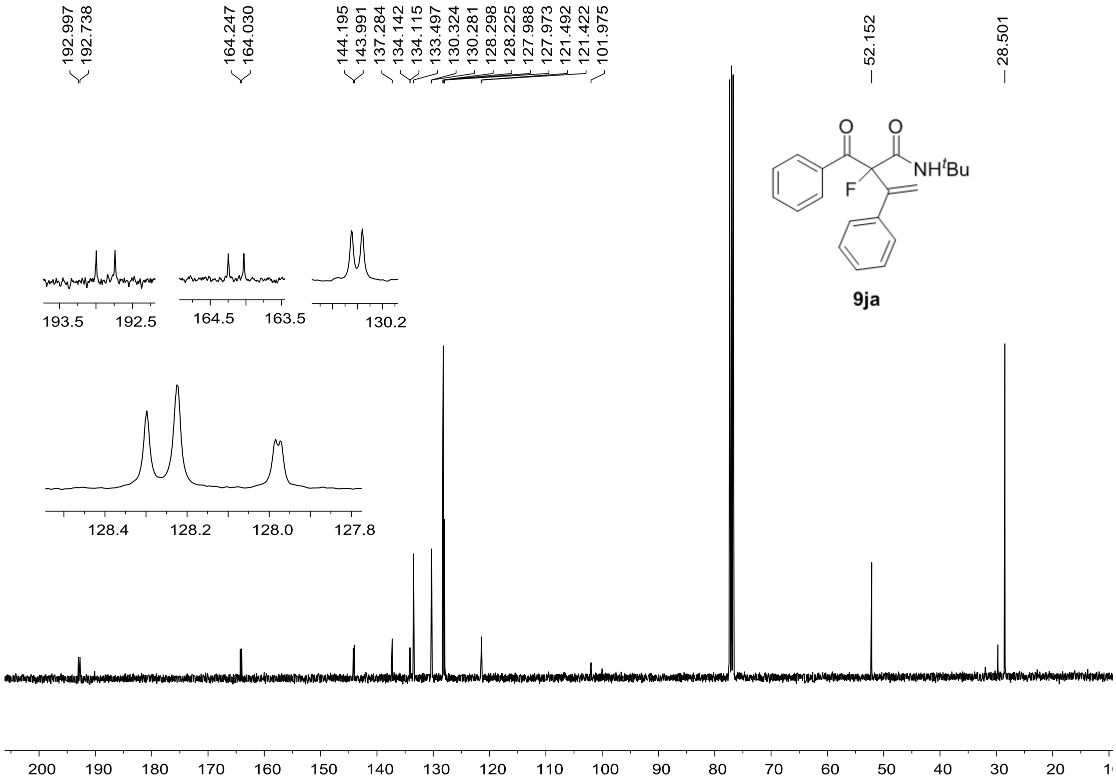

Supplementary Figure 123. ^{13}C NMR spectra for product **9ja**

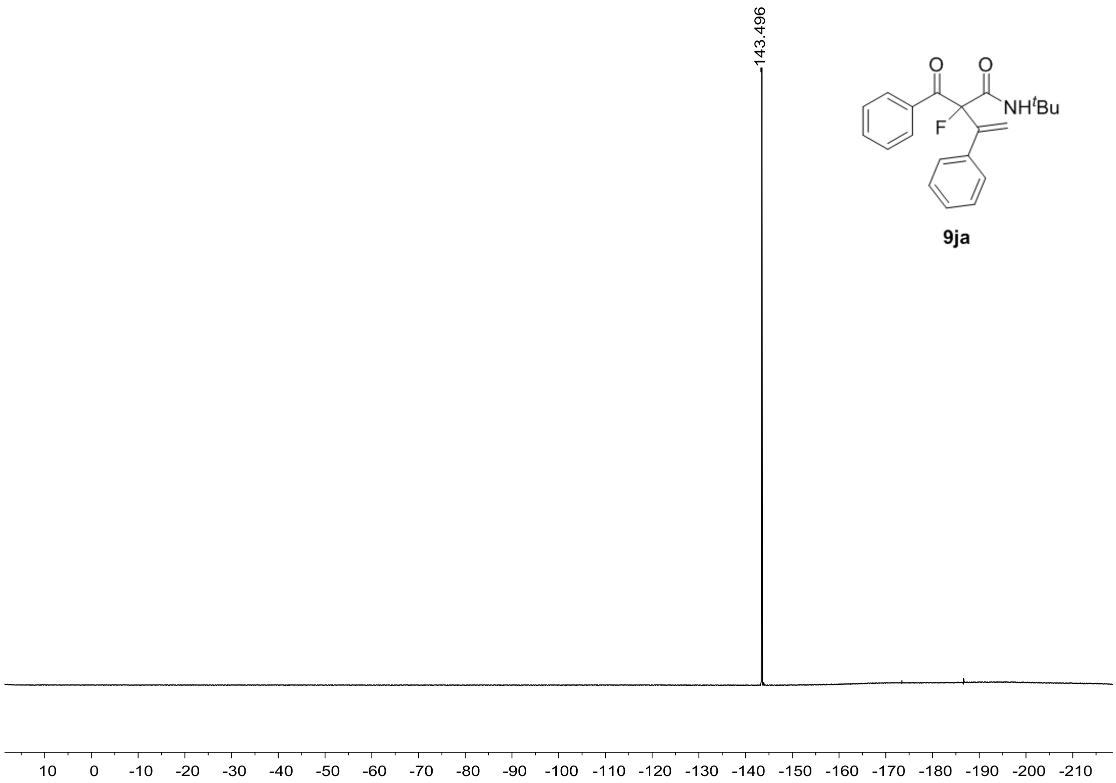

Supplementary Figure 124. ^{19}F NMR spectra for product **9ja**

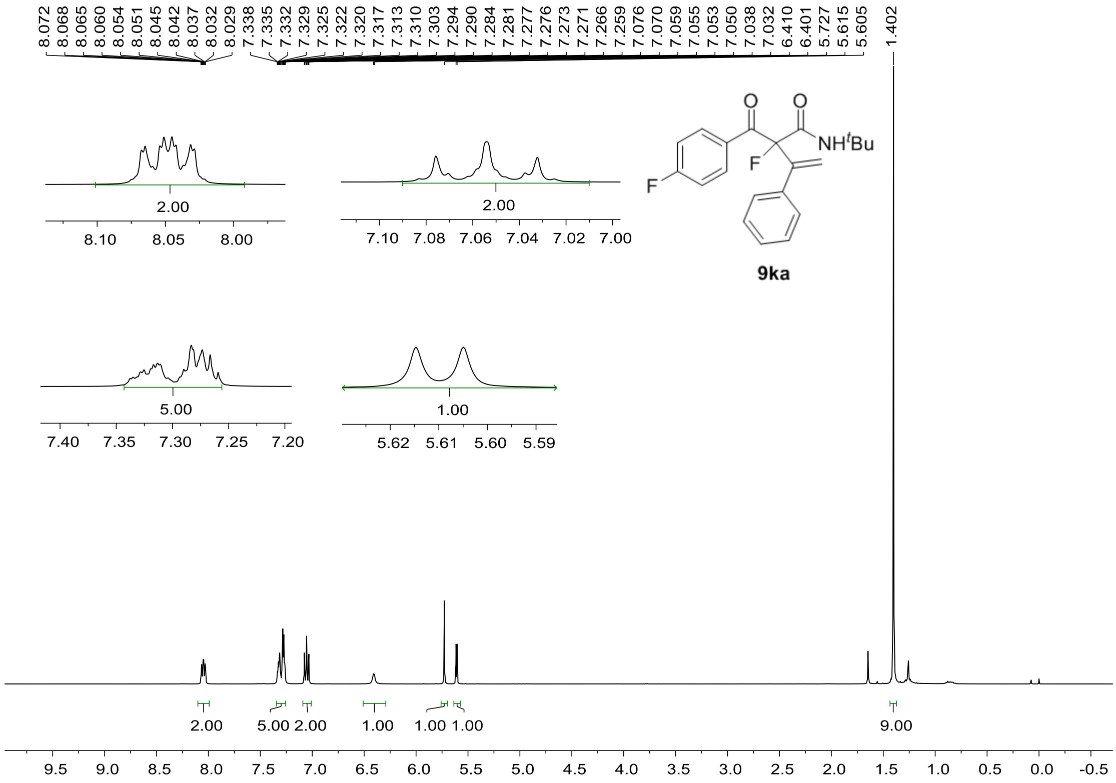

Supplementary Figure 125. ^{1}H NMR spectra for product **9ka**

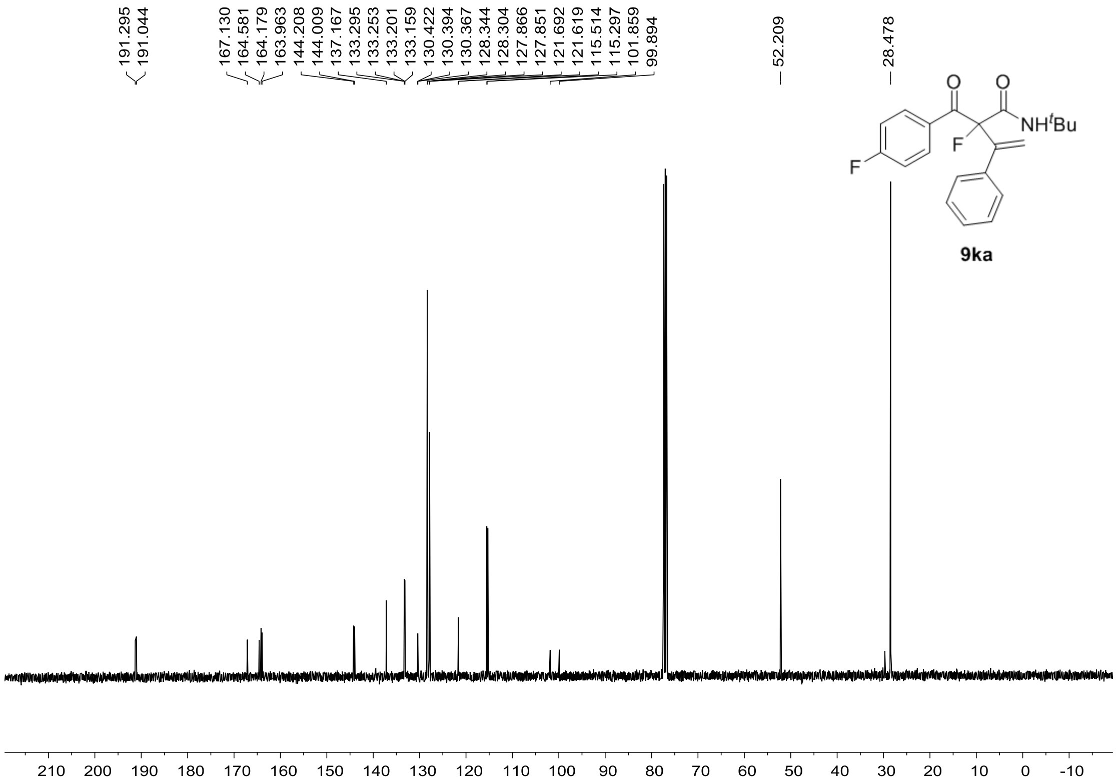

Supplementary Figure 126. ^{13}C NMR spectra for product **9ka**

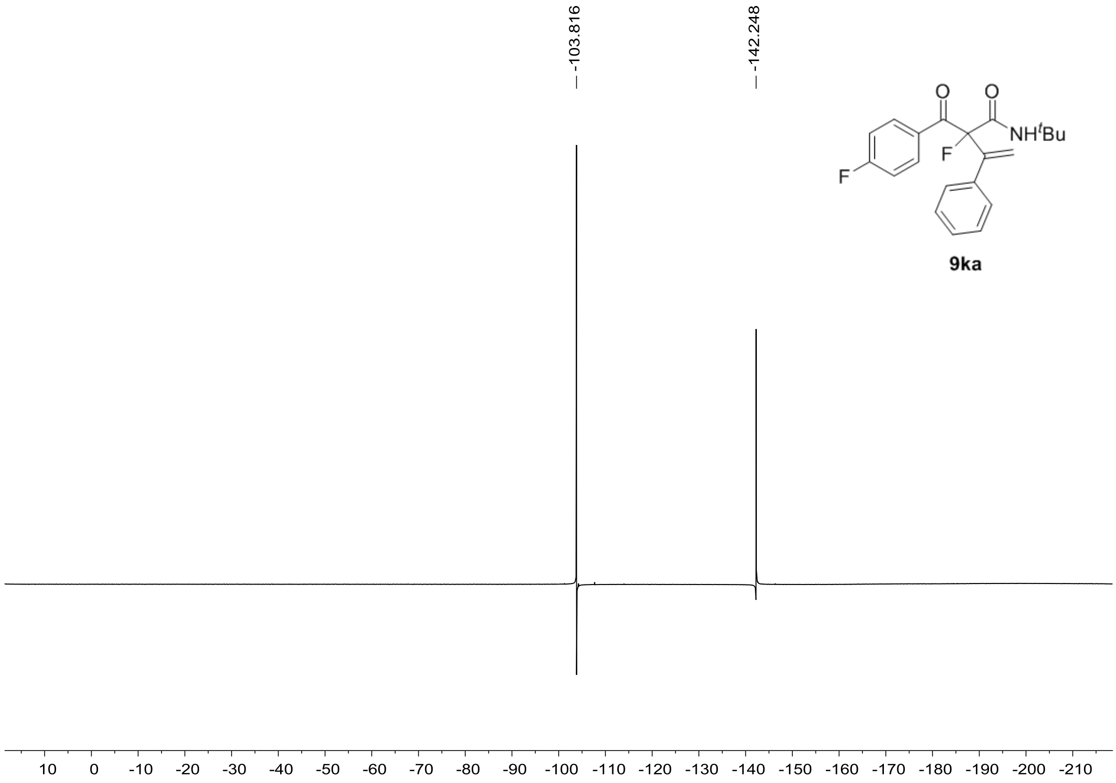

Supplementary Figure 127. ^{19}F NMR spectra for product **9ka**

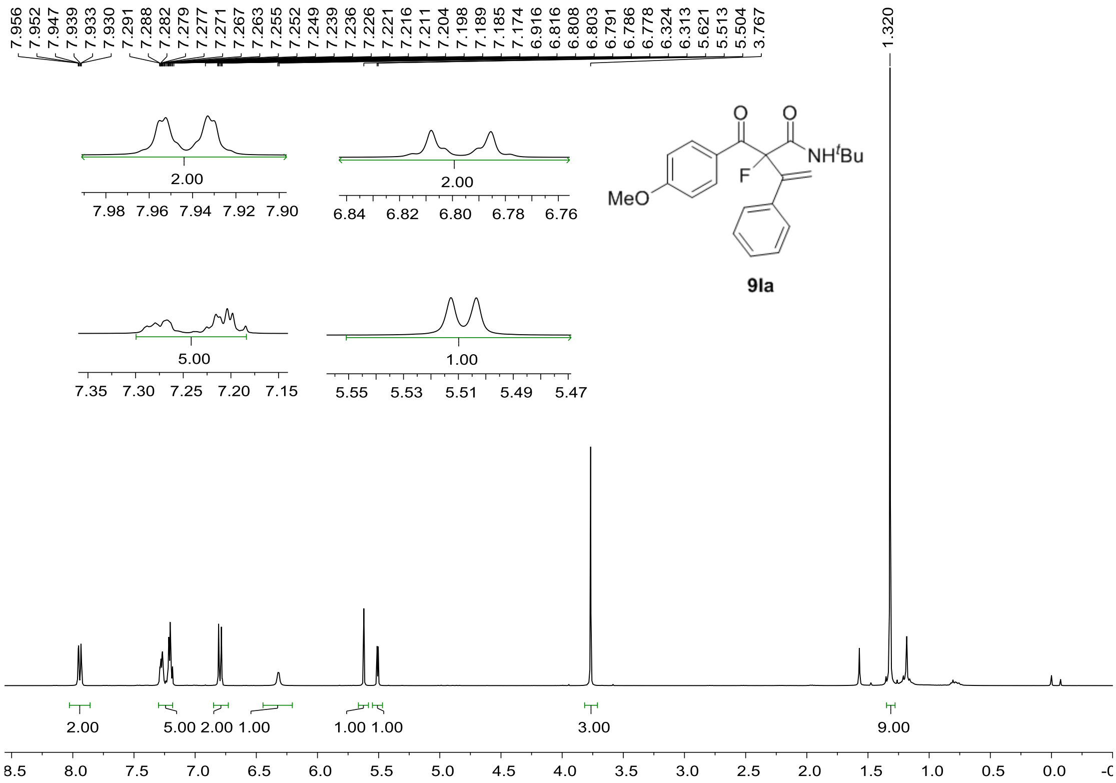

Supplementary Figure 128. ^{1}H NMR spectra for product **9la**

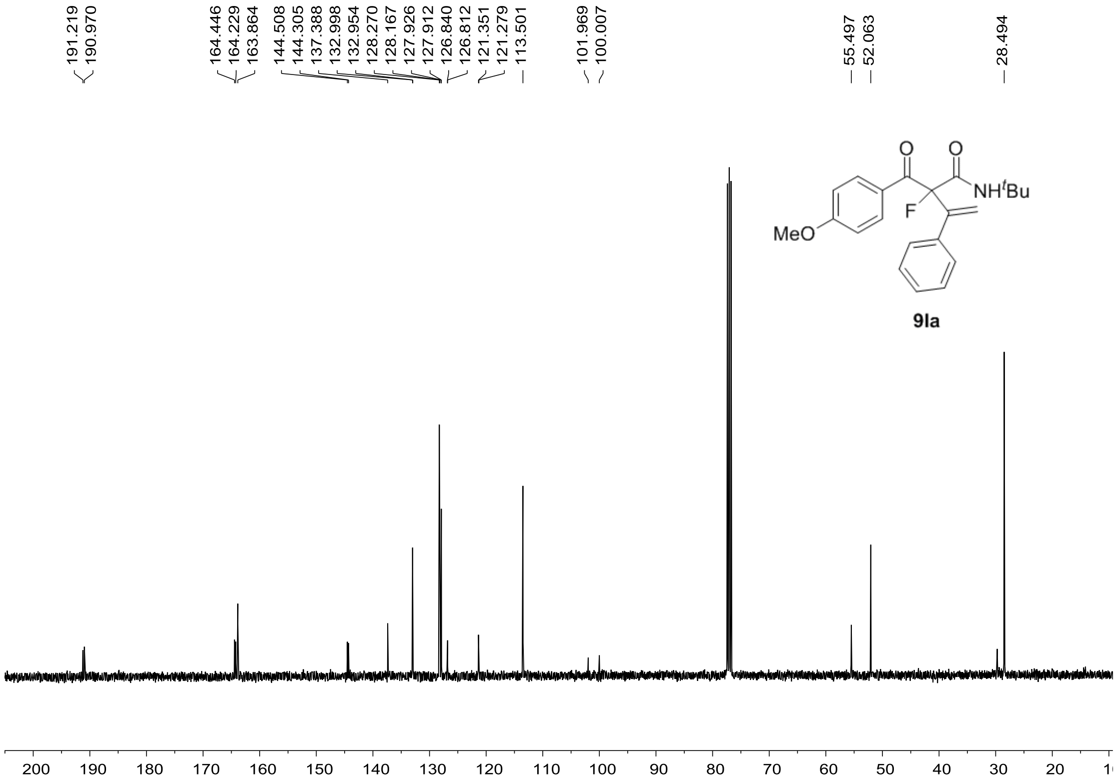

Supplementary Figure 129. ^{13}C NMR spectra for product **9la**

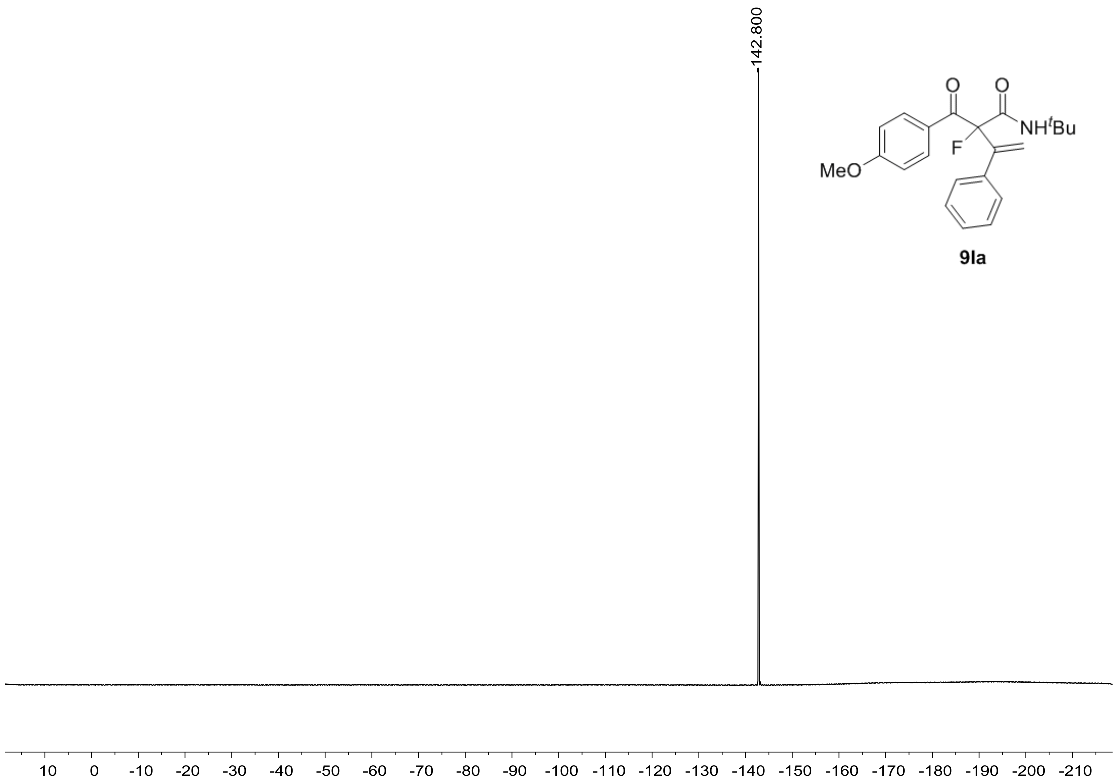

Supplementary Figure 130. ^{19}F NMR spectra for product **9la**

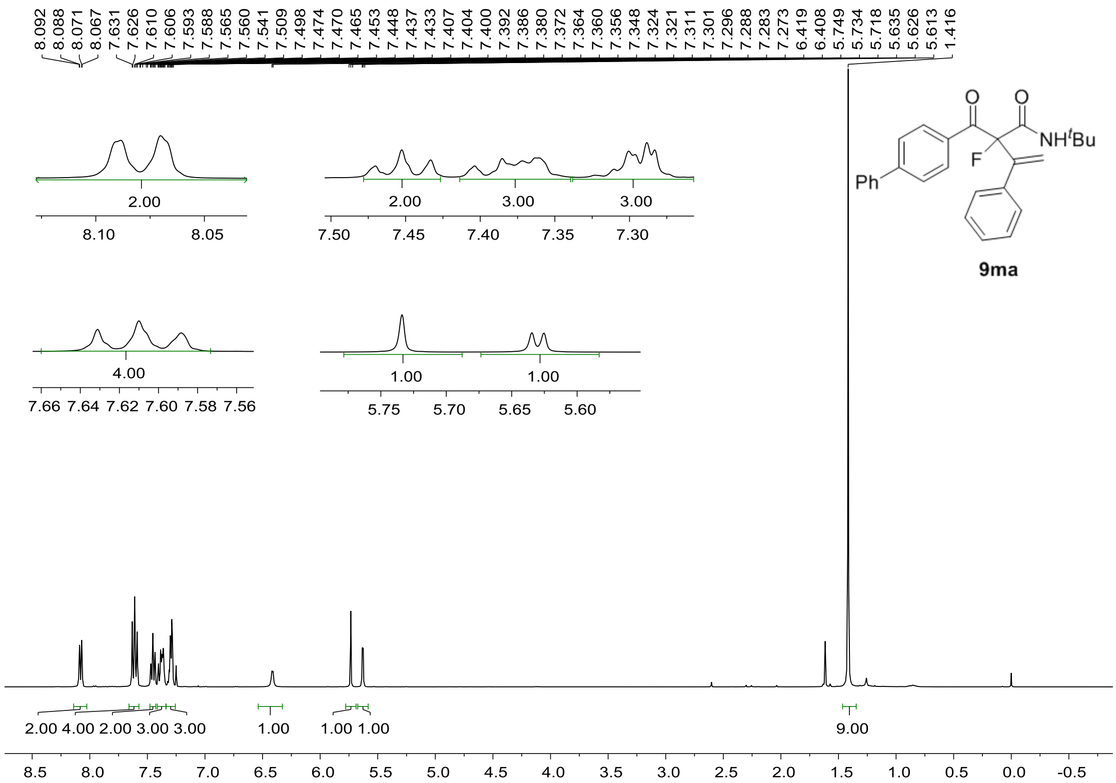

Supplementary Figure 131. ^{1}H NMR spectra for product **9ma**

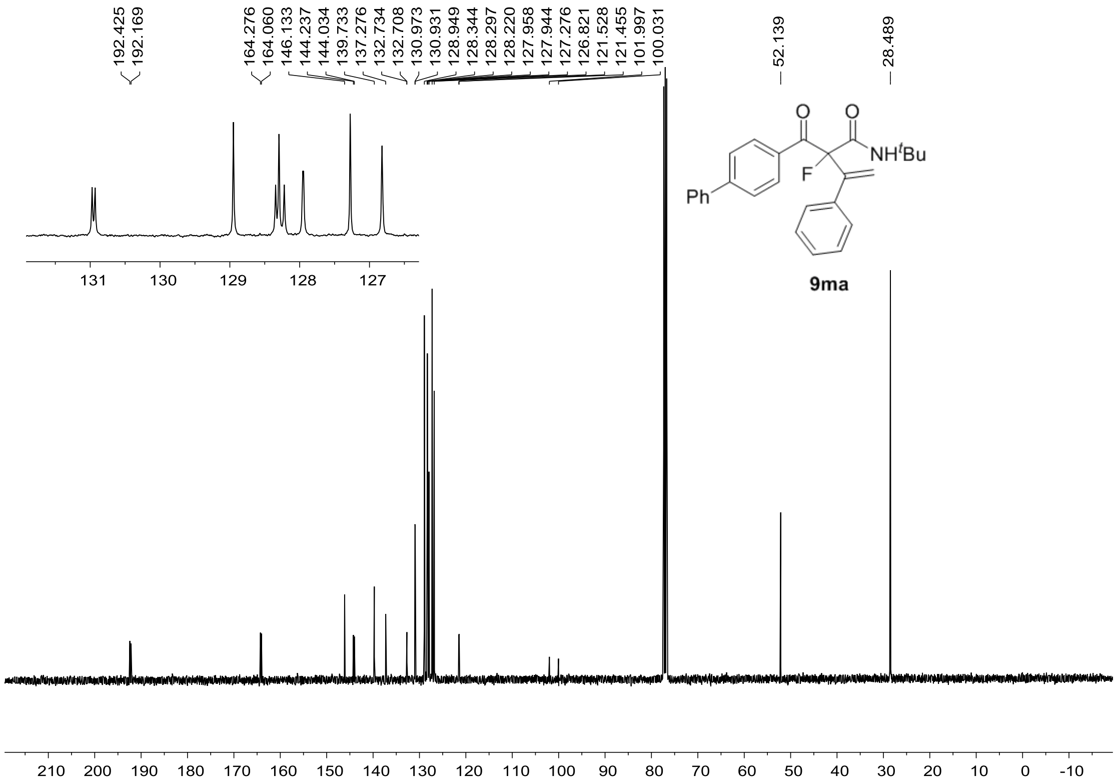

Supplementary Figure 132. ^{13}C NMR spectra for product **9ma**

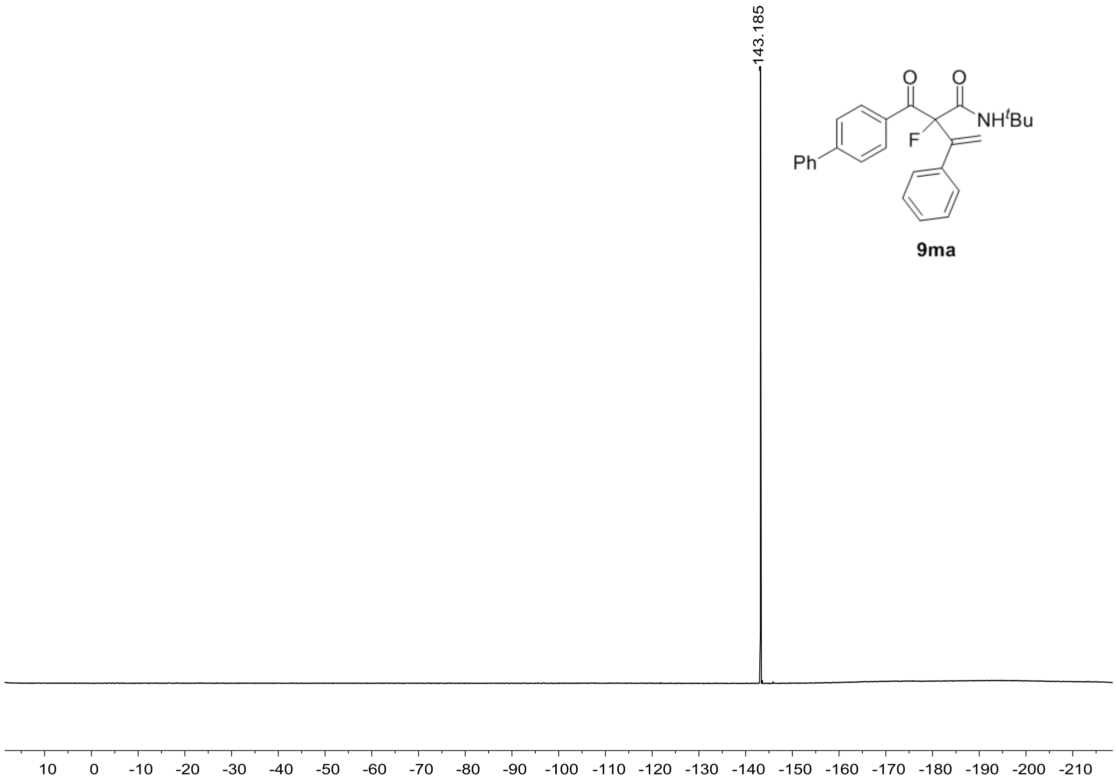

Supplementary Figure 133. ^{19}F NMR spectra for product **9ma**

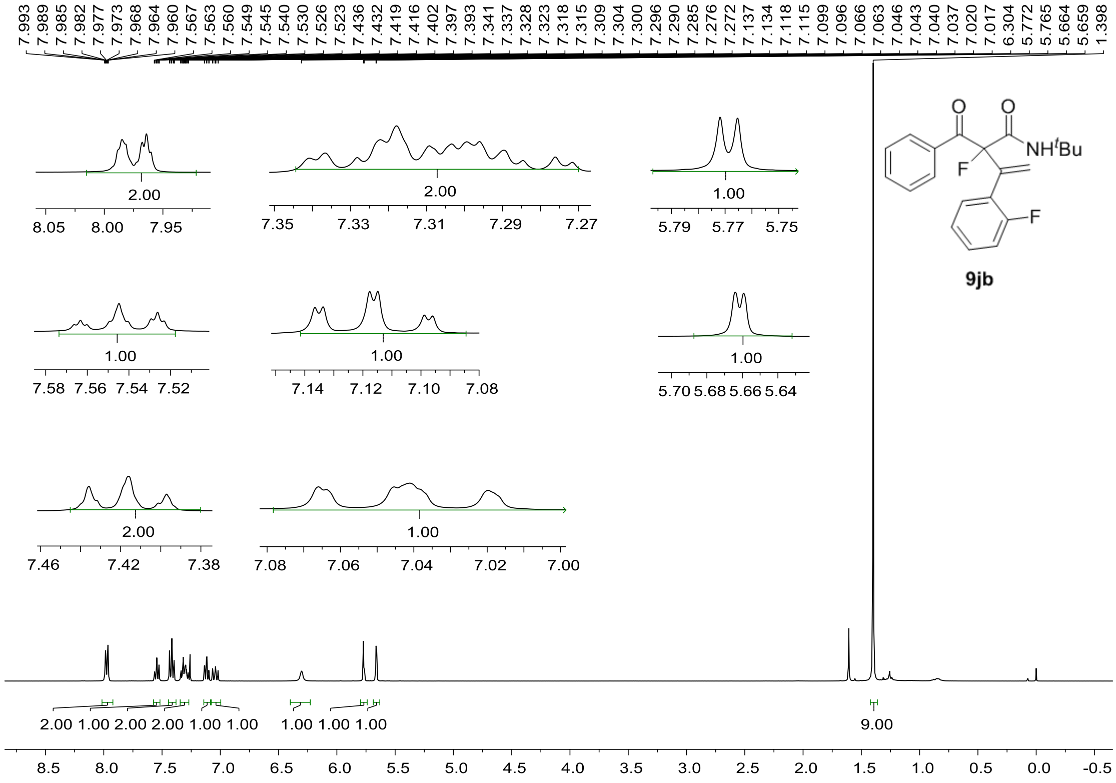

Supplementary Figure 134. ^{1}H NMR spectra for product **9jb**

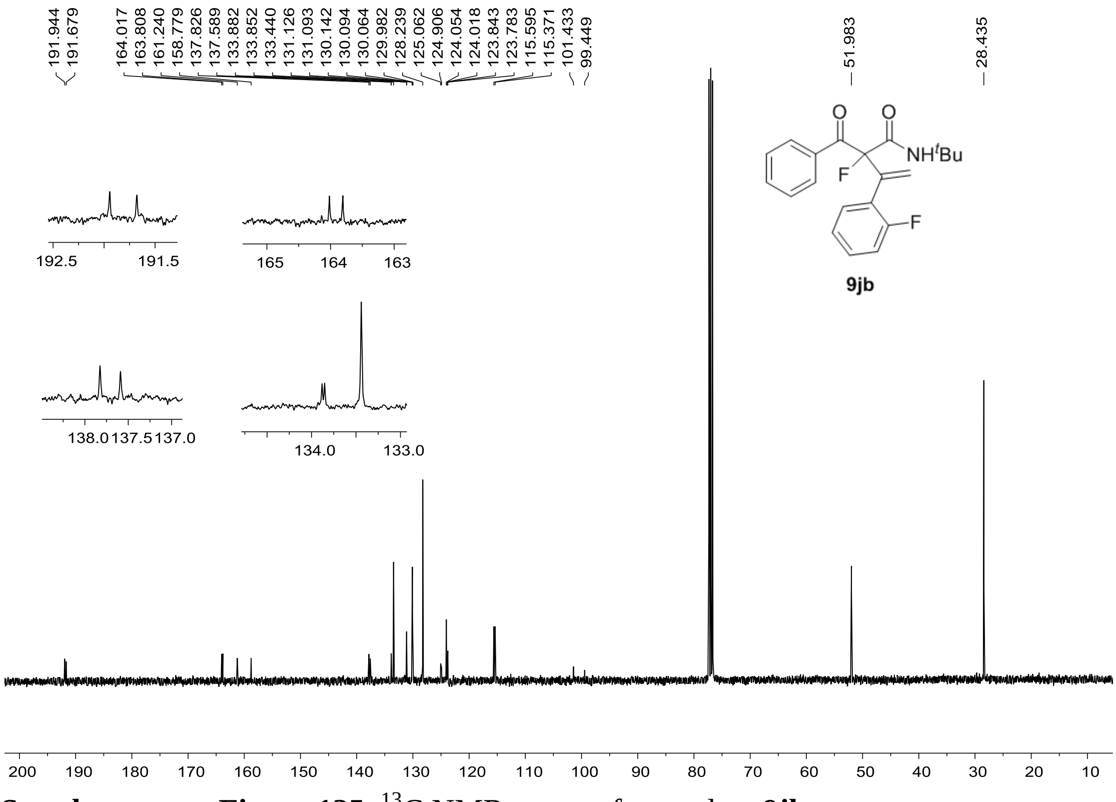

Supplementary Figure 135. ^{13}C NMR spectra for product **9jb**

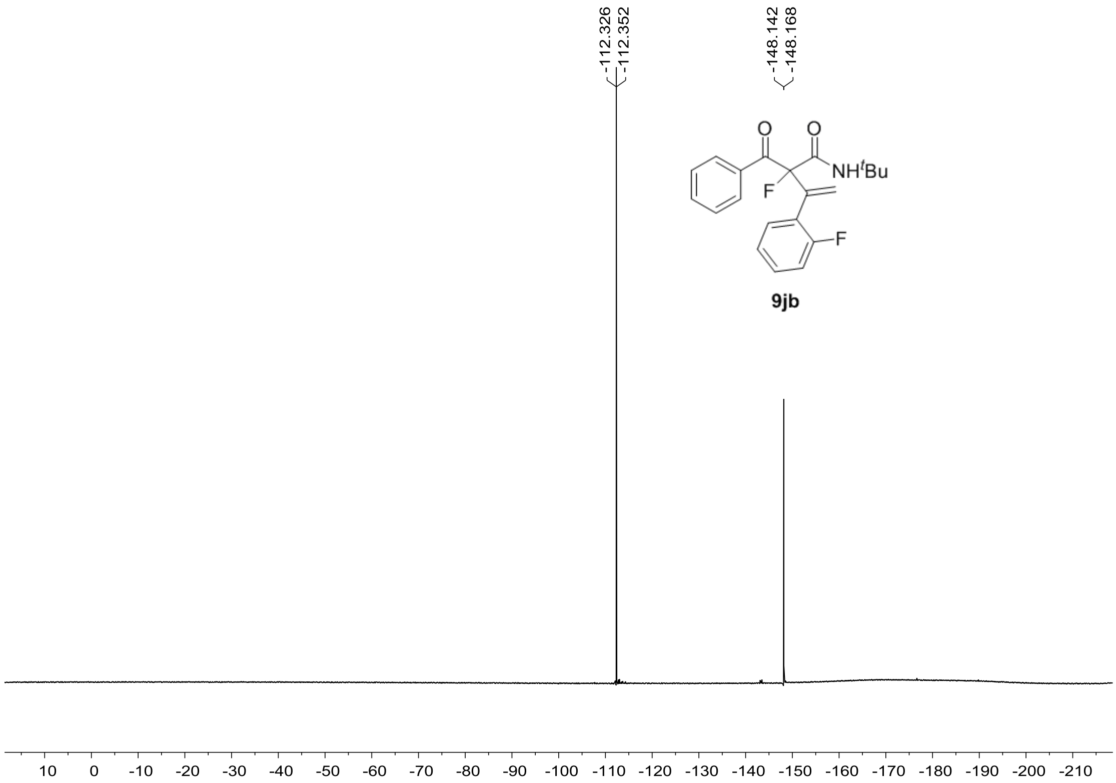

Supplementary Figure 136. ^{19}F NMR spectra for product **9jb**

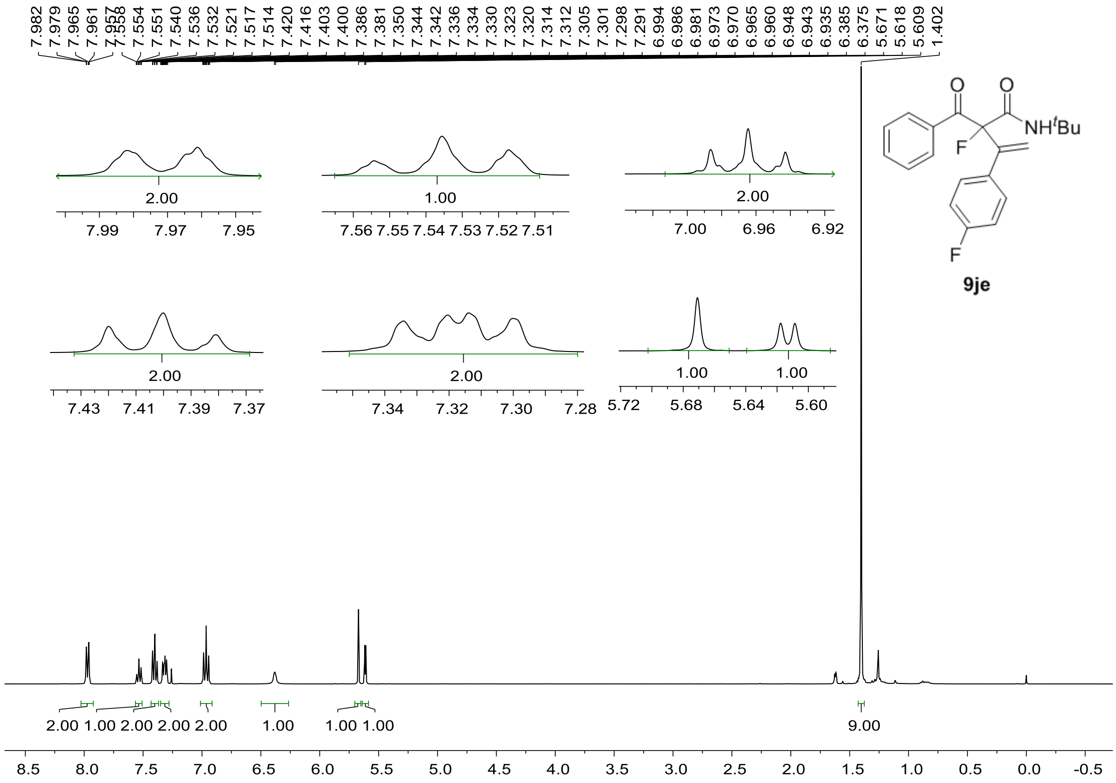

Supplementary Figure 137. ^{1}H NMR spectra for product **9je**

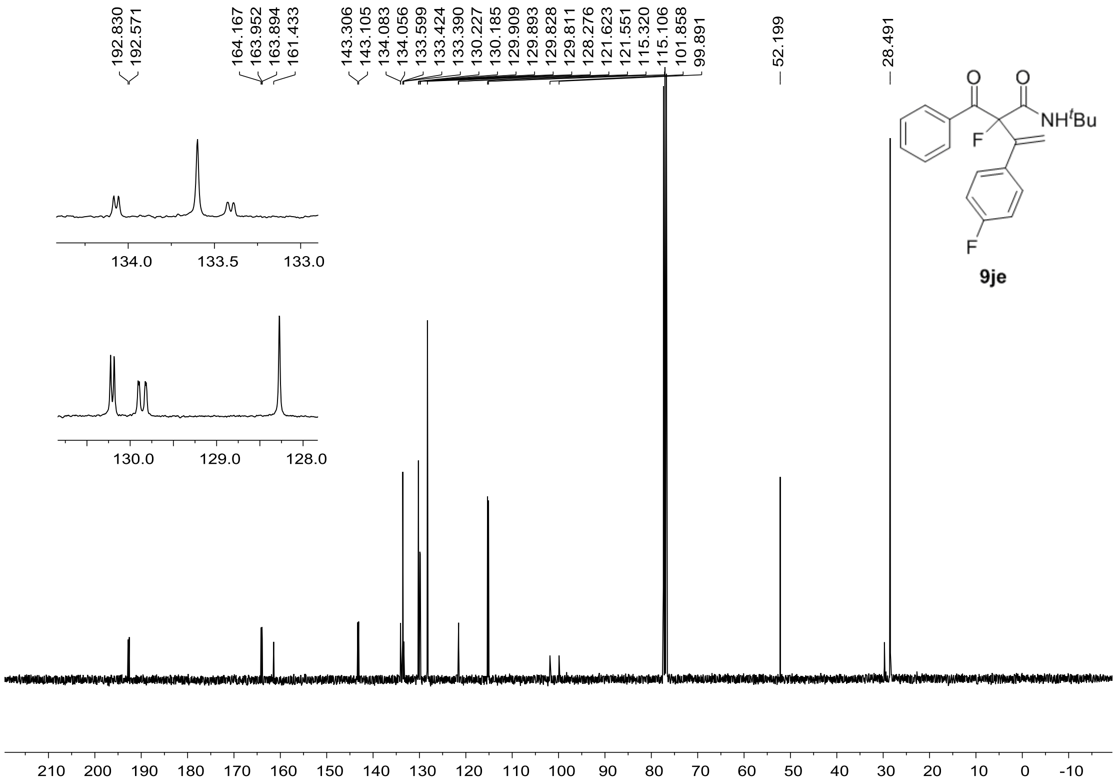

Supplementary Figure 138. ^{13}C NMR spectra for product **9je**

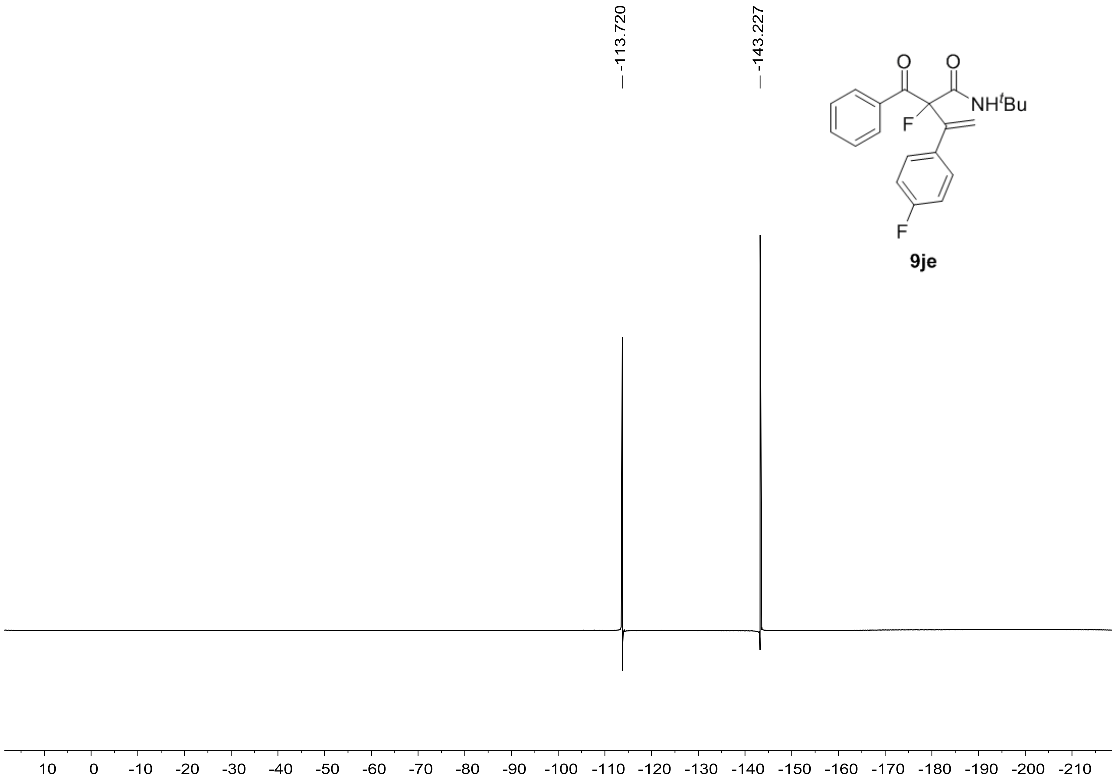

Supplementary Figure 139. ^{19}F NMR spectra for product **9je**

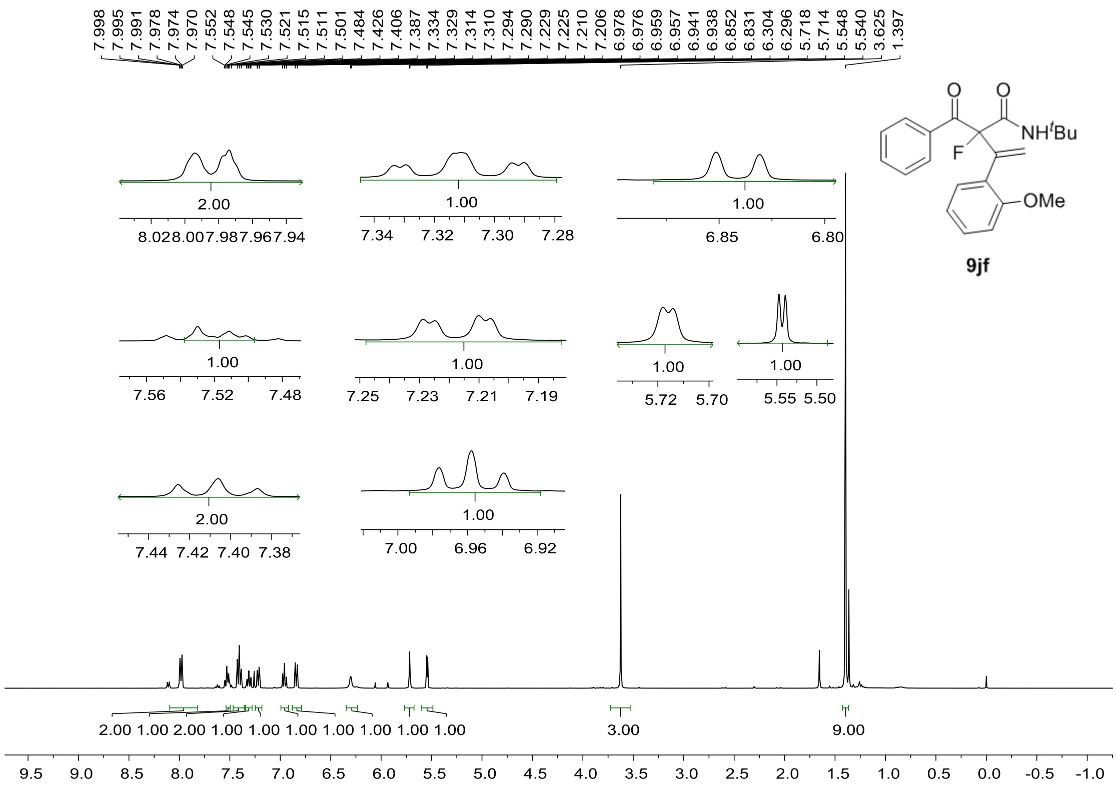

Supplementary Figure 140. ^{1}H NMR spectra for product **9jf**

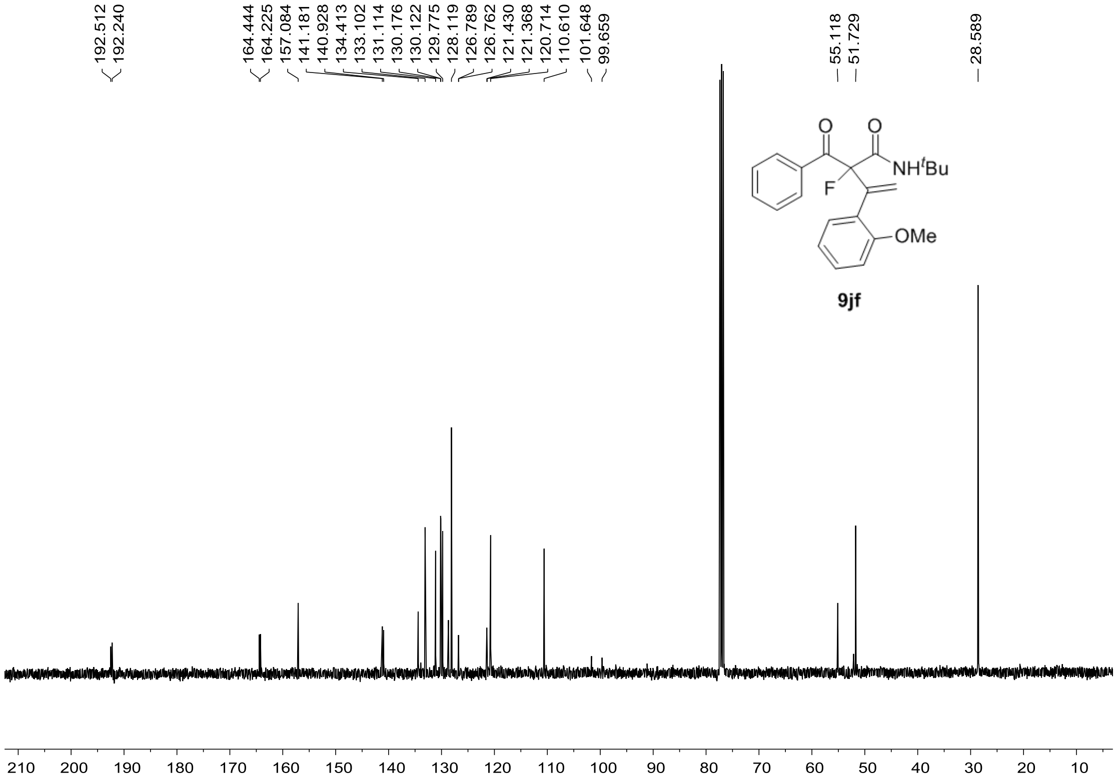

Supplementary Figure 141. ^{13}C NMR spectra for product **9jf**

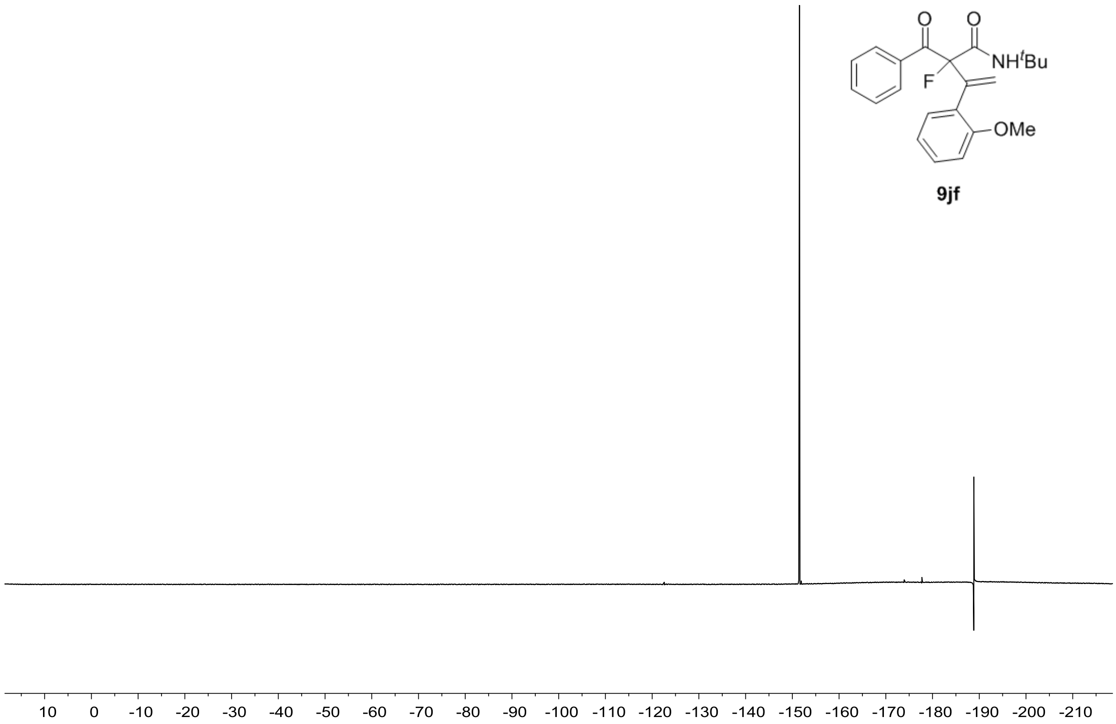

Supplementary Figure 142. ^{19}F NMR spectra for product **9jf**

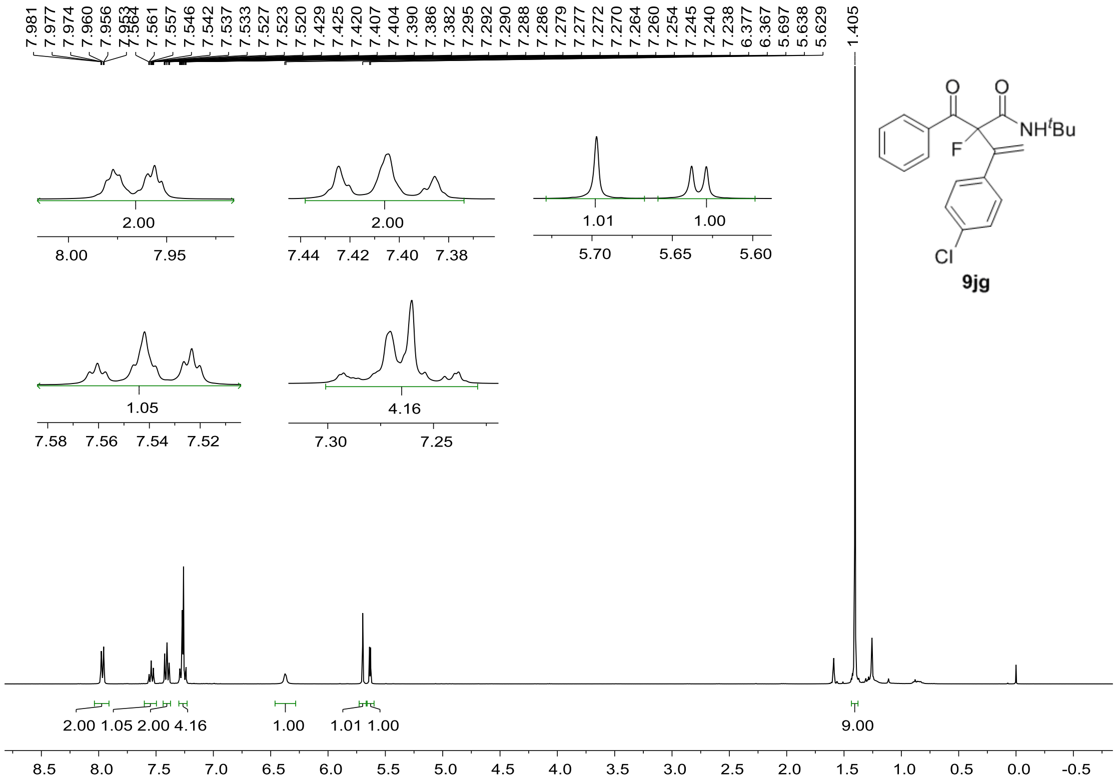

Supplementary Figure 143. ^{1}H NMR spectra for product **9jg**

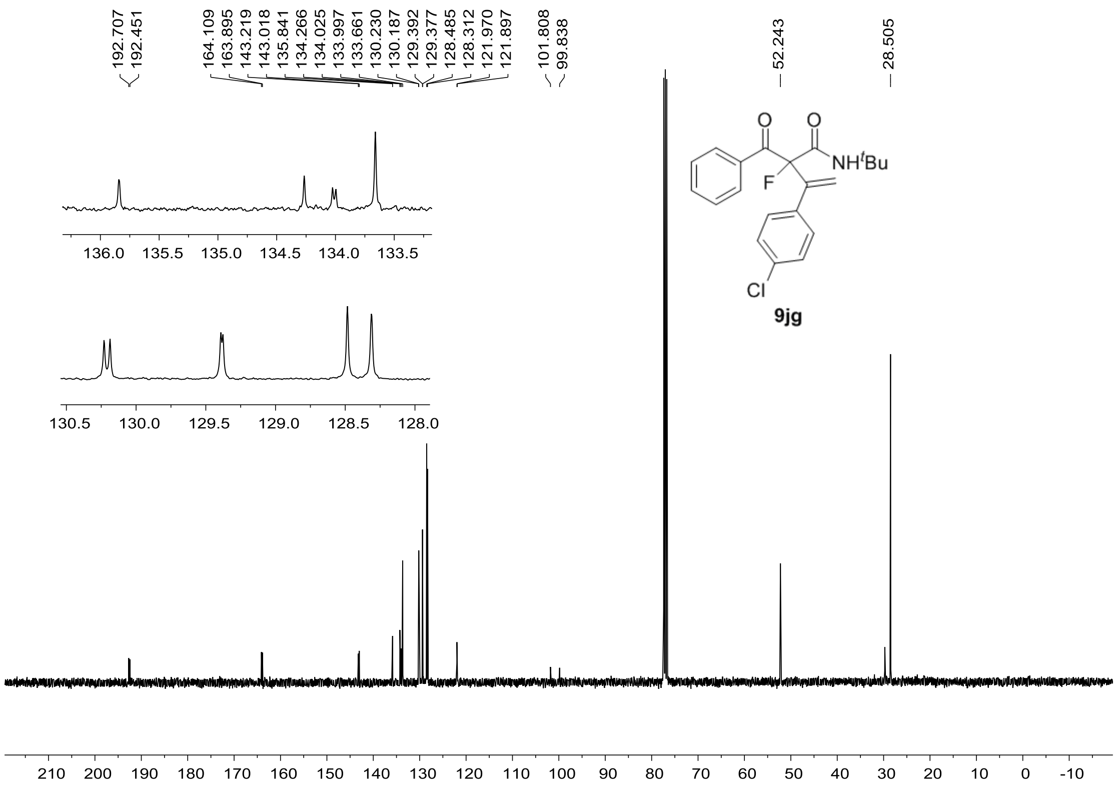

Supplementary Figure 144. ^{13}C NMR spectra for product **9jg**

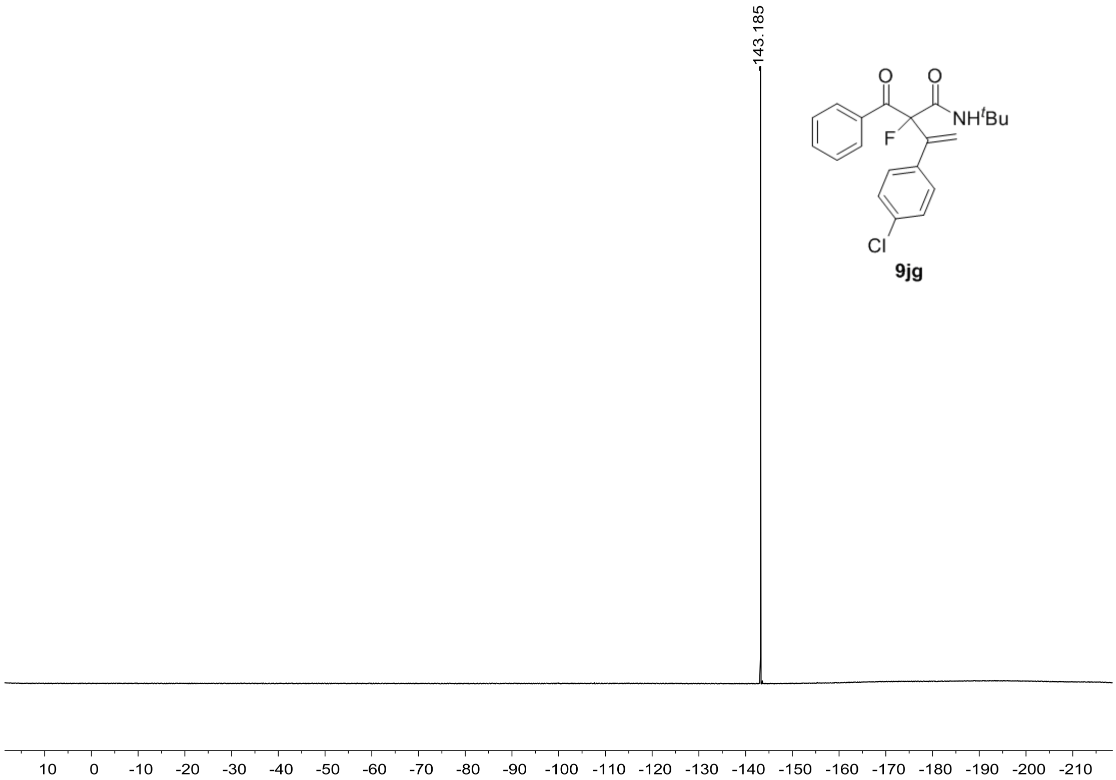

Supplementary Figure 145. ^{19}F NMR spectra for product **9jg**

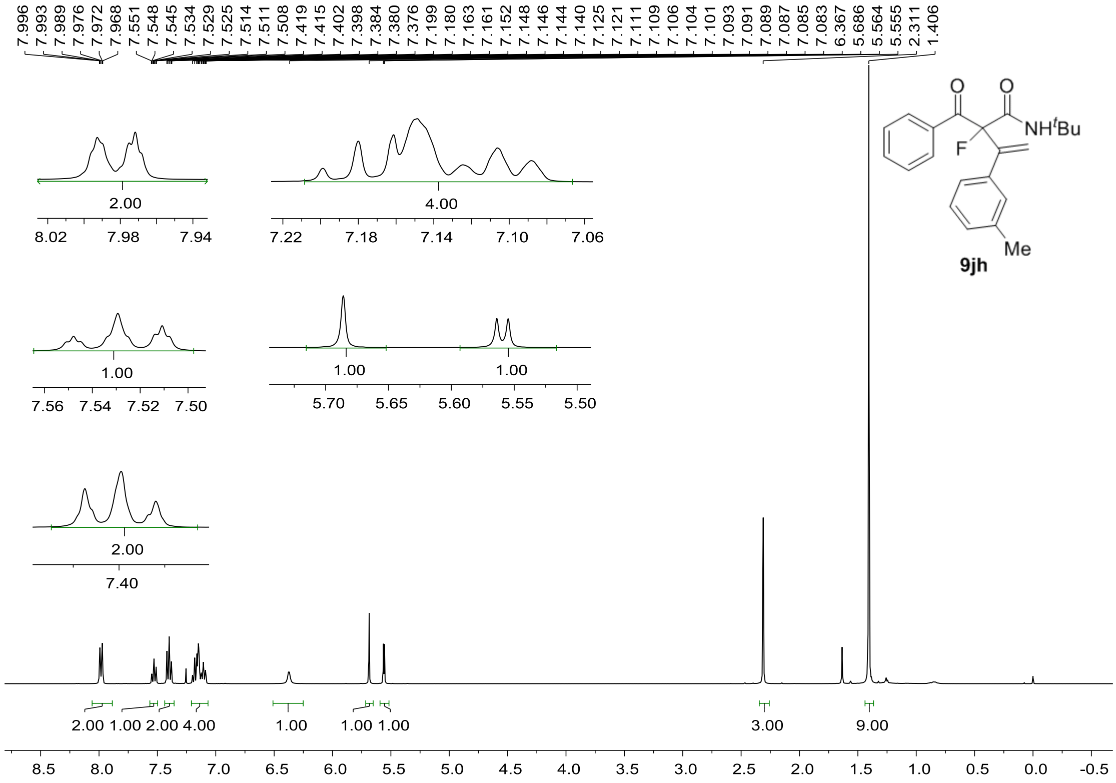

Supplementary Figure 146. ^{1}H NMR spectra for product **9jh**

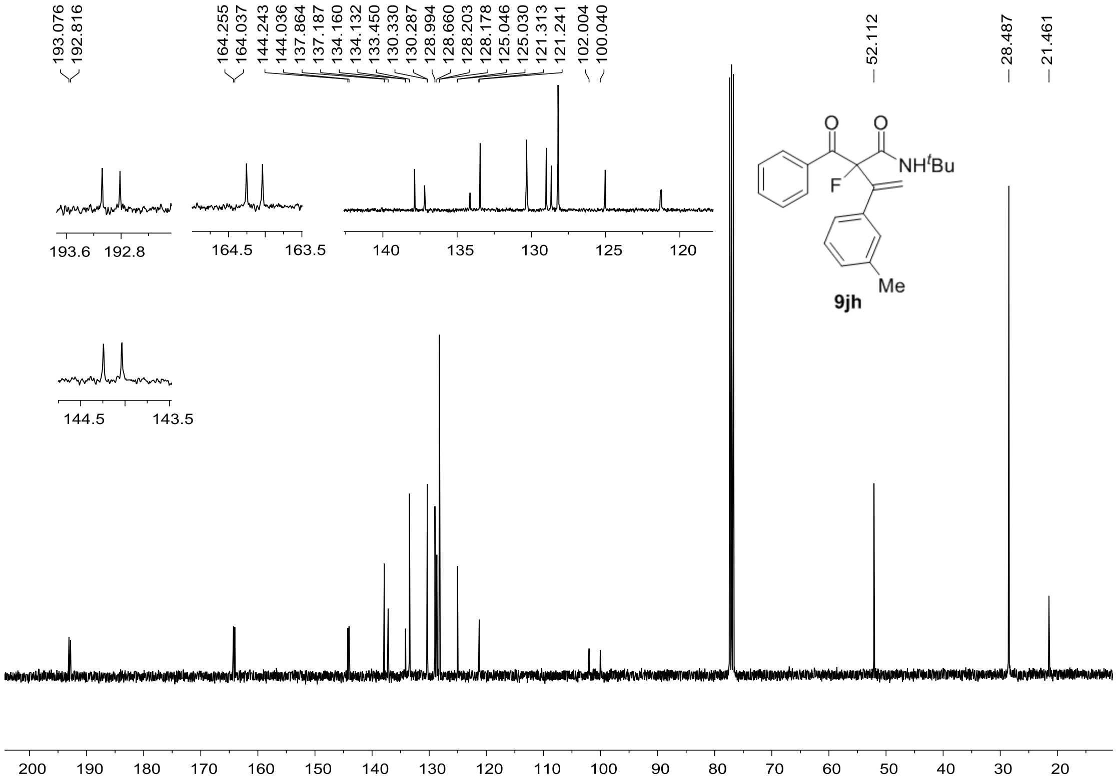

Supplementary Figure 147. ^{13}C NMR spectra for product **9jh**

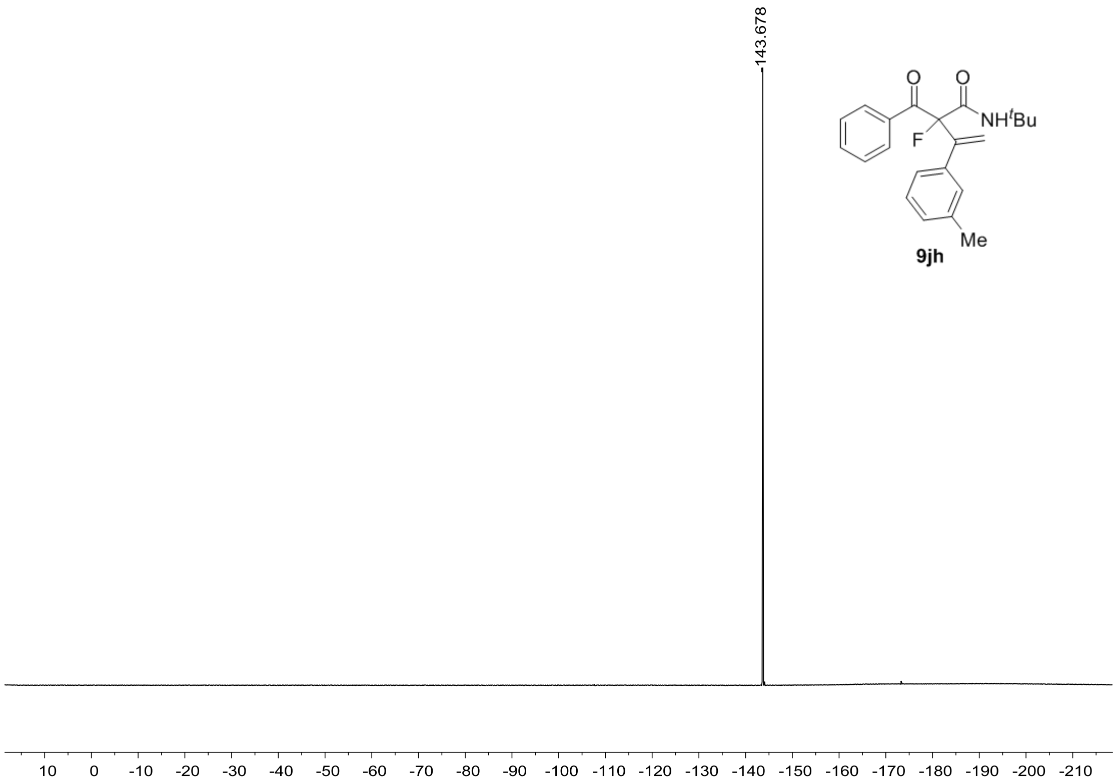

Supplementary Figure 148. ^{19}F NMR spectra for product **9jh**

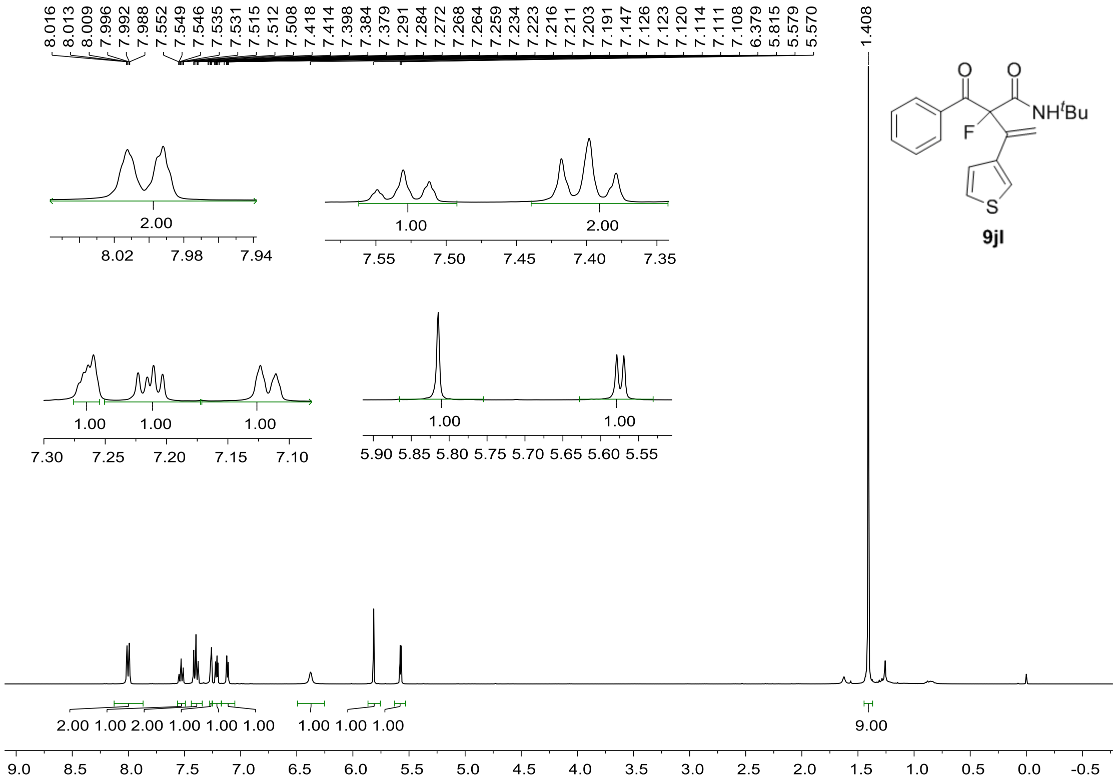

Supplementary Figure 149. ^{1}H NMR spectra for product **9jl**

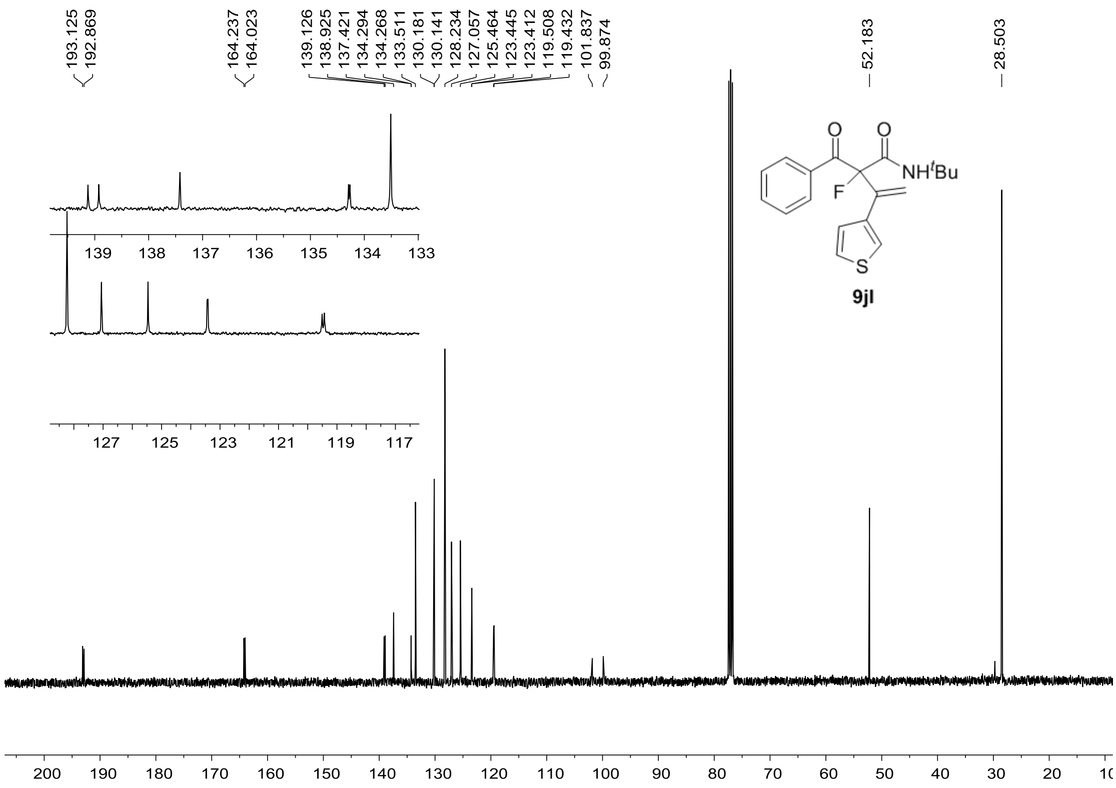

Supplementary Figure 150. ^{13}C NMR spectra for product **9jl**

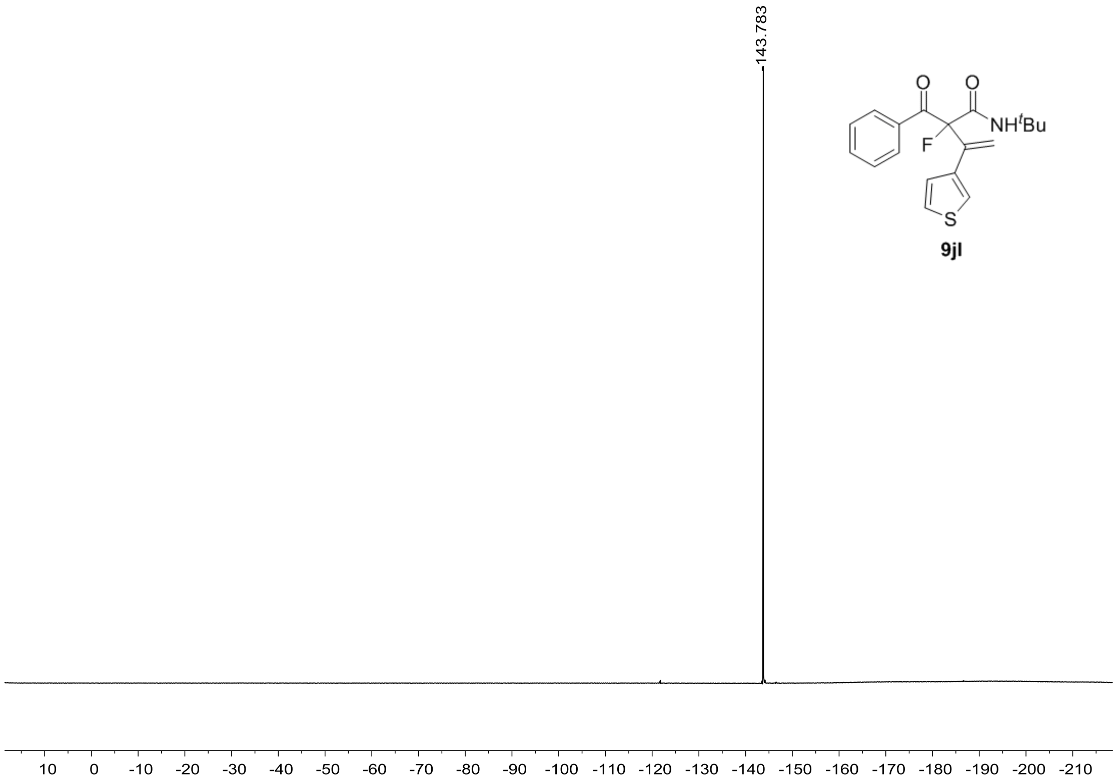

Supplementary Figure 151. ^{19}F NMR spectra for product **9jl**

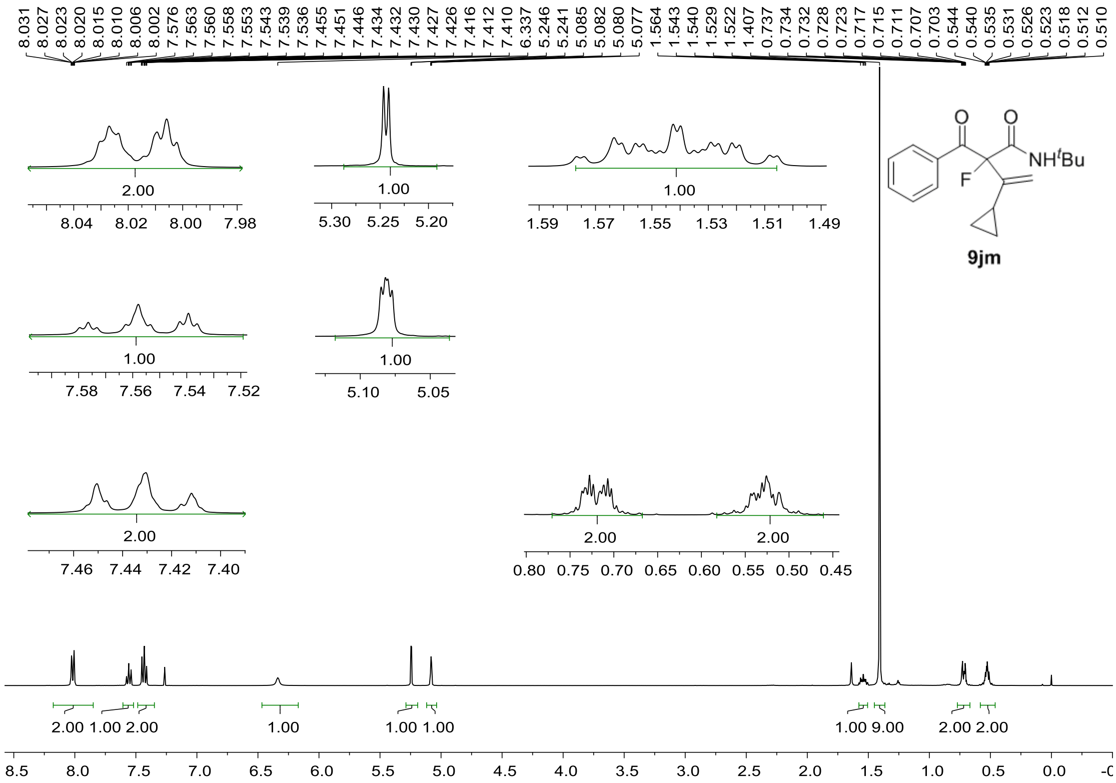

Supplementary Figure 152. ^{1}H NMR spectra for product **9jm**

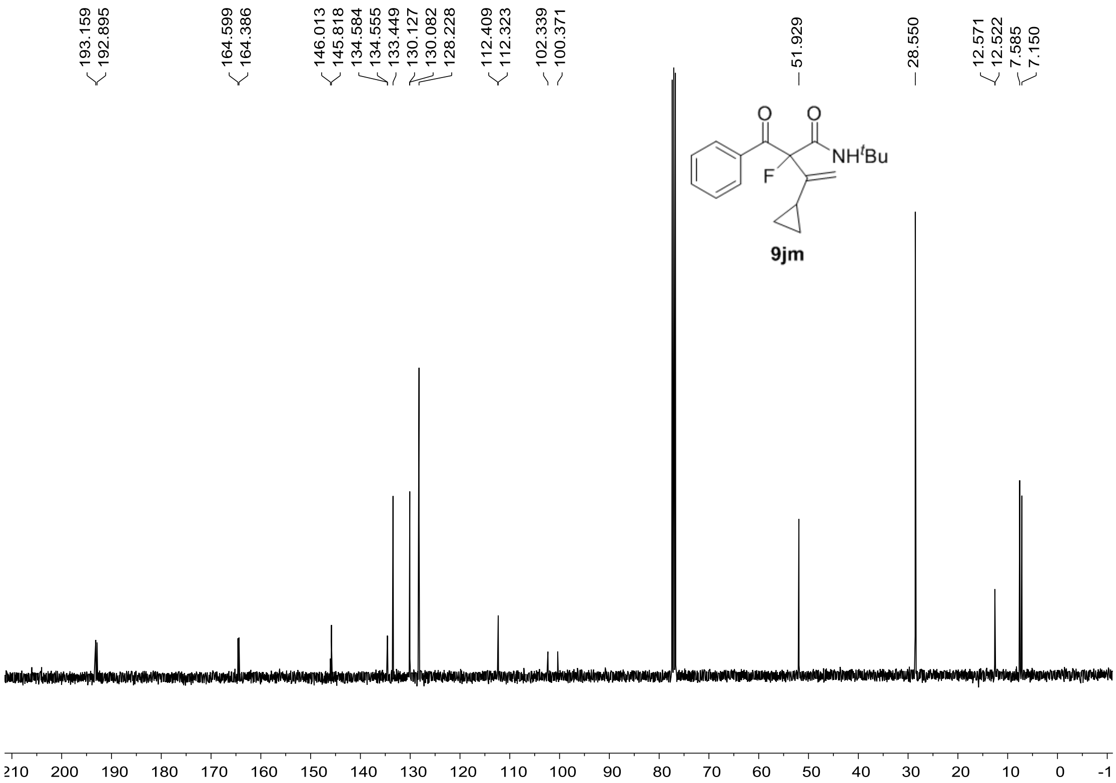

Supplementary Figure 153. ^{13}C NMR spectra for product **9jm**

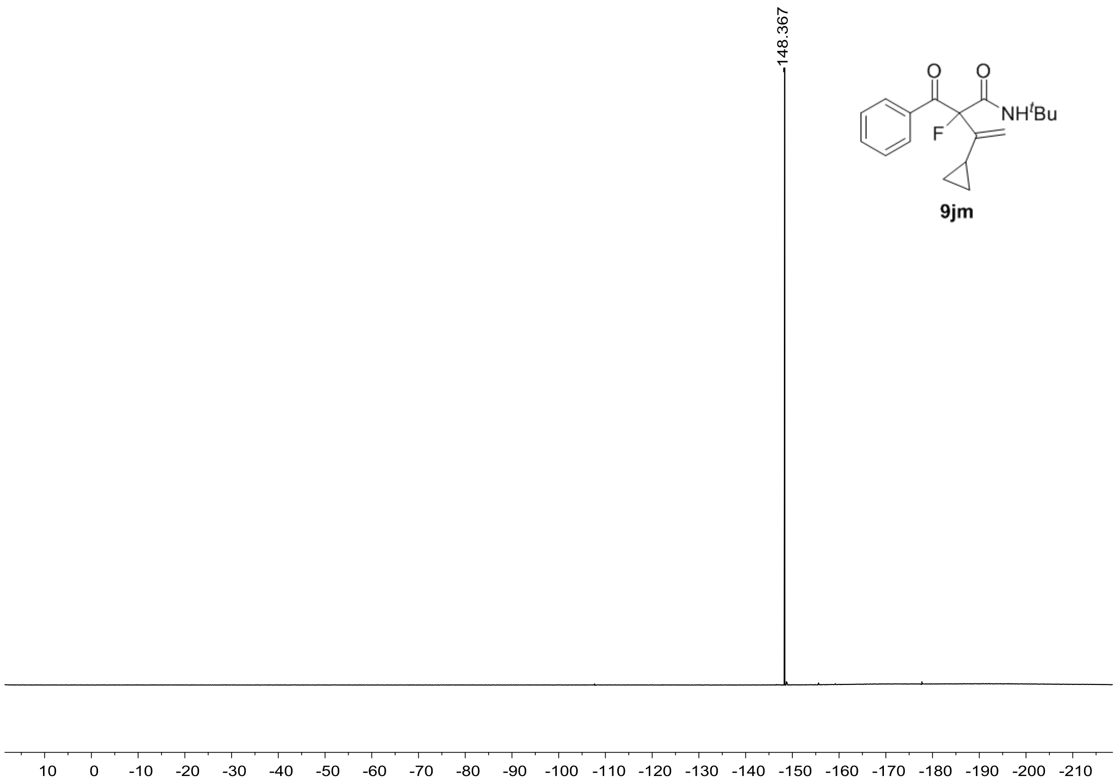

Supplementary Figure 154. ^{19}F NMR spectra for product **9jm**

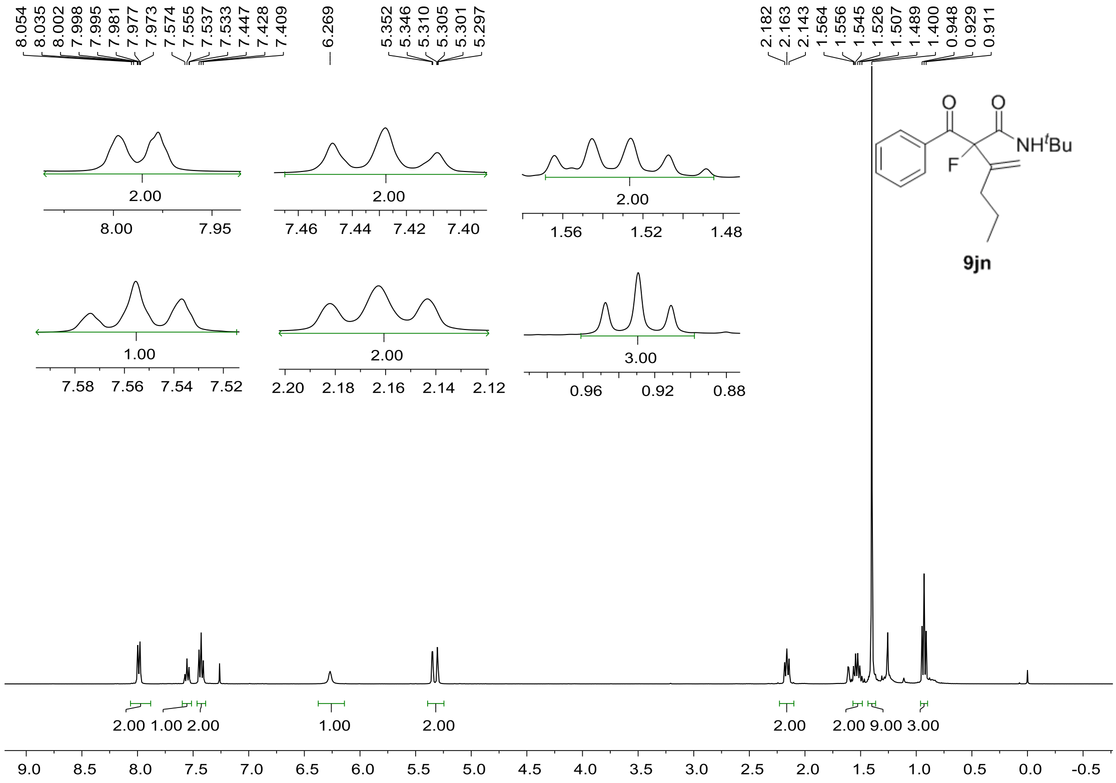

Supplementary Figure 155. ^{1}H NMR spectra for product **9jn**

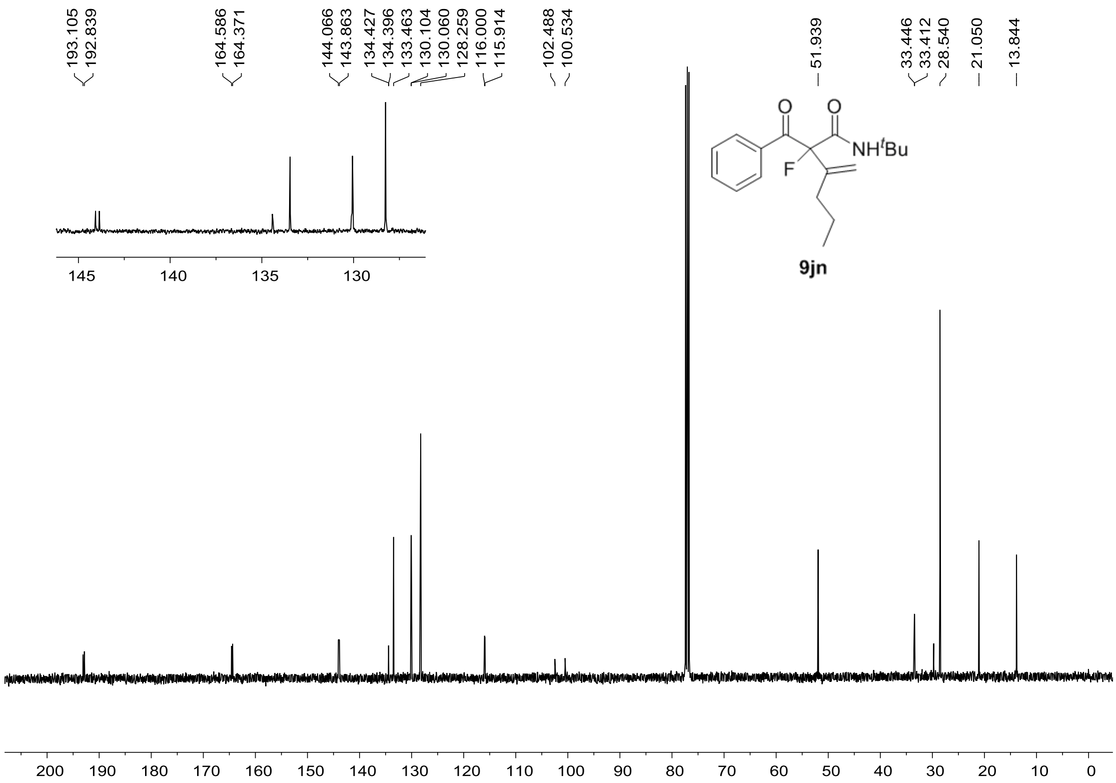

Supplementary Figure 156. ^{13}C NMR spectra for product **9jn**

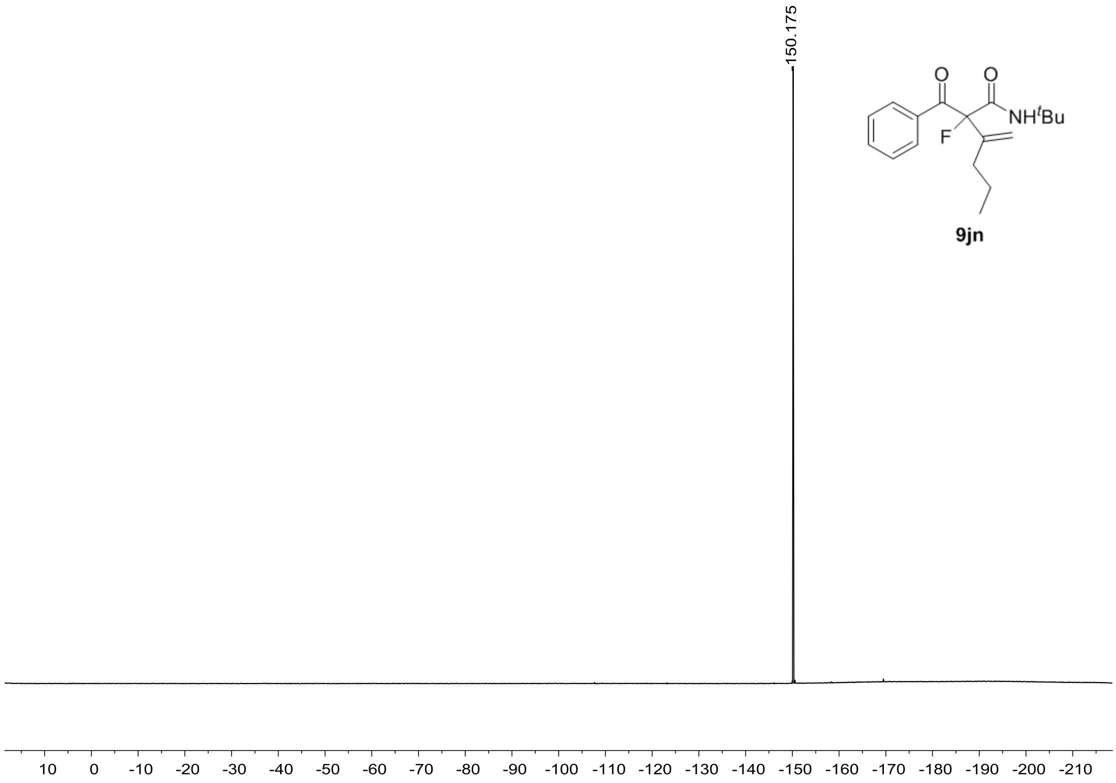

Supplementary Figure 157. ^{19}F NMR spectra for product **9jn**

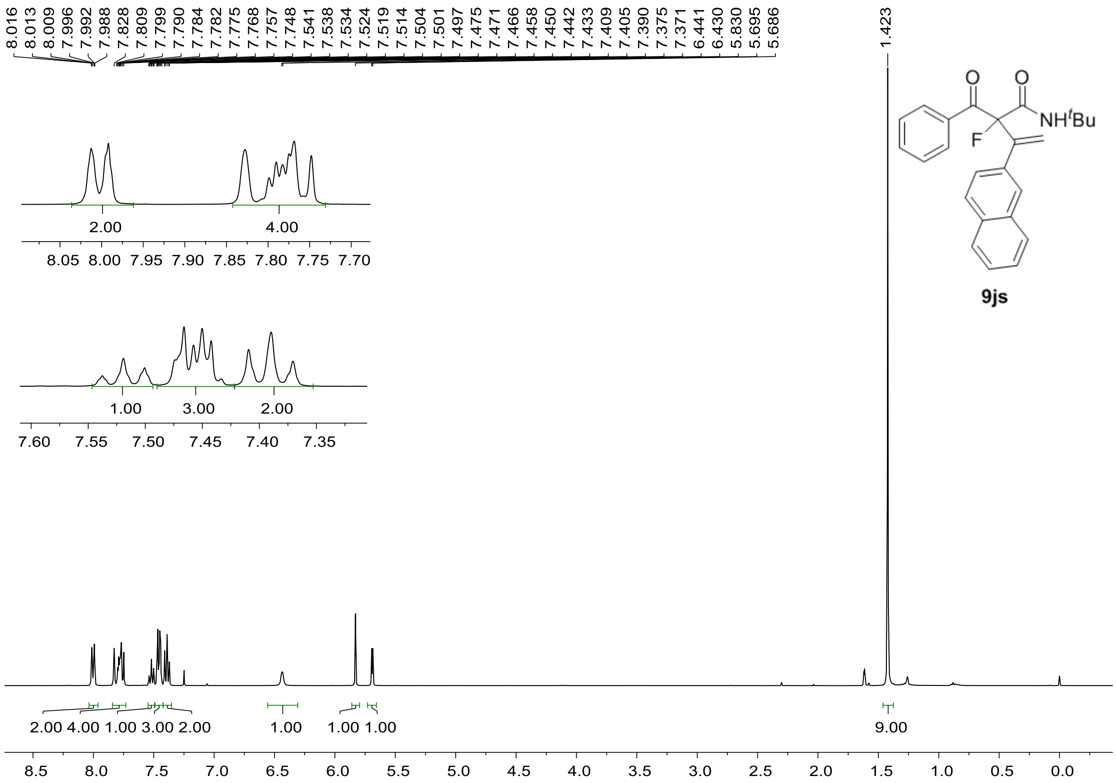

Supplementary Figure 158. ^{1}H NMR spectra for product **9js**

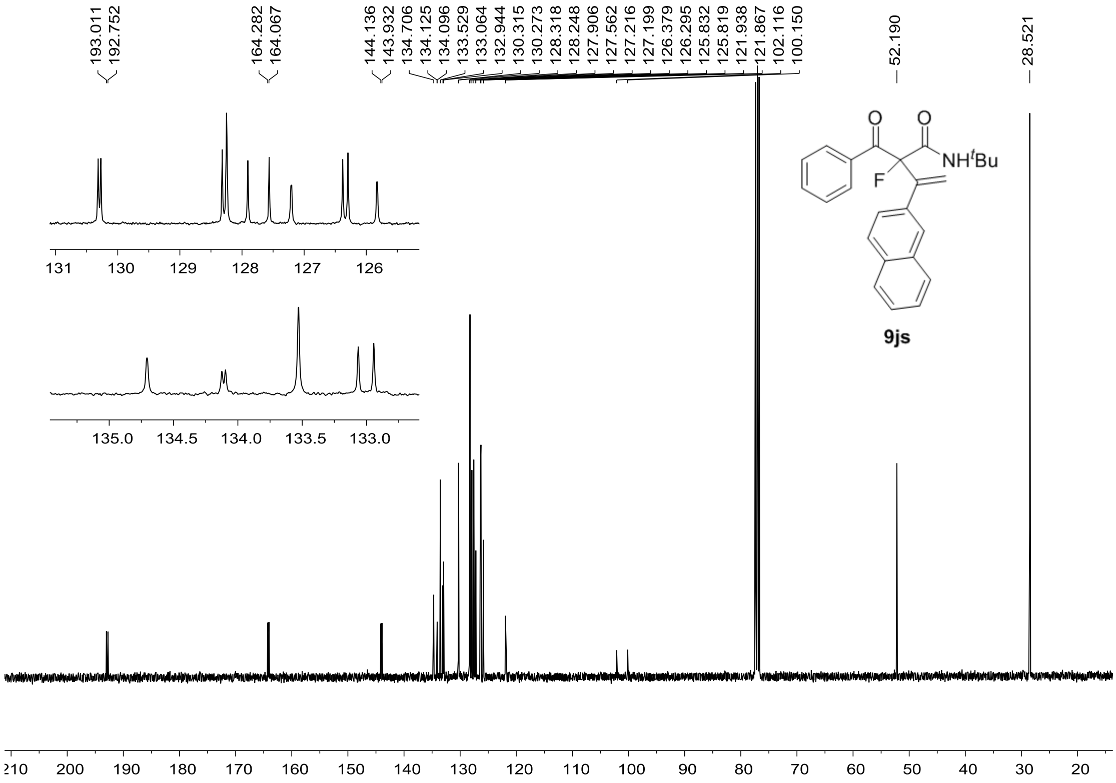

Supplementary Figure 159. ^{13}C NMR spectra for product **9js**

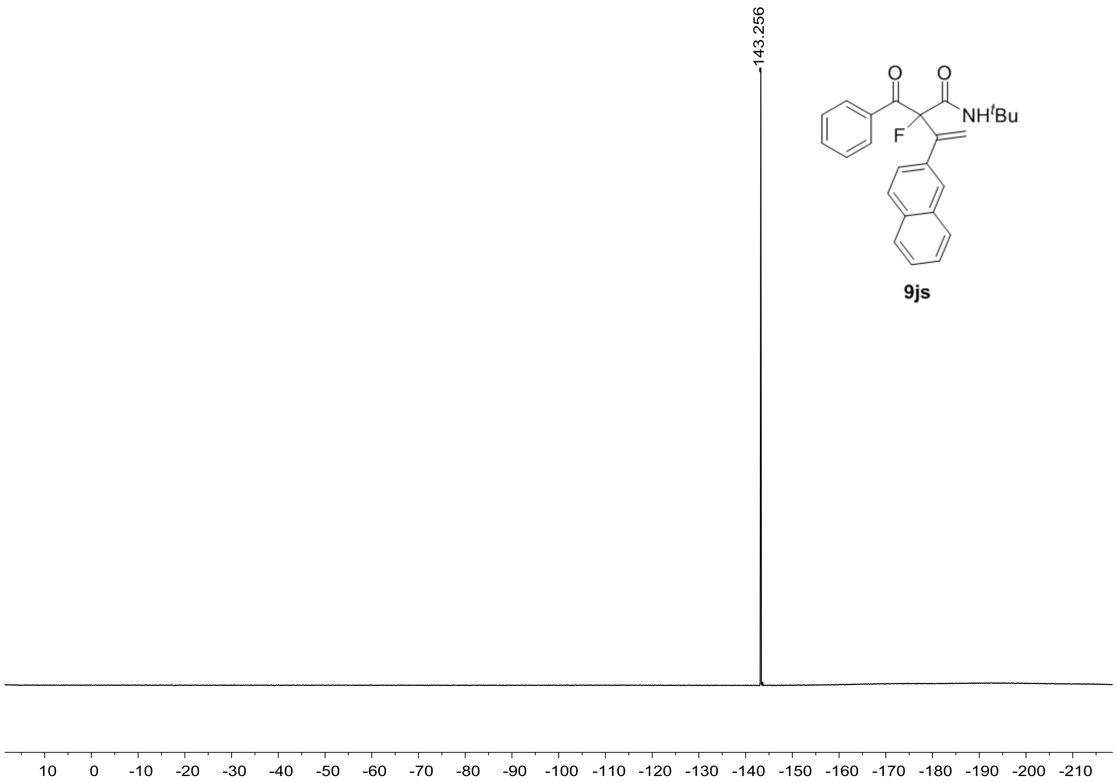

Supplementary Figure 160. ^{19}F NMR spectra for product **9js**

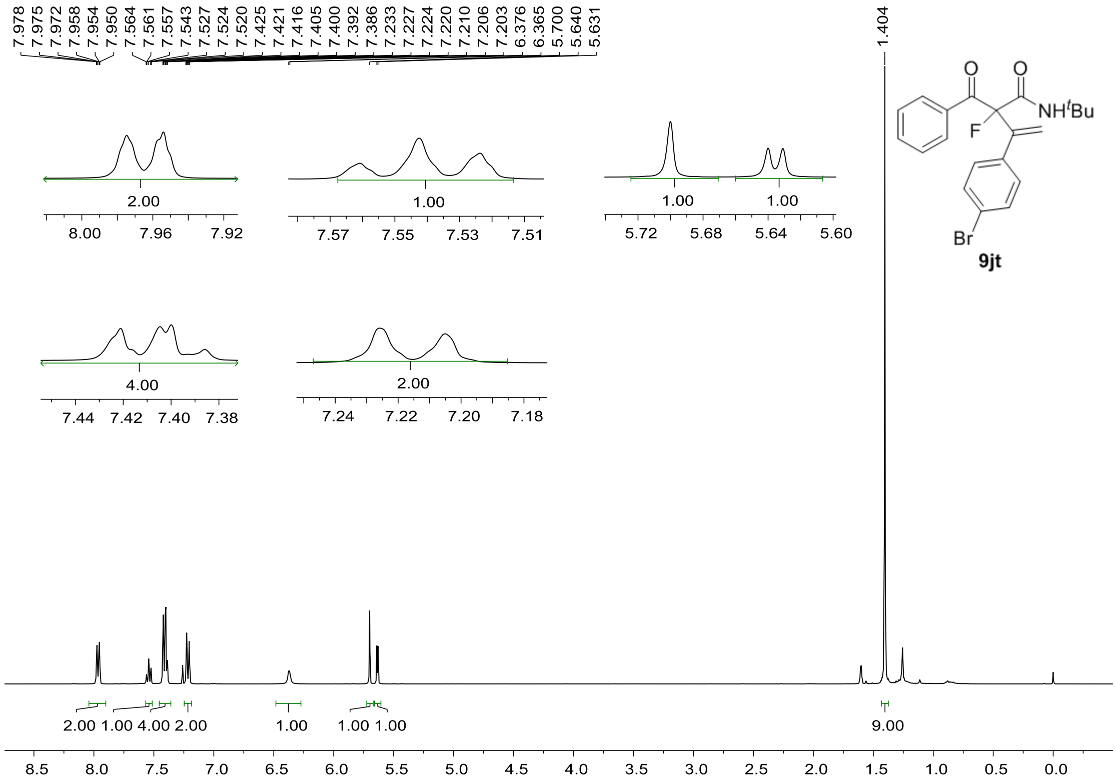

Supplementary Figure 161. ^{1}H NMR spectra for product **9jt**

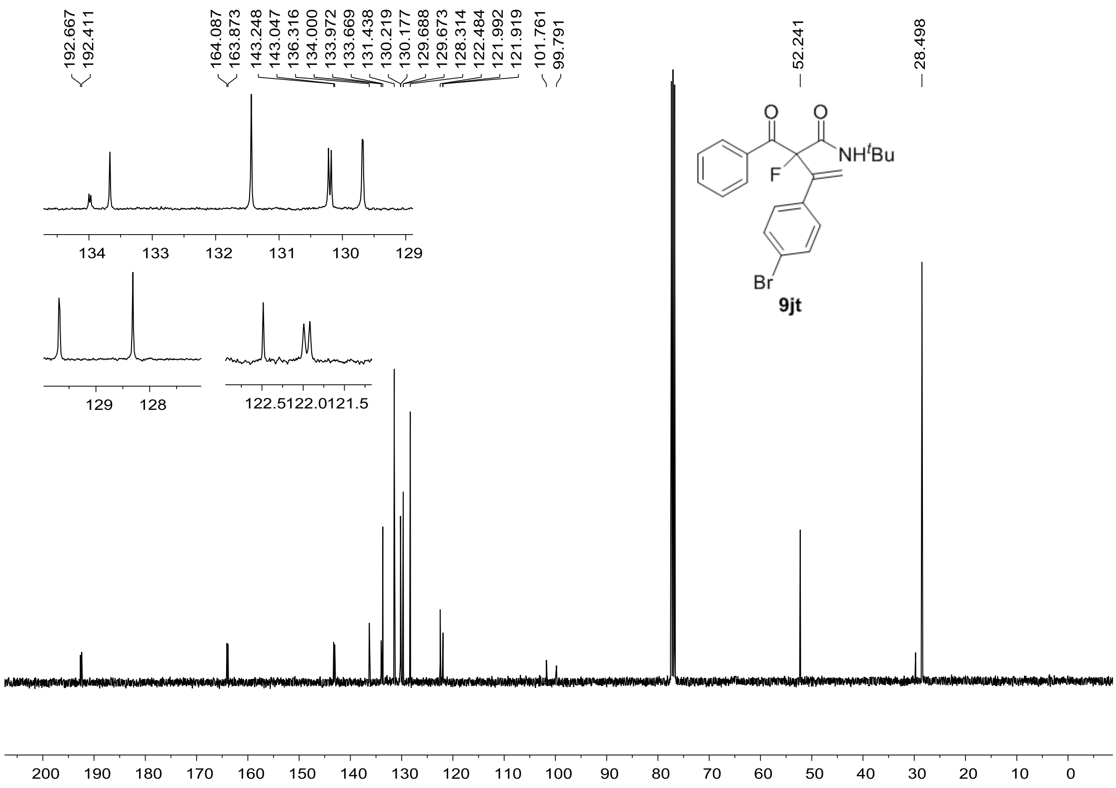

Supplementary Figure 162. ^{13}C NMR spectra for product **9jt**

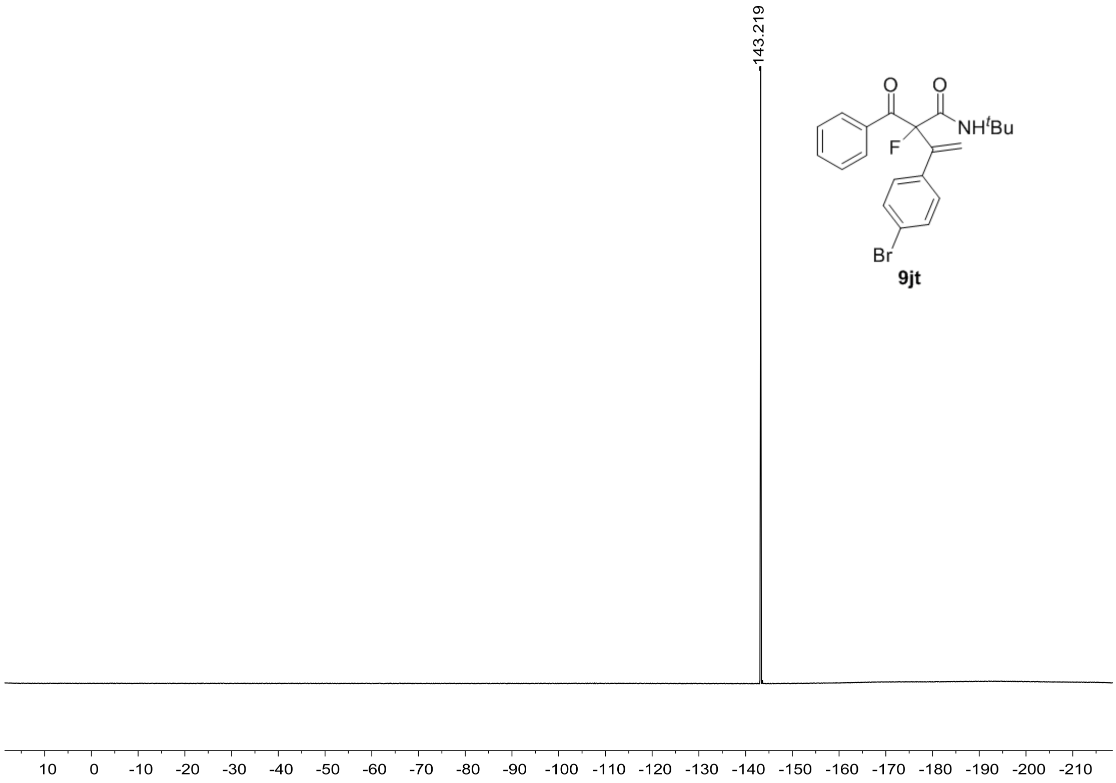

Supplementary Figure 163. ^{19}F NMR spectra for product **9jt**

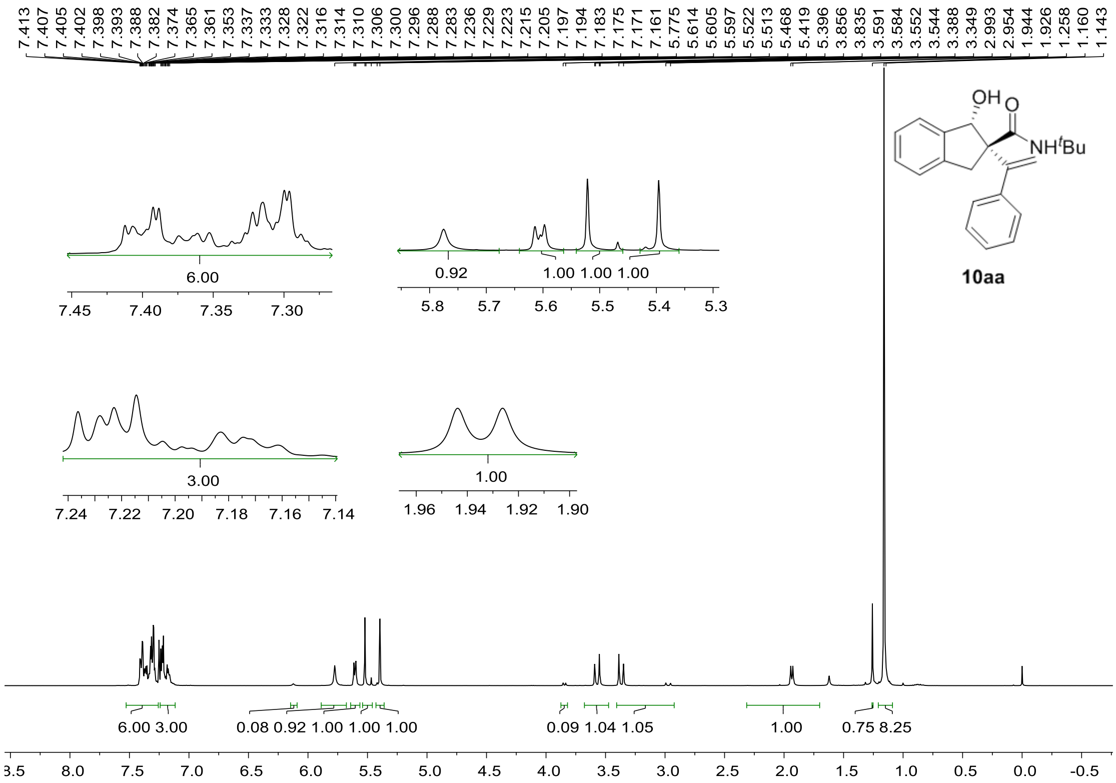

Supplementary Figure 164. ^{1}H NMR spectra for product **10aa**

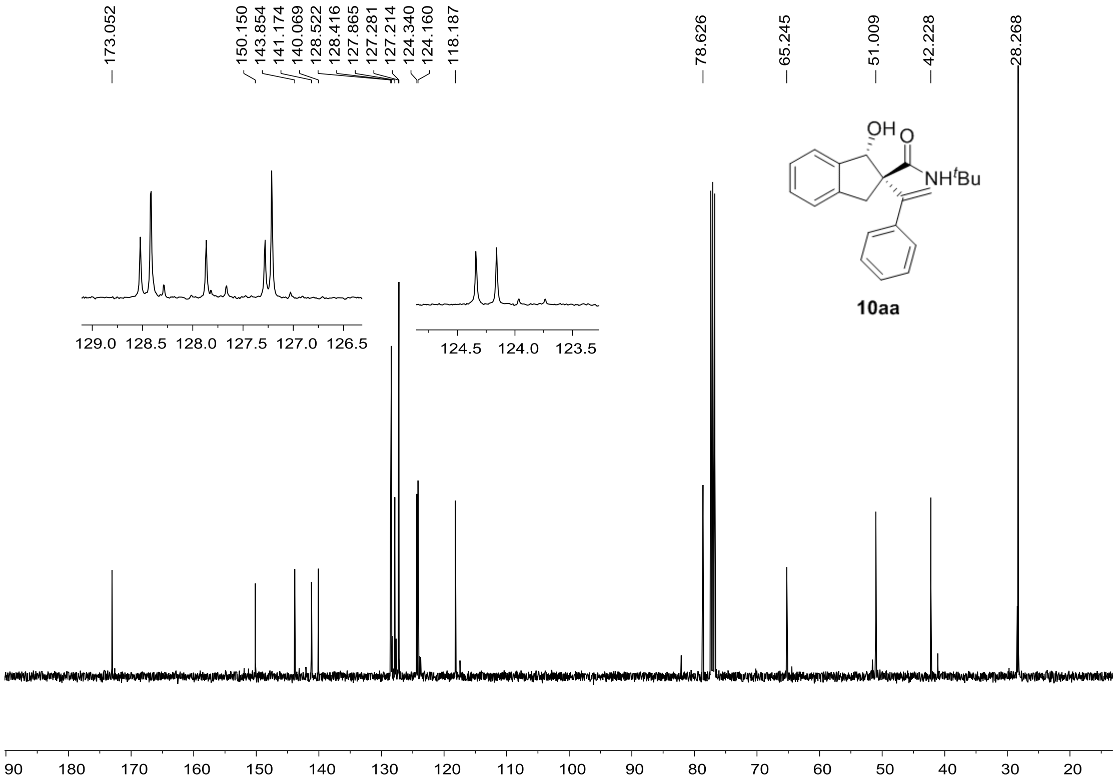

Supplementary Figure 165. ^{13}C NMR spectra for product **10aa**

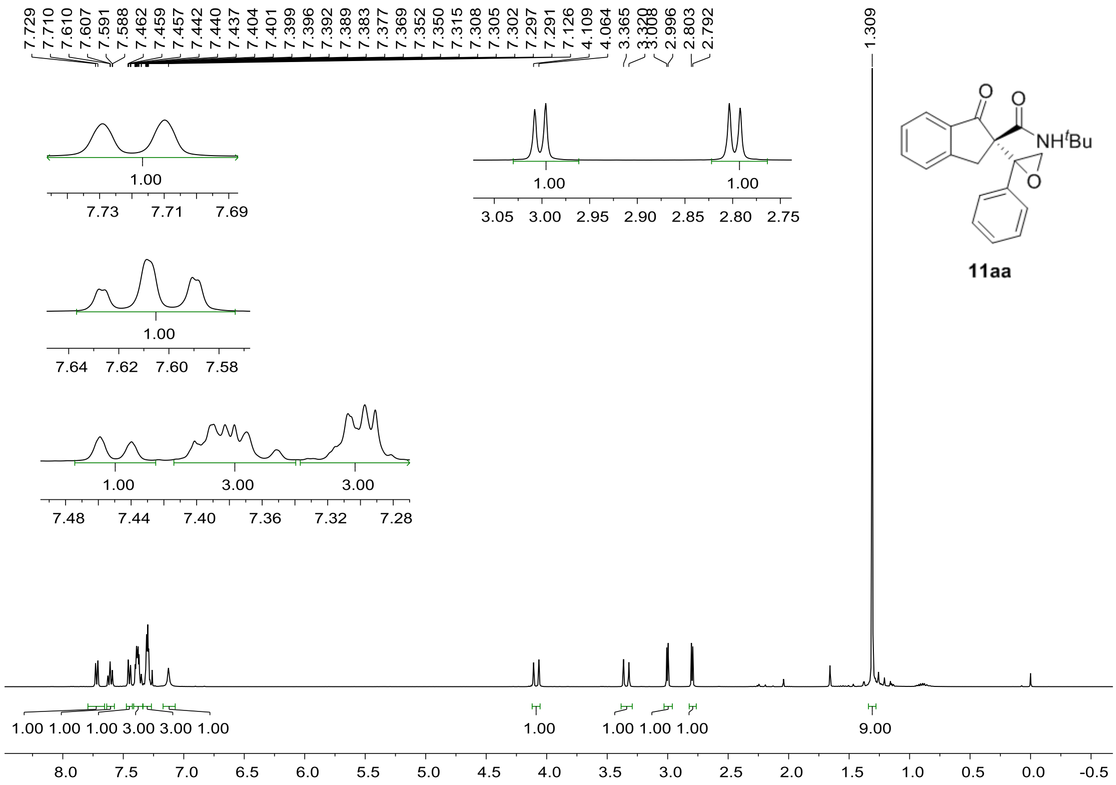

Supplementary Figure 166. ^{1}H NMR spectra for product **11aa**

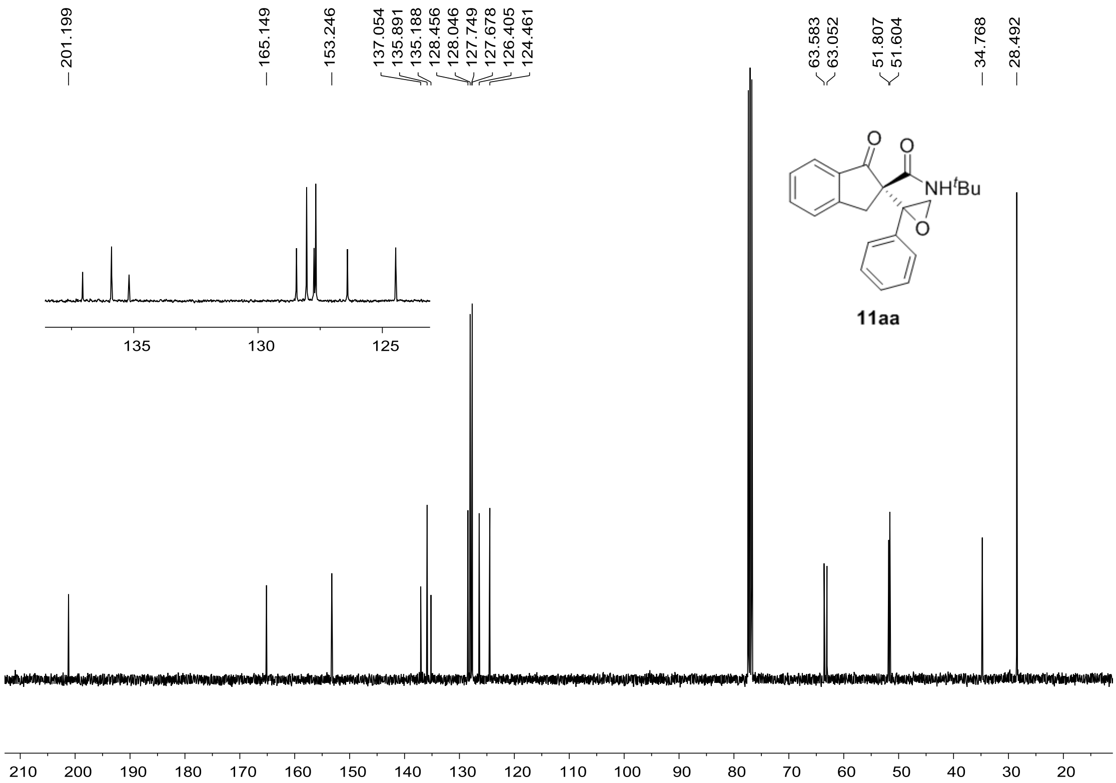

Supplementary Figure 167. ^{13}C NMR spectra for product **11aa**

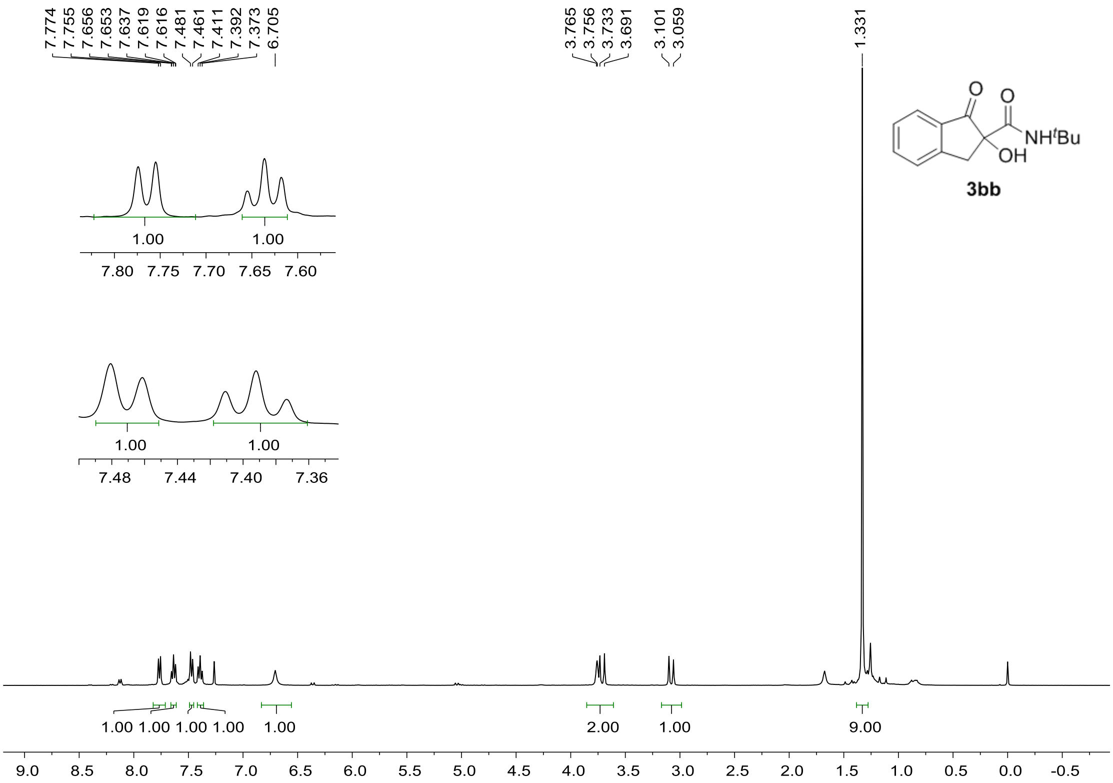

Supplementary Figure 168. ^{1}H NMR spectra for product **3bb**

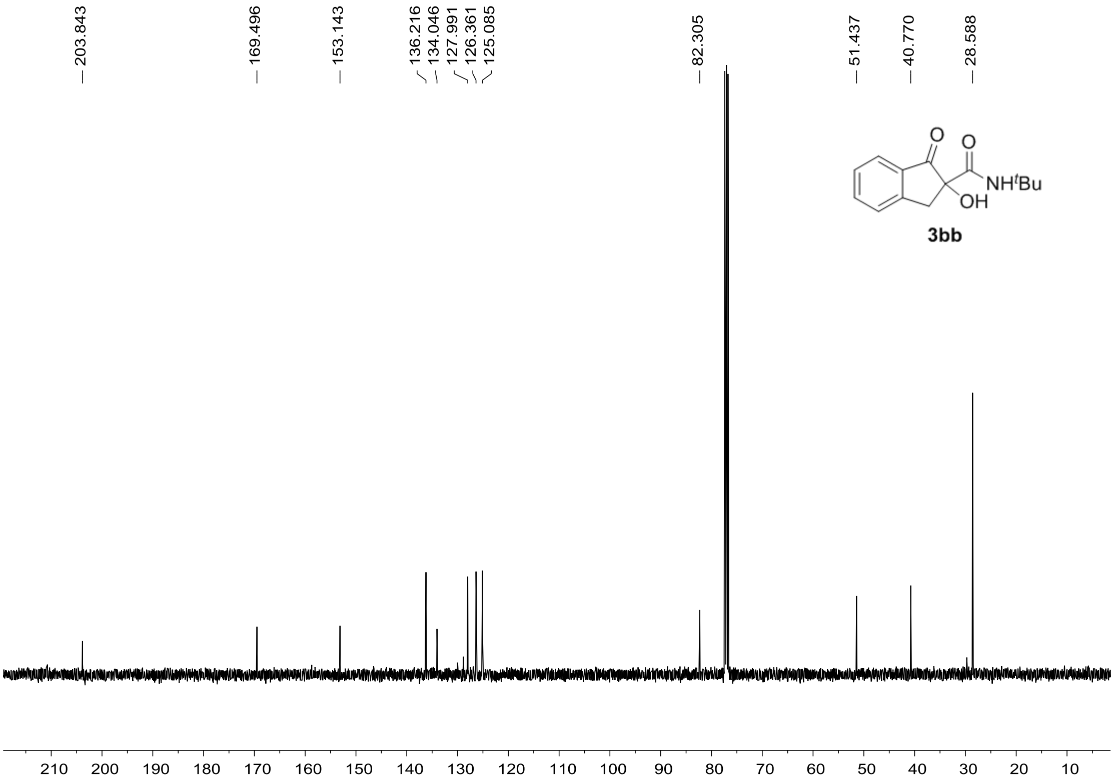

Supplementary Figure 169. ^{13}C NMR spectra for product **3bb**

9. Copy of CD spectra in CH_2Cl_2

3aa

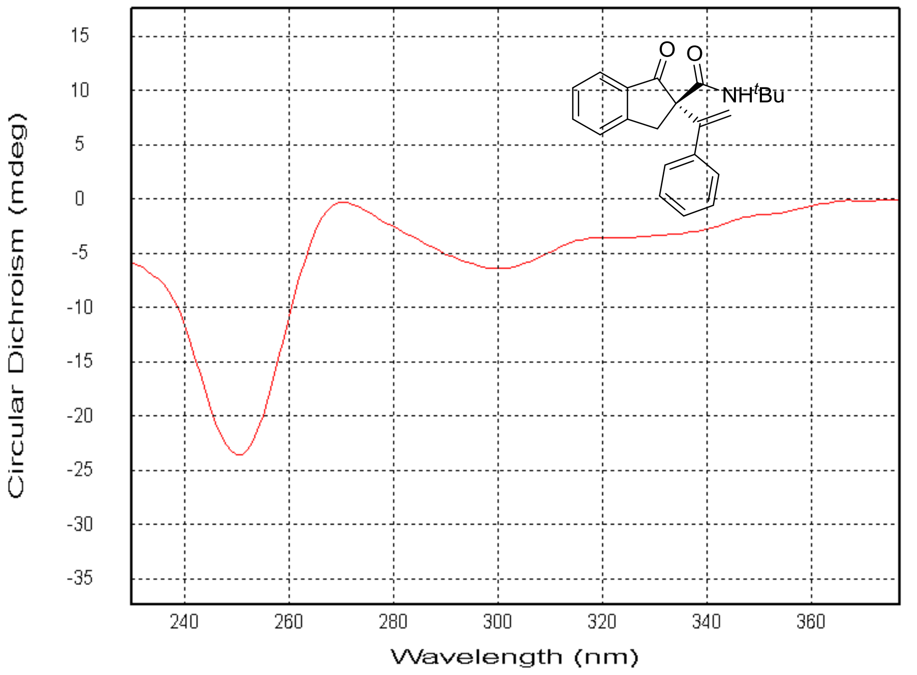

3ba

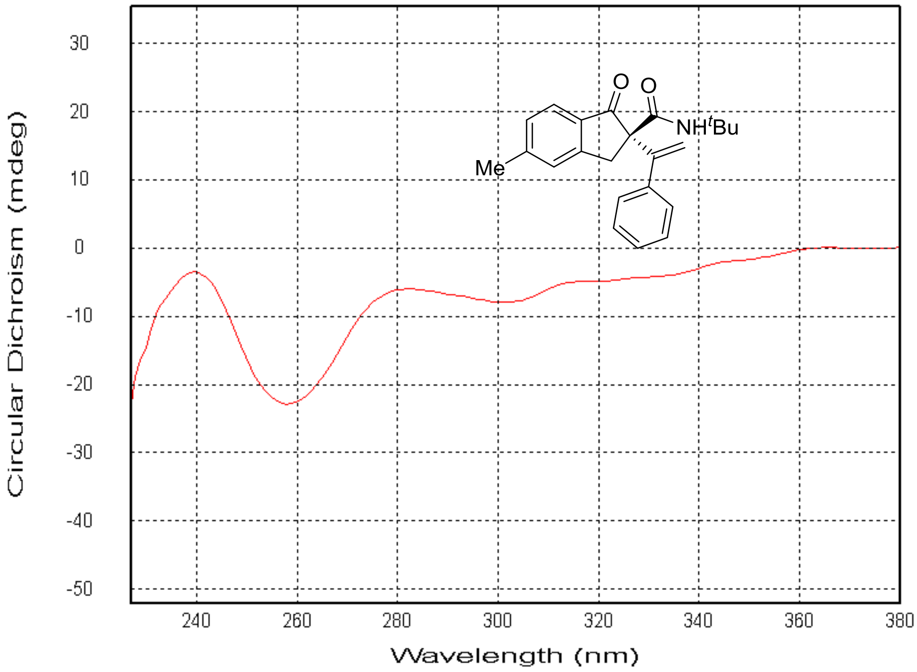

3ca

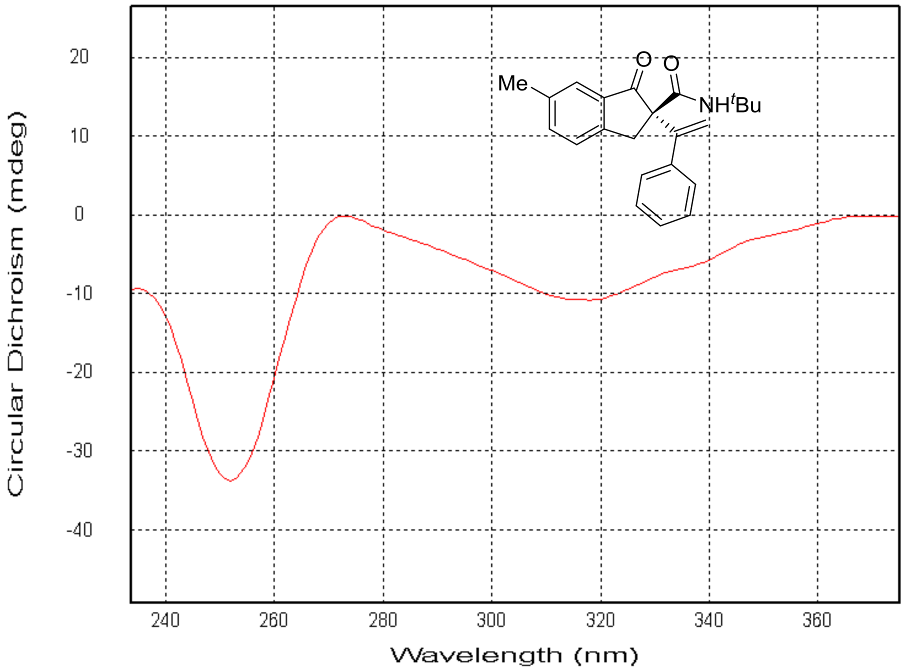
Circular Dichroism (mdeg)
20
10
0
-10
-20
-30
-40
240
260
280
300
320
340
360
Wavelength (nm)
Me
O
O
NH^{t}Bu

3da

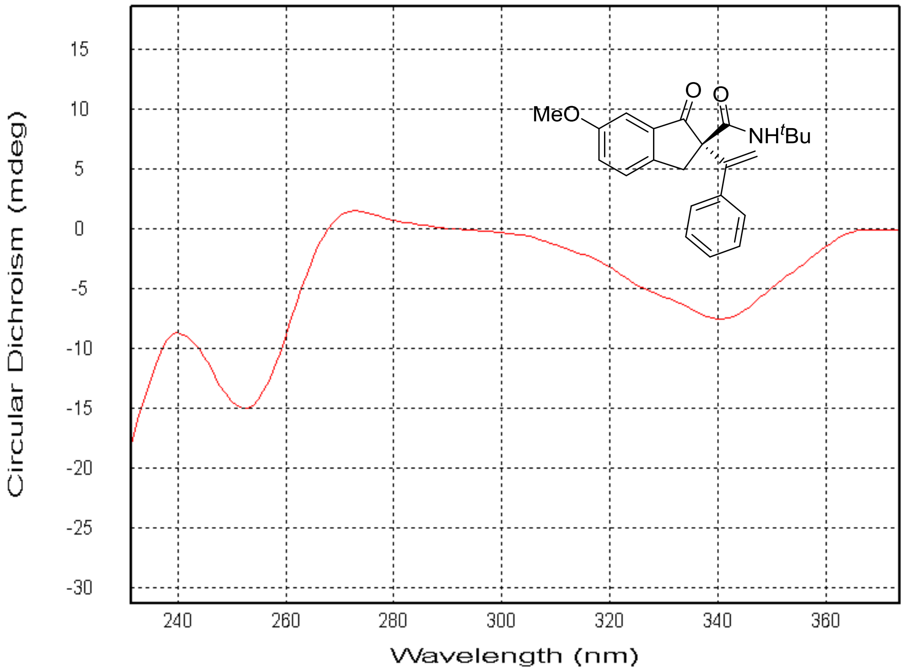
Circular Dichroism (mdeg)
15
10
5
0
-5
-10
-15
-20
-25
-30
240
260
280
300
320
340
360
Wavelength (nm)
MeO
O
O
NH^{t}Bu

3fa

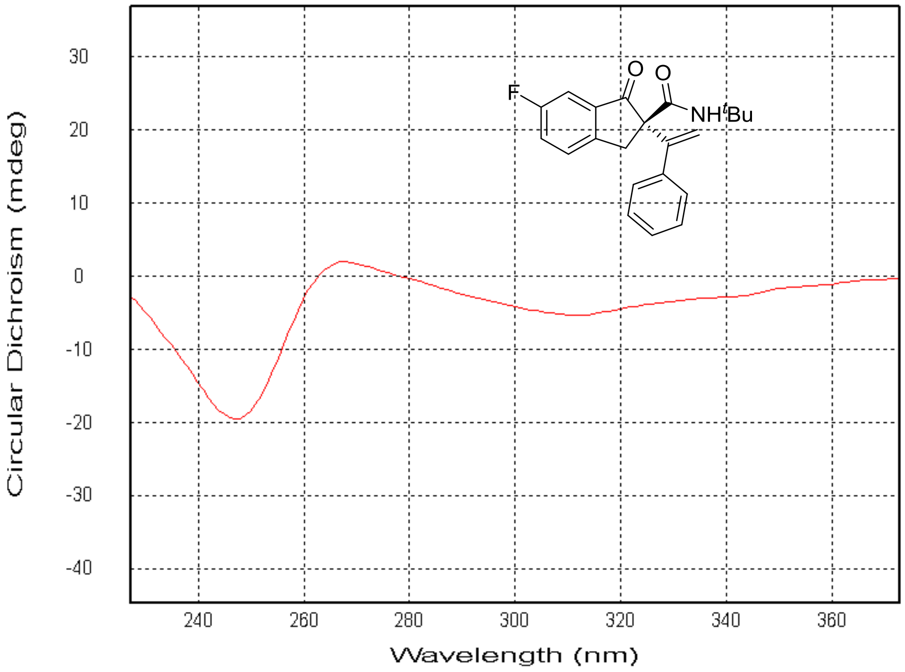

30
20
10
0
-10
-20
-30
-40
Circular Dichroism (mdeg)
240
260
280
300
320
340
360
Wavelength (nm)
F
O
O
NH^{t}Bu

3ga

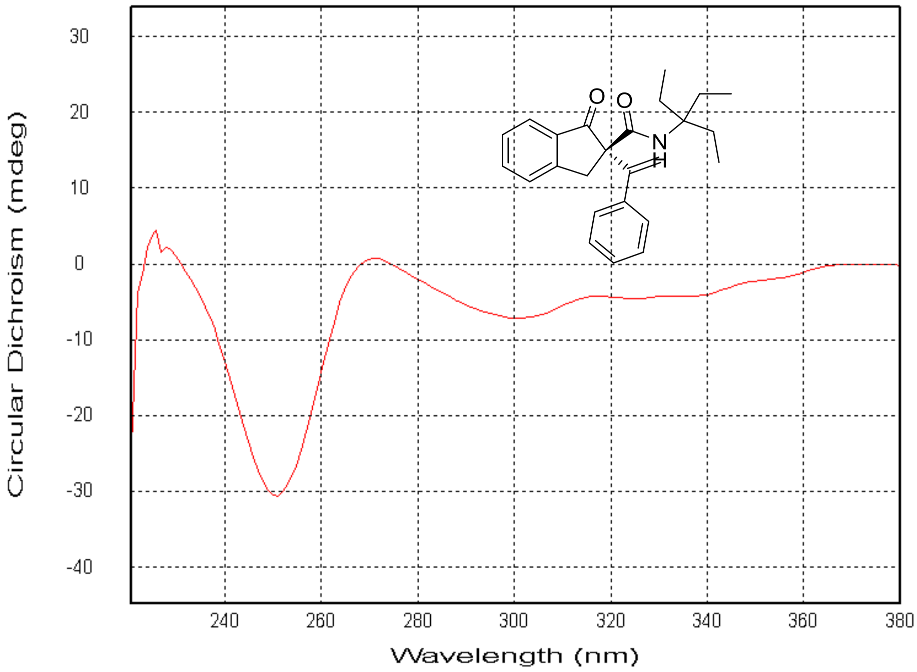

30
20
10
0
-10
-20
-30
-40
Circular Dichroism (mdeg)
240
260
280
300
320
340
360
380
Wavelength (nm)
O
O
N
H

3ab

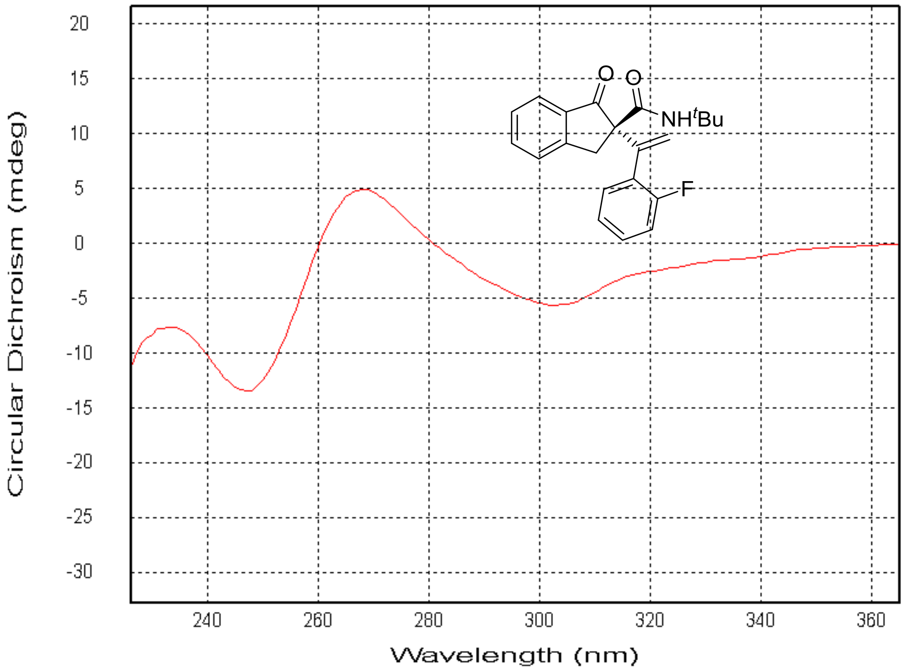
20
15
10
5
0
-5
-10
-15
-20
-25
-30
Circular Dichroism (mdeg)
240
260
280
300
320
340
360
Wavelength (nm)
O
O
NH^{t}Bu
F

3ac

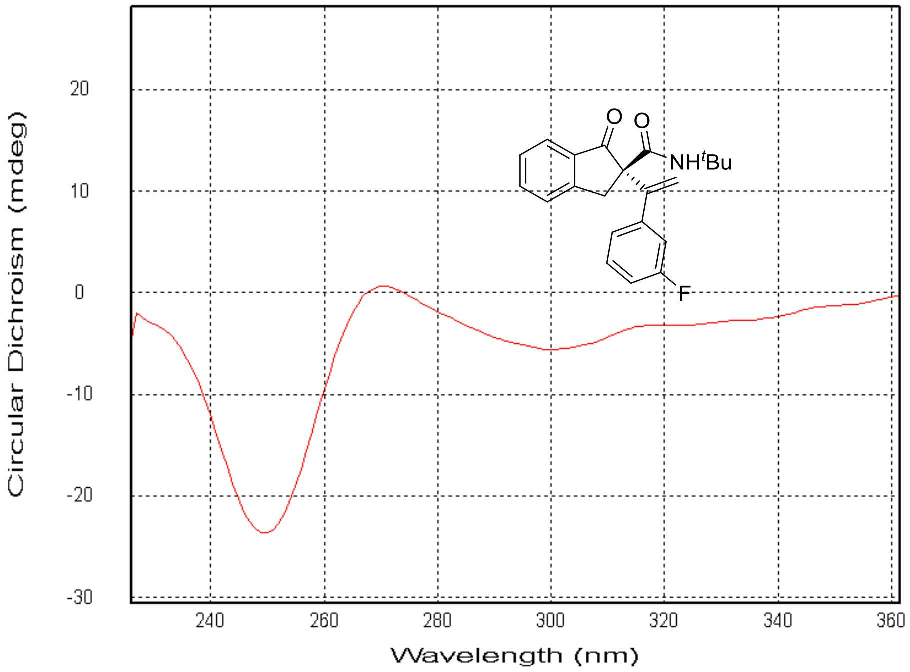
20
10
0
-10
-20
-30
Circular Dichroism (mdeg)
240
260
280
300
320
340
360
Wavelength (nm)
O
O
NH^{t}Bu
F

3ae

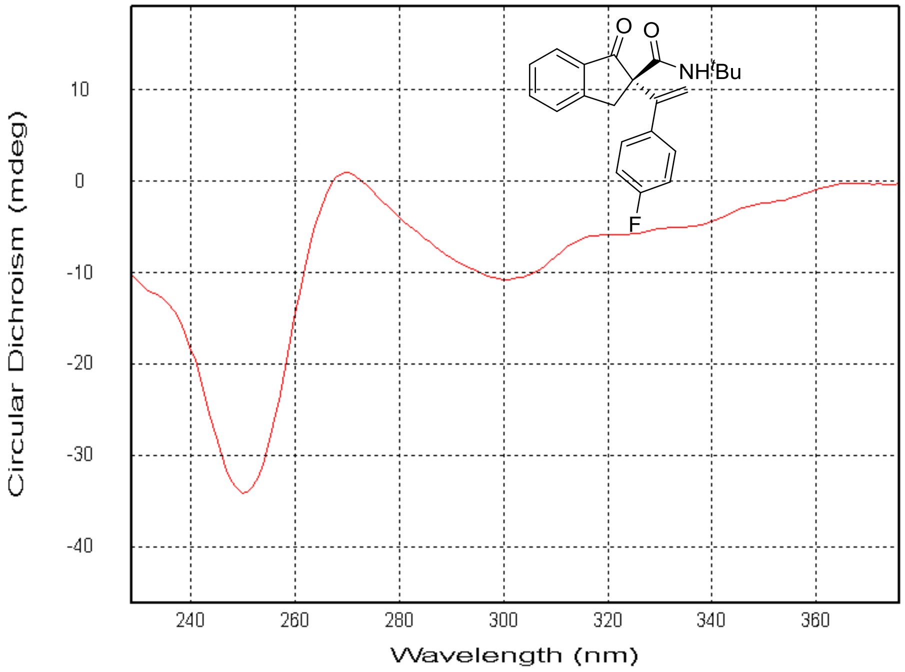

10
0
-10
-20
-30
-40
240
260
280
300
320
340
360
Circular Dichroism (mdeg)
Wavelength (nm)
O
O
NH^{t}Bu
F

3ag

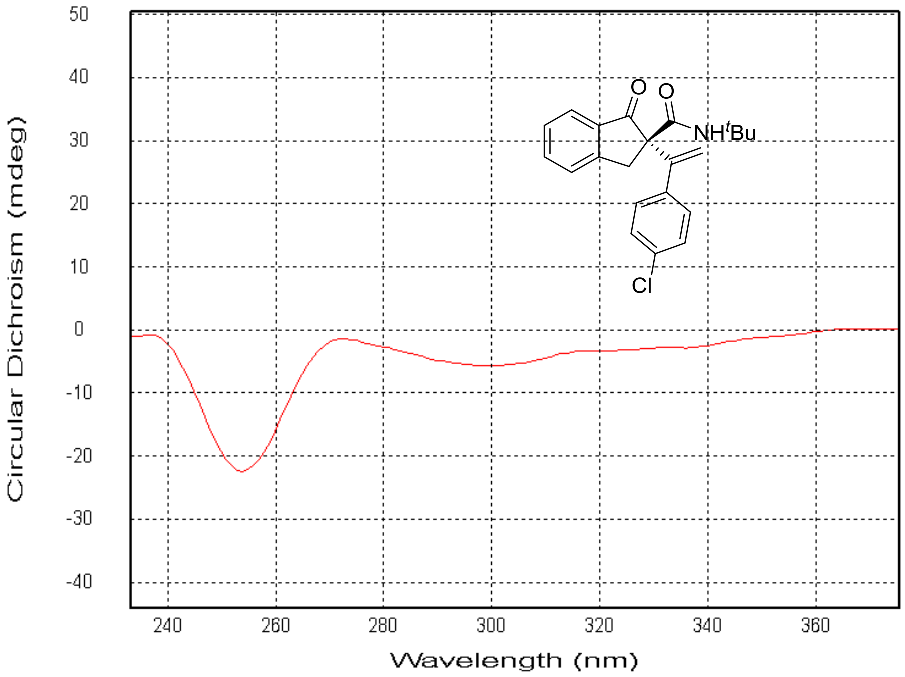

50
40
30
20
10
0
-10
-20
-30
-40
240
260
280
300
320
340
360
Circular Dichroism (mdeg)
Wavelength (nm)
O
O
NH^{t}Bu
Cl

3ah

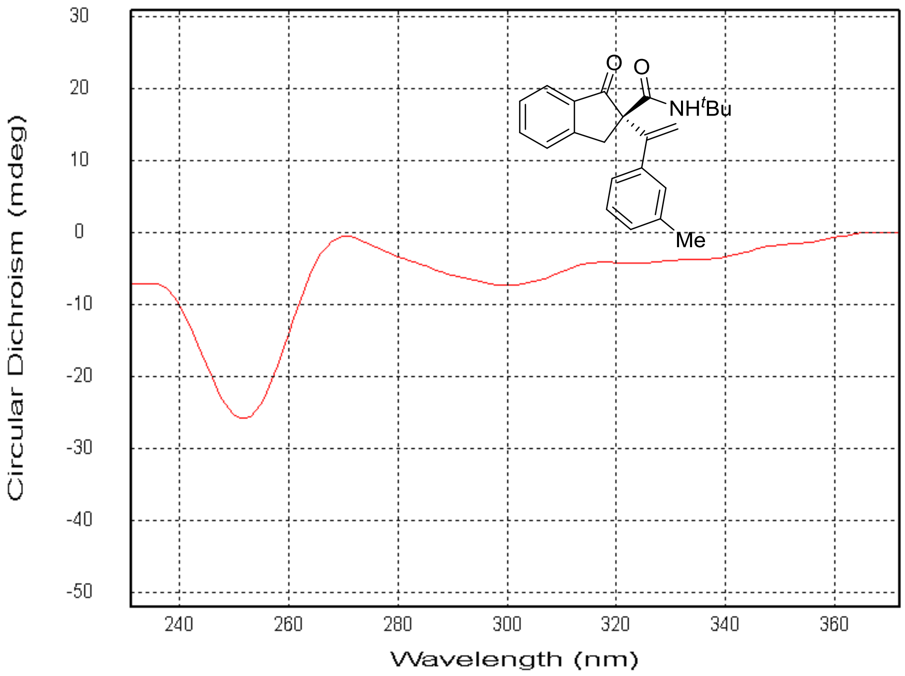

10. Supplementary References

1. Wen, Y. H.; Huang, X.; Huang, J. L.; Xiong, Y.; Qin, B. & Feng, X. M. Asymmetric cyanosilylation of aldehydes catalyzed by novel organo catalysts. *Synlett*. 2445-2448 (2005).
2. Moss, T. A.; Alba, A.; Hepworth, D. & Dixon, D. J. Efficient base catalyzed alkylation reactions with aziridine electrophiles. *Chem. Commun.* 2474-2476 (2008).
3. Zheng, L.-S.; Wei, Y.-L.; Jiang, K.-Z.; Deng, Y.; Zheng, Z.-J. & Xu, L.-W. Enantioselective fluorination of β-ketoamides catalyzed by Ar-BINMOL-derived salan–copper complex. *Adv. Synth. Catal.* **356**, 3769-3776 (2014).
4. Wang, Y. K.; Zheng, Z. H.; Lian, M. M.; Yin, H.; Zhao, J. N.; Meng, Q. W. & Gao, Z. X. Photo-organocatalytic Enantioselective α-hydroxylation of β-keto Esters and β-keto Amides with Oxygen under Phase Transfer Catalysis. *Green. Chemistry*. **18**, 5493-5499 (2016).
5. Kim, J.; Shokova, E.; Tafeenko, V. & Kovalev, V. $(CF_3CO)_2O/CF_3SO_3H$-mediated Synthesis of 1,3-Diketones from Carboxylic acids and Aromatic Ketones. *Beilstein J. Org. Chem.* **10**, 2270-2278 (2014).
6. Tang, L.; Yang, Z.; Jiao, J. C.; Cui, Y.; Zou, G. D.; Zhou, Q. J.; Zhou, Y. Q.; Rao, W. H. & Ma, X. T. Chemoselective Mono- and Difluorination of 1,3-Dicarbonyl Compounds. *Journal of Organic Chemistry*. **84**, 10449-10458 (2019).
7. The X-ray crystallographic coordinate for structure **10aa** reported in this study has been deposited at the Cambriged Crystallographic Data Centre (CCDC), under

deposition number 1989114. The data can be obtained free of charge from The Cambridge Crystallographic Data Centre via https://www.ccdc.cam.ac.uk/structures/.
